# Smart and Multifunctional Materials Based on Electroactive Poly(vinylidene fluoride): Recent Advances and Opportunities in Sensors, Actuators, Energy, Environmental, and Biomedical Applications

**DOI:** 10.1021/acs.chemrev.3c00196

**Published:** 2023-09-20

**Authors:** Carlos M. Costa, Vanessa F. Cardoso, Pedro Martins, Daniela M. Correia, Renato Gonçalves, Pedro Costa, Vitor Correia, Clarisse Ribeiro, Margarida M. Fernandes, Pedro M. Martins, Senentxu Lanceros-Méndez

**Affiliations:** † Physics Centre of Minho and Porto Universities (CF-UM-UP), University of Minho, 4710-057 Braga, Portugal; ‡ Laboratory of Physics for Materials and Emergent Technologies, LapMET, University of Minho, 4710-057 Braga, Portugal; § Institute of Science and Innovation for Bio-Sustainability (IB-S), University of Minho, 4710-057 Braga, Portugal; ∥ CMEMS-UMinho, University of Minho, DEI, Campus de Azurém, 4800-058 Guimarães, Portugal; ⊥ LABBELS-Associate Laboratory, Campus de Gualtar, 4800-058 Braga, Guimarães, Portugal; # Center of Chemistry, University of Minho, 4710-057 Braga, Portugal; ∇ Institute for Polymers and Composites IPC, University of Minho, 4804-533 Guimarães, Portugal; ○ Centre of Molecular and Environmental Biology, University of Minho, Campus de Gualtar, 4710-057 Braga, Portugal; ◆ BCMaterials, Basque Center for Materials, Applications and Nanostructures, UPV/EHU Science Park, 48940 Leioa, Spain; ¶ Ikerbasque, Basque Foundation for Science, 48009 Bilbao, Spain

## Abstract

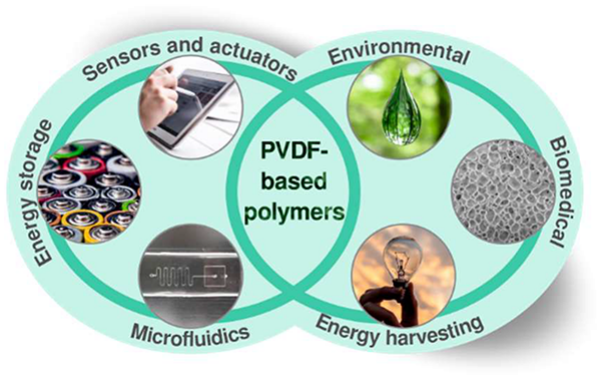

From scientific and technological points of view, poly(vinylidene fluoride), PVDF, is one of the most exciting polymers due to its overall physicochemical characteristics. This polymer can crystalize into five crystalline phases and can be processed in the form of films, fibers, membranes, and specific microstructures, being the physical properties controllable over a wide range through appropriate chemical modifications. Moreover, PVDF-based materials are characterized by excellent chemical, mechanical, thermal, and radiation resistance, and for their outstanding electroactive properties, including high dielectric, piezoelectric, pyroelectric, and ferroelectric response, being the best among polymer systems and thus noteworthy for an increasing number of technologies. This review summarizes and critically discusses the latest advances in PVDF and its copolymers, composites, and blends, including their main characteristics and processability, together with their tailorability and implementation in areas including sensors, actuators, energy harvesting and storage devices, environmental membranes, microfluidic, tissue engineering, and antimicrobial applications. The main conclusions, challenges and future trends concerning materials and application areas are also presented.

## 1 Introduction

In the scope of the circular economy concept, aiming to combine sustainability and social development, a collaborative and interrelated society based on smart technologies is needed to address the urgent and relevant issues raised in the scope of the energy transition, reduction of the environmental impact, novel healthcare paradigms, sustainable mobility, and artificial intelligence, among others.^1,2^ The ongoing technological transitions require advanced, smart, and multifunctional materials to support the Internet of Things (IoT) concept, based on an increasing number of interconnected physical objects, sensors, and actuators, also leading to the Industry 4.0 paradigm, aiming to optimize materials, processes, and products, from concept to manufacturing.^3,4^

Many of the materials required for these technologies are based on polymer, as they present chemical stability, easy processability, tailorable properties, and low cost, some of them also showing electroactive properties such as piezoelectricity, pyroelectricity, and ferroelectricity.^5^ In particular, piezoelectric polymers can convert mechanical to electrical signals or vice versa, a characteristic that is taken to advantage in different areas such as sensors and actuators, biomedicine, energy generation, and storage, among others.^6,7^

Within smart polymer-based materials, poly(vinylidene fluoride), PVDF, and its copolymers, poly(vinylidene fluoride-*co*-hexafluoropropylene) (poly(VDF-*co*-HFP)), poly(vinylidene fluoride-*co*-trifluoroethylene) (poly(VDF-*co*-TrFE)), and poly(vinylidene fluoride-*co*-chlorotrifluoroethylene) (poly(VDF-*co*-CTFE)), stand out based on their high dielectric constant (*ε*′ up to 18), high piezoelectric coefficients (|*d_31_|* up to 30 pC·.N^–1^, |*d*_33_*|* up to 140 pC·N^–1^), high purity, excellent mechanical properties, high resistance against chemicals, suitable thermal resistance, tailorable surface properties and morphology, among others, that depend on the specific crystalline phase of the polymer.^8−13^ Other polymers, such as odd-numbered Nylons,^14^ polylactic acid (PLLA),^15^ poly(lactic-*co*-glycolic acid) (PLGA),^16^ poly(3-hydroxybutyrate-*co*-3-hydroxyvalerate) (PHBV),^17^ and cellulose acetate (CA),^18^ have also emerged and been applied in various technological applications based on their reasonable electroactive properties combined with some other suitable properties including biocompatibility, biodegradability, natural origin, or other relevante characteristics.

The piezoelectric properties of PVDF were discovered by Kawai in 1961 and were attributed to the cooperative alignment of dipoles and charge trapping caused by the high polarization of this polymer in specific phases.^19^ Further, it was demonstrated that the piezoelectricity in PVDF also depends on the electrostriction constant, Poisson ratio, and crystal structure.^20^ In 1961, the first commercially produced PVDF grades were named Kynar of Pennwalt (nowadays Arkema) and were synthesized by the polymerization of vinylidene fluoride (VDF), mainly by aqueous emulsion and suspension polymerization technique.^21^

PVDF and its copolymers are one of the most robust and multifunctional polymeric materials, demonstrating its applicability in a wide variety of applications, including sensors, electronic devices, piezoelectric generators, scaffolds for tissue engineering, and portable analytical devices, among others.^22,23^ The sustainability concerns of this fluorinated polymer are being addressed by its durability and multifunctionality in the applications. More environmentally friendly syntheses and efforts to recover and/or recycle and/or reuse them are also occurring.^24−26^

Considering its properties such as high mechanical strength and durability in harsh environmental conditions, PVDF is one of the most widely used fluoropolymers after poly(tetrafluoroethylene) (PTFE), with a market price around US $14/kg.^27^ Approximately 49% of the market share corresponds to HFP-modified PVDF, a monomer added to further increase polymer flexibility.^27^ Regarding other PVDF-based polymers and their applicability for advanced technological applications, the price will decrease with increasing production scale. Nevertheless, the price of these materials will not be as cheap as PVDF, taking into account the cost of other gas monomers and also the polymerization processes.^25^

In 2021, its market reached a value of ∼880 million dollars, and taking into account the market growth in the electronics sector and the demand for lithium (Li)-ion batteries applied in electric vehicles where PVDF is used as a polymer binder for electrodes, its annual growth rate is expected to increase ∼7% by 2027.^28^

This review focuses on the main properties and processing of PVDF, its copolymers, blends, and composites, together with their main application areas from sensors/actuators to biomedical applications, i.e, high value-added applications, with particular attention to the role of their electroactive properties.

## 2 Poly(vinylidene fluoride): Main Properties and Polymorphism

PVDF is a semicrystalline polymer that crystallizes radially into a spherulitic structure, with its chains being approximately plane-normal. It comprises of a repeating unit (CH_2_–CF_2_) with a spacing of 2.6 Å.^10^ Its dipole moment originates from the electronegative fluorine (δ−) to the electropositive hydrogen (δ+) and it is perpendicular to the polymer chains for the β-phase, while for the other phases, the dipole moment is not perpendicular to the polymer chain, as it will be described in the following.^29,30^

PVDF can crystallize in different crystalline phases, identified as α, β, γ, δ, and ε, depending on the processing conditions.^31^ The most relevant crystalline phases for applications are the α-phase, which is thermodynamically more stable when obtained by cooling from the melt, and the β-phase, which provides the highest electroactive properties: piezo-, pyro-, and ferroelectricity.^32^

**Figure 1:**
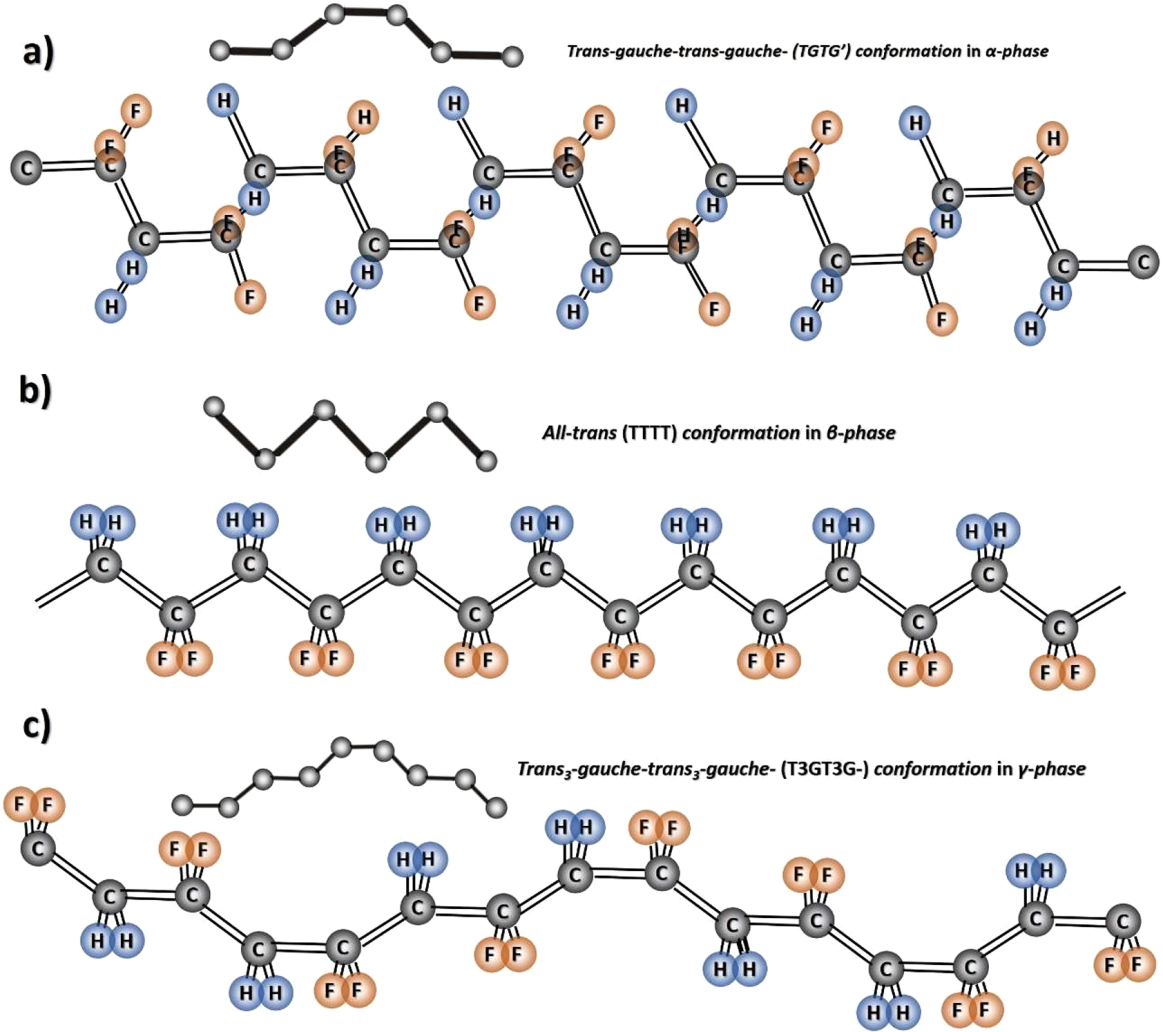
Polymer chain conformation of (a) α-phase, (b) β-phase, and (c) γ-phase of PVDF.

The α-phase is characterized by an orthorhombic unit cell with a *P*2*cm* space group, the polymer chains being organized in a trans-gauche (TGTG^–^) conformational structure, as shown in Figure 1a). The dipole moment of the conformation repeat unit is 4.0 × 10^–28^ cm·C.^33^ The β-phase is also organized in orthorhombic unit cells, but with a *Cm*2*m* space group. In this phase, the chain conformation is planar zigzag (all-trans), as shown in Figure 1b), with the dipolar moments of the chains parallel to the crystallographic *b*-axis.^34^ The conformation repeat unit of the β-phase shows a dipolar moment of 7.0 × 10^–28^ cm·C and polarization of 131 mC·m^–2^. The δ-phase of PVDF is the polar version of the α-phase by the application of a high electric field, resulting in the inversion of the dipole moments along the applied field. The chain conformation is similar to the α-phase, Figure 1a. PVDF in the γ-phase reveals an intermediate conformation between β- and α-phases (TTTG^+^TTTG^–^)^35^ (Figure 1c). Furthermore, the ε-phase is quite difficult to obtain and its conformation is similar to the γ-phase. Due to those conformational characteristics, the α- and the ε-phases are nonpolar, whereas the, β-, γ-, and δ-phases are polar.^29,36^

The identification of the different crystalline phases of PVDF is typically achieved by X-ray diffraction and Fourier-transform infrared spectroscopy (FTIR) techniques.^11^

PVDF is characterized by a glass transition temperature at *T*_*g*_ −34 °C, a degree of crystallinity between 35 and 60%, and a melting temperature between 160 and 190 °C, depending on the processing conditions and crystalline phases. Furthermore, the thermal degradation temperature is between 400 to 450 °C independent of the crystalline phase, the degree of crystallinity, and the processing method. In addition, the number of head-to-head defects is between 6% and 9%, as determined by nuclear magnetic resonance (NMR).^37^

PVDF is characterized by chemical, mechanical, radiation, and thermal resistance, due to the high electronegativity of the fluorine atoms in the chain and the high bond dissociation energy of the C–F bond.^38^

Regarding the electrical properties, the ε′ of PVDF is between 7 and 13, depending on the crystalline phase, the crystalline phase content and the crystalline domain size.^39^ The dielectric constant of the β-phase is highest when compared to the α- and γ-phases due to the higher polarity of this phase. Another critical parameter that contributes to the dielectric properties is the interfaces between the amorphous and crystalline regions.^39^ Also, a region defined as oriented amorphous fraction (OAF) that connects mobile amorphous fraction and the lamellar crystal, participates in ferroelectric switching of PVDF and enhances its β-phase, dielectric, and ferroelectric properties.^40,41^

The processing annealing temperature and time allow tuning the dielectric and piezoelectric characteristics of PVDF, the dielectric and piezoelectric responses decreasing strongly in the first 4 h at temperatures above 80 °C.^42^

The dielectric behavior as a function of frequency and temperature shows two main relaxation processes dominated by the β-relaxation that corresponds to the *T*_*g*_, attributed to cooperative segmental movements of the main chains within the amorphous and amorphous/crystalline interface regions of the polymer. The other main relaxation process is identified as α- or α_c_-relaxation, it occurs at temperatures above 60 °C, and it is associated with cooperative molecular motions within the crystalline fraction.^43^ These relaxations can also be observed by dynamical mechanical analysis. The poling process, the application of an electric field to provide orientation to the dipolar moments, affects the dynamics of these relaxation processes.^43^

The displacement (*D*)-electric field (*E*) hysteresis loops are also strongly dependent on crystal phase and crystallinity. They are affected by the processing conditions and thermal treatments, in particular in terms of maximum and remnant polarization.^39^ The irreversible polarization depends on the amorphous phase, as increasing amorphous phase content leads to more free space for the inversion of the crystalline domains.^39^

PVDF is characterized by excellent mechanical properties with a Young modulus >1.5 GPa, and the poling process increases the mechanical response along the preferred microstructure orientation. Also, as the temperature increases, the poling effect on mechanical behavior is reduced due to increased molecular mobility.^44^ The mechanical properties are also determined by the polymer’s morphology and degree of crystallinity and, therefore, by the processing conditions.^8^

The optical properties of PVDF are dependent on polymer microstructure. For a compact and dense morphology, the optical transmittance is between 80 and 90% in the visible spectrum with a refractive index between 1.39 and 1.47.^45,46^

Considering its excellent thermal, mechanical, electrical, and electroactive properties and also its tunability to be implemented in a wide range of application requirements, substantial efforts have been devoted to develop a variety of processing techniques and conditions with a focus on improving materials integration and device performance.

### 2.1 Processability

As referred previously, PVDF is a polymer with notable polymorphism, showing five main crystalline phases: α, β, γ, δ, and ε, depending on the processing conditions (mainly, processing temperature and time).^47^ From a technological point of view, the β-phase is the one with the highest piezoelectric, pyroelectric, and ferroelectric response, and it is the most implemented in applications where an electric response or a deformation (actuation) are required. In turn, the α-phase is the most stable thermodynamically when directly obtained from the melt.^48^

**Figure 2:**
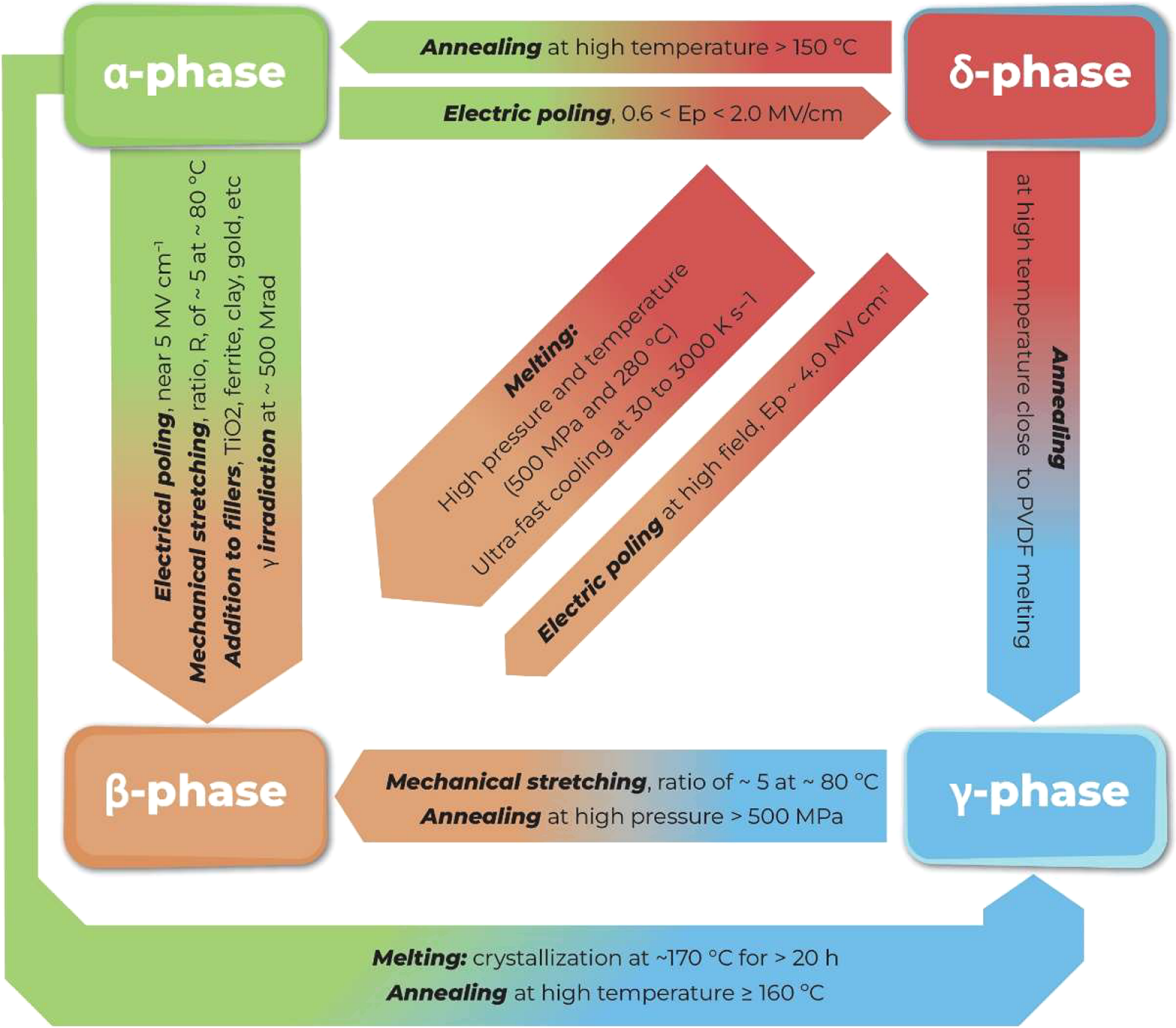
Main processing treatments and conditions to obtain the different PVDF phases.

Figure 2 shows the main processing treatments to obtain the different phases of PVDF.

When processed above the temperature of 110 °C, the predominant phase is the α-phase. The β-phase is obtained from solution processing with polar solvent at crystallization temperatures below 70 °C. The γ-phase is typically obtained by annealing and mechanical stretching processed from other polymer phases.^49^

The β-phase can be induced from the α-phase through different processing strategies, such as mechanical stretching, annealing, cooling, pressing, the addition of different fillers and polymers, and electrical poling (Ep), among others.^50−52^

The merits of PVDF are not only based on their chemical, mechanical, and thermal stability, together with tunable electroactive properties but also on allowing it to be produced in a variety of designs and morphologies such as nonporous (dense) films, porous (membrane) films, fibers, microspheres, patterned/three-dimensional (3D) formats by processing techniques including extrusion,^53^ injection molding,^54^ electrospinning,^55^ phase separation processes, particulate leaching, freeze extraction, or printing technologies, among others.^56^ Thus, over the past decades, a large variety of processing methods have been developed to produce PVDF in specific shapes to meet specific application requirements, including sensors and actuators,^57,58^ energy storage,^59^ filtration membranes,^60^ microfluidics,^61^ tissue engineering,^62^ or drug delivery,^63^ among others.^8^

**Figure 3:**
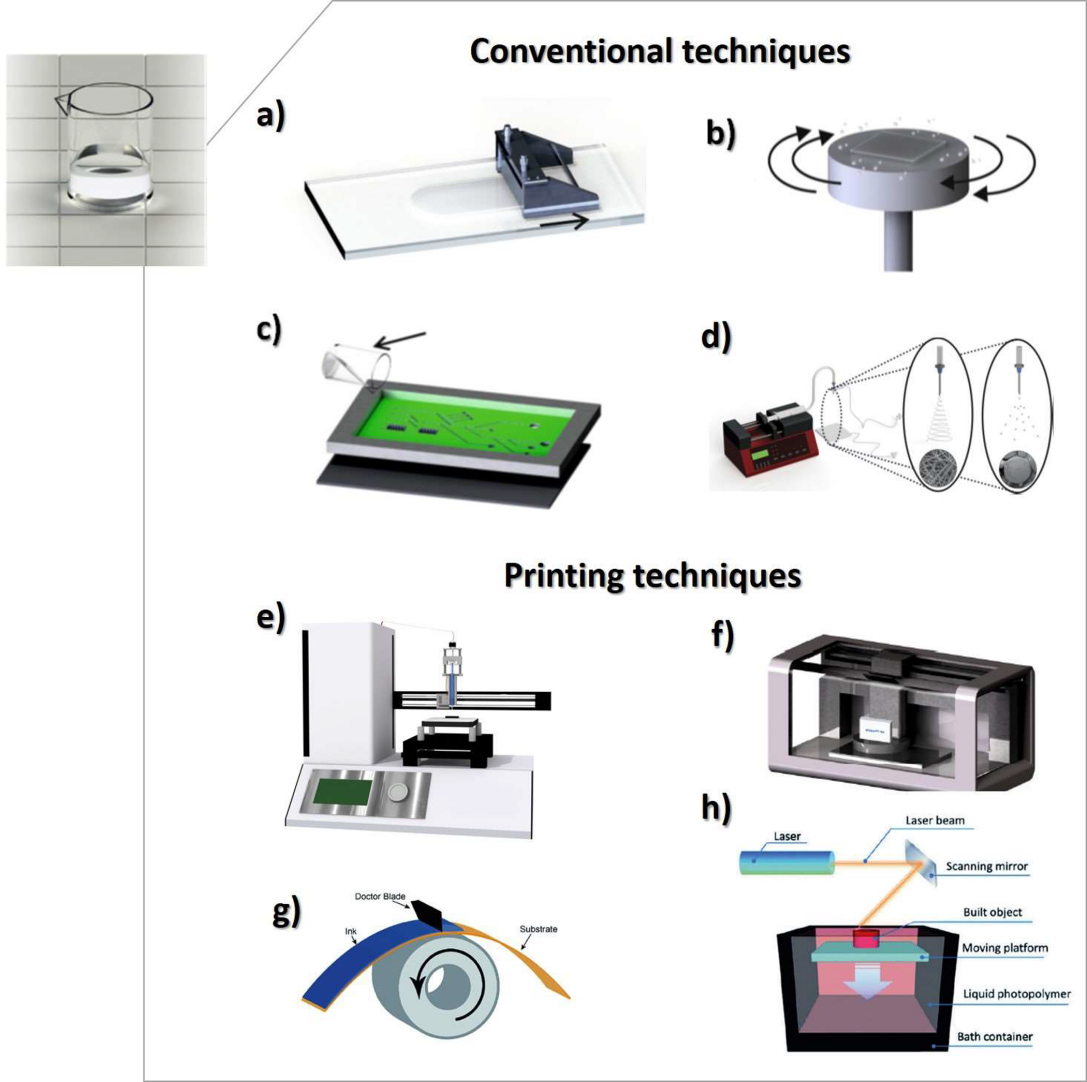
Solvent-based processing techniques for PVDF: (a) doctor blade, (b) spin-coating, (c) screen-printing, (d) electrospinning, (e) direct-ink-writing (DIW), (f) inkjet printg, (g) roll-to-roll printing/coating processes, and (h) lithography.

Together with the processing methods, the processing conditions, such as processing temperature, between 190 and 280 °C (230 to 290 °C for extrusion and 200 to 270 °C for injection molding) when processed from the melt and the solvent type and solvent evaporation temperature strongly affects polymer phase content, crystallinity, and morphology.^64^ With respect to solvent based processing, the selection of the solvent is essential because it affects the thermodynamic properties of the solution, the processing techniques that can be used, and the final physicochemical characteristics of the processed material. Figure 3 shows a schematic representation of characteristic processing techniques used to obtain PVDF in different morphologies, described below, from solvent-based solutions.

In addition to conventional processing techniques, PVDF has also been processed by additive manufacturing techniques (Figure 3) such as direct-ink-writing (DIW),^65^ roll-to-roll printing/coating processes,^66^ among others, which are of low fabrication cost.

Common solvents of PVDF are *N*,*N*-dimethyl acetaminde (DMA), *N*,*N*-dimethylformamide (DMF), *N*-methyl-2-pyrrolidone (NMP), methyl ethyl ketone (MEK), tetrahydrofuran (THF), and dimethyl sulfoxide (DMSO), among others.^67,68^ However, from an environmental point of view, these solvents do not allow sustainable processing because of their toxicity. Thus, greener solvents are necessary to reduce the environmental impact of materials and processes. Thus, one of the main challenges in PVDF processing is to change the common solvents with environmentally friendlier ones.^45^ However, the challenge of green processing is not simple, still under strong development efforts. Nonetheless, 1, 3-dioxalane (DXL) and *N*,*N*′-dimethylpropyleneurea (DMPU) are promising alternatives for being environmentally friendly (toxicity index of 5 and 4, respectively, according to Hodge and Sterner scale).^45,69^

Various established solvent-based processing techniques have been applied to process PVDF in the electroactive β-phase and new ones have emerged in the past decades. In the scope of the present review, the most used ones to obtain a specific morphologies or structures will be briefly addressed in the following.^47^

PVDF dense films with a controllable thickness, ranging from a few to hundreds of micrometers, are typically fabricated by doctor blade.^70^ The phase obtained can be tailored according to the solvent evaporation temperature, the β-phase being obtained at solvent evaporation temperatures below 70 °C. In contrast, α-phase films are obtained at higher temperatures or above the polymer’s melting temperature where rapid evaporation of the solvent occurs. A transition from α- to β-phase can be induced by mechanical stretching of the α-film at a specific temperature and electrical poling is applied to optimize the electroactive response by favoring the orientation of the dipoles along the direction of the applied field.^11^ For smaller thicknesses, from hundreds tens of nanometers to dozens of micrometers, spin-coating is applied to produce highly uniform films deposited directly on the desired substrates.^71,72^ Films and specific structures can be also achieved by using additive manufacturing technologies, such as inkjet-printing, screen-printing, and spray-printing.^73^ In this case, dense or porous morphologies can be obtained by specific postprocessing thermal treatments. Nanoscale films can be prepared using a monomolecular film assembly method: the Langmuir–Blodgett (LB) method.^74,75^ Moreover, nonsolvent-induced phase separation (NIPS)^76^ and temperature-induced phase separation (TIPS)^77^ are used to obtained porous films.

Further, techniques such as solvent casting particulate leaching and solvent casting 3D nylon template [144] are also used to obtain porous membranes with controllable pore size and interconnectivity. In turn, patterned and 3D PVDF dense and porous structures can be obtained by replica molding using molds featuring the inverse desired structure^78^ or by 3D printing.^79^ To obtain random or aligned PVDF fibers, electrospinning is the technique usually employed, as it allows to produce fibers with diameters from hundreds to dozens of nanometers by using static or rotating collectors to obtain randomly oriented or oriented fibers, respectively.^80^ In the electrospinning process, parameters such as the solvent evaporation temperature, the electric applied field and the stretching forces exercise during the process induce the crystallization of the PVDF fibers predominantly in the β-phase.^81^ Further, by controlling processing parameters such as solution viscosity, microspheres can also be obtained by electrospray, with diameters ranging from hundreds of nanometers to a few micrometers, in addition to other conventional techniques such as phase separation or precipitation, emulsion/solvent evaporation, and microfluidics.^82^ In addition to the previously mentioned procedures to obtain PVDF in the electroactive β-phase, a common strategy is the integration of fillers, such as cellulose, carbon nanotubes (CNTs), zeolites, and piezoelectric ceramics nanoparticles, into the polymer solution.^83^ These type of fillers and others featuring active properties, e.g., magnetic iron oxide (Fe_3_O_4_) and cobalt ferrites (CoFe_2_O_4_) nanoparticles, have been introduced to obtain composites with tuned physicochemical and multifunctional properties.^84^ In order to implement β-phase PVDF for technological applications by maximizing the electroactive response, a poling process is applied through static electric fields allowing to realign the dipole moments along the field direction.^85^

### 2.2 Poly(vinylidene fluoride)-Based Copolymers

**Figure 4:**
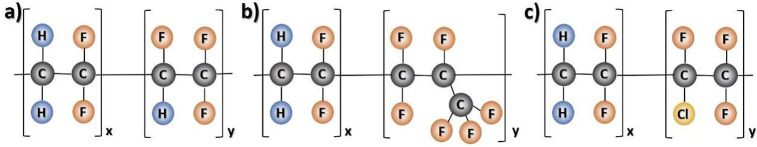
Schematic representation of the chemical structure of (a) poly(VDF-*co*-TrFE), (b) poly(VDF-*co*-HFP), and (c) poly(VDF-*co*-CTFE).

The development of PVDF copolymers such as poly(VDF-*co*-TrFE), poly(VDF-*co*-HFP), and poly(VDF-*co*-CTFE) has allowed not only to obtain the polymer directly in the electroactive phase when processed from the melt but also to improve/adapt the degree of crystallinity and the electroactive response for specific technological demands. The structure of the main PVDF copolymers is represented in Figure 4.^11^

Table 1 shows the main application fields of PVDF and its copolymers. For each application, the most used PVDF and copolymers are indicated.

**Table 1:** Main Application Areas of PVDF and Copolymers

	application area						
polymer	sensors	actuators	energy generation	energy storage	environmental monitoring and remediation	microfluids	biomedical applications
PVDF	yes	yes	yes	yes		yes	yes
poly(VDF-*co*-TrFE)	yes	yes	yes	yes	yes	yes	yes
poly(VDF-*co*-HFP)				yes	yes		
poly(VDF-*co*-CTFE)				yes			

The PVDF copolymer less used is poly(VDF-*co*-CTFE), being evaluated for applications such as Li-ion batteries, but still being little explored because it has similar properties to poly(VDF-*co*-HFP). The most used fluorinated polymers for Li-ion batteries are PVDF as polymer binder for electrodes and poly(VDF-*co*-HFP) for separator membranes and solid polymer electrolytes (SPEs).^86,87^

For applications that require the piezoelectric effect, the most used polymers are PVDF and poly(VDF-*co*-TrFE).^88^ In fact, poly(VDF-*co*-TrFE) is one of the most studied copolymers of PVDF. When the VDF content (Content_VDF_) is <49 mol%, poly(VDF-*co*-TrFE) crystallizes into 3/1-helical phase and when 49 mol% ≤ Content_VDF_ ≤ 55 mol%, poly(VDF-*co*-TrFE) crystallizes into 3/1-helical phase and trans-planar phase. Moreover, when 55 mol% < Content_VDF_ < 80 mol%, poly(VDF-*co*-TrFE) crystallizes into trans-planar phase,^13^ independently of the processing conditions, once the addition of the third fluoride atom in the TrFE monomer unit leads to a significant steric hindrance that favors the all-trans polymer chain conformation and induces the ferroelectric β-phase independently of the processing method and conditions: melt or solution casting. Due to the intrinsic presence of chemical defects (TrFE units), randomly distributed among PVDF sequences, this copolymer typically has a reduced degree of crystallinity compared to the PVDF homopolymer.^89^ However, the crystallinity can be tuned between 35 and nearly 100% depending on the polymerization method and processing conductions.^72^ Those properties ensure high electric output, sensitivity, comprehensive frequency response, and flexibility, characteristics required for applications such as haptics, sensors, and artificial muscles.^90^ Moreover, poly(VDF-*co*-TrFE) has two phase transitions, depending on the crystallization conditions, thermal treatment and molar ratio.^91^ The copolymer poly(VDF-*co*-TrFE) in the molar ratio (VDF/TrFE) (75/25) shows a first-order ferroelectric–paraelectric (FE-PE) transition at 140 °C under heating and a PE-FE transition by cooling at 75 °C, showing therefore a large thermal hysteresis.^91,92^ For this copolymer, an emerging toroidal polar topology has been observed in poly(VDF-*co*-TrFE) with VDF content of 70 mol%.^72^ This consists of the effective alignment of the lamellar crystal with its interchain dipoles perpendicular to the polymer chains self-organizing into a concentric pattern.^72^

In turn, poly(VDF-*co*-HFP), consisting of incorporating the amorphous phase of hexafluoropropylene (HFP) in the PVDF homopolymer, is a chemically inert copolymer, presenting a lower crystallinity when compared with PVDF due to the presence of the bulky CF_3_ groups. For such reasons, this polymer has been mainly used for applications in polymer electrolytes for rechargeable Li-ion batteries and for producing membranes for organophilic pervaporation. Poly(VDF-*co*-HFP) has the highest *d*_31_ piezoelectric constant (21 pC·N^–1^) among all PVDF’s copolymers.

In the case of poly(VDF-*co*-CTFE), the crystalline properties depend on the CTFE content, the semicrystalline state being only obtained for CTFE contents lower than 16 mol%, while an amorphous state is present for higher CTFE concentrations. The introduction of bulky CTFE makes the structure lose, resulting in an easier orientation of dipoles under an external electric field. Poly(VDF-*co*-CTFE) exhibits optimized piezoelectric properties (|*d*_33_| can reach values of 140 pC·N^–1^ when poled at a DC bias of 70 MV·m^–1^), higher electrostrictive strain response (5.5%), and higher ε′ of 13 when compared with PVDF. As a result, broader ferroelectric hysteresis loops are commonly observed in poly(VDF-*co*-CTFE) that are advantageous for energy storage applications, ensuring a high overall electric-energy density that can be charged/discharged in specific electronics applications.^48,93^

An interesting polymer in the PVDF family is poly(vinylidene fluoride-*ter*-trifluoroethylene-*ter*-chlorofluoroethylene) (poly(VDF-*ter*-TrFE-*ter*-CFE)) which is a *ter*-polymer with relaxor ferroelectric properties and high dielectric constant.^94^ This polymer has been used in electrocaloric devices with entropy changes of 37.5 J ·kg^–1^·K^–1^,^95^ actuators,^96^ bioMEMs, and microfluidic devices,^97^ among others. The addition of the third monomer affects the crystalline properties and consequently the dielectric properties.^25^ This terpolymer under 40 MV·m^–1^ shows an electromechanical coupling factor (*k*_33_) of 88% and a *d*_33_ > 1000 pC·V^–1^.^98^ Considering its properties, this *ter*-polymer has been also used as a polymer binder for electrodes in Li-ion batteries devices.^99^ In the future, it is expected to find application in other high-tech areas.

### 2.3 Poly(vinylidene fluoride)-Based Composites

The exciting properties of PVDF have led to a high and growing interest, resulting in the combination with specific fillers for the development of high-performance and multifunctional PVDF-based composites with distinct morphologies and physicochemical properties. Thus, PVDF-based composites result from the combination of one or two different fillers with complementary characteristics to improve some specific properties or to induce new ones, such as electrical conductivity or magnetic properties. It has been reported that there are PVDF composites with more than 30 different fillers. Recently, the trend has been the development of PVDF composites with the addition of more than one filler with complementary properties to reduce the filler amount and enhance the functionality.^100^ The most representative and commonly used fillers in PVDF and its copolymers-based composites are magnetic nanoparticles (CoFe_2_O_4_ or Fe_3_O_4_),^101−103^ CNTs,^104−106^ ceramic particles, e.g., barium titanate (BaTiO_3_),^107,108^ zinc oxide (ZnO),^109−111^ titanium dioxide (TiO_2_),^112−114^ zeolites, or clays,^115,116^ among others, promoting the development of a wide range of multifunctional composites materials.

PVDF composites are interesting because their final properties can be fine-tuned by the proper selection of filler size, shape and content, dispersion, interface, and interaction between filler and polymer, together with the processing conditions.^117,118^ Moreover, the easy processability of PVDF composites allows its integration in various application areas, including sensors and actuators, energy generation and storage systems, environmental sensing and remediation, and biomedical applications, among others, as it will be shown in the following chapters in this review.

### 2.4 Poly(vinylidene fluoride)-Based Blends

PVDF and copolymers are also widely used in blends with different types of polymers, including poly(methyl methacrylate) (PMMA),^119^ poly(*o*-methoxyaniline) (POMA),^120^ poly(aniline) (PANI),^121^ PLLA,^122^ poly(ethylene terephthalate) (PET),^123^ poly(vinyl chloride) (PVC),^124^ poly(ethylene oxide) (PEO),^125^ poly(vinyl alcohol) (PVA),^126^ poly(amide 11) (PA11),^127^ and poly(carbonate) (PC),^128^ among others, as well as with different ionic liquids (ILs).^129^ Those polymer blends allow to improve processability, nucleate specific crystalline phases, or tune optical and electrical properties, among others.

PVDF/PMMA is the most used blend in which the dynamic heterogeneity^130^ promotes the formation of the β-phase.^131^ Further, the piezoelectric effect increases with the addition of PMMA to PVDF.^132^ This blend has been applied in optical applications,^92^ separator membranes for Li-ion batteries,^133^ controllable wettability switching triggered by external electric field,^134^ pyroelectric application,^135^ as well as a coating for the conservation of historic structures exposed to atmospheric agents,^136^ among several other applications.

Another interesting blend is PLLA/PVDF, composed of two piezoelectric polymers and suitable for energy harvesting devices.^122^

To enhance electrical properties, various blends of PVDF with conductive polymers (PANI),^137^ polypyrrole (PPy),^138^ and poly(3,4polyethylenedioxythiophene-polystyrenesulfonate) (PEDOT:PSS)^139^ have been produced for electrodes in sensors/actuators and biomedical applications.

Also, to improve dielectric strength and flexibility, blends of PVDF with poly(VDF-*ter*-TrFE-*ter*-CFE) have been developed, being suitable for dielectrics and energy storage applications, as the dielectric breakdown strength of this blend is improved due to increased modulus of elasticity and reduced mobility of the polymer chains.^94^

More recently, PVDF blends with different ILs are being developed taking into account the tunability of ILs, leading to diverse applications from biomedicine to energy storage.^129,140^

### 2.5 Sustainability and Circular Economy Considerations

Considering the excellent properties and applicability of PVDF-based materials, leading to an increasing demand, sustainability, and end-of-life considerations are essential.

In terms of durability, PVDF polymer is highly durable as it can be reused and processed for up to five times, which is very important in advanced applications and in the scope of sustainability.^141^

A life cycle assessment (LCA) has been carried out to assess the environmental impact of PVDF, in which an acceptable value for the global warming potential (GWP) has been obtained (55.8 kg CO_2_ equiv·kg^–1^ PVDF) due to the large demand for chlorine during its production.^142^

With regard to processing and to increase sustainability, it has been demonstrated the suitability of replacing the conventional solvents with green solvents, including cyclic carbonate solvents^143^ and acetyl tributyl citrate (ATBC),^144^ among others.

To further reduce environmental impact, it is necessary to define more efficient processing and recycling routes, as this polymer starts to degrade at relatively low temperatures (>400 °C). Thermal degradation process such as thermolysis is used to reuse and recycle PVDF.^141^ Also, the decomposition of PVDF and its copolymers can be obtained by mineralization processes in subcritical water with the addition of KMnO_4_ as oxidizing agent.^145^

One of the possible ways is mechanical recycling through remelting through extrusion. In the United States of America (USA) and Europe, several companies are specialized in recycling PVDF.^146^ Furthermore, considering that PVDF is a partially fluorinated polymer, more assessments of the toxicity and low carbon economy are important in the future.

In summary, considering its demand, two major producers (Solvay and Arkema) will expand its production capacity in the next year,^147^ being therefore urgent to reduce environmental impact all along the value chain of PVDF by implementing green chemistry, safe and recyclable by design concepts.^148^

## 3 Applications

Initially, PVDF was used in wires, cables, and tubes, among others, based on its excellent mechanical properties, high thermal stability, and processability, but considering also its electroactive properties, it has been applied in high added-value applications such as sensors, actuators, energy harvesting, and storage systems, environmental, microfluidics, and biomedical applications, which is the focus of this review. Thus, in this review, the state-of-art and corresponding discussion are divided by application type.

**Figure 5:**
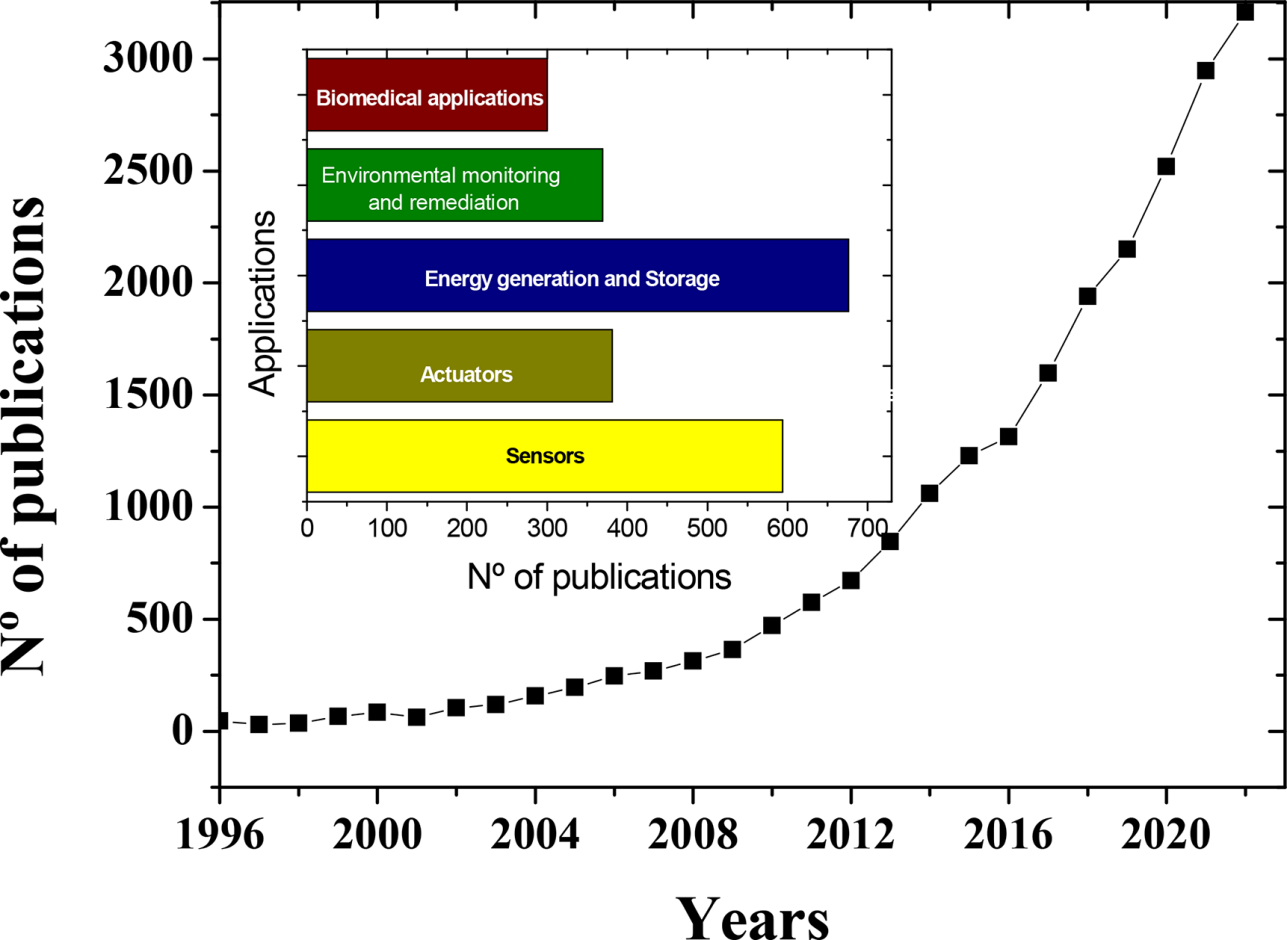
Scientific articles published over the years until 2022, considering PVDF and its copolymers, also indicated by application area. Search performed in Scopus database: accessed on July 7, 2023, with the keywords “PVDF”, “PVDF co-polymers,” and their corresponding applications.

Considering its wide range of applications, including sensors, actuators, energy harvesting, and storage, environmental monitoring and remediation and biomedicine (including microfluidics, portable analytical devices (point-of-care, POC)), Figure 5 shows the growing number of scientific articles published in recent years by focusing on PVDF and copolymers. The publications by application area are also stated.

Figure 5 shows the strong growth of works related to PVDF for various applications in recent years, focusing on the development and understanding of multifunctional composites, the development of advanced processing conditions and procedures, and the demonstration of their applicability in different areas.

**Figure 6:**
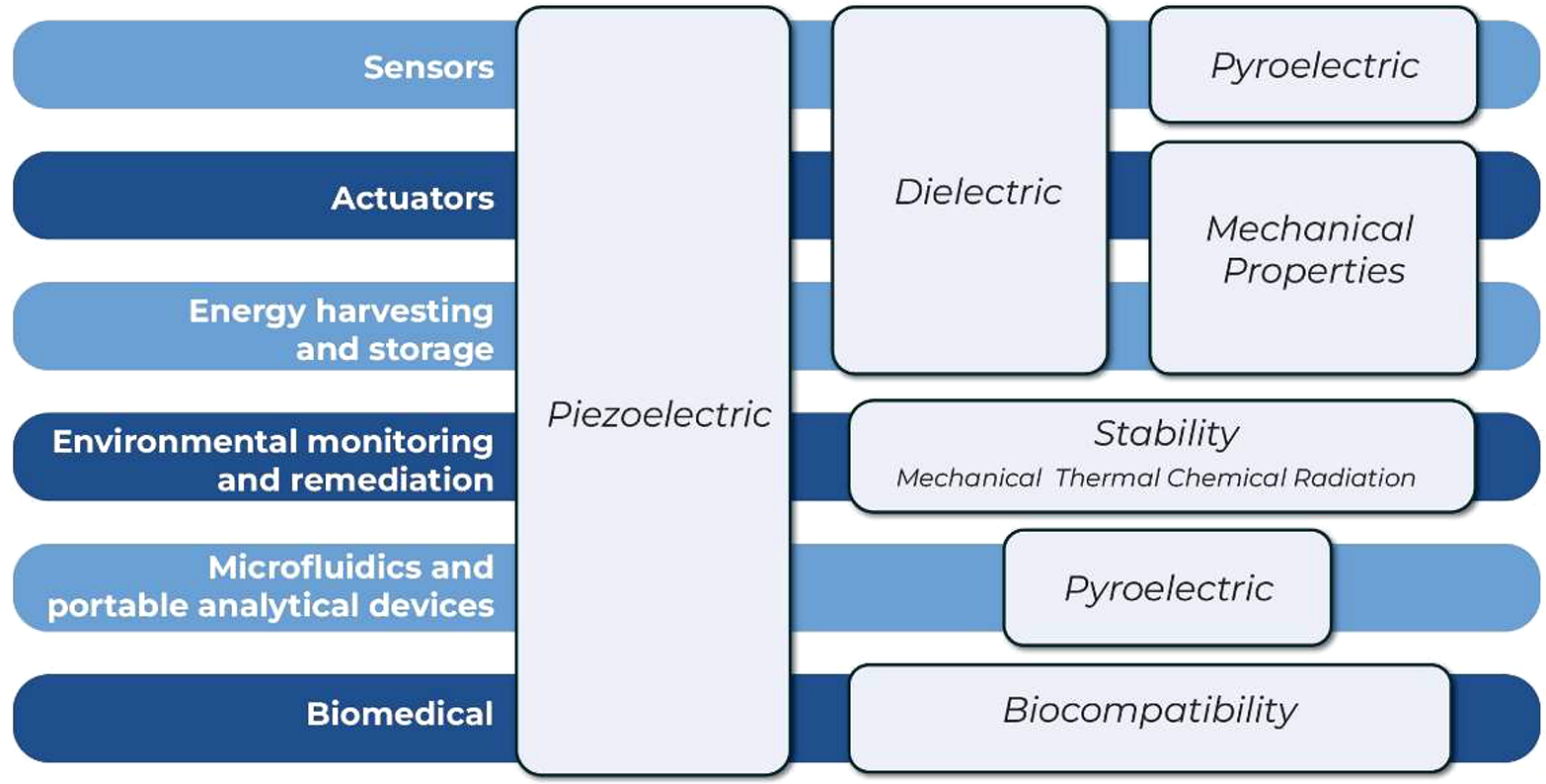
Summary of the main application areas of PVDF-based materials, together with the main properties that may play a relevant role in those applications.

In the following, the state of the art on the different application areas is presented, composed by a brief introduction, principal challenges, respective trends, and final remarks. A particular focus relies on the main functional, electroactive, and physicochemical properties of the developed materials, which as summarized in Figure 6, are also emphasized according to the application requirements.

The main property common to most applications is the piezoelectric effect, which is one of the main differential characteristics of PVDF-based polymers with respect to other polymers.

**Figure 7:**
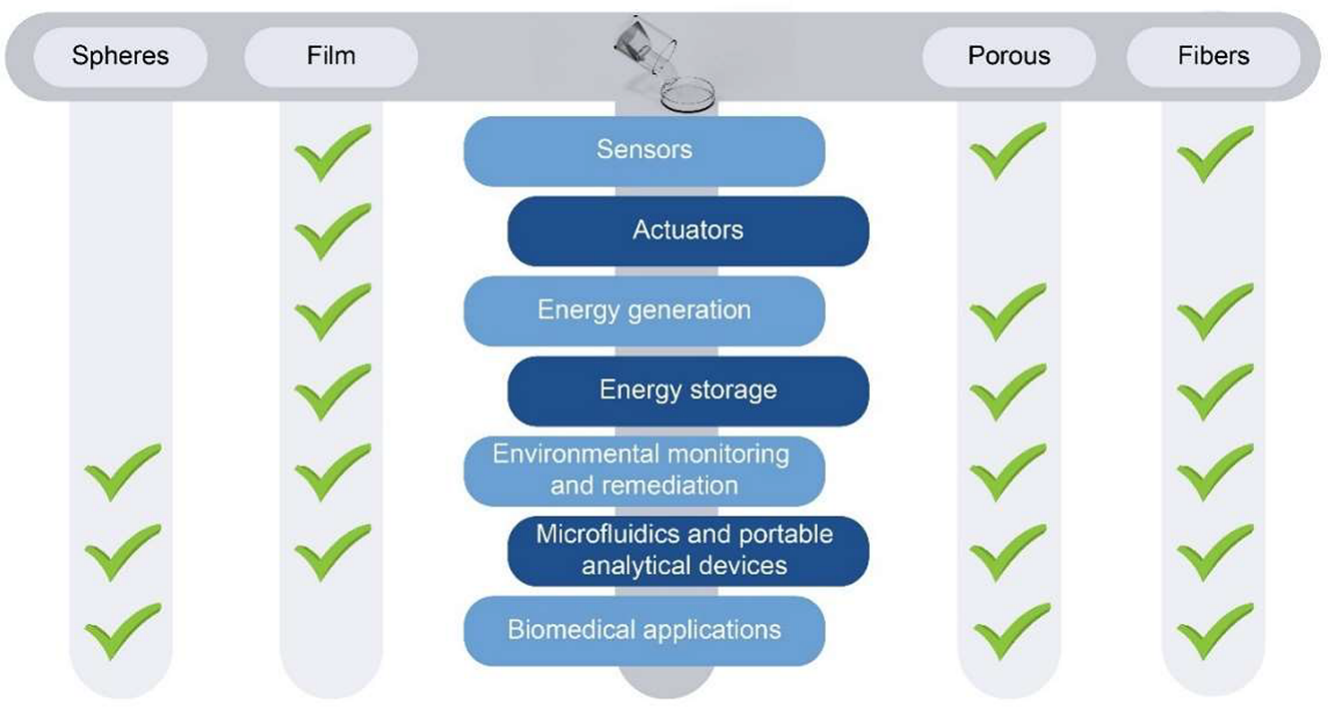
Main morphology types of PVDF-based materials for different application areas.

Furthermore, for many applications, the morphology of the samples plays a critical role, Figure 7 showing the main morphologies of PVDF-based materials required for the different applications.

Morphologies can be obtain through the different techniques previously discussed (section 2.1). Considering the different materials functional characteristics and morphologies, the state-of-art for each application area will be presented in the following.

### 3.1 Sensors

A sensor is a device that detects different stimuli and provides a specific response. Different types of sensors have been developed based on PVDF considering the specific stimulus, transduction mechanism, and materials morphology.

**Figure 8:**
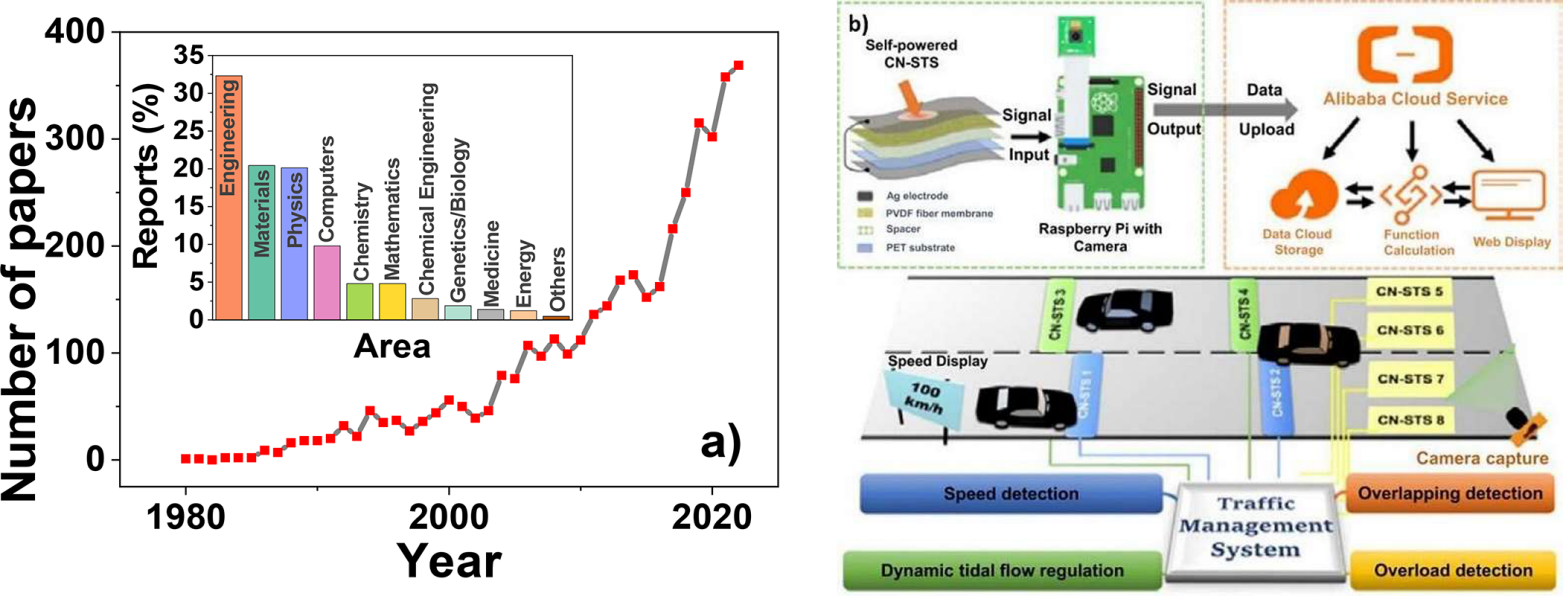
(a) Number of papers (reported on SCOPUS database: accessed on February 1, 2023) regarding the PVDF+sensor topic until 2022. Inset: Distribution of papers (reported on SCOPUS database: accessed on February 1, 2023) by area regarding the PVDF+sensor topic until 2023. (b) Very recent (2022) example of using a PVDF-based sensor on a smart traffic monitoring and management system with IoT connectivity. Reproduced with permission from ref (149). Copyright 2022 Elsevier.

Over the past decades, PVDF-based sensors have experience a growing interest, as shown by the high number of published articles related to this subject (∼400) and the number of areas in which these materials had substantial impact ranging from engineering to medicine (Figure 8a).

Forty years separate the theoretical work of Lang et al.^150^ that introduced the idea of using PVDF stress sensors for biomedical piezoelectric palpation devices, and the very recent triboelectric sensor array for the IoT-based smart traffic monitoring and management system reported by Yan et al.^149^ that allows speed, overlapping, and overload detection as well as dynamic tidal flow regulation, under the framework of smart traffic control (Figure 8b). In this historical leap, the focus has shifted from the most fundamental studies (although there is still need for them) to the exploration of technological niches, to develop new technologies that respond to the requirements of modern cities and smart societies. The dramatic growth and densification in modern cities require smart solutions to address critical demands such as mobility, healthcare, energy, and civil infrastructure. In this context, the digitalization of the society and the economy, enabled by the IoT concept, is one of the most promising enabling technologies for tackling these challenges by creating interconnected physical objects, sensors, and networks.^151^ To ensure effective communication between all those objects/materials, smart materials, particularly piezoelectric, and pyroelectric ones, are needed; once these smart materials allow the development of sensors that can measure properties such as vibration, strain, temperature, and heat.^152^

Even knowing that PVDF’s both piezoelectric and pyroelectric coefficients (including those found in its copolymers) are lower than those reported for ferroelectric ceramics, PVDF, its copolymers, and PVDF-based composites display relevant advantages for sensing applications such as softness and flexibility, lightweight, low electrical permittivity, small thermal conductivity, printability, and excellent impedance matching to both air and water.^11^

For the above-mentioned characteristics, PVDF’s piezoelectric effect has been extensively used in technological applications that involve the detection of mechanical stimuli such as force, pressure, and strain (both compressive and tensile), tactile awareness, vibrational, acceleration, and acoustic.^11^ This widespread applicability is also intimately related to the PVDF’s broad frequency bandwidth, high sensitivity, robustness, easy processing, high environmental and chemical stability, and reliability. Also relevant is that PVDF-based sensing devices can be self-powered, only requiring a simple ground connection to one of their electrodes.^153,154^ This particular attribute is a pivotal milestone for sustainability, processability, and integrability in mobile, wearable and hard-to-access devices and objects, over competing sensing technologies.^153,155^ Additionally, the addition of fillers into the PVDF matrix, besides being able to increase the dielectric, pyroelectric, and piezoelectric responses of the polymeric matrix, also allows introduction of new functionalities/capabilities such as magnetic, ionic, or conductive, opening new application highways for other technological devices, such as magnetic sensors, moisture sensors or piezoresistive sensors.^11,156^

Important figures of merit (FOM) for PVDF-based sensors are the sensitivity (*s*) and electromechanical coupling coefficient (*K*), the sensitivity being highly dependent on the piezoelectric voltage coefficient (*g*). In practice, if the generated voltage signal is small, it has to be enhanced by an electronic amplifier.^157^ The electromechanical coupling coefficient *k* is usually used to describe such conversion efficiency between electrical and mechanical energy, according to eqs 1 and 2,^157^ where *d* refers to piezoelectric coefficient, ε′ refers to dielectric constant, ε_0_ refers to the permittivity of free space, and *s* refers to the compliance.
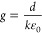
1
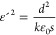
2

From the experimentally pioneer PVDF insole multisensor for pedobarography (study of the pressure distribution under the foot in standing and walking animals) developed by Pedotti et al.,^158^ key innovations in the PVDF-sensing area are now related to the optimization of new structures or processing technologies for better performances (increasing electrical performance while maintaining flexibility), or new application scenarios that have rarely employed PVDF-based materials,^157^ such as piezotronics, spintronics, sensors based on luminescence (photo or mechano), steady-state sensors, and supersensitive cellular sensing devices.^159^

This section highlights the use of PVDF in sensing technologies since the 1980s. It will focus on examples showcasing the use of PVDF-based materials on temperature, pH, gas, stress, biosensors/human health monitoring, and environmental sensing. In each of the examples, an emphasis will be given on detailing the polymer’s key feature to achieve the desired application and the reported FOM (sensitivity, selectivity, limit of detection, reproducibility, and stability) for the described sensing platforms.

#### 3.1.1 Capacitive Sensors

In the 1960s, capacitive sensors started to be used to measure strains at high temperatures with good long-term stability.^160,161^ Capacitive sensors have some advantages such as low-power consumption (capacitive sensors do not consume DC currents), and the sensor output can be directly designed to digital output using energy-efficient capacitance-to-digital converter circuitry, avoiding nonidealities of voltage buffers and signal conditioning integrated circuits.^162^

More recently, flexible pressure sensors have attracted much attention due to their ability to measure the local contact pressure and its spatial distribution with exceptional stability and repeatability. Such features are beneficial for a wide range of applications including prosthetics, health monitoring, or human–machine interaction.^163^ In this context, Luo et al. proposed a micropillar–PVDF device with high sensitivity (0.43 kPa^–1^) in the low-pressure regime (<1 kPa) and with a Δ*C*/*C* = 0.6 under a bending angle of 90°, suitable for capacitive pressure sensors on wearable devices and human-machine interactive tools.

PVDF has also been used in wearable applications for capacitive sensing in the form of fibers.^164^ The reported change in capacity in the PVDF sample reaches maximum values of Δ*C*/*C* = 0.38 for a pressure of 69.35 kPa. The introduction of TiO_2_ nanoparticles into the PVDF fiber composition increased the Δ*C*/*C* to the value of 0.47, which is appropriate for insole sensors in sports shoes.^165^ Still in composites, a (poly(VDF-*co*-HFP))/1-ethyl-3-methylimidazolium bis(trifluoromethylsulfonyl)imide ([EMIM][TFSI]) IL composite film (with a 6.5/3.5 weight ratio) has been proposed for textile-based capacitive pressure sensing.^164^ The proposed device exhibited a large value of Δ*C*/*C* (∼200) and a sensitivity of 9.51 kPa^–1^ for a pressure of 100 kPa. Additionally, a 3 × 3 pressure sensor array able to detect not only position but also weight and object types was discussed. In a different strategy and aiming the successful detection of harmful gases under the umbrella of wearable electronics, a bendable capacitive sensing device composed of UiO-66-NH_2_ and electrospun PVDF was also proposed. The Δ*C*/*C* = 0.01 allowed to detect small concentrations of sulfur dioxide (SO_2_) (150 ppm),^166^ opening a new exciting application area in this type of polymer-based composites.

#### 3.1.2 Pyroelectric Sensors

**Figure 9:**
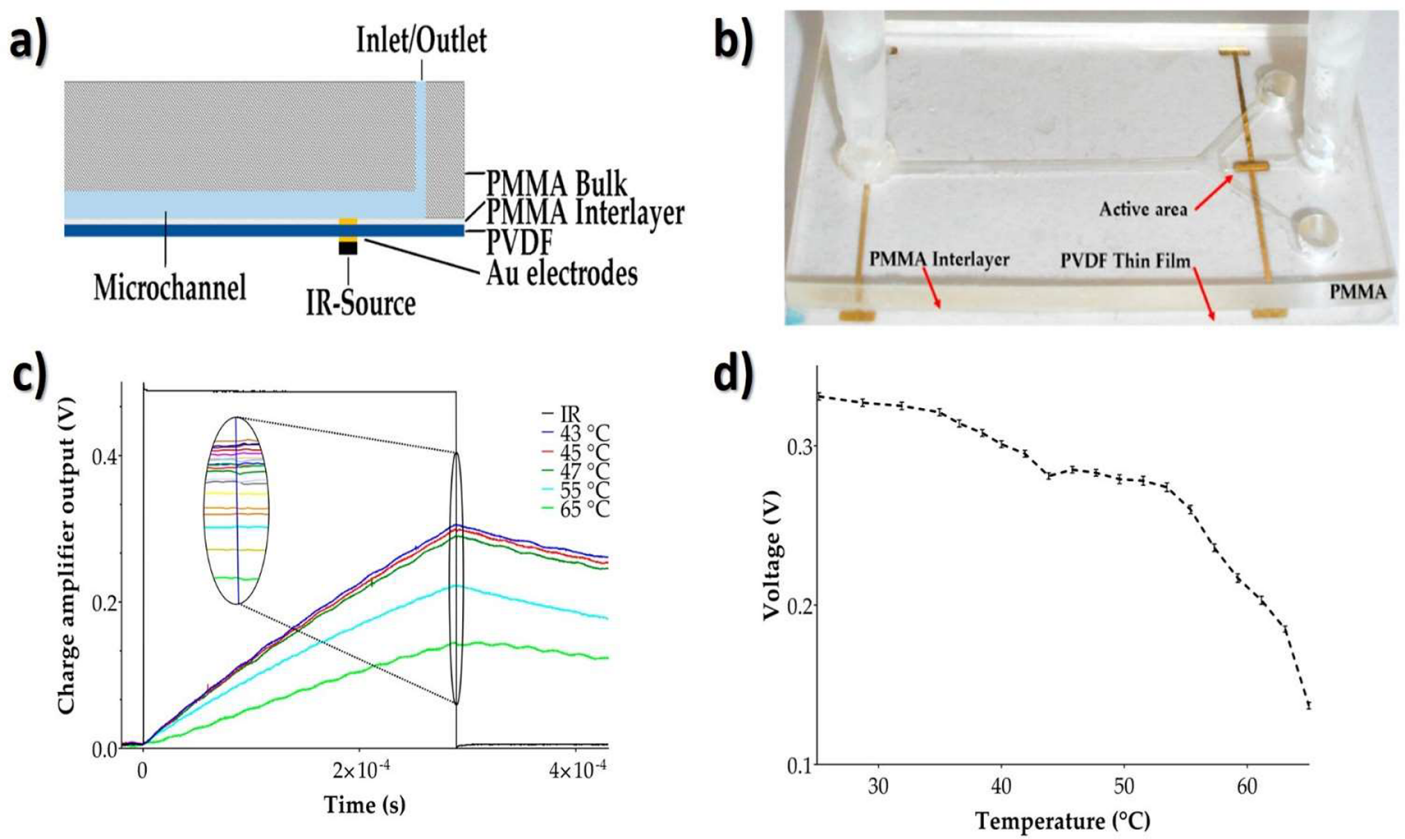
(a) Cross-section representation and (b) photograph of the PVDF-based microfluidic device on which the gold (Au) contacts were deposited to act as both active and electrical components to the read-out circuit. (c) Experimental results (charge amplifier voltage output vs time) were obtained at five different target temperatures (from 43 to 65 °C). The black line exhibits the data obtained for infrared radiation-light-emitting diode (IR-LED) stimulation. (d) Calibration of the proposed pyroelectric sensing device inside the microfluidic channel. Reproduced with permission from ref (168). Copyright 2017 Multidisciplinary Digital Publishing Institute.

Once PVDF has piezoelectric properties and a robust pyroelectric response (dipolar variations induced by heat variations^167^), it can be used for pyroelectric sensing as demonstrated by S. Pullano et al.,^168^ which presented a ferroelectric polymer-based temperature sensor designed and optimized for microfluidic devices (area that will be discussed in more detail in section 3.5) (Figure 9).

The reported performance of the PVDF pyroelectric sensor (∼0.3 V voltage variation for a temperature range between 25 and 65 °C) took advantage of the high compatibility between PVDF and PMMA in the fabrication of disposable devices. Such performance can be even further improved with the inclusion of electronic microsystems such as low-noise complementary metal-oxide semiconductor (CMOS) charge amplifiers and filtering tools on silicon boards. In a similar approach, the successful integration of a ∼28 μm thick film of PVDF into a system-on-a-chip board was achieved, facilitating the fast monitoring of temperature in specific sites of the biological fluid and avoiding errors in the assessment of thermal evolution of the fluid during the study.^168^ Such PVDF-based pyroelectric sensor can detect the absolute temperature instead of the temperature gradient, with a ∼0.005 s response time in a temperature range of −40 to 65 °C and with a sensitivity of 5 mV·°C^–1^.

From a theoretical point of view, Jia et al.^169^ conceived a PVDF-based pyroelectric sensor at the circuit level, being the theoretical model optimized with the introduction of experimental details in the environment of Multisim so that the simulated data could be accurately compared with real data. The theoretical sensitivity of the device was found to be 0.1063 V·°C^–1^, very similar to the experimentally measured one (0.1068 V·°C^–1^). Additionally, it was stated that the key features to achieve consistent simulated results were the: (i) intensity of pulse current and (ii) width of the current pulse.

Aiming to mimic some of the functionalities of human skin, Lee et al.^170^ demonstrated that an ∼80 μm thick polymer-based film (composed of PVDF, ZnO nanostructures, and graphene (Gr) electrodes) was capable of simultaneously measuring pressure (∼10 Pa resolution by sensing the electrical resistance variation through the piezoresistance of the material) and temperature (in the 20–120 °C range with a sensitivity of ∼13 mΩ·°C^–1^). Such a technological platform exhibited a pressure detection limit 3 orders of magnitude higher than the required for artificial skin with a sensitivity of ∼11 mΩ.Pa^–1^, opening promising application potential in the area of biorobotics.

Still with respect to the e-skin concept, a similar PVDF film with a thickness of ∼110 μm, a piezoelectric response of 106 pC·(Vm)^−1^, an area of 10 cm^2^, a pyroelectric coefficient of 300 C·(m^2^ °C)^−1^, and an output voltage of about 3 mV has been reported by Yuji et al.^171^ to monitor temperature variations of the human skin. The sensitivity was found to be 2 mV·°C^–1^ in the 10–50 °C temperature range.

A novel, lightweight, low-cost, and high flexible triboelectric nanogenerator composed of PVDF (6 cm × 3 cm × 1 mm), polytetrafluoroethylene (PTFE), and copper foil as the electrode was used by Zhu et al.^172^ as a self-powered temperature sensor, exhibiting a detection range of 10–90 °C, 0.01 s response time, and a 3.5 s reset time. Humidity was found to be an essential factor in the output voltage of the sensor: at 20 °C, the output voltages were 42, 37, and 32 V for a relative humidity of 70, 80, and 90%, respectively, being the sensitivity maximized for the measurement at 70% relative humidity conditions (2.1 mV.°C^–1^), showing a promising application potential in the environmental, safety, and biomedical fields.

By adding BaTiO_3_ into a poly(VDF-*co*-TrFE) matrix, Gupta et al.^173^ was able to monitor temperatures in the 26–70 °C range with an almost linear response and a sensitivity of 15.34 mV·°C^–1^, opening new and exciting directions for temperature tactile sensing in robotic applications.

In the same “composite strategy,” Hernández-Rivera et al.^174^ produced a piezoelectric PVDF/Gr membrane through electrospinning, for respiratory rate and temperature sensing with a maximum sensitivity of ∼0.34 pC·°C^–1^. Such sensors can be applied to other areas such as moisture, light, and pressure sensing due to their optimized electroactive properties.

Knowing that temperature sensing ability is essential for successful robot perception (allowing biomimetic information acquisition), Sun et al.^175^ developed a soft robotic manipulator capable of temperature sensing. The structure of the PVDF-based sensor composed of a poled PVDF film, silver (Ag) electrodes on both surfaces of PVDF, and a PET thin film packing allowed a sensitivity of 0.478 V·°C^–1^. When coupled to the triboelectric nanogenerator tactile sensory data, such temperature data obtained from the PVDF-based sensor can allow shape recognition of objects and devices, offering great potential for IoT-related human-machine interfaces.

Changing the focus to near-infrared (NIR) sensing/manipulation, Liu et al.^176^ proposed a flexible battery-less implantable device composed of Gr and PVDF. Such a device was found to exhibit a good relationship between its response (temperature, temperature-change rate, output open-circuit, voltage, and short-circuit current) and the NIR irradiation stimulus. The maximum voltage and current were 2 V and 200 nA, respectively. Additionally, the harvested energy was able to light up 4 commercial LEDs, stimulate a live rat’s heart and actuate a frog’s nerve, opening possible applications in the bioelectronics field.

Because PVDF cannot be directly used to detect light because of its weak absorption in the visible and NIR zone, the incorporation of nanostructures such as gold (Au) nanocages has been proposed to develop PVDF-based composites able to convert light into heat and electricity.^177^ A voltage output of 7.2 V, a current of 28.1 nA, and a temperature variation of 50 °C were detected when the PVDF/Au composite was subjected to NIR cycles (808 nm diode laser with a power density of 0.2 W·cm^–2^). More recently, electrospun PVDF/cesium tungsten oxide composites were evaluated for NIR-triggered pyroelectric sensing and harvesting.^178^ For the sample with 7 wt % (weight percentage) of cesium tungsten oxide it was determined that an output of 4.36 V and 214 nA (for a NIR radiation with 2.26 kW·m^–2^) was high enough to power liquid crystal display (LCD) timers and 4 LEDs.

#### 3.1.3 Piezoelectric Sensors

Due to the wide frequency response range, wide pressure range (up to 20 GPa), high sensitivity, good mechanical properties, and easy processing, PVDF is the most used material for polymer-based stress, strain, impact, and vibration sensors. This reputation came in the 80s of the last century when Domenici et al.^179^ produced a shear stress detection sensor composed of an elastic layer of PVDF sandwiched between two rubber layers and fixed to a rigid substrate. The experimentally obtained sensitivity (6 nC·N^–1^·m^–1^) was double than the one predicted analytically (3 nC·N^–1^·m^–1^), a substantial difference in the sensor output attributed to factors such as the presence of friction between PVDF and rubber layers. Two decades later, Kärki et al.^180^ developed a stress sensing device prototype composed of commercial PVDF with four separated sensor components. The reported sensitivities were found to be 12.6 mV·N^–1^ for the normal force, 223.9 mV·N^–1^ for the anterior–posterior shear force, and 55.2 mV·N^–1^ for the medial–lateral shear force. Despite such promising results, the proposed sensor is only an early prototype; further developments such as the development of a matrix sensor, electronic components, array-type solutions, and long-term evaluation are still needed.

**Figure 10:**
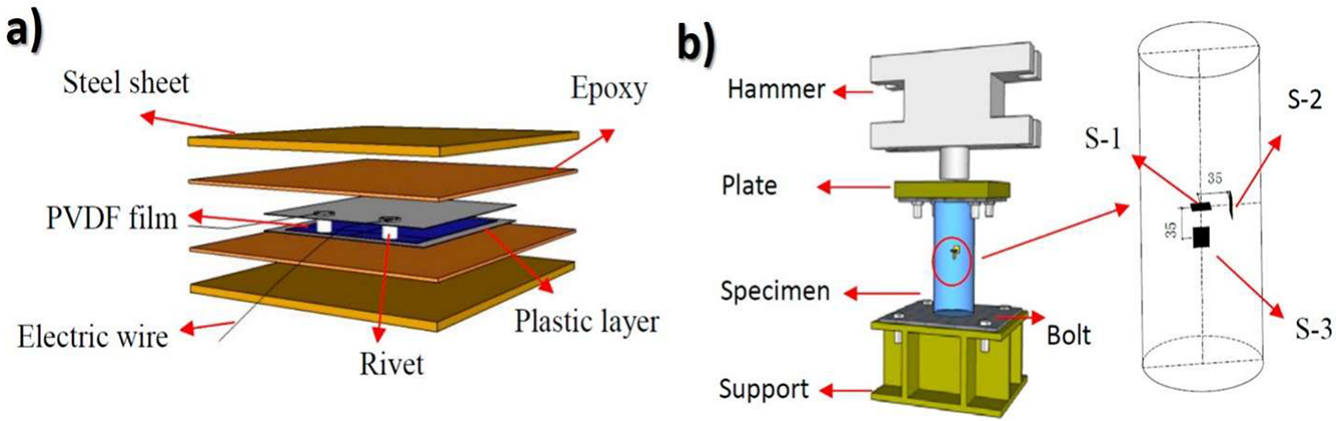
(a) Schematic representation of (a) the PVDF smart sensor, and (b) the location of the PVDF smart sensors (S-1, S-2, and S-3). Reproduced with permission from ref (181). Copyright 2018 Multidisciplinary Digital Publishing Institute.

Following a different strategy, a PVDF (22 mm × 18 mm × 0.3 mm) piezoelectric smart sensor (PVDF as a sensing element that is insulated and protected by PET layers to ensure good toughness, tensile strength, and impact resistance) was used to monitor impact (Figure 10). For that, the PVDF-based sensor was placed into a multilayer structure and successfully applied to study the internal stress of the concrete core of a concrete-filled steel tubular column under impact loads with a sensitivity of ∼1.32 MPa·V^–1^.^181^

The authors stated that similar PVDF-based impact sensors could be embedded in the concrete-filled steel tubular structures in a distributed way to evaluate the internal stress distribution when the material structure is exposed to impact loads.

With the aim to measure the interfacial stress of a composite aluminum (Al) beam with a deposited ice layer, Akitegetse et al.^182^ embedded ∼25 μm thick PVDF strips, reporting good repeatability in the measurements (standard deviation of 4–8%), providing a new and creative tool for the accurate measure of ice’s adhesion strength on different substrates. The proposed PVDF-based sensing device directly measured (through the piezoelectric effect) the mechanical stresses (0.5 MPa for an ice thickness of 2.5 mm, 0.2 MPa for an ice thickness of 4 mm, and 0.05 MPa for an ice thickness of 10 mm) induced by mechanical stimulus at the substrate/ice interface.

Four years later, Cai et al.^183^ designed a PVDF (∼30 μm × 30 mm^2^× 30 mm^2^) stress sensor with fast response (ns), high sensibility (49.2 GN·C^–1^), and wide working range (0–5 cm) suitable for antiexplosion elements.

Still, in an explosive/ballistic perspective, Ma et al.^184^ designed a temperature compensation of a PVDF stress sensor to be used to evaluate gun propellant charge compression stress with a sensitivity of ∼53 pC·N^–1^. Additionally, the maximum relative error after temperature compensation in the −40 to 30 °C range was found to be less than 0.0134%, revealing that PVDF stress sensors can be accurately used on the compression stress test of a gun’s propellant charge.

More recently, and linking biomedicine with robotics, Li et al.^185^ developed a built-in sensor system for internal shear strain and stress distribution measurement by embedding PVDF within the artificial skin of soft robotics. The determined 50 pC·mm^–1^ sensitivity is suitable for application in soft material strain/stress measurements and in soft robotics development. Electrospun PVDF was also used for vibration measurement in a string.^152^ The proposed sensor (composed of 218 ± 53 nm fibers) exhibited a linear relationship (0.2 mV·με^–1^) between output voltage (0–600 mV) and strain (0–2300 με).

#### 3.1.4 pH and Gas Sensors

The sensing of pH value is relevant in many areas, such as chemical engineering, environmental industry, and the biomedical field.^186^ Due to the combined effect of the variation in surface chemistry and the roughness of the PVDF structure, linked to its high mechanical strength, chemical resistance, and thermal stability, this ferroelectric polymer is often used in pH sensing applications.^187^

In this context, a self-powered pH sensor based on a PVDF/ZnO hybrid composite nanogenerator was produced through a solution-casting technique by Saravanakumar et al.^188^ The proposed PVDF/ZnO hybrid composite revealed a maximum open-circuit voltage of 6.9 V and a short-circuit current of 0.96 μA, with an output power of 6.624 μW under uniaxial compression that powered the sensor with a sensitivity of 0.06 V per pH unit (pH^–1^).

A pH-sensitive porous membrane composed of PVDF and acrylic acid was successfully synthesized through molecular graft copolymerization of acrylic acid with ozone-preactivated PVDF backbone.^189^ The flux of the aqueous solution through the PVDF-based sensor induced a strong and reversible dependence on pH solution (the solution flux through the membrane decreased from ∼5 mL·cm^–2^·min^–1^ to ∼2 mL·cm^2–^·min^–1^ with increasing pH from 1 to 6), a fact that proved the pH-sensing capability of the device. Flow measurements and caffeine release experiments performed with a similar membrane composition (PVDF/acrylic acid) showed that the porous structures exhibited a pH-dependent behavior.^190^ The solution flux through the membrane decreased from ∼4 mL·cm^–2^·min^–1^ to ∼1 mL·cm^–2^·min^–1^ with increasing pH from 1 to 6.5. Other preliminary results reported in the same work, related to release experiments with caffeine as a model drug, suggested that it is possible to use similar pH-sensitive-PVDF membranes to induce pH-sensitive dissolution of drugs. Ju el al.^191^ presented the development of antifouling PVDF/poly(methyl methacrylate-2-hydroxyethyl methacrylate-acrylic acid) microfiltration composite membranes for pH-sensing applications. Such membranes were found to have excellent pH sensitivity (the solution flux through the membrane decreased from ∼1 mL·cm^–2^·min^–1^ to ∼0.7 mL·cm^2–^·min^–1^ with increasing pH from 2 to 11), pH reversibility response, and good protein antifouling properties.

More recently, Pastore et al.^192^ improved the pH determination of a PVDF-based colorimetric sensor by combining two acid–base indicators (tetrabromophenol blue and phenol Red), the resulting hue being detected with a charge-coupled device (CCD) camera. The reported inflection prediction error of the sensor was in the range of 0.01–0.16 pH unit, and the sensitivity (ΔH/ΔpH) of 1.01 is appropriate for pH colorimetric sensors.

Changing the focus to the biomedical area and knowing that detecting the pH value at the wound sites could monitor and support the wound healing process, Zhao et al. fabricated a PANI-modified PVDF yarn for pH sensing,^186^ being reported an 8.53 mV·pH^–1^ sensitivity in the pH range from 4.0 to 8.0. Such performance proved that the electrospun PANI-PVDF yarn has high potential application in smart surgery dressings.

PVDF has also been used in gas sensing devices due to its flexibility, mechanical strength, thermal stability, tailorable porosity, and superior adsorption and desorption characteristics.^193^ In this line, a PVDF/palladium (Pd) all-optical laser-intensity-amplitude-modulated hydrogen (H_2_) sensor has been produced by Mandelis et al.^194^ The detection range of this durable and robust sensor (0.2 −100% of hydrogen volume concentration) is suitable for sensitive monitoring of the explosive range (4% by volume of H_2_ in the air), the sensor’s output reflectivity varying from ∼0.273 to ∼0.250 when the H_2_ volume concentration increases from 0.2 to 100%. A solid-state sensor for detecting H_2_ gas concentrations as small as 0.075% in volume has been developed based on commercial PVDF pyroelectric films sputter-coated with Pd (or an aluminum-nickel (Al-Ni) double layer).^195^ The high resolution is related to the variation of the pyroelectric coefficient of the film due to electrostatic interactions of adsorbed H_2_ ions with the PVDF matrix upon hydrogenation and selective absorption by the metallic coating.

PVDF/iron vanadate (FeVO_4_) porous layers produced by a doctor blade have been successfully tested for oxygen (O_2_) sensing devices, being the best sensitivity (resistance of O_2_/resistance of nitrogen (N_2_)) of 0.29 ± 0.01 obtained at an optimal working temperature of 250 °C.^196^ The trapping of electrons can explain such high sensitivity to adsorbed O_2_ species and the resulting band bending that caused the measured resistance change.

The AC/DC electrical properties of Li and PVDF/titanium (Ti) codoped nickel oxide (NiO) composites were studied and optimized for their use as ammonia (NH_3_) sensors.^197^ It was discovered that the response time (decreasing from ∼250 s to ∼30 s with increasing temperature from 300 to 420 °C) and sensitivity (decreasing from ∼0.12 to ∼0 ppm^–1^ with increasing temperature from 300 to 420 °C) of the sensor was strongly dependent on temperature.

**Figure 11:**
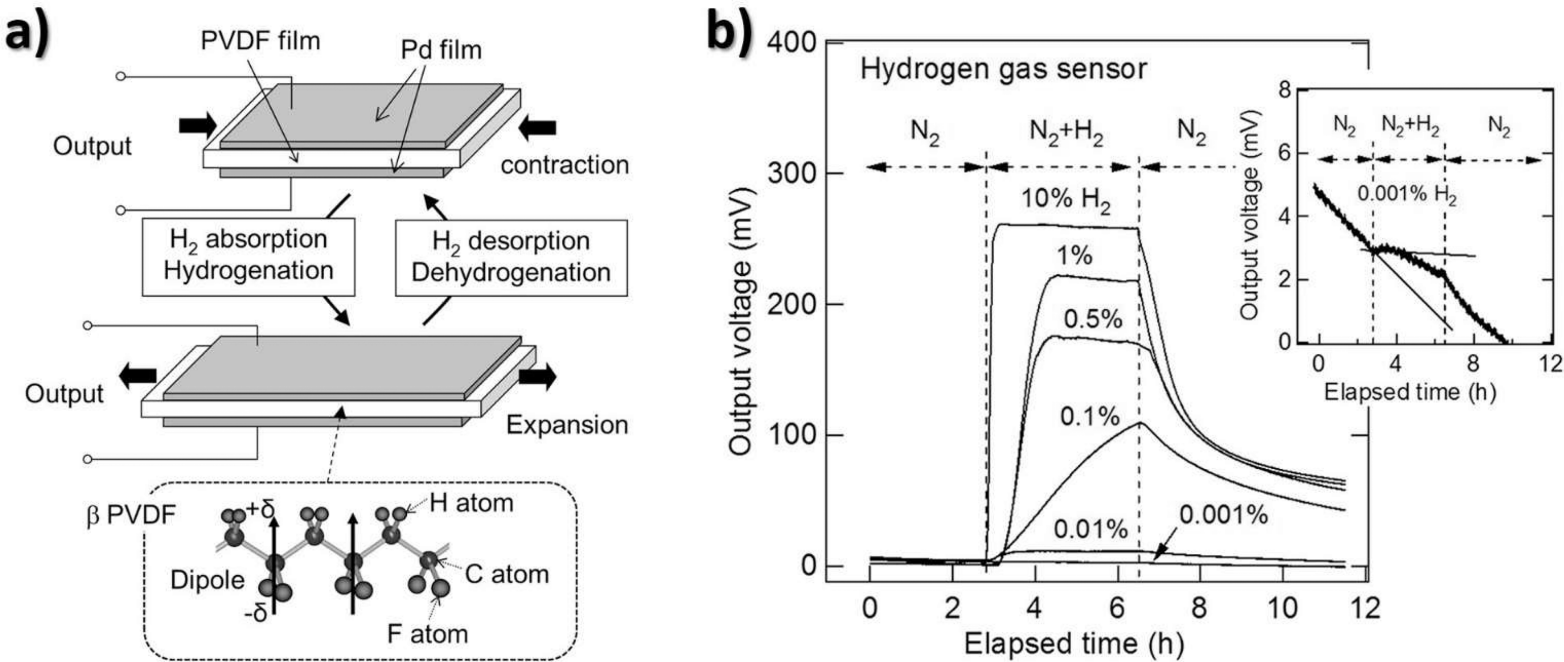
(a) Schematics of the piezoelectric H_2_ sensor composed of PVDF and Pd. (b) Output voltages (mV) of the proposed gas sensor as a function of time (h). The inset reveals the magnified response curve collected for 0.001% H_2_ concentration. Reproduced with permission from ref (199). Copyright 2017 Elsevier.

After proposing a new type of organic H_2_ gas sensor in which a β-PVDF film was coated with thin films of Pd on both sides with a sensitivity of ∼50 mV·L^–1^·min^–1^,^198^ Imai et al.^199^ (Figure 11a,b) tested the response characteristics of the sensors (response at H_2_ exposure ∼100%, detection sensitivity of ∼250 mV·L^–1^·min^–1^ and recovery time of ∼500 s), demonstrating that the characteristics of the sensor response depends on the PVDF’s microstructure.^199^

In the field of resonators, a mechanical resonator made of a thin glass plate driven by ferroelectric PVDF polymer foils was optimized for N_2_-sulfur hexafluoride (SF_6_) gas flow and humidity measurements.^200^ The particular 2/0 mode vibrational deflection of the plate was selected, as it ensures high sensitivity (the resolution was in the order of 2% relative humidity and 0.2 m·s^–1^ gas flow), appropriate for the determination of both the density of gases and humidity in the air.

Chen et al.^201^ also applied a resonator composed of ZnO piezoelectric stack, a tungsten/silicon dioxide (W/SiO_2_) Bragg reflector, and a PVDF sensing material for the detection of nerve gas. The testing results revealed that the proposed resonator has high sensitivity (718 kHz·ppm^–1^), reversibility (15 min recovery), and reproducibility (over 30 days) in the sensing of nerve gas.

More recently, Xu et al.^202^ incorporated porous PVDF/Gr membranes on surface acoustic wave sensors to increase the sensor’s response time. The sensitivity of the dimethyl methyl phosphonate (DMMP) sensor reached the remarkable value of −1.407 kHz·ppm^–1^, the response time and recovery time of the sensor being improved 4.5 and 5.8 s, respectively, with the incorporation of the PVDF/Gr membranes. The much improved performance was explained through increased adsorption as a result of the electrostatic interaction between DMMP and PVDF chains.

#### 3.1.5 Biomedical Sensors

The capability of transforming electrical stimuli into mechanical response, and mechanical stimuli to electrical response, in combination with the excellent physical, chemical, and mechanical characteristics of PVDF and its copolymers, have been increasingly applied in the biomedical field to develop biosensors and tools for health monitoring.^8^

One of the most cited work on the use of PVDF-based biosensors is the one from Manesh et al.,^203^ regarding the evaluation of electrospun PVDF/poly(aminophenylboronic acid) (PAPBA) composite nanofibrous membranes as a glucose sensor. The sensor worked on the 1–15 mM glucose concentration range with a sensitivity of 2.3 μA·mM^–1^, linearity of 0.997, and a response time of less than 6 s, all those features being stable over 50 days. Such work opened the way for the electrospinning technology to be extended to the fabrication of other sensors through judicious loading of sensing materials into the polymer-based fibrous matrix.

A similar strategy was followed by Zhang et al.^204^ by adding multiwalled carbon nanotubes (MWCNTs) and platinum (Pt) nanoparticles to PVDF. After electrospinning, polymer-based nanofibrous membranes were obtained with highly stable biosensing properties (with selective detection of both hydrogen peroxide (H_2_O_2_) and glucose with a sensitivity of −0.2 μA·mM^–1^). Such composite structures can be easily used in other technological applications such as energy storage, cytology, and tissue engineering.

Tanaka et al. designed a haptic finger using PVDF piezopolymer films as a sensory receptor.^205^ The suitability of the proposed sensor to monitor human skin conditions was investigated after sliding the sensor over skin surfaces from 30 people, being found that the two parameters (index of roughness and hardness) generated using such haptic finger displayed good agreement with subjective/clinical assessments of the tested skin conditions. PVDF sensor output from 0 to ∼30 mV allowed conclusion that from all 30 persons who participated in the study, 5 exhibited xerosis, 7 atopic dermatitis, and 2 psoriases. Such results were encouraging for looking at the suitability of similar devices in other fields, such as the cosmetic area, where the variations of skin properties are more subtle and more challenging to be detected.

Knowing that problematic and recurrent sleep apnea upsets the sleep of humans and that it can lead to sleep disorders such as severe snoring, fatigue, daytime sleepiness, and systemic hypertension, Hwang et al.^206^ presented a sleep apnea monitoring method based on PVDF sensors (4 × 1 array with ∼1.1 mm thick) for continuous and accurate monitoring of apneic events that occurred during sleep. The correlation coefficient of the sensors for the apnea–hypopnea index (AHI) values was determined to be 0.94. The areas under the receiver operating curves at three AHI threshold levels (>5, >15, and >20) for sleep apnea diagnosis were 0.98, 0.99, and 0.98, respectively, and most importantly, for minute-by-minute apnea detection, the method classified sleep apnea with an average sensitivity of 72.9%, specificity of 90.6%, accuracy of 85.5% and kappa statistic of 0.60. All of these experimental results validate the PVDF sensor system for the monitoring/detection of sleep apnea in both home and ambulatory environments.

**Figure 12:**
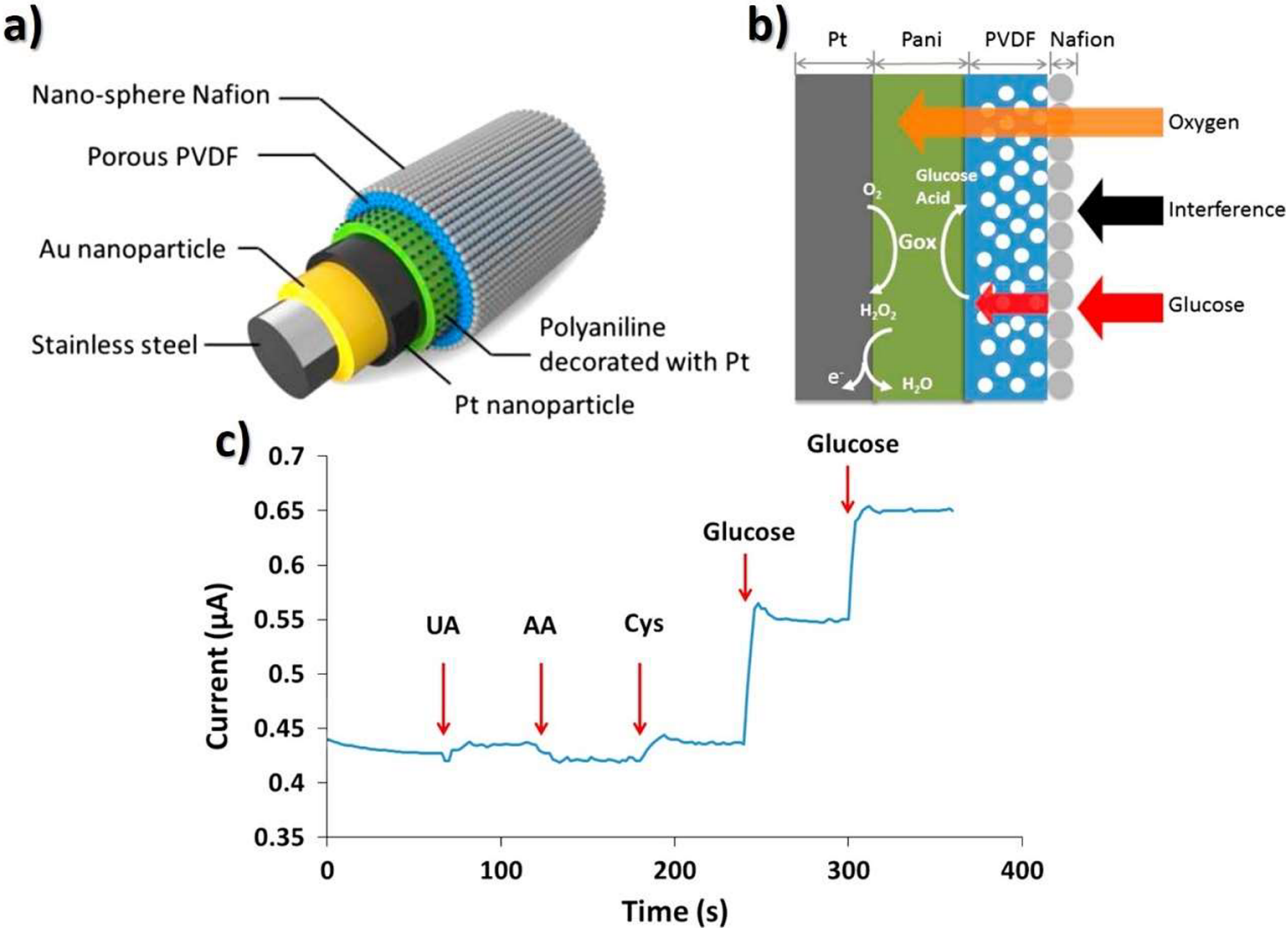
Schematic representation of the glucose sensing needle. (a) Layered nanostructures and (b) operation principle. (c) Relation between current (μA) and time (s) with successive additions of 0.5 mM glucose (twice), 0.1 mM uric acid (UA), 0.1 mM ascorbic acid (AA), and 0.1 mM l-cysteine (Cys) under 0.65 V electrode potential. Reproduced with permission from ref (207). Copyright 2015 Elsevier.

Being the continuous glucose sensing with reliable *in vivo* performance expected to improve glucose concentration regulation and thus reduce the number of complications related to diabetes mellitus, PVDF-Nafion nanomembranes coated microneedles were produced for *in vivo* transcutaneous implantable glucose sensing (Figure 12).^207^

The obtained porous structure with a nanosphere top layer fabricated using one-time deposition avoided multiple dipping and subsequent loss of enzyme activity, leading to high selectivity, low response time (30 s), high sensitivity (∼0.23 μA.mM^–1^), and high linearity (*R*^2^ = 0.998) in the 0–20 mmol·L^–1^ range. Additionally, reliable *in vivo* test results in mice were reported.

Aiming also to detect glucose, Xing et al.^208^ introduced nickel(II) hydroxide (Ni(OH)_2_) and CNTs into a PVDF matrix, obtaining a highly stable sensing material whose stability was confirmed by cyclic voltammetry measurements in sodium hydroxide solution (NaOH) (0.50 M, scan rate 100 mV·s^–1^). The PVDF-based composite films maintained the electrocatalytic activity of the nano-Ni(OH)_2_ and, for such reason, was used to fabricate a nonenzymatic biosensors for electrochemical detection of glucose. Amperometric measurements revealed that the proposed sensor exhibited good anti-interference properties toward some substances (maltose, fructose, urea, and AA), with a reported detection limit of 0.023 mM and a wide linear range from 0.25 to 39.26 mM (*R*^2^ = 0.998), which are comparable with commercially available blood glucose sensors. The calculated sensitivity was 0.65 μA·mM^–1^ with a deviation of less than 5%.

Another glucose sensing device was developed using a capacitive biosensor based on PVDF thin film, consisting of a PVDF film sandwiched between two Ag electrodes.^209^ The sensor was evaluated for glucose concentrations in the 0.013–5.85 M range and various glucose oxidase (GOx) enzyme concentrations between 4882.8 and 2.5 million units·L^–1^, reported that the device output increased from 0 to ∼5.5 μV with increasing glucose concentration up to 5.85 M, showing a detection limit of 1.3 × 10^–2^ M.

Regarding antigen sensing, Sanguino et al.^210^ reported the use of PVDF Immobilon-P as a sensitive layer coupled with a transducer to function as affinity immunosensors, able to distinguish phosphate-buffered saline (PBS) buffer and antigen horseradish peroxidase (HRP) solutions, based on the capacitance variations (PBS, 0 to −0.6 pF; and HRP, 0 to 0.1 pF). Such measurements could be taken at a fixed frequency, making the sensor instrumentation particularly simple and easily scalable for practical applications such as clinical diagnosis, food monitoring, industrial controls, and environmental measurements.

Once nucleic acid testing (NAT) represents stable, safe, selective, and specific detection of infectious and inherited diseases, a new diaphragm mass biosensor based on PVDF piezoelectric film was developed for detecting nucleic acids.^211^ Experimental results showed that the mass sensitivity was 0.185 kHz·μg^–1^, obtained for a diaphragm with a diameter of 5 mm. Additionally, the value of frequency shift was found to be directly proportional to the content of the target solution. Such performance validated the PVDF-based sensor for low-cost real-time fabrication of NAT tools.

With respect to electrochromic sensing, Santiago-Malagón et al.^212^ used a PVDF-based ion-gel electrolyte (poly(VDF-*co*-HFP), IL 1-ethyl-3-methylimidazolium trifluoromethanesulfonate, EMIM-Tf, and potassium triflate) to protect the cathode display and to provide an adequate chemical environment for the operation of a skin-patch electrochromic lactate biosensor. Such a protection layer avoided the bleaching of the cathode by acids (ascorbic and uric). The sensor was found to work with lactate concentrations in the range of 0–10 mM with a contrast ratio of 1.43, up to 24 min response time, and 85% of the color change displayed within 10 min. The skin-patch electrochromic lactate biosensor represents a promising route for controlling target molecules that are exerted through perspiration.

#### 3.1.6 Environmental Sensors

Among several materials used for the fabrication of polymer-based environmental sensors, PVDF is one of the most commonly used due not only to its excellent mechanical strength and high thermal and chemical resistance but also because this polymer withstands chlorine disinfection.^213^

Aiming to evaluate random environmental vibrations (such as wind flow, waterfall, or transportation of vehicles), a large area, highly sensitive, and flexible pressure sensor has been produced based on electrospun Ce^3+^/PVDF/Gr composite nanofibers.^214^ The developed Ce^3+^/PVDF/Gr composite sensing device could detect pressure as low as ∼2 Pa with high sensitivity (30 mV·Pa^–1^). Furthermore, the Ce^3+^/PVDF/Gr composite sensor could also be used as an effective nanogenerator as it can generate an output voltage of 11 V with a current density of ∼6 nA·cm^–2^ upon repetitive application of mechanical stress, making this device self-powered.

Also, from a “self-powered device” perspective, Guo et al.^215^ developed a stretchable sensor fabricated by serpentine PVDF film for multiple dynamic monitoring, which can efficiently detect various mechanical stimuli relevant to specific environmental or biological species. The serpentine device, composed of three layers, with two aluminum layers coated on a piezoelectric PVDF film, exhibited a sensitivity of 6mV·stretch^–1^ and a maximum voltage of 350 mV at a frequency of 10 Hz.

**Figure 13:**
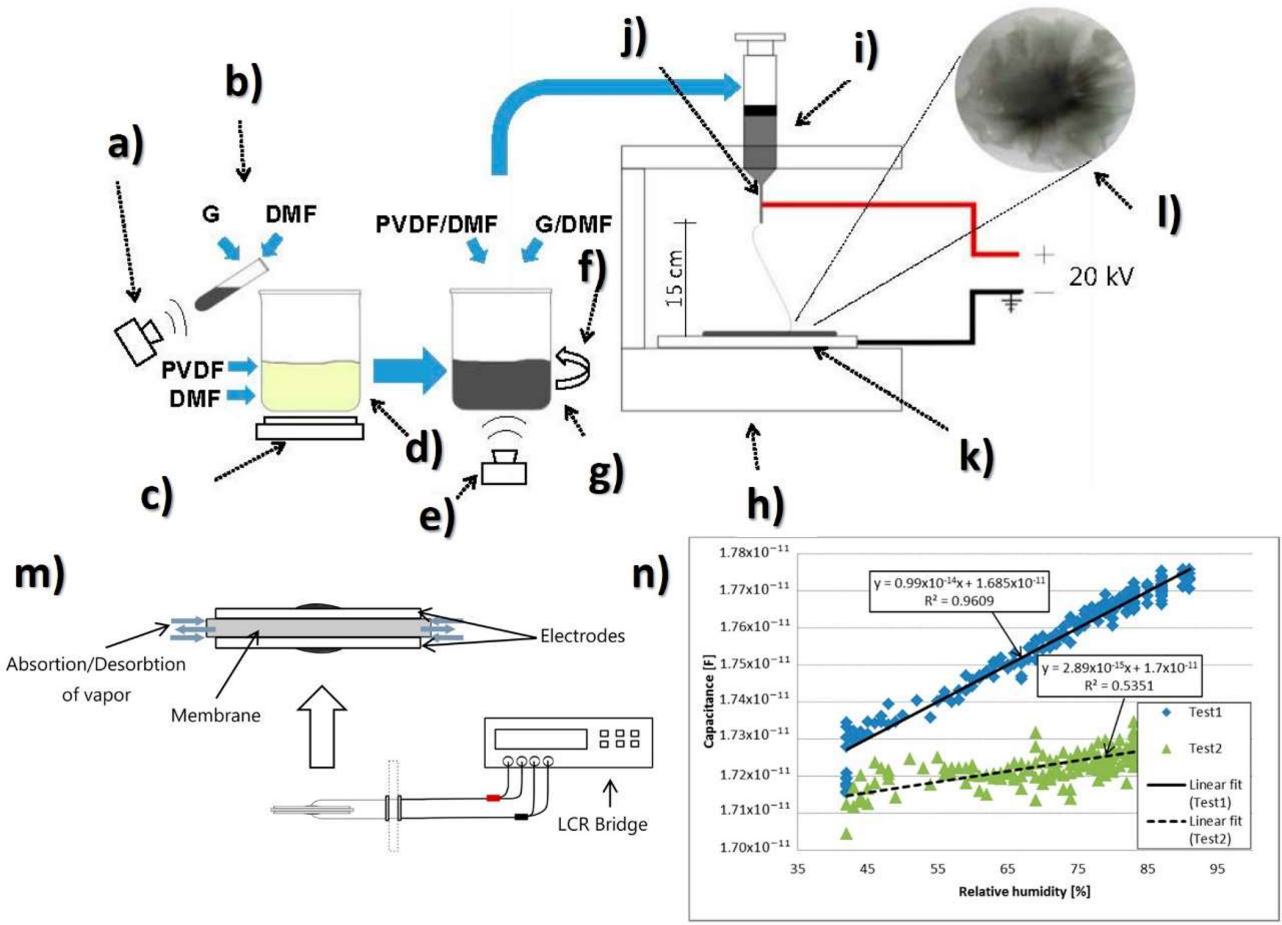
(a) Schematics of the production procedure: (a) sonication, (b) Gr/DMF solution, (c) electric heater, (d) PVDF/DMF solution, (e) sonication, (f) stirring, (g) PVDF/Gr/DMF solution, (h) electrospinning equipment, (i) needle, (j) syringe tip, (k) collector plate, and (l) electrospun membrane. (m) Scheme of the humidity sensing principle. (n) Capacitive response as a function of the relative humidity of the PVDF-based sensor. Reproduced with permission from ref (216). Copyright 2017 Multidisciplinary Digital Publishing Institute.

Once humidity sensors are widely required in agriculture, environmental conservation, and climatology, PVDF/Gr membranes were evaluated as capacitive humidity sensors (Figure 13).^216^

PVDF blended with Gr was developed to improve the PVDF electrical properties, allowing the use of PVDF/Gr membranes as capacitive humidity sensors. The observed good response time (18 s), high sensitivity (0.0463 pF/% of relative humidity), repeatability, linearity (*R*^2^ = 0.993), and low noise of the PVDF/Gr composite membrane sensors open other possible applications such as filtration, growth of living tissues, and prosthetics applications.

To explore underwater environments and operate in underwater missions, sensing the surrounding environment is an essential topic for developing innovative underwater robots. Once this issue is particularly sensitive on robotic fish, a PVDF pressure sensor was developed^217^ and integrated with a small water-proofed charge amplifier. The pressure PVDF-based sensor was optimized in a water tank, reporting a sensitivity of 0.071 Pa·mV^–1^. This sensor can be used to study other fishes and their actions/reactions in real-time.

Other environmental stimuli, such as light or pressure, can also be monitored by using both piezoelectricity and pyroelectricity of PVDF.^218^ Multipiezo/pyroelectric sensors with transparent electrodes (Ag nanowires and PEDOT:PSS) exhibit a pressure and light sensitivity of 80 mV·Pa^–1^ and 42 V·cm^2^·W^–1^, respectively. These values are favorably compared to the ones of Al-based electrodes, with the advantage of defining the electrodes directly on the sensitive foil. The reported optimized sensors reach root-mean-square powers for the piezoelectric effect and pyroelectric effect of ∼1 μW and ∼0.42 μW, respectively, for an active PVDF area of 8 cm^2^. This strategy makes it easy to detect and quantify all kinds of environmental properties such as humidity, pressure, heat, light, or vibration.

More recently, Jang et al.^219^ developed PVDF/ZnO-based hydrazine sensors. As a working principle, ZnO was previously functionalized with poly(VDF-*co*-HFP) to expose the polymeric chains to hydrazine, allowing physical H_2_ bonding/interactions that induced a change in the charge transfer properties of the ZnO films and improved the sensing behavior of the device. Hydrazine is regarded as a toxic and carcinogenic chemical that damages the liver, lungs, kidneys, and central nervous system (CNS), and for such reason, the Environmental Protection Agency (EPA) has limited the threshold in drinking water to less than 10 ppb (∼0.3 μmol·L^–1^). Thus, the study reported by Jang et al. is in line with the need to define suitable sensing strategies of the toxic hydrazine. The PVDF/ZnO-based sensor exhibited hydrazine detection limits up to 0.01 nM (sub-ppt level) and reproducibility over 96%.

#### 3.1.7 Magnetic Sensors

Besides the nucleation of the electroactive phases of PVDF (such as β or γ), the addition of fillers induces additional effects that bring added value to the use of PVDF-based nanocomposites for technological applications.^11^ As an essential family of PVDF-based nanocomposites, magnetic nanocomposites with magnetically responsive features have attracted increasing attention because of their magnetic functionality and wireless activation.^220,221^

Despite PVDF-based magnetic composites find many major applications as actuators,^222^ vibration control,^223^ ultrasonic transducers,^224^ batteries,^225^ filters,^226^ chemical warfare protection,^227^ and in the biological field,^228^ their impact with respect to magnetoelectric sensing applications are particularly interesting.^156^ For example, the addition of magnetostrictive fillers into the PVDF matrix allows the production of all-printed multilayer magnetic-responsive materials with improved magnetoelectric response suitable for sensing devices,^229^ transparent magnetoelectric materials for advanced invisible sensing applications,^230^ and anisotropic magnetoelectric sensors with good linearity (*R*^2^ = 0.995) with application potential on digital compasses, global positioning system (GPS) devices, and biomedical sensing.^231^

It has been reported that the introduction of different nanoparticles into a poly(VDF-*co*-TrFE) matrix, such as Zn_0.2_Mn_0.8_Fe_2_O_4_ (ZMFO), CoFe_2_O_4_, and Fe_3_O_4_, do not change the piezoelectric response of the polymer matrix (∼−28 pC·N^–1^) but leads to distinct magnetoelectric responses,^232^ such as 6.5 mV·cm^–1^·Oe^–1^, at an optimum magnetic field of 0.26 T, and 0.8 mV·cm^–1^·Oe^–1^, at an optimum magnetic field of 0.15T, for poly(VDF-*co*-TrFE)/CoFe_2_O_4_ and poly(VDF-*co*-TrFE)/Fe_3_O_4_ composites, respectively. In contrast, the magnetoelectric response of poly(VDF-*co*-TrFE)/ZMFO showed no hysteresis and high dependence on the ZMFO filler content. Such findings allow further tailoring of the magnetoelectric sensing properties for specific magnetic environments.

**Figure 14:**
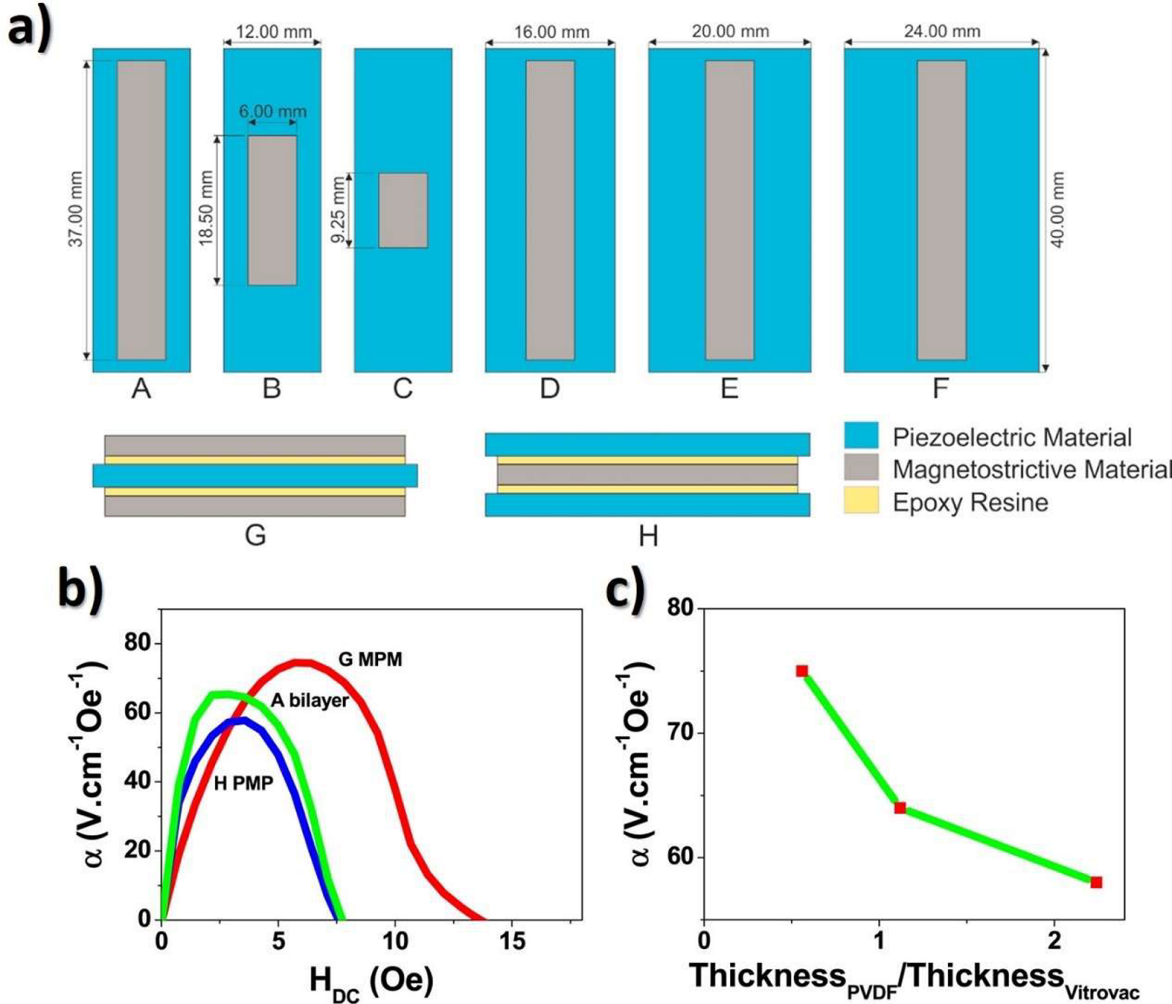
(a) Schematic representation of Vitrovac/PVDF magnetoelectric composites produced in the study (A–F). Representation of the lateral view of the three-layer composites (G,H). (b) magnetoelectric response obtained from laminates with bilayer composite (sample A), three-layer magnetostrictive–piezoelectric–magnetostrictive (MPM) (sample G) composite, and three-layer PMP (sample H) configurations. (c) Magnetoelectric response obtained as a function of the different aspect ratios. Reproduced with permission from ref (233). Copyright 2015 Elsevier.

Regarding laminated composites, the combination of PVDF and Vitrovac 4040 materials on trilayered and bilayered magnetoelectric flexible composite structures of varying geometries and sizes allowed to optimize the magnetic sensitivity of polymer-based magnetic sensors^233^ (Figure 14).

From the magnetoelectric measurements, it was confirmed that trilayered composite structures, i.e., MPM type, revealed a higher magnetoelectric response (75 V·cm^–1^·Oe^–1^) than the bilayer structure (66 V·cm^–1^·.Oe^–1^). Additionally, the magnetoelectric voltage coefficient decreased with an increasing longitudinal aspect ratio between PVDF and Vitrovac layers (from 1.1 to 4.3), with a maximum magnetoelectric voltage coefficient of 66 V·cm^–1^·Oe^–1^.

A PVDF/carbonyl magnetic composite film was produced to be used as magnetic field and deformation bisensor.^234^ It was reported that when the magnetic field varied from 0 to 600 mT, the generated magnetoelectric charges of PVDF/carbonyl film increased from 0 to 676 pC (sensitivity of ∼1.13 pC·mT^–1^). The quantitative relationship between the magnetic field and magnetoelectric charges was obtained by the polynomial fitting method, and the correlation coefficient was up to ∼0.97. Additionally, and experimentally proving the bisensing properties, the piezoelectric charges under 2, 4, 6, 8, and 10 mm bending displacement were 3.0, 9.6, 14.9, 18.6, and 24.6 pC, respectively. Due to the optimized piezoelectricity, excellent stability, lightweight, and high flexibility, PVDF/carbonyl magnetic composite films showed promising applications in deformation sensors and magnetic field sensors.

A different magnetoelectric coupling/sensing technology was introduced by Brito-Pereira et al., where a poly(VDF-*co*-TrFE)/wax/CoFe_2_O_4_ magnetic sensing device was screen printed, revealing a *R*^2^ of 0.9991 and a sensitivity of 30 V·T^–1^.^235^ The device was able to harvest magnetic energy with a maximum output power density of 9.7 mW·cm^–3^, demonstrating self-powered sensing capability. Contrary to the traditional magnetoelectric effect on composites (piezoelectric effect coupled to magnetostrictive effect through mechanical interactions) in the magnetoelectric effect reported in this study, the piezoelectric poly(VDF-*co*-TrFE) is mechanically activated through the movement of a coupled layer rich in magnetic components (wax/CoFe_2_O_4_), which in turn was activated by the magnetic force of an approaching magnet. Such magnetoelectric coupling and sensing/harvesting performance allied to Bluetooth connectivity offers excellent potential for incorporation in IoT-related applications.

In an environmentally friendlier approach, and knowing that due to its highest piezoelectric response among polymers and capability to crystalize into the piezoelectric β-phase, poly(VDF-*co*-TrFE) is the most suitable polymer for polymer-based magnetoelectric sensing materials (with over 80% of the total reports), Lima et al.^236^ evaluated the possibility to change the traditionally used DMF solvent (a toxic chemical) by *greener* ones, demonstrating that poly(VDF-*co*-TrFE)/CoFe_2_O_4_ nanocomposites can be successfully prepared from solution using environmentally friendlier solvents (DMSO, DMPU, and triethyl phosphate (TEO)). It was shown that the prepared composite films, with a maximum magnetoelectric voltage coefficient of 35 mV cm^–1^·Oe^–1^ and a maximum sensitivity of 2.2 mV·T^–1^ are suitable for applications, highlighting the path for a new generation of more sustainable magnetoelectric sensors.

#### 3.1.8 Other Sensors and Materials

Due to the already mentioned outstanding properties such as excellent chemical resistance, thermal stability, small acoustic impedances, high electrical insulation, high flexibility, and membrane forming features, PVDF-based sensors are effectively used in a vast variety of other application areas.^22^

More recently, PVDF-based materials have gained considerable attention in electromagnetic shielding. Due to the ever-increasing use of electronic devices and wireless technologies, electromagnetic interference (EMI) has become a significant concern in the framework of the digitalization of society. PVDF shows great potential as an effective EMI shielding material in the form of composites, that have already demonstrated excellent EMI shielding performance through the ability to block or absorb electromagnetic waves, and to protect sensitive electronic components from environmental interference.^237,238^

These composites have been produced from various fillers, such as conductive (carbonaceous), magnetic (ferrite), and ceramic (ZnO) particles, among others, where the morphology and size of the fillers, their dispersion and other parameters such as the thickness of the composite play an important role in the electromagnetic shielding performance.^238^ To improve its performance, it is essential to further understand and tune the interfaces established between the fillers and the PVDF matrix.

Pressure sensors are an increasingly popular application area of PVDF-based materials, the work of A. Shirinov et al.^167^ being an excellent example of this, once it reported a pressure sensor with a PVDF foil that is a low-cost alternative for the measurement of pressure changes (∼3 V output for a ∼200 kPa input) in chemically aggressive media with a limited need for accuracy. This type of sensor can be used in the biomedical area, namely in endoscopic graspers with high sensitivity (50 V·N^–1^), an extensive dynamic range (near static up to a few MHz), and a high signal-to-noise ratio.^239^

In the cases that sensors are required to measure small deformations, such as wearable cardiorespiratory signal sensor devices for monitoring sleep conditions, electronic components (amplifiers) can magnify the signal to a certain level.^240^ The applicability range of PVDF sensors for vital signal measurements was increased by Y. Wang et al.,^241^ opening new directions such as sensing garment pressure, blood pressure, heartbeat rate, respiration rate, and accidental impacts on the human body. PVDF nanofibrous fabrics were prepared by electrospinning with excellent sensitivity (42 mV·N^–1^) and high response to external mechanical forces (∼4 N).

By adding 2 wt % of functionalized Gr into PVDF, Eswaraiah et al.^242^ optimized the strain sensing performance under tensile loads, useful for applications such as advanced aerospace, mechanical, bionics, and medical technologies. With the use of different carbonaceous nanofillers (few-layer graphene (FLG), graphene nanoplatelets (G-NPL), graphene oxide (GO), reduced graphene oxide (rGO), and single-walled carbon nanohorns (SWCNH)), Costa et al.^243^ increased the electrical conductivity 9 orders of magnitude, from σ ∼5 × 10^–11^ S·m^–1^ from pure PVDF to σ ∼1 × 10^–2^ S·m^–1^ for rGO/PVDF composites, with 5 wt % nanofillers, reaching high linearity and significant piezoresistive gauge factors of ∼11 for deformations between 0.5 and 2 mm, very useful for strain sensing applications.^243^

Table 2 summarizes the application area, materials, and FOM for the different sensor types discussed in this section.

**Table 2:** Summary of Representative PVDF-Based Sensors, Materials and Applications Areas, Indicating Also the Reported FOM: Sensitivity (*s*), Detection Range (*dr*), Standard Deviation (*sd*), Sensor Output (*so*), and Detection Limit (*dl*)

application area	materials	FOM/mechanism	ref
Capacitive Sensor			
human–machine interface	PVDF	*s* = 0.6 Δ*C*/*C* mechanical to capacitive	(163)
sport sensing	PVDF/TiO_2_	*s* = 0.5 Δ*C*/*C* mechanical to capacitive	(165)
textile sensing	PVDF/[EMIM][TFSI]	*s* = 200 Δ*C*/*C* mechanical to capacitive	(164)
SO_2_ sensing	PVDF/UiO-66-NH_2_	*s* = 0.01 Δ*C*/*C* mechanical to capacitive	(166)
Temperature Sensor			
microfluidic	PVDF/PMMA	*s* = 5 mV·°C^–1^ pyro- to electric	(168)
human skin	PVDF/ZnO/Gr	*s* = 13 mΩ·°C^–1^ pyro- to resistive	(170)
E-skin	PVDF	*s* = 2 mV·°C^–1^ pyro- to electric	(171)
safety monitoring	PVDF/PTFE/CU	*s* = 2.1 mV·°C^–1^ pyro- to electric	(172)
robotic	poly(VDF-*co*-TrFE)/ BaTiO_3_	*s* = 15.34 mV·°C^–1^ pyro to electric	(173)
physiological	PVDF/Gr	*s* = 0.34 pC·°C^–1^ pyro- to capacitive	(174)
robotic	PVDF/PET/Ag	*s* = 0.478 V·°C^–1^ pyro- to electric	(175)
pH Sensor			
biosensing	PVDF/ZnO	*s* = 0.06V·pH^–1^ ionic to electric	(188)
sensitive membranes	PVDF/acrylic acid	*s* = 0.6 mL·cm^–2^·min^–1^·pH^–1^ electrostatic to colorimetric	(190)
multifunctional membranes	PVDF/poly(methyl methacrylate-2-hydroxyethyl methacrylate-acrylic acid)	*s* = 0.03 mL·cm^–2^·min^–1^·pH^–1^ electrostatic to colorimetric	(191)
colorimetry	PVDF/tetrabromophenol blue/phenol red	*s* = 1.01 ΔH/ΔpH ionic to colorimetric	(192)
wound monitoring	PVDF/PANI	*s* = 8.53 mV /pH^–1^ ionic to electric	(186)
Gas Sensor			
H_2_	PVDF/Pd	*d*_r_ = 0.2–100% photopyroelectric to optical	(194)
H_2_	β-PVDF/Pd	*s* = 250 mV/L^–1^·min^–1^ electrostatic to capacitive	(199)
O_2_	PVDF/FeVO_4_	*s*(RO_2_/RN_2_) = 0.29 electrostatic to resistive	(196)
NH_3_	PVDF/Ti/NiO	*s* = 0.12 ppm^–1^ electrostatic to electric	(197)
N_2_-SF_6_	PVDF	*s* = 2% relative humidity and 0.2 m·s^–1^ gas flow mechanical to electric	(200)
nerve	PVDF/ZnO/ W/SiO_2_	*s* = 718 kHz·ppm^–1^ mechanical to electric	(201)
DMMP	PVDF/Gr	*s* = 1.407 kHz·ppm^–1^ acoustic to electric	(202)
Stress Sensor			
shear	PVDF/rubber	*s* = 3 nC·N^–1^·m^–1^ mechanic to capacitive	(179)
medial–lateral shear	PVDF	*s* = 55.2 mV·N^–1^ mechanic to electric	(180)
internal	PVDF/PET	*s* = 1.32 MPa·V^–1^ mechanic to electric	(181)
internal	PVDF	*s* = 50 pC·mm^–1^ mechanic to capacitive	(185)
interfacial	PVDF	*s*_d_ = 4–8% mechanic to electric	(182)
shock wave	PVDF	*s* = 49.2 GN·C^–1^ mechanic to electric	(183)
ballistic	PVDF	*s* = 53 pC·N^–1^ mechanic to capacitive	(184)
Vibration Sensor			
string	PVDF	*s* = 0.2 mV·με^–1^ mechanic to electric	(152)
Bio Sensor			
glucose	PVDF/PAPBA	*s* = 2.3 μA·mM^–1^ amperometric (oxidation/redution to electric)	(203)
glucose	PVDF-Nafion	*s* = 0.23 μA·mM^–1^ amperometric	(207)
glucose	PVDF/ Ni(OH)_2_/CNTs	*s* = 0.65 μA·mM^–1^ amperometric	(208)
glucose	PVDF/Ag	*so* = 5.5 μV capacitive to electric	(209)
H_2_O_2_ and glucose	PVDF/CNTs/Pt	*s* = 0.2 μA·mM^–1^ amperometric	(204)
haptic finger	PVDF	*so* = 30 mV mechanic to electric	(205)
sleep apnea	PVDF	*s* = 72.9% mechanic to electric	(206)
antigen	PVDF Immobilon-P	*s* = 0.1 pF electrostatic to capacitive	(210)
NAT	PVDF	*s* = 0.185 kHz·μg^–1^ electrostatic to electric	(211)
Lactate	poly(VDF-*co*-HFP)/EMIM-Tf/potassium triflate	*dl* = 10 mM electric to colorimetric	(212)
Environmental Sensor			
random vibrations	Ce^3+^/PVDF/Gr	*s* = 30mV·Pa^–1^ mechanic to electric	(214)
environmental stimuli	PVDF	*s* = 6 mV·stretch^–1^ mechanic to electric	(215)
humidity	PVDF/Gr	*s* = 0.0463 pF·%^–1^ of relative humidity electrostatic to capacitive	(216)
pressure	PVDF	*s* = 0.071 Pa·mV^–1^ mechanic to electric	(217)
light/pressure	PVDF/Ag/PEDOT:PSS	*s*_light_ = 80 mV·Pa^–1^*s*_pressure_ = 42 V cm^2^·W^–1^ mechanic/photonic to electric	(218)
hydrazine	PVDF/ZnO	*dl* = 0.01 nM electrostatic to electric	(219)
Magnetic Sensors			
magnetoelectric, printed	poly(VDF-*co*-TrFE)/PVDF/CoFe_2_O_4_	*s* = 1.0 mV·T^–1^ magnetic to electric	(229)
magnetoelectric, transparent	poly(VDF-*co*-TrFE)/Fe_72.5_Si_12.5_B_15_	*s* = 247 mV·T^–1^ magnetic to electric	(230)
magnetoelectric, anisotropic	poly(VDF-*co*-TrFE)/ CoFeOOH	*s* = 0.008 mV·T^–1^ magnetic to electric	(231)
magnetoelectric, isotropic	poly(VDF-*co*-TrFE)/CoFe_2_O_4_	*s* = 0.125 mV·T^–1^ magnetic to electric	(232)
magnetoelectric, laminated	PVDF/Vitrovac 4040	*s* = 550 V·T^–1^ magnetic to electric	(233)
deformation bisensor	PVDF/carbonyl	*s* = 1.13 pC·mT^–1^ magnetic to capacitive	(234)
magnetoelectric, nonmagnetostrictive	poly(VDF-*co*-TrFE)/wax/CoFe_2_O_4_	*s* = 30 V·T^–1^ magnetic to electric	(235)
magnetoelectric, greener	poly(VDF-*co*-TrFE)/ CoFe_2_O_4_	*s* = 2.2 mV·T^–1^ magnetic to electric	(236)
Other Sensors			
pressure sensor for chemically aggressive media	PVDF	*s* = 50V·N^–1^ mechanic to electric	(167)
wearable cardiorespiratory signal sensor	PVDF	*s* = 42.00 mV·N^–1^ mechanic to electric	(240)
aerospace, mechanical, bionics, and medical technologies	PVDF/Gr	*so* = 200 MPa mechanic to electric	(242)
piezoresistive strain sensing	PVDF/carbonaceous nanofillers	*s* = 5 × 10^–11^ S·m^–1^ mechanic to electric	(243)

Considering the applicability of PVDF-based materials in different sensors types and all the promising advances summarized in Table 2, the time ahead is even more challenging and encouraging for the development of low-cost, low-waste, low-energy sensors, with improved performance, microstructures, and integration into both rigid and flexible substrates.

### 3.2 Actuators

PVDF and its copolymers have been extensively studied and applied in the field of actuators,^244^ commonly defined as systems able to convert an energy from an external source into a mechanical energy in a controllable way.^245^ Particularly, actuators based on smart materials present the ability to modify their shape when environmental changes occur (e.g., pH, electrical signals, magnetic inputs, temperature variations, among others) by transducing the specific input into motion.^246^

Actuators have been attracted significant attention for applications owing to their strong potential in different fields, including microelectronics and fabrication, soft robotics, haptics, microfluidic systems, and medical devices.^245^

Different types of actuators have been developed based on different types of materials, such as shape memory alloys (SMA), piezoelectric, electroactive polymers (electronic and ionic), electrostatic, magnetoactive, and ferrofluids. Among them, polymer-based actuators have gained special attention mainly due to their tunable physical and chemical properties, easy processability in different morphologies and shapes, and the wide range of physicochemical inputs promoting conformation variations, and the broad range of stress and strain outputs.^246^ The actuation mechanism of polymer-based actuators is classified attending to the input stimulus: electric field, magnetic field, ionic, pneumatic, and thermal, electromechanical polymer-based actuators being the most extensively studied.

Significant efforts have been devoted to develop electromechanical actuators with rapid responses at low applied voltages and controllable displacement and frequency. Additionally, increased attention has been devoted to fabricating actuators with reduced size and a wide range of performance.^247,248^ Different materials such as electroactive and shape memory polymers, SMA, and pressurized fluids are commonly employed in the development of actuators.^249^ Among all the above-mentioned materials, electromechanical actuators based on electroactive polymers (EAP) represent one of the most suitable approaches mainly due to their flexibility, lightweight, low-cost, and the ability to achieve higher actuation strains, typically over 300–400%.^250^ Advanced EAP materials have been developed since the beginning of the 1990s and can induce large strains, 2 orders of magnitude higher than electroactive ceramics (EAC) and with a faster response speed, improved resilience, and lower density in comparison with SMA as represented in Table 3.^50^ Due to their intrinsic characteristics, such as piezoelectric responsiveness, PVDF and its copolymers are the electroactive polymers most studied in the field of EAPs actuators. Further, when compared with inorganic piezoelectric materials (e.g., lead zirconate titanate or zinc oxide, among others) these polymers presents large advantages like their high polarity, dielectric constant, easy processing, mechanical robustness and flexibility, and low price. It is noticeable that PVDF and its copolymers can be also easily combined with different materials namely ILs, GO, or magnetic nanoparticles, among others, aiming to the implementation of novel functionalities in the actuators, including self-sensing characteristics.^251^

**Table 3:** Comparison of the Properties of EAP, SMA, and EAC^a^

property	electroactive polymers (EAP)	shape memory alloys (SMA)	electroactive ceramics (EAC)
actuation strain	over 300%	<8% short fatigue life	typically 0.1–0.3%
force (MPa)	0.1–40.0	200	30–40
reaction speed	μs to min	ms to min	μs to s
density (kg·m^–3^)	1000–2500	5000–6000	6000–8000
drive voltage	10–150 V·μm^–1^ for electronic EAP, 1–7 V for ionic EAP	5 V	50–800 V
consumed power	m-W	W	W
fracture behavior	resilient, elastic	resilient, elastic	fragile

a Reproduced with permission from ref (50). Copyright 2019 Institute of Physics Publishing.

Depending on the actuation mechanism, EAP-based actuators are classified into two distinct types: electronic or ionic EAP-based actuators.^250^ The principle of both electronic and ionic EAP-based actuators is activated through the application of an electrical potential, with the main difference relying on the energy transference mechanism. In electronic EAP actuators, the transference of energy is governed by the electronic and/or dipolar structure, while in ionic EAP actuators the energy transference occurs by ions. The difference in the mechanism transference process between both types of the above-mentioned actuators determine different advantages and disadvantages, specifically associated with the actuation force variation and applied voltage.^252^

Among these types of materials, ionic electroactive materials have gained particular attention due to their ability to operate at low driving voltages, their flexibility, lightweight, and low-cost, overcoming the high voltages required to achieve large actuation strains by electronic electroactive materials.^247^Table 4 summarizes the main advantages of electronic and ionic EAP.

**Table 4:** Advantages and Disadvantages of Electronic and Ionic EAPs^a^

EAPs	advantages	disadvantages
electronic	long actuation time in room conditions	high voltage requirement (20–150 MV·m^–1^)
	fast actuation response time (msec)	unidirectional operation due to electrostriction effect
	large actuation force	requiring prestrain at >300%
	high energy density (mechanical)	
	maintain deformation under DC voltage	

ionic	extensive bending (on average)	unstainable strain under DC
	low voltage actuation	slow response time range in seconds

	bidirectional operation with voltage polarity	low actuation force in bending
		electrolyte requires humid condition
		electrolysis occurs at >1.23 V when a system involves water

a Adapted from ref (252).

#### 3.2.1 Electronic Electroactive Polymers-Based Actuators

Within electronic actuators, piezoelectric polymers have been extensively explored as soft actuators instead of piezoceramics due to their higher stretchability, lower density, and shape adaptability.^253^ In fact, piezoelectric polymers can be easily processed into different morphologies and shapes with an ordered crystalline phase and high-density dipole moments.^254^ Besides the low piezoelectricity of polymers compared to piezoelectric crystals and ceramics, their unique mechanical properties expand the interest and application of piezoelectric materials as actuators.^253^

The working principle of EAP-based actuators developed by using piezoelectric polymers relies on the electrical field induced mechanical variations or mechanically induced electrical variations related to dipolar variations. They are characterized by a rapid response time, high applied driving fields, large operation forces, and low displacements in both directions.^254^ Another type of electronic EAP occurs in dielectric elastomers (DE) and rely on the Maxwell stress as actuator principle: when the energy is applied to the polymer of electronic EAP and transferred through the electrodes (covering the material on either side). The resulted Maxwell stress created by the applied electric field leads to the shape material deformation of the polymeric material. However, it is difficult to establish a precise relationship between the voltage and actuator displacement due to the creep phenomena, vibration, and hysteresis.^254^

Several advantages associated to dielectric elastomer actuators are their actuation under a DC applied voltage, their workability in dry conditions, allowing a long-term actuation. Further, they present a fast response time and a deformation maintenance under the application of a constant DC voltage. Additionally, they also presents a high density of mechanical energy and large operation forces comparatively to ionic EAP actuators. However, the deformation only occurs in one direction and high operation voltages of approximately 150 MV·m^–1^ are required to the deformation process, leading to the necessity of use special circuits.^252^

By comparing piezoelectric actuators with electromagnetic actuators, piezoelectric ones are the most promising developed microactuators due to their easy fabrication, compact size, fast response, low noise, and lightweight/cost-effectiveness enabling electromechanical devices.^255^ Further, the integration of multiple piezoelectric stages leads to the enhancement of the functionality of piezoelectric actuators and promotes the achievement of multiple degrees of freedom motion by stepping piezoelectric actuators or direct actuation from traditional actuators.^256^

This type of actuator has been applied in different fields, such as soft robotics, artificial muscles, and electronic devices,^255^ leading to a considerable market demand resulting in new piezoelectric materials and devices every year.^253^

The piezoelectric actuator configuration depends on the application, being the critical specifications related to the actuator’s force, displacement, and operating voltage. Other important factors that strongly affect the actuator performance are the material stiffness, capacitance (function of the excitation voltage frequency), and resonant frequency (frequency response to the maximum output amplitude actuator response). Specifically to piezoelectric actuators, the critical parameter is the force needed to promote the elongation of the device, usually in terms of N·μm^–1^.

Among all piezoelectric polymers, PVDF and its copolymers are the most widely exploited in developing piezoelectric actuators due to its lightweight, easy processability, high flexibility, and presence of a polar β-phase responsible for its piezoelectric properties, with the dipole aligned in the same direction. The combination of PVDF’s higher flexibility with a most significant amount of β-phase leads to the development of actuators without needing an additional postprocessing method.^254^ Poly(VDF-*co*-TrFE) copolymer developed a high strain of 4% compared to piezoelectric ceramics (∼0.2%).^253^

PVDF-based actuators have been applied in different fields, such as controlled displays, acoustic emission monitoring tools, artificial muscles, and robots.^8^ Ultrasonic PVDF-based actuators were developed for the first time in 1972, being the first electro-acoustic actuators commercialized in 1975.^8^

#### 3.2.2 Self-Sensing and Shape Memory Bending Actuators

The principle of self-sensing bending actuators is based on the applied voltage and current, the actuator deflection detected from the electrical driving voltage. Also, the high-frequency detection concerning the electrical impedance deformation-dependence on the actuator, including resistance and capacitance, enables a self-sensing actuator.^257^ The piezoelectric element can also be used as an actuator and sensor (time-sharing approach). This type of actuator is generally used on dynamic unloaded operations, allowing the control of a cantilever without load at the tip.^257^

**Figure 15:**
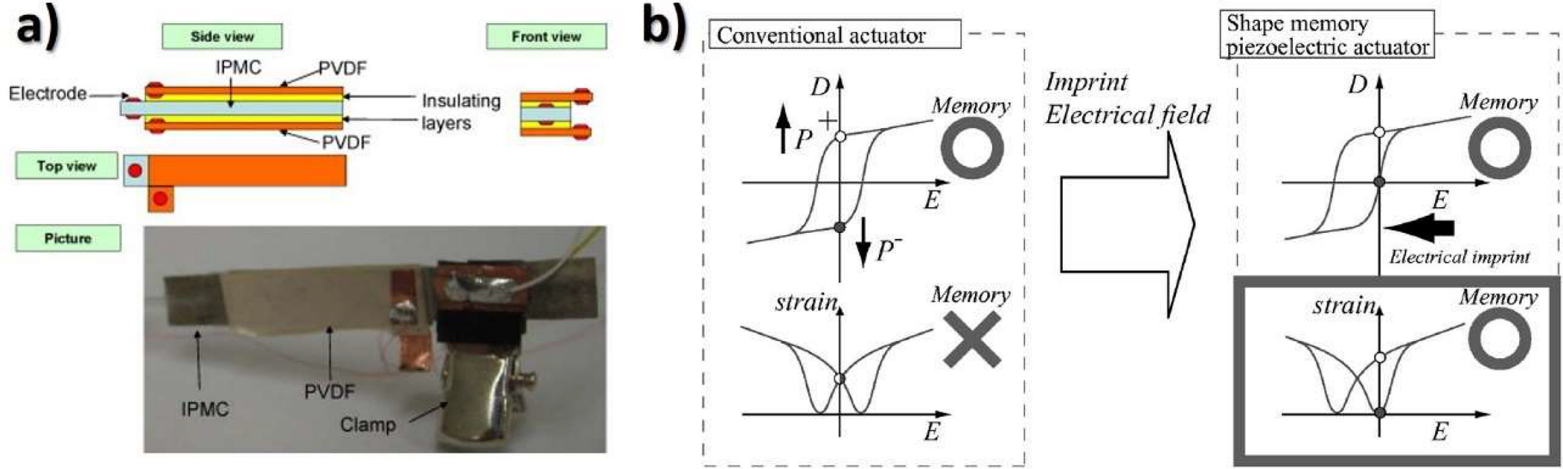
(a) Design of the IPMC/PVDF composite structure for sensing of bending output (force sensor not shown). Reproduced with permission from ref (258). Copyright 2008 Elsevier. (b) Principle of the piezoelectric memory effect by control of the imprint electrical field. Reproduced with permission from ref (259). Copyright 2007 American Institute of Physics Publishing.

PVDF has been explored for self-sensing actuators development. As an example, it has been used in the design of an integrated sensory actuator in which PVDF films were bonded to an ionic polymer–metal composite (IPMC), and a differential charge sensing circuit was used to provide feedback on displacement and force outputs of the IPMC actuator (Figure 15a).^258^ Differential configurations for sensing were used, eliminating sensing signals by thermal drift or feedthrough signal actuation. The developed system well-captured interaction forces as low as μN.^258^

PVDF/PZT composites with a significantly increase in the *d*_33_ value have been developed by extrusion casting and their electromechanical strain properties evaluated. An increase in the mechanical strain with the electrical field intensity occurs, reaching 0.8 μm at an applied electric field of 200 kV·mm^–1^. PZT/PVDF film reached a |*d*_33_| value of 35 pC·N^–1^ and an electromecanical actuation of 1.6% (higher than a piezoelectric ceramic).^260^

PVDF has been also combined with indium tin oxide (ITO) (ITO/PVDF) and with CNTs for self-sensing and microactuator applications in order to explore the induced displacement under applied voltage and varying frequency using a laser displacement sensor. The CNT/PVDF nanocomposites exhibited better performance as self-sensors and microactuators (∼0.1% under an applied voltage of 10 V at 0.5 Hz).^261^

Another interesting class of actuators relies on shape memory piezoelectric materials being the operation of this type of actuator based on the application of small pulsed voltages, leading to small consumption operation (Figure 15b).^259^ This type of actuator presents substantial advantages when compared with piezoelectric actuators, namely when applied as a mechanical relay switch, requiring the operation of a continuous voltage to maintain “on” and “off” conditions. Further, piezoelectric actuators require a large DC voltage to be applied for a stable, specific actuator position, implying the use of large electric amplifiers. Contrarily to piezoelectric actuators, shape piezoelectric actuators allow an actuator stable position, concerning the modes “on” and “off” of the actuator, without an applying electrical field, being the actuator stable in the two positions.^259^ The actuator polarization is reversed after an applied voltage. With the switch changing from “on” to “off” by applying a pulsed voltage, a reverse of the polarization occurs, the electrical source being able to be disconnected, leading to reduced energy consumption. It is noticeable that the actuator voltage shape can be conducted by a pulsed shape voltage generated by combining a small voltage source with transformers and capacitors. The charge accumulation to the capacitor allows the shape memory actuator operation as a pulsed voltage (through the transformer).^259^

As a piezoelectric polymer, PVDF has been used to develop shape memory piezoelectric actuators. To achieve a maximum bending of a cantilever actuator, a PVDF-based unimorph actuator was integrated with a shape memory polymer (poly(urethane) (PU) (Scotch tape-PVDF-SMP).^262^ A simulation study was performed being observed that the heat generated by the piezoelectric PVDF layer contributed to the total actuator deformation. Further, the generated heat can be used to increase the maximum bending of the actuator. The shape memory polymer layer length and mounting location influence the bending motion of the actuator, and the use of an equal layer of PVDF at the center of the actuator results in a bending angle increase to 40° when compared to the bending resulting from the piezo bending (4°) at 20 V.μm^–1^.^262^ Different cantilever PVDF-based actuators have also been developed, displaying the Scotch tape-PVDF-SMP layer-based actuator the highest bending performance, as summarized in Table 5.

**Table 5:** Comparison of Different PVDF-Based Bending Actuators^a^

materials active/passive	dimensions of PVDF layer (l × *w* × *t* in mm)	moment of inertia (m^4^)	max electric-field (V·μm^–1^)	tip deflection (mm)	bending curvature (1·m^–1^)
PVDF	2 × 0.5 × 0.02	3.33 × 10^–19^	15	0.1	
PVDF	60 × 20 × 0.16	6.83 × 10^–15^	3.75	0.3	
PVDF/terpolymer/Scotch tape	30 × 20 × 0.03	4.50 × 10^–17^	70		140
PVDF/terpolymer/Scotch tape	30 × 10 × 0.035	3.57 × 10^–17^	100		150
PVDF/Scotch tape/SMP	20 × 2.5 × 0.05	2.60 × 10^–17^	20	0.83	104

a Reproduced with permission from ref (262). Copyright 2021 Multidisciplinary Digital Publishing Institute.

PVDF films have also been combined with SMA to develop gripper actuators with force improvement performance and control of the position. Despite the shape memory actuator advantages (simple structure and high energy density), it is challenging to control the output force. The combination of both types of materials is of great interest for the tactile control of the force feedback of the driven gripper, allowing an accurate grasping control and a rapid response.^263^

**Figure 16:**
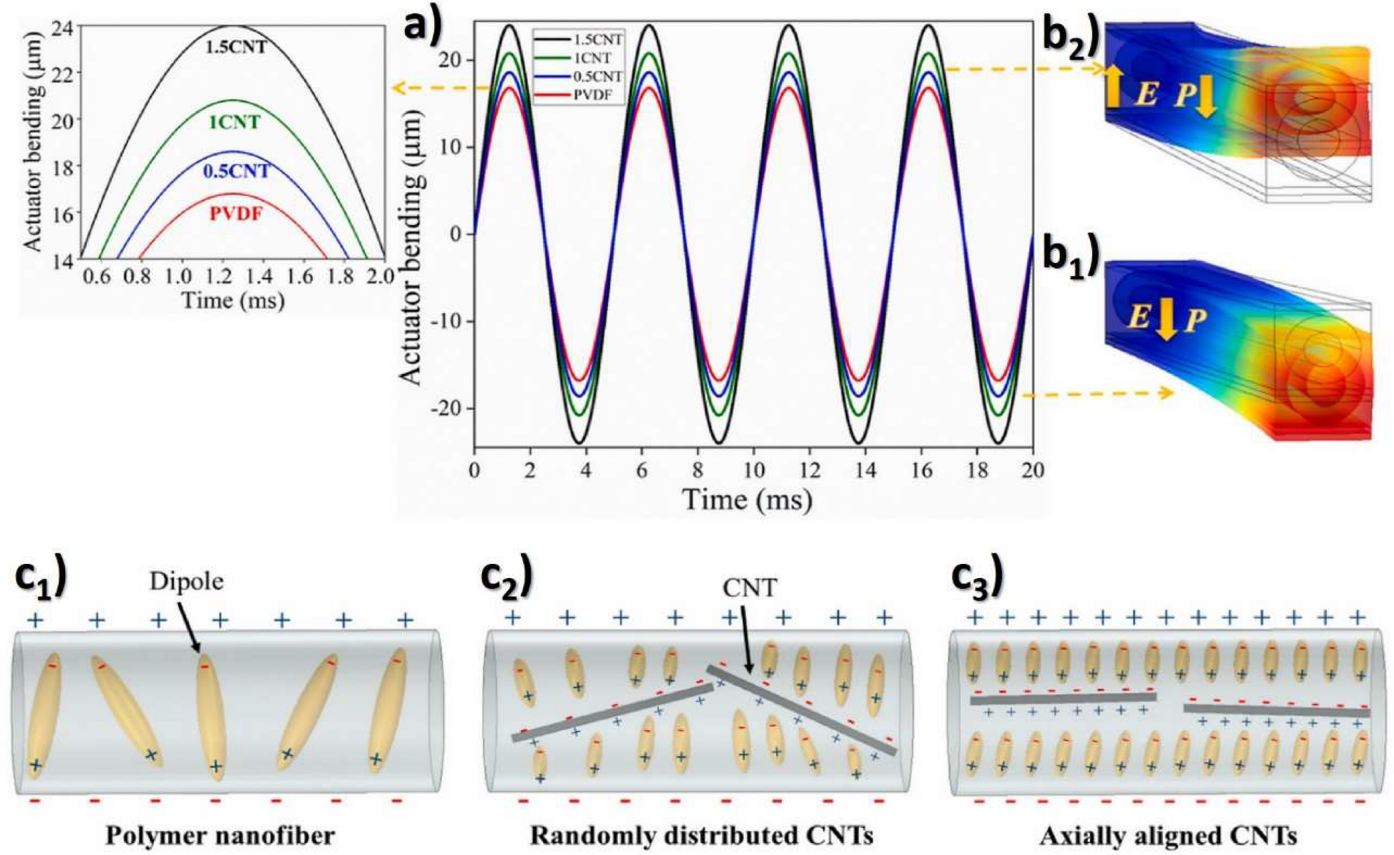
(a) Piezoelectric actuation of PVDF/MWCNT nanofibers under 4 V·μm^–1^. Schematic of the (b_1_) downward and (b_2_) upward bending of the actuators. Poling mechanism and dipole distribution diagram of the (c1) pristine PVDF nanofibers and PVDF nanofibers containing (c2) randomly distributed CNTs and (c3) axially aligned CNTs. Reproduced with permission from ref (255). Copyright 2021 Elsevier.

Electrospun PVDF fibers also present potential for actuation, increasing the actuation performance with the introduction of MWCNTs, reaching deformations up to 24 μm under an applied electric field of 4 V·μm^–1^ (Figure 16).^255^

Table 6 summarizes studies concerning the development of electrospun actuators based on PVDF nanofibers.

**Table 6:** Piezoelectric Actuators Based on PVDF Nanofibers^a^

			output/input	
piezoelectric actuator	dimensions	deformation	maximum deformation (*d*)	applied electric field (*E*)
PVDF/MWCNT	4 cm × 1.5 cm	bending	24 μm	4 V·μm^–1^
thin shell PVDF/CNT aligned hollow nanofibers)	4 cm × 1.5 cm	bending	18 μm	4 V·μm^–1^
fixed-fixed PVDF/MWCNT single microfiber	1 mm × 10 μm	center displacement	23 μm	1.5 kV·mm^–1^
fixed-fixed PVDF single microfiber	500 μm × 2.6 μm	center displacement	∼1.5 μm	1.2 V·μm^–1^
PVDF/MWCNT fibrous membranes	10 mm × 15 mm	center displacement	<1.5 μm	2 V·μm^–1^

a Reproduced with permission from ref (255). Copyright 2021 Elsevier.

#### 3.2.3 Ionic Electroactive Polymer-Based Actuators

With respect to ionic EAP actuators, significant efforts have been performed, especially based on the combination of PVDF with conducting polymers, CNTs or ILs commonly defined as salts entirely composed by cations and anions. The later are receiving increasing attention based on the large bending actuations at low driving voltages. ILs have unique characteristics such as high ionic conductivity and an excellent electrochemical and chemical stability. The incorporation of ILs within a fluorinated matrix allows tailoring the bending response of the actuator and moreover, the physicochemical properties and improve the electroactive response of PVDF.

#### 3.2.4 Electromechanical Ionic Actuation Mechanism

Electromechanical ionic actuators allow to convert the electrical signals into the development of a mechanical force, resulting in a displacement. In general, there are composed by a separator and two electrode layers.^264^ The principle of actuation mechanism of ionic EAP actuators is based on the transference of the applied energy by ions, with the ions movement occurring between two electrodes. The ions movement leads to an imbalance of ions distribution within a material, resulting in a pressure gradient that induces a mechanical deformation.^252^

The actuation displacement mechanism is controlled by the applied frequency and the applied current–voltage. The displacement results from an applied AC or DC voltage, and it is evaluated by the movement of the actuator using eq 3, attending to electrode area and the strain of the sample through the thickness of the actuator (*d*), the developed displacement along the *x* axes (δ) and the sample free length (*L*):^265^
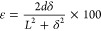
3

**Figure 17:**
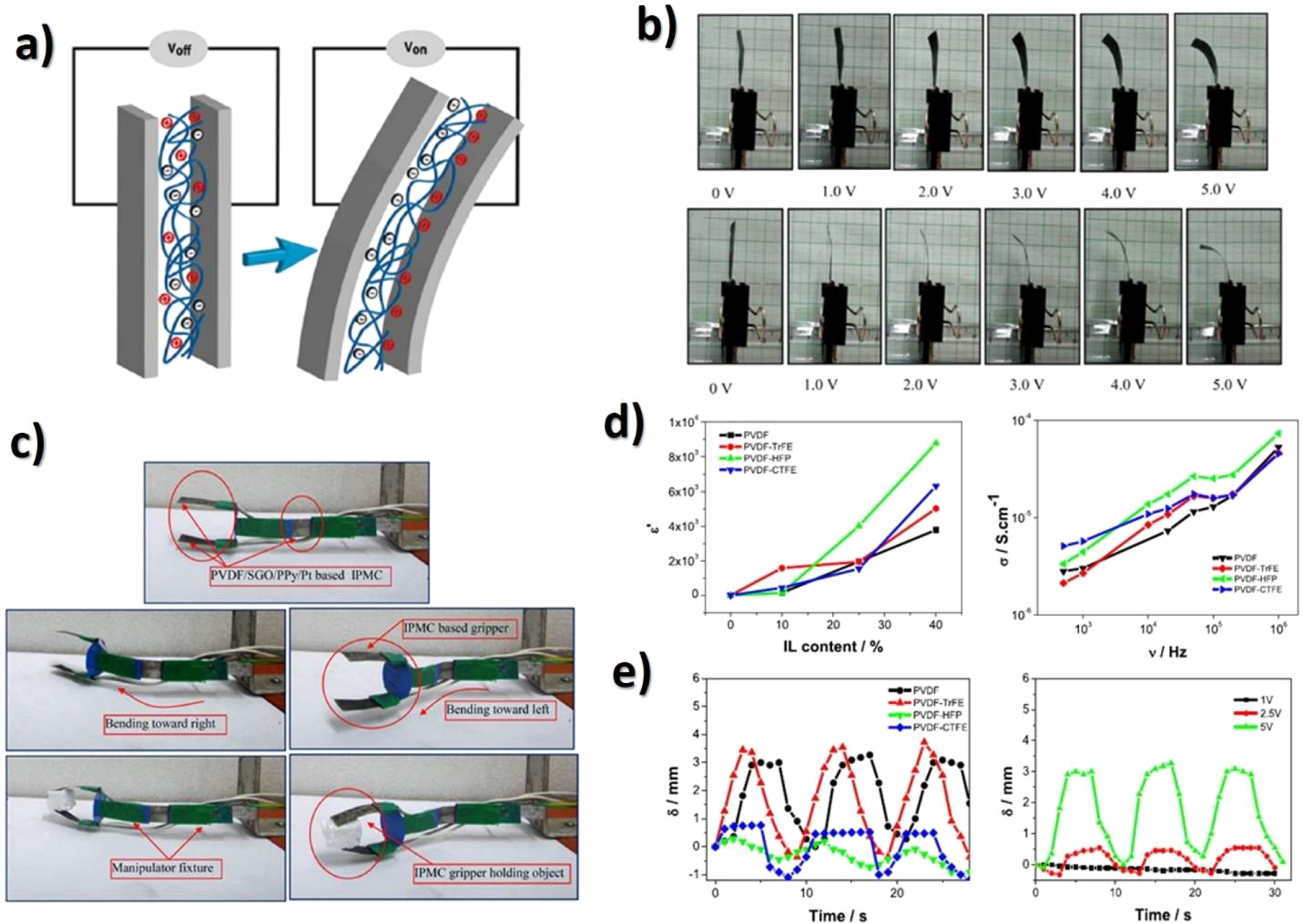
(a) Illustration of the ion migration and bending response of ILs/PVDF composites. Reproduced with permission from ref (265). Copyright 2019 American Chemical Society. (b) Successive deflection behavior of (a) PVDF/SGO/Pt and (b) PVDF/SGO/PPy/ionic polymer metal composite membrane actuators. Reproduced with permission from ref (269). Copyright 2019 Springer Nature. (c) Flexible link manipulator based on PVDF/SGO/PPy/Pt ionic polymer metal composite membrane actuator. Reproduced with permission from ref (269). Copyright 2019 Springer Nature. (d) Dielectric constant and AC conductivity for different polymer matrices with 40 wt % of ILs. Reproduced with permission from ref (266). Copyright 2019 Elsevier. (e) Displacement as a function of time at a frequency of 100 mHz for different polymer matrices with 40 wt % IL content at an applied potential difference of 5 Vpp and for IL/PVDF composites as a function of applied potential difference for a frequency of 100 mHz. Reproduced with permission from ref (266). Copyright 2019 Elsevier.

As briefly reported, in electronic EAP actuators, commonly based on piezoelectric polymers, DE, electrostrictive and ferroelectric polymers, and liquid crystal elastomers, the actuation mechanism is observed upon the application of high voltages in order to achieve large actuation strains.^247^ These limitations can be overcome by ionic EAPs, including IPMC, conductive polymers, and CNTs.^247^ In the last years, much attention have been paid in the development of ionic EAP actuators combining an ionic conductive filler, commonly ILs, and a polymer matrix, in which the actuation mechanism involves the ion’s diffusion and mobility into the polymer matrix.^265,266^ An ionic current is generated in the separator when an applied voltage is applied between the two electrode layers.^265,267^ The lowest potential barrier between the electrode and the separator layer promotes the ion migration of the charges of the positive ions (cations) and negative charges (anions) to the negative and positive side, respectively, close to the electrodes, as schematized in Figure 17a).^265^ Further, additional advantages of ionic EAP-based actuators rely on the low voltage operation, high flexibility, lightweight, and capability of working in bot dry and aqueous media.^268^

#### 3.2.5 Actuator Bending Performance

PVDF based materials combining conductive polymers have been developed to obtain IPMCs actuators. An ionic polymer metal composite membrane based on PVDF sulfonated GO composite membranes coated with Ppy and Pt metal with enhanced electromechanical properties has been developed.^269^ The observed displacement results from the applied voltage to the membrane electrodes, which produces an electric field that activates the actuator. Both PVDF/SG/Pt and PVDF/SGO/PPy/Pt IPMC membranes reach a maximum displacement of 10 and 14 mm, respectively, mainly due to the presence of the conductive polymer (Figure 17b).^269^

The IPMC actuators based on PVDF/SGO/PPy/Pt, demonstrated potential for robotic applications by handling small objects.^269^ The composite membrane PVDF/SGO/PPy/Pt actuator was used as the flexible joint, and other two IPMC joints were integrated at the end of flexible links (Figure 17c). The bidirectional bending of the link manipulator allowed the manipulation of objects, holding the object between two positions under an applied DC voltage of 5 V.^269^

Electromechanical actuators based on ILs have been emerging as an exciting approach in the last years. ILs have gained a particular interest in the actuators field due to their high ionic conductivity, electrochemical stability window between 4 and 6 V, and excellent chemical stability.^270,271^ Additionally, ILs are considered green, nonflammable, and nonvolatile solvents.^272^

Apart from the high ILs conductivity, the inclusion of these salts into a polymer matrix induces a material plasticizing effect, promoting a decrease in *T_g_*.^265,266,273^ The conjugation of those properties and the ability to tailor cations and anions size, type, and chain length, and ILs concentration into the polymer matrix allows the development of high conductive ionic EAP actuators with high performance.^265,267^ Incorporating high conductive ILs into different fluorinated polymer matrices results in the development of high ionic conductive matrixes with strong potential for actuators.

Different types of ILs have been incorporated within the PVDF and PVDF copolymers matrixes aiming the development of actuators with a high bending response at low applied voltages. The influence of PVDF and its copolymers poly(VDF-*co*-HFP), poly(VDF-*co*-TrFE), and poly(VDF-*co*-CTFE) in the actuator performance was evaluated by the incorporation of different contents of the IL 1-ethyl-3-methylimidazolium bis(trifluoromethanesulfonyl)imide ([Emim][TFSI]).^266^ Independently of the fluorinated matrix type, the IL incorporation promotes the β-phase nucleation, the decrease in the crystallinity degree, the Young modulus decrease and an increase in the electrical conductivity and dielectric constant (Figure 17d).

Among all fluorinated matrixes, PVDF/ILs and poly(VDF-*co*-TrFE)/ILs achieved the highest bending response (∼3 and 3.5 mm, respectively) for the maximum ILs content, as a result of higher polar β-phase contents which favors ionic mobility to allows switching bending direction. Thus, bending response and in this sense the actuator performance is influenced by the polymer chain free rotation. Bulk chemical groups in poly(VDF-*co*-CTFE) and ion–dipole interactions between the IL and poly(VDF-*co*-HFP), decrease the flexibility of the polymer chain, and hinders the ion mobility, leading to lower displacements (∼1.7 mm and 0.7 mm, respectively), the bending actuation being favored by the higher amorphous state of the IL/poly(VDF-*co*-CTFE) composite (Figure 17e).^266^

The bending performance of composites based on different ILs (*N*,*N*,*N*-trimethyl-*N*-(2-hydroxyethyl) ammonium bis(trifluoromethylsulfonyl)imide ([N_1112_(OH)][TFSI]) and 1-ethyl-3-methylimidazolium ethylsulfate ([Emim][C_2_SO_4_]) and PVDF were evaluated at different voltages (2, 5, and 10 V) at 10 mHz and with different ILs contents.^250^ The bending response is more dependent on the IL content, developing the [N_1112_(OH)][TFSI]/PVDF at 5 V the highest bending strain (10.5 mm). The nontoxicity of the [Emim][C_2_SO_4_]/PVDF composites demonstrated the suitability of these composites for biomedical applications.^250^

**Figure 18:**
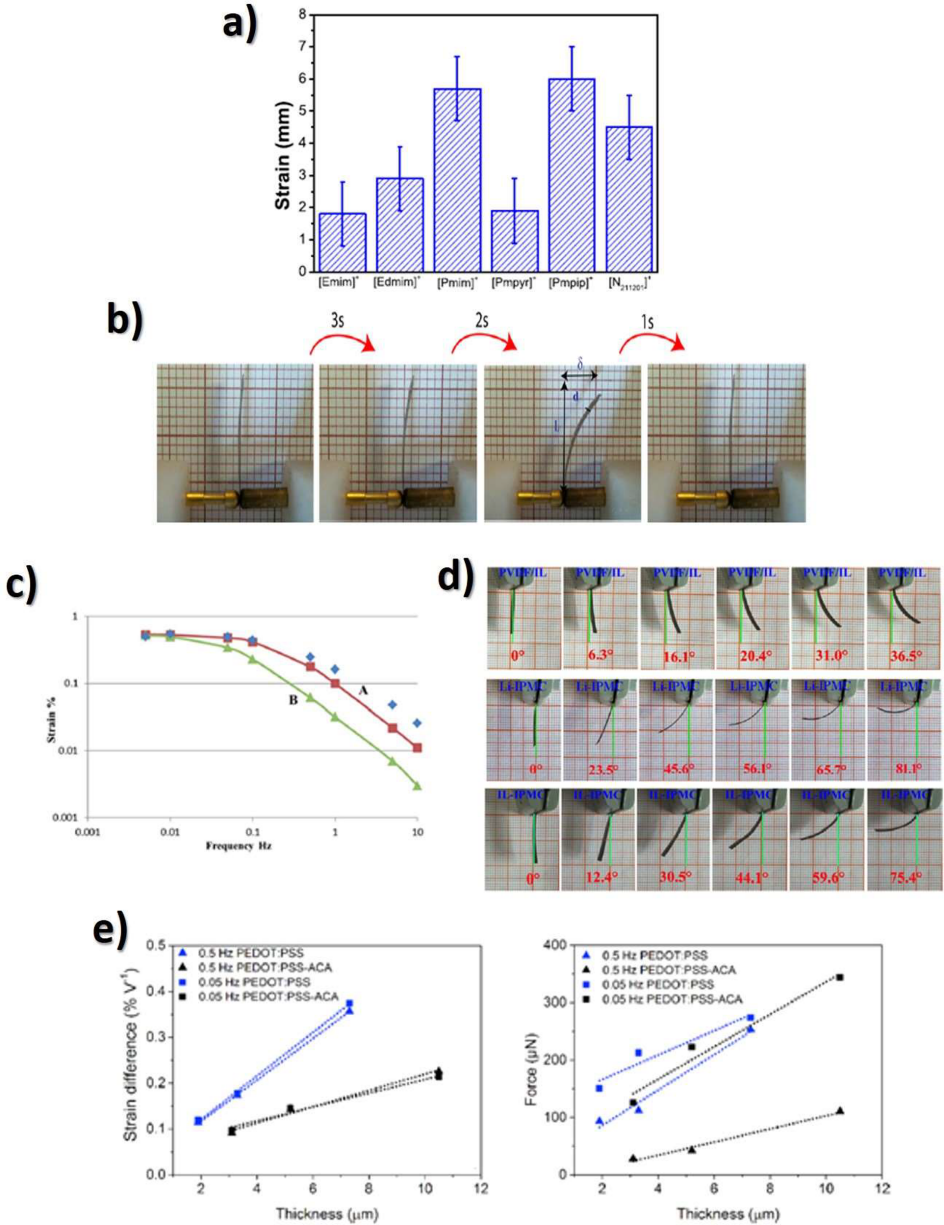
(a) Displacement of the composites for an applied voltage of 5 V and a frequency of 100 mHz and (b) schematic representation of the ion migration and bending response as a function of time for the [Pmim][TFSI]/PVDF composite. Reproduced with permission from ref (265). Copyright 2019 American Chemical Society. (c) Frequency dependence of the strain of the poly(VDF-*co*-HFP)–Nafion–SWCNT–EMIM[BF_4_] device with poly(VDF-*co*-HFP):Nafion ratio of 1:3. Reproduced with permission from ref (275). Copyright 2017 Royal Society of Chemistry. (d) Images of PVDF/IL-based IPMC (control, top), Li–IPMC (middle), and IL–IPMC (bottom) actuated by a sinusoidal electrical signal. Reproduced with permission from ref (276). Copyright 2019 American Chemical Society. (e) Bending displacement actuator driven by square wave potential signal and blocking force (exponential chirp waveform potential) dependency on the thickness of the electrode at 0.5 and 0.05 Hz in the potential range ± 1 V. Reproduced with permission from ref (277). Copyright 2018 Elsevier.

The bending performance is also cation/anion type and chain length dependent, increasing the bending strain with increasing cation size.^274^ The incorporation of different IL alkyl side cation chains of a variable family type (pyridinium, imidazolium, and ammonium ions) and chain length sharing the same anion [TFSI]^−^ revealed a decrease of the electrical conductivity with increasing cation alkyl chain size. Furthermore, larger cation size promotes a higher bending response as a result of the strong ion–dipole interactions between the IL and polymer, and a decrease of the Young modulus, increasing the ions movement within the polymer matrix and in this sense the bending actuation.^265^ The composites propylimidazolium ([Pmim])[TFSI]/PVDF and propylmethylpiperidinium ([Pmpip]) [TFSI] developed the highest bending strains (5.7 and 6.0 mm), respectively, at 5 V and 100 mHz (Figure 18a,b).^265^

PVDF incorporating ILs comprising different anion types, [TFSI]^−^ and chloride ([Cl]^−^)), and the same cation, hexyl-3-methylimidazolium ([C_6_mim]^+^),^267^ revealed a anion type and IL content influence in the actuator performance. A maximum bending response of 0.53% at an applied voltage of 10 V was obtained for the [C_6_mim][Cl]/PVDF composites containing 40 wt % of the IL.^267^

Other conductive fillers have been also used in combination with ILs to increase the actuator performance. The performance of ionic fluoropolymer (nafion) and nonionic poly(VDF-*co*-HFP)/IL gel hybrid actuators based on single-walled carbon nanotubes (SWCNT) (Nafion-poly(VDF-*co*-HFP)-IL-SWCNT gel) were compared with actuators based on poly(VDF-*co*-HFP)-IL-SWCNT gel electrolytes.^275^ Due to the high ionic conductivity of the Nafion-poly(VDF-*co*-HFP)-IL gel electrolyte, this actuator presents the highest strain, being approximately 1.6 and 1.5 times higher, respectively, than the corresponding values for the poly(VDF-*co*-HFP)-SWCNT-IL actuator (Figure 18c). The involved actuation mechanism results from the IL cations and anions movement.^275^ Other studies also report the incorporation of IĹs into the poly(VDF-*co*-HFP) as electrolyte actuators with potential in the development of wearable and energy-conversion devices.^278^

PVDF/poly(vinyl pyrrolidone) (PVP) IPMC actuator films with enhanced inner channels were developed using an IL, the 1-ethyl-3-methylimidazolium tetrafluoroborate ([Emim][BF_4_]), as a sacrificial porogen to promote either water- or IL-driven ion-exchange and by coating Gr/PVDF flexible electrodes on PVDF/PVP films.^276^ With an applied AC field, a continuous electromechanical response was achieved with maximum swing angles of ±36.5 and ± 75.4°, as shown in Figure 18d. These flexible IL-driven IPMCs actuators can find applications in the design of artificial muscles and displacement/vibration sensors.^276^

Nowadays, printing technologies have gained particular attention in the scientific field and, more recently, in developing electromechanical actuators. I. Põldsalu et al.^277^ developed actuators by printing electrodes based on PEDOT:PSS and PEDOT:PSS-carbon aerogel (ACA) with different thickness on both sides of IL 1-ethyl-3-methylimidazolium trifluoromethanesulfonate ([Emim][T_f_O])-saturated hydrophilic PVDF membranes to evaluate the thickness influence into the electromechanical actuation (Figure 18e).^277^ The deposition of 20 printed electrode layers results in a linear correlation between the electromechanical parameters: surface electrode resistance, electromechanical strain, and the blocking force of the actuator.^277^

The equivalent bending elastic modulus upon electromechanical actuation decreases with the addition of PEDOT:PSS (P5, P10, P20) printed layers to the electrode. A relation between the electrode layer thickness of the actuators and the strain to charge ratio revealed that for PEDOT:PSS actuators, the strain to charge ratio increases with increasing electrode layer thickness, decreasing the ratio with electrode layer thickness for DOT:PSS-ACA actuators.

**Figure 19:**
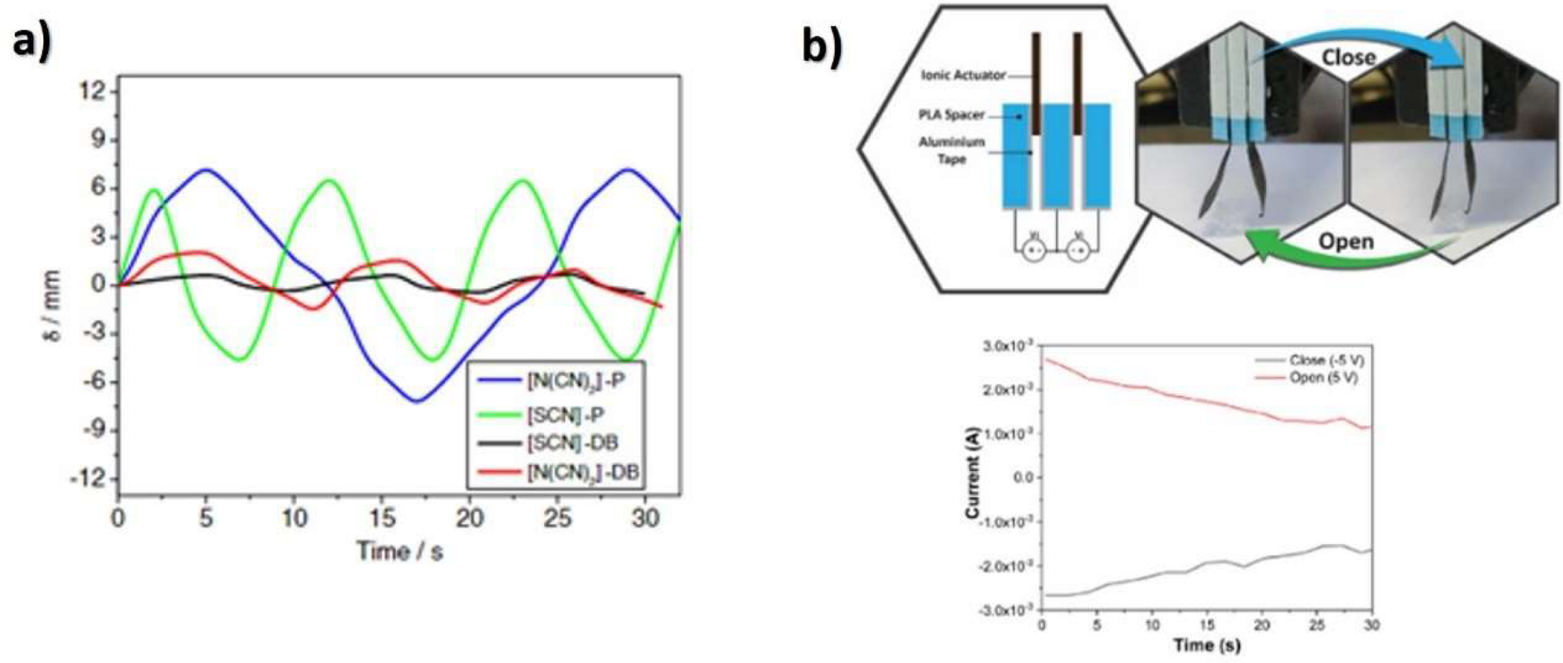
(a) Displacement as a function of time of the [Bmim][SCN]/PVDF and [Bmim][N(CN)_2_]/PVDF samples obtained by doctor blade and DIW under an applied voltage of 4 Vpp and a frequency of 0.1 Hz. Reproduced with permission from ref (279). Copyright 2021 Wiley-VCH. (b) [Bmim][N(CN_2_)]/PVDF micro gripper actuator and developed current as a function of time. Reproduced with permission from ref (280). Copyright 2021 Elsevier.

The efficiency of printed PVDF/IL electromechanical actuators obtained by direct ink printing comparatively to PVDF/IL films obtained by a solvent casting procedure was also studied through the development of printed and solvent casted PVDF-based materials incorporating the ILs 1-butyl-3-methylimidazolium dicyanamide [Bmim][N(CN)_2_] and 1-butyl-3-methylimidazolium thiocyanate [Bmim][SCN].^279^ The efficiency and suitability of the printing technologies instead of the solvent casting method for the development of high-performance soft actuators is demonstrated by [Bmim][N(CN)_2_]/PVDF high displacement (7.5 mm) for an applied voltage of 4 Vpp at a frequency of 0.1 Hz (Figure 19a).^279^

The implementation of PVDF/IL printable materials in soft robotics was demonstrated by developing a printed micro gripper.^280^ [Bmim][SCN]/PVDF developed the highest bending displacement (1 mm) at an applied voltage of 4 V in DC mode, and the PVDF/IL implementation as a micro gripper is shown in Figure 19b). Upon an applied voltage of −5 V to the [Bmim][N(CN_2_)]/PVDF film, a current of ∼−2.6 mA is generated as a result of ionic charge from IL movement close to the electrodes, resulting in a micro gripper closing. The current values decrease due to the lower sample mobility after a few seconds. After applying 5 V, the micro gripper moves to the open position.^280^

Table 7 summarizes PVDF-based polymer composites as advanced functional materials for electromechanical actuators. High-performing actuators have been achieved by varying the cation and anion type, cation chain length, and IL type. Further, the combination of different polymer matrixes with PVDF, such as PEDOT:PSS, among others, has also been explored.

**Table 7:** Summary of Representative PVDF-Based Polymer Composites for Ionic Electromechanical Actuators

polymer matrix	conductive filler	maximum strain (mm)	application	ref
PVDF/SG/Pt	PPy	10	soft robotics	(269)
PVDF/SGO/PPy/Pt		14		

PVDF	[Emim][TFSI]	3		(266)
Poly(VDF-*co*-TrFE)		3.5		
Poly(VDF-*co*-CTFE)		1.7		
Poly(VDF-co-HFP)		0.7		

PVDF	[N_1112_(OH)][TFSI]	10.5	biomedical	(250)
	[Emim][C_2_SO_4_]	1.7		

	[Emim][TFSI]	1.8		(265)
	[Edmim][TFSI]	2.9		
	[Pmim][TFSI]	5.7		
	[Pmpip][TFSI]	6.0		
	[Pmpyr][TFSI]	1.9		
	[N_1112_(OH)][TFSI]	4.5		

	[Emim][Cl]	0.42		(267)
	[C_6_mim][Cl]	2.5		
	[Emim][TFSI]	3.73		
	[C_10_mim][TFSI]	0.88		

	[C_6_mim][Cl]	4.4		(267)
	[C_6_mim][TFSI]	3.4		

PVDF	[Bmim][SCN]	7.0		(279)
	[Bmim][N(CN)_2_]	7.5		

PVDF	[Bmim][N(CN_2_)]	0.9	micro gripper	(280)
	[Bmim][C(CN_3_)]	0.6		
	[Bmim][SCN]	1.0		

PVDF	[Emim][BF_4_]	11.1	artificial muscles	(276)
PVDF/PVP	LiCl	12.6		

Besides the high interest on developing electromechanical actuators (electronic and ionic), several relevant issues are still to be addressed. Drawbacks associated with the actuator cycling stability, durability over time, and the generated actuator force must be overcome. Efforts must also be devoted to the development of printable actuators to improve device integration.

### 3.3 Energy Harvesting and Storage

Energy harvesting and storage are essential challenges to be addressed in the scope of the current energy and digital transitions to improve sustainability. The harvesting of unused and wasted energy from various sources at reasonable costs are continuously researched based on a variety of physical principles. Energy harvesting technologies such as piezoelectric, pyroelectric, or triboelectric nanogenerators, electromagnetic or thermoelectric devices, have been developed to harvest otherwise lost kinetic or thermal energy in the form of electricity to charge low-power devices or to store in batteries. Since 2006, polymer-based piezoelectric energy harvesting systems have been increasingly developed.^281^ Pyroelectric and triboelectric devices were proposed in 2012.^282^ Since then, several research groups and companies have been exploring this field, focusing on energy conversion efficiency and the implementation in practical applications. Among polymers, PVDF and its copolymers are the most investigated for the development of energy harvesting systems,^283^ due to their electroactive properties, mainly in the polar β-phase, with the largest piezoelectric coefficients and dieletric constant. The most used PVDF copolymers for energy harvesting is poly(VDF-*co*-TrFE), due to its improved piezoelectric and dieletric properties.^284^

Energy storage devices have been widely used to convert chemical into electrical energy, and include Li-ion batteries that have been commercialized since the 1990s. Battery main components are anode, cathode, and separator. Different materials and material combinations have been explored for these individual components of the battery to improve battery capacity and energy efficiency. In particular, polymer-based materials have been extensively researched for Li batteries using several filler types, geometry, and preparation techniques. PVDF-based composites processed by solvent casting and electrospinning techniques are among the most studied materials to improve separator battery performance. Electrodes are based on different active materials with large filler content and PVDF as a binder material. Compatibilization between fillers and binder and parameters optimization such processing temperature, or polymers microstructure, have been continuously improved. Polymer blends promote improved adhesion and mechanical characteristics to the electrodes.

SPEs are safer, environmental friendlier, but up to now show lower performance than conventional separator membranes for Li-ion batteries. Different studies focus on the development of specific polymers and filler combinations to improve ionic conductivity, ion diffusion, and electrolyte stability. PVDF-based polymer reinforced with Li-based materials are among the most used materials in composites development for high-performance SPEs materials.

#### 3.3.1 Energy Harvesting

Polymer-based materials are capable of generating energy using different intrinsic phenomena: piezoelectricity,^11,285,286^ pyroelectricity,^287−289^ triboelectricity,^290−292^ thermoelectricity,^293,294^ and electromagnetics.^295,296^ Although ceramics and single crystals can present enhanced electroactive properties, polymers have unique mechanical properties, including high tensile stress and low weight. With flexible polymers, such as PVDF and its copolymers, energy harvesters can be easily be implemented into a variety of systems, including wearables and self-power sensors.^295^ Thus, PVDF and copolymers and the respective composites are nowadays the most studied and employed polymers in energy harvesting systems development,^297−299^ using mechanical energy and thermal gradients from the surrounding environment.

In particular, polymer composites have been extensively explored to improve the performance of energy harvesting systems, mostly based on ceramics, single crystals, and conductive particles, as the most used fillers.^290,297,300^ PVDF microstructure has been also tailored for energy harvesting technologies, including in the form of homogeneous and porous films,^290^ nano- or microfibers, and two-dimensional (2D)/3D structures.^287^

In the following, the main PVDF based energy harvesting systems are presented.

##### 3.3.1.1 Mechanical Devices

Piezoelectric materials generate an electrical voltage when a mechanical stress is applied (direct effect). The direct effect is therefore intensively studied for energy harvesting, combining reinforcing fillers with high piezoelectric coefficients (*d*_31_ and *d*_33_) with ferroelectric polymers.

**Figure 20:**
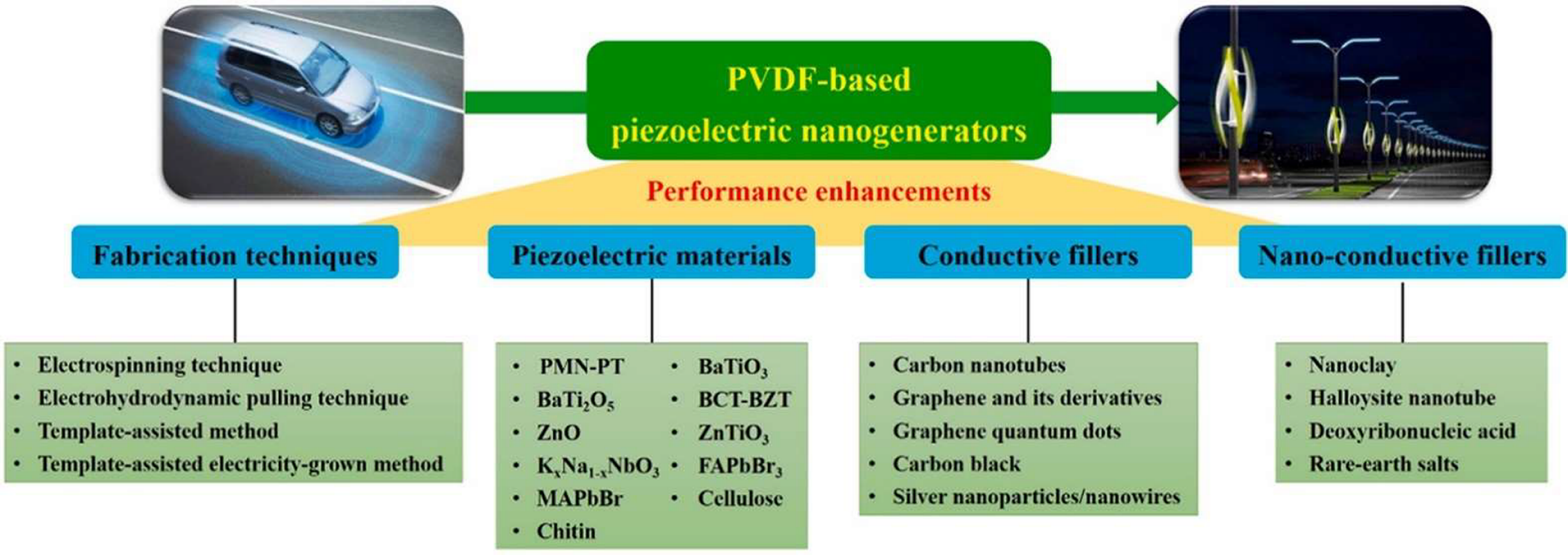
PVDF-based technologies for energy harvesting piezoelectric generators. Reproduced with permission from ref (305). Copyright 2019 Elsevier.

Fillers used to improve the energy harvesting properties of PVDF-based generators are typically ceramics or single crystals,^290,300,301^ as well as some carbonaceous^290^ materials, as shown in Figure 20. Ceramics are the most used materials due to the high dielectric and piezoelectric responses. PZT presents outstanding piezoelectric properties (*d*_33_ ∼304 pC·N^–1^), and it is most commonly used in commercial applications. Nevertheless, due to the toxicity of lead, a wide range of ceramics have been studied for lead-free applications, such as, BaTiO_3_, (*d*_33_ ∼150 pC·N^–1^) or ZnO (*d*_33_ ∼15–23 pC·N^–1^), among other ceramics materials with significant lower *d*_33_ coefficient.^297,301,302^

The piezoelectric harvested energy (*E*) of a material can be determined by eq 4:
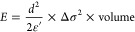
4where the *d* is the piezoelectric charge coefficient, ε′ is the dielectric constant, and σ is the stress.^303,304^

Piezoelectric harvesters based on PVDF have been developed by a variety of techniques, including solution casting, electrospinning, electrohydrodynamic, spin-coating, template-assisted method, and additive manufacturing, among others.^290,306,307^

**Figure 21:**
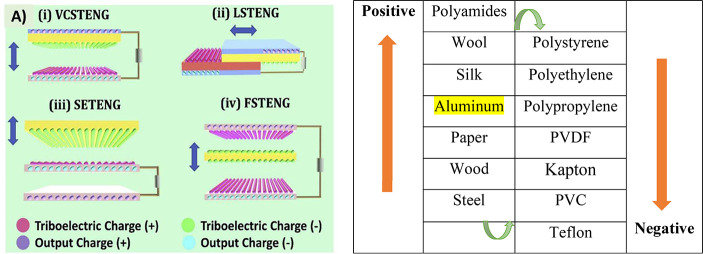
Schematic representation of the main working modes of triboelectric nanogenerators (NG): (i) vertical contact separation mode, (ii) lateral sliding mode, (iii) single electrode mode, and (iv) free-standing mode. Reproduced with permission from ref (311). Copyright 2019 Elsevier. Triboelectric series with some most common positive (losing electrons) and negative materials (gaining electrons). Adapted from refs (312 and 313). Copyright 2019 Springer and 2004 Elsevier.

Another mechano-electrical energy harvesting system, triboelectricity, relies on contact electrification between two distinct materials.^290,291^ Mechanical energy is converted into electrical energy, using four types of contact modes (Figure 21). Vertical contact-separation (VCSTENG) mode is the most used and the one with enhanced performance compared with sliding (LSTENG), single-electrode (SETENG), and free-standing modes.^308^ Placing in contact two different materials and due to their intrinsic surface properties, a potential is generated, known as electrification by contact.^290,291^ Van de Graaff generators are the most famous triboelectric devices using electrostatic electrical energy. Polarity, electron affinity, or surface potential are some parameters that influence the triboelectricity between two materials.^290,291,309^ In particular, the triboelectric effect depends on the polarity of the induced charge of the two distinct materials when subjected to frictional contact with one another, depending on the triboelectric series.^290,310^ Almost all metals and insulator pair materials show a relevant triboelectric effect, so there are a wide range of materials to be used in triboelectric devices.^310^

The triboelectric effect can be maximized using appropriate microfabrication technologies (lithography, etching, or deposition methods^309^), novel materials or by tailoring their properties (shape, structure, and surface roughness^309^). Triboelectric devices are governed by eq 5:^314^
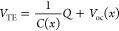
5where *V*_TE_ is the voltage generated between materials, *Q* and *C* are the transferred charges and capacitance between electrodes, and the *V*_oc_ is the open circuit voltage.

##### 3.3.1.2 Magnetoelectric Devices

**Figure 22:**
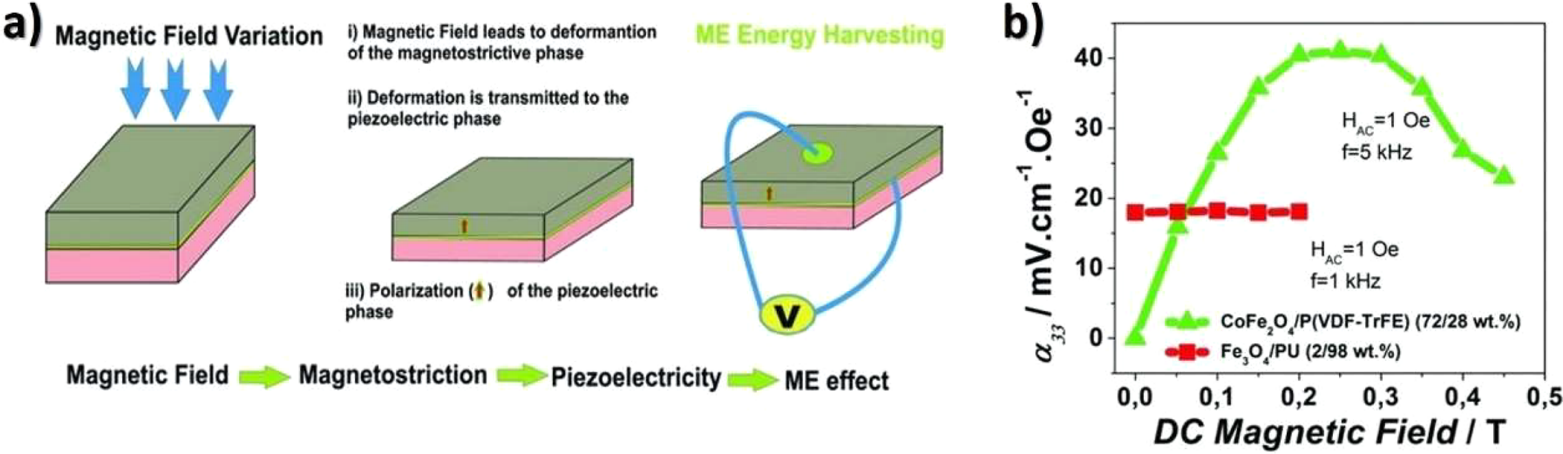
(a) Illustration of the magnetoelectric energy harvesting mechanism and (b) magnetoelectric coefficient. Reproduced with permission from ref (296). Copyright 2013 Wiley-VCH.

The magnetoelectric effect can be also used for energy harvesting based on the coupling of the magnetostrictive and piezoelectric phases of particulate or laminated composites.^315^ As illustrated in Figure 22, when the magnetic field induces a mechanical change in the magnetic materials due to magnetostriction, the mechanical variation is transduced to the piezoelectric material, leading to the development of a voltage due to the piezoelectric response of the PVDF-based materials. Thus, applying a magnetic field, the magnetic material suffers a strain that will generate an electrical voltage in the piezoelectric phase (magnetoelectric effect).^315^ The energy harvesting generated by these type of systems is very low, with some nano- to μW of power.^296,316^ For their lower power compared with other energy harvesting systems, magnetoelectric laminates and composites are more used as sensors.^296^ Commercial magnetostrictive materials typically used for this application are Vitrovac (Fe_39_Ni_39_Mo_4_Si_6_B_12_), Terfenol-D (Tb_0.3_Dy_0.7_Fe_1.9–2_), and Metglas (Fe_81_B_13.5_Si_3.5_C_2_), which are usually used with a piezoelectric polymer, most often PVDF and copolymers.^233,317^ The magnetoelectric effect is quantified by the magnetoelectric coefficient (*a*_ME_) described in eq 6:
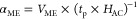
6where *V*_ME_ is the induced magnetoelectric voltage, *t*_p_ is the thickness of the piezoelectric material, and *H*_AC_ is the applied magnetic field.^317^

##### 3.3.1.3 Thermoelectric and Pyroelectric Devices

Thermoelectric materials are capable of converting heat into electrical energy, physical phenomenon known as the Seebeck effect (*S*), that depends on the temperature gradient *S* = Δ*V*/Δ*T*. This process involves charge and heat transport, the phonons and electrons being the main carriers. Their overall efficiency depends on the FOM (eq 7), which depends on the electrical conductivity, the Seebeck effect, and thermal conductivity.

Polymers doped with p-type and n-type nanofillers are used as thermoelectric materials.^318^ Thermoelectric devices are typically reinforced with one-dimensional (1D) metallic or carbon nanomaterials^318^ and semiconductor telluride-based materials, such as bismuth (Bi_2_Te_3_), animonium (Sb_2_Te_3_), and lead telluride (PbTe).^319^ Also, conductive polymers forming n-type or p-type materials are used, typically based on modified coordination polymers.^318,320^

**Figure 23:**
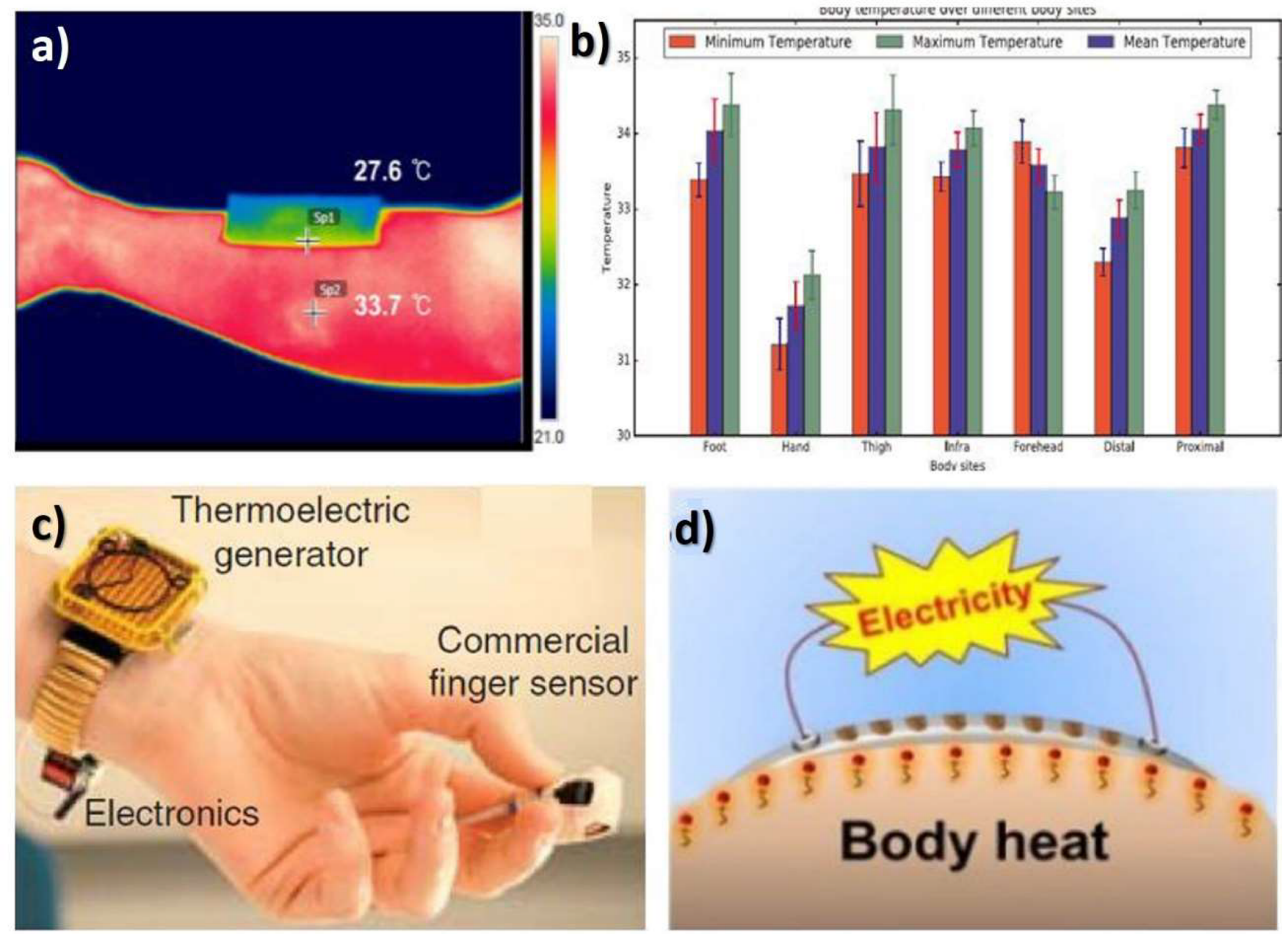
Temperature difference between human arm and the surface of the thermoelectric energy conversion device at room temperature (a,c,d) with average temperature for different body zones (b). Reproduced with permission from ref (294). Copyright 2019 Elsevier.

The FOM of the thermoelectric materials is represented by *T*, known as the power factor of the device, described in eq 7:^293^
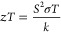
7where *S* is the Seebeck effect, σ and *k* are the electrical and thermal conductivity, respectively, and *T* is the absolute temperature. The power factor (PF) of the materials is defined as PF = *S*^2^σ.^321^ Ceramics and conductive polymers are the most commonly used materials for thermoelectric generation due to their thermoelectric properties,^293,318^ but PVDF-based materials can also present interesting thermoelectric properties.^318,322^ Conductive polymers with different post-treatments, different solvents, or organic solutions of inorganic salts, allow improvement of the *T* values, leading to power factors larger than 300 μW·m^–1^·K^–2^,^318^ much higher than pristine conductive polymers, showing 5 to 6 orders of magnitude lower.^318^ The goal of thermoelectric materials is to reach *T* of ∼2,^321^ which can be approached with nanostructured materials. Tailored nanomaterials with high-performance thermoelectric output possess power factor values between 0.8 and 2.5 W m^–1^·K^–1^.^321^ Human body energy harvesting is the most common application in literature (Figure 23).^294^

Pyroelectric materials can also generate electrical energy when subjected to temperature variations due to their intrinsic spontaneous polarization. Pyroelectric noncentrosymmetric polar crystals present a coupling between electrical polarization and temperature, implying that a change in temperature causes a change in the electrical dipole moment. Pyroelectric devices based on PVDF have been investigated.^288^ Pyroelectric material converts heat into electrical energy and pyroelectric output is optimized by a material with high pyroelectric and thermal coefficients, low electric and dielectric properties, and low specific heat.^288^ PVDF, copolymers and composites reinforced with ceramics^288^ including PZT, BaTiO_3_, and related ceramics have been used for pyroelectric device development based on the higher pyroelectric coefficient (γ) compared to polymers, having the polymers improved mechanical properties for device applications.^288^ The pyroelectric coefficient of polymers and polymer composites is typically lower than 1 C·m^–2^·K^–1^, whereas for ceramics the values range from 3 to 18 C·m^–2^·K^–1^.^288^ Thus the development of suitable polymer/ceramic composites is still the main way to optimize the overall performance of this type of energy harvesting devices.

The voltage and current generated by a pyroelectric energy harvesting device (*V*_PyE_and *i*_PyE_, respectively) can be determined by eq 8 and 9:^323^
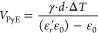
8
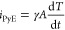
9where *d* and *A* are thickness and electrodes area, Δ*T* is the temperature change, d*T*/d*t* is the temperature variation rate, and the *ε*_r_^′^ and *ε*_0_ are the permittivity of the material and vacuum, respectively.^323^

**Figure 24:**
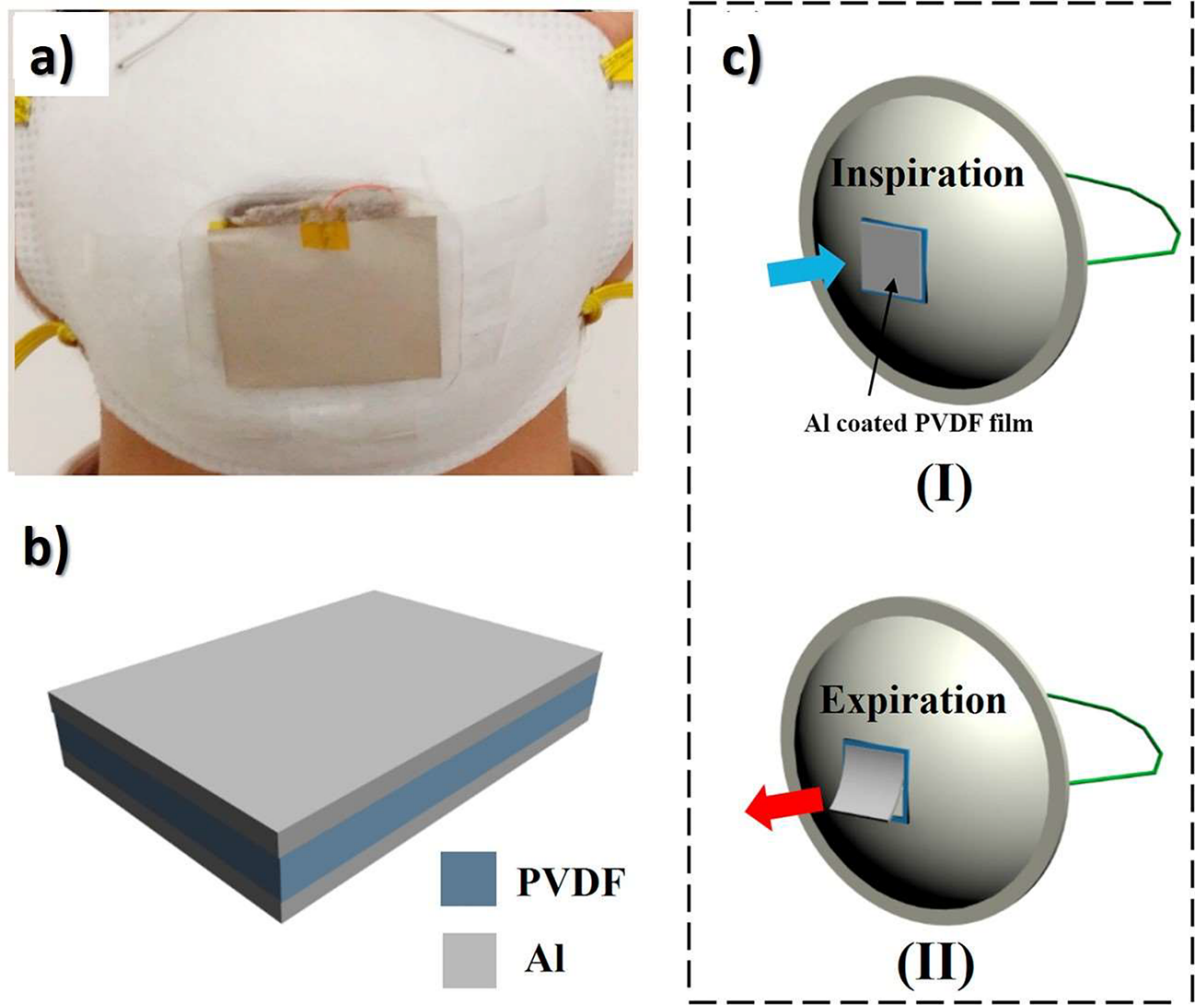
(a) Representation of the piezoelectric device and (b) pyroelectric PVDF film with Al electrodes. (c) Schematic representation of a pyroelectric driven by human respiration: (I) Inspiration, (II) Expiration. Reproduced with permission from ref (324). Copyright 2017 Elsevier.

Pyroelectricity can be generated from any heat gradient source, increasing for large and rapid temperature changing sources such as human breath, as represented in Figure 24.^324^

The most relevant PVDF based energy harvesting systems are summarized in Table 8, classified after the energy harvesting principle and the indication of the conversion FOM.

**Table 8:**
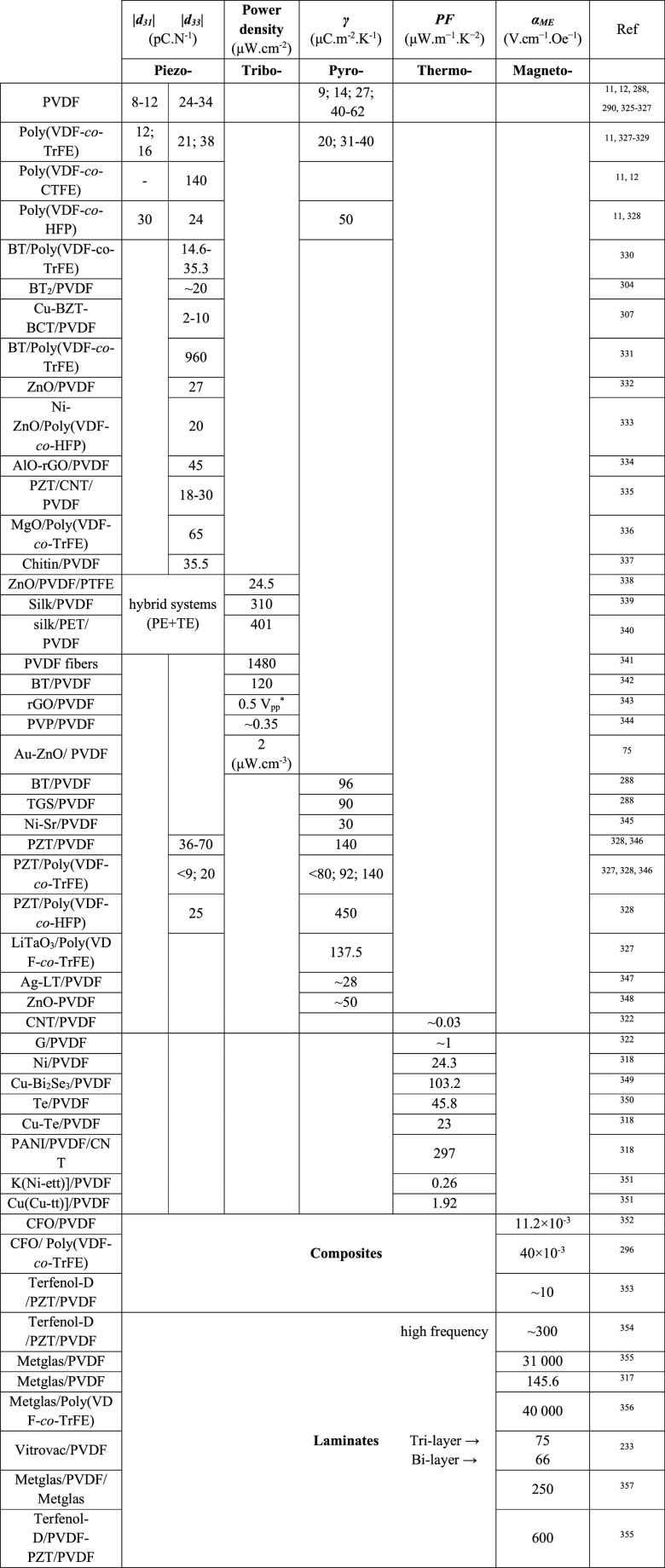
Functional Characteristics of the Most Representative PVDF Based Energy Harvesting Systems, Classified after the Main Conversion Mechanism and FOM^11,12,75,233,288,296,304,307,317,318,322,325,326,327−357^

Table 8 shows that the output power/energy of PVDF-based energy harvesting generators is enhanced with the reinforcement of the host matrix with ceramic, crystalline, carbon nano- or microparticles.^333,358,359^ The applications of PVDF-based energy harvesters is focused on low-power consumption devices, self-powered sensors or actuators and biomedical systems.^309^ Piezoelectric materials can generate power density from some μW per unit area in PVDF composites^304,330,360^ to some mW per area^307,335,337,348,361,362^ or even 16 W·m^–2^ in roadway application.^363^ Roadway applications generate higher output voltages due to larger impact/forces, depending on vehicles speed and weight, increasing the energy generated with both parameters.^363^ Further, the generated energy is larger in bending than in pressure modes.^364^ The energy generated critically depends on the harvester geometry, processing, stimulus, and efficiency, among other parameters such as the optimization of the electronic circuit of the harvester device.^365^ Composite materials with high-dieletric ceramics allow improvement of the generated output energy.^366^

Triboelectrics can generates up to near 1.5 mW·cm^–2^ in optimized PVDF fibers. Morphology, effective area and surface potential are critical parameters to improve harvesting performance, together with the opposite charges of the electrodes.^367^ Pyroelectric output power density can reach some μW·cm^–2^.^324,368^ As example, the pyroelectric solar radiation energy harvesting can generate 1 μW·cm^–2^.^369^ With respect to flexible thermoelectric generator, the output power can be improved by increasing the number of p–n junction pairs.^318^ In turn, magnetoelectric devices can generate a few μW of power in prototype systems.^317,370^

The different energy harvesting phenomena can be also combined into a single device, maximizing the output power given by a unique external stimulus applied to the device.^309,371^ Piezoelectric–triboelectric is the most studied combination typically based on PVDF as piezoelectric material and, at the same time, as one of the triboelectric elements. Typically, the piezoelectric devices generate a larger output current and the triboelectric generate larger output voltages, and their combination maximizes the generated power.^309,338,371^ Additionally, using transparent materials, it is possible to combine solar energy harvesters with piezo- and pyroelectric energy harvesters.^371^ PVDF-based material combined with a flexible solar cell fully charges a Li-ion battery of 1.5 V in a few hours.^371^ The electromagnetic–triboelectric effect is another hybrid effect that can generate up to 500 mW·m^–2^.^309^

In summary, piezoelectric energy harvesting is the most used phenomena for powering low-power energy devices, as it is the most simple to implement, is able to harvest ubiquitous mechanical energy in a simple configuration and can typically generate a power of μW, reaching to mW or even larger power outputs in specific applications such as road harvesting systems.^363^

##### 3.3.1.4 Applicability Considerations for Poly(vinylidene fluoride)-Based Energy Harvesting Systems

Piezoelectric materials, in particular PVDF and its copolymers, are widely studied polymer materials to be integrated as a generator element, given their high piezoelectric response and being flexible materials with high resistance to force application and deformation, which can generate energy based on different physical phenomena, or a combination of them.

**Figure 25:**
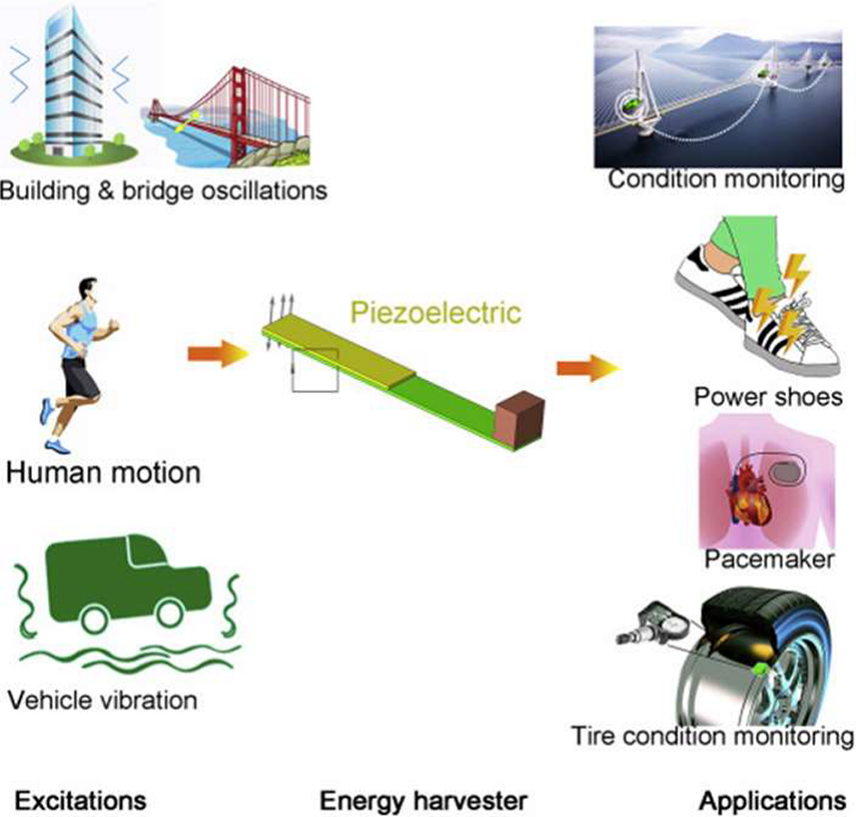
Some of the typical energy sources, from human movements to environmental stimuli, used for energy harvesting processes. Reproduced with permission from ref (303). Copyright 2018 Elsevier.

Due to strong research in this area, the energy generated by piezo- and triboelectric NG and triboelectric NG has been increased from the order of μW to the order of several mW or higher^363,372,373^ using distinct mechanical environmental or human stimuli, as shown in Figure 25, mainly in oscillation/bending and force/pressure stimulus.

The maximization of the energetic transduction efficiency (from mechanical, thermal, or magnetic to electrical energy) is based on optimizing the intrinsic properties of PVDF, copolymers, and composites, as well as on-device building factors such as resonance frequency, structural configuration, and resonance tension.^374^ Based on the adjustment of these parameters, transduction between the generator and the power source can be optimized, which reduces system losses. Depending on the application, the frequency and acceleration may present different values, hence there is a need to adjust the generator to a power source or to ensure that the generator has the broadest possible response range, for systems where these values are quite dynamic, as shown in Table 9. It is noticeable that frequency critically influences the energy per unit of time, whereas acceleration influences the energy per stimulus.

**Table 9:** Peak Frequency and Acceleration for Various Energy Sources^a^

source	frequency (Hz)	acceleration (m·s^–1^)
human walking	2–3	2–3
car engine compartment	200	12
door closing	125	3
induction motor	10–300	500
diesel motor	10–10^3^	500
industrial break	10–100	0–100
washing and drying machine	121	3.5

a Adapted from refs (375 and 376). Copyright 2018 Springer and 2019 IEEE.

At an early stage in the development of piezoelectric NG solutions, they were typically based on the traditional single element cantilever format,^376^ although new architectures have been developed that allow the optimization of the generated energy, such as stacked cantilevers, circular diaphragms, cymbal configurations,^377^ and capillary format,^378^ among others.

Based on advanced materials manufacturing processes, including the processing of nanofibers and nanospheres, it is possible to obtain an increase in energy efficiency, where it is already possible to verify responses in the order of 2.6 mW·cm^–2^ of power density.^378^Table 10 shows a summary of the generated power according to the stimuli, to the materials morphology and to the harvesting method.

**Table 10:** Compilation of Harvested Power Output of Representative NGs Based on PVDF

material	stimulus	type	method	power density	ref
PVDF	piezo	film	bending pressure	1.7 mW·cm^–2^	(363)
PVDF	piezo	film	bending pressure	0.9 mW·cm^–2^	(364)
PVDF + activated carbon	piezo	film	surface pressure variation	6.3 mW·cm^–2^	(378)
poly(VDF-*co*-TrFE)	piezo	electrospun webs	cantilever	5.9 mW·cm^–3^	(379)
PVDF + ZnO	piezo	porous film	surface pressure variation	0.17 mW·cm^–3^	(380)
poly(VDF-*co*-HFP)-TEA-BF_4_	piezo	yarn	cantilever	43 μW·h·cm^–2^	(381)
PVDF + GO-AlO	piezo	composite	cantilever	27.97 μW·cm^–3^	(334)
ZnSnO_3_-poly(VDF-*co*-HFP)/Al	tribo	fibers	vertical-contact	0.09 mW·cm^–2^	(382)
PVDF-Gn/AL	tribo	film	vertical-contact	2.6 mW·cm^–2^	(383)
PVDF/AL	tribo	film	vertical contact	0.26 mW·cm^–2^	(384)
PVDF	pyro	film	surface temperature variation	1.08 W·cm^–3^	(368)
PVDF	pyro	film	surface temperature variation	0.67 μW·cm^–2^	(324)
PVDF	pyro	film	surface temperature variation	2.4 μW·cm^–2^	(385)
SWCNT/PVDF	thermo	composite fibers	terminals temperature variation	0.38 mW·m^–1^K^–2^	(386)
PVDF/Al	thermo	film	surfaces differential temperature	88 μW·m^–1^K^–2^	(323)
MWCNT/PVDF	thermo	film	terminals temperature variation	58 μW·K^–1^	(322)
(Fe_64_Co_17_Si_6.6_B_12.4_)/PVDF	magneto	multifilm laminated	magnetic variation	1.5 mW·cm^–3^	(357)
Metglas/PVDF/Metglas	magneto	multifilm laminated	magnetic variation	0.9 mW·cm^–3^	(357)

Piezo- and triboelectric based energy harvesters are the most common NGs in the literature and, more important, those that generate more power output on the order of few W·m^–2^. Besides, both stimuli can be combined in a contact-separation or bending modes, enhancing the energy generated.

With respect to PVDF-based triboelectric NGs, several approaches have been adopted to improve performance, such as work function, dielectric constant, surface resistivity, and carrier density, among others.^387^

The performance of a triboelectric harvester is strongly dependent on material selection, based on the experimental “triboelectric series” tables,^388^ preferably being selected materials pairs from the opposite ends of the table, such as the case of polyamides, wool, or Al for positive tribopolarity materials and the case of Teflon, polyvinyl chloride, polyimide, or PVDF for negative tribopolarity materials.^389^ There are two main representative operating modes in the triboelectric NGs, vertical separation and side sliding contacts.^387^ Instantaneous power density is reported on the order of tens of mW·cm^–2^,^387^ similar to enhanced piezoelectric NGs. However, a good energy transduction efficiency has not been demonstrated, a large input energy in the system being necessary when compared with the output energy, hence the weak or nonexistent current applicability. Similar to piezoelectric NGs, to increase transduction efficiency, the materials manufacturing process has been optimized, increasing the surface area with the insertion of nanopores or nonsurface structures, as well as a surface coatings based on PVDF nanofibers,^373,390^ where power outputs in the order of 10 mW·cm^–2^ have been obtained.^373,390^ Thus, morphology and surface properties are critical for improving output performance of the triboelectric NGs and piezoelectric NGs.

Pyroelectric generators have been researched as a promising technology for IoT applications, where polymer-based solutions are the focus of study.^391,392^ Despite the several approaches, high-performance systems are related to generators based on pristine PVDF in a film topology, although micropatterned structures, nanowire structures, and fiber structures have also been reported, as shown in Table 10. Despite the exciting results in the order of 1.1 W·cm^–3^^368^ generated power densities, its applicability is conditioned by the need to guarantee a long cycle of thermal stability and solar radiation, as well as to guarantee that the energy collection substrate or electrode maintains the mechanical properties under heat and sun irradiation, and/or there is not induced damage by the light.

Thermoelectric generators have some similarities when compared to pyroelectric generators, but their method of operation is based on the collection of electrical energy from the residual heat by the Seebeck effect, so it is necessary to implement bimaterial solutions (similar to the triboelectric systems), that is, p-type and n-type thermoelectric materials. In this way, organic thermoelectric materials have attracted attention due to possible applications in flexible thermoelectric generators processed by solution, where it is possible to obtain bimaterial fibers capable of generating power on the order of 0.38 mW·m^–1^·K^–2^, as shown in Table 10. Although the output performance is lower when compared to other solutions, their processing method is opening unexplored fields of application. On the other hand, its applicability is also very dependent on ensuring a long cycle of thermal stability and solar radiation, as well as ensuring that the substrates, electrodes, or energy collection fibers maintain their mechanical properties under heat.

The magnetoelectric effect provides an innovative energy generation solution. Although the energy sources are not abundant, it may allow the supply of energy to inaccessible or remote zones, as it is possible to generate/transmit noncontact energy into devices by exposure to AC magnetic fields.

With the fast evolution of organic materials, the magnetoelectric coefficients obtained are in the same order of magnitude as the best obtained in inorganic magnetoelectric materials. Despite the great potential of these solutions, only a few studies have been dedicated to capturing energy from magnetoelectric materials based on polymers, as shown in Table 10, where power outputs on the order of 1.5 mW·cm^–3^ have been reported. The evolution of these devices in the IoT area has been stagnating based on the complexity of the developed systems and the specific needs of the required excitation fields.

On the other hand, this solution presents promising results in the biological area as it allows supplying energy in a controlled way in difficult to access otherwise biological environments.

##### 3.3.1.5 Electronic Circuits for Energy Harvesting Systems

**Figure 26:**
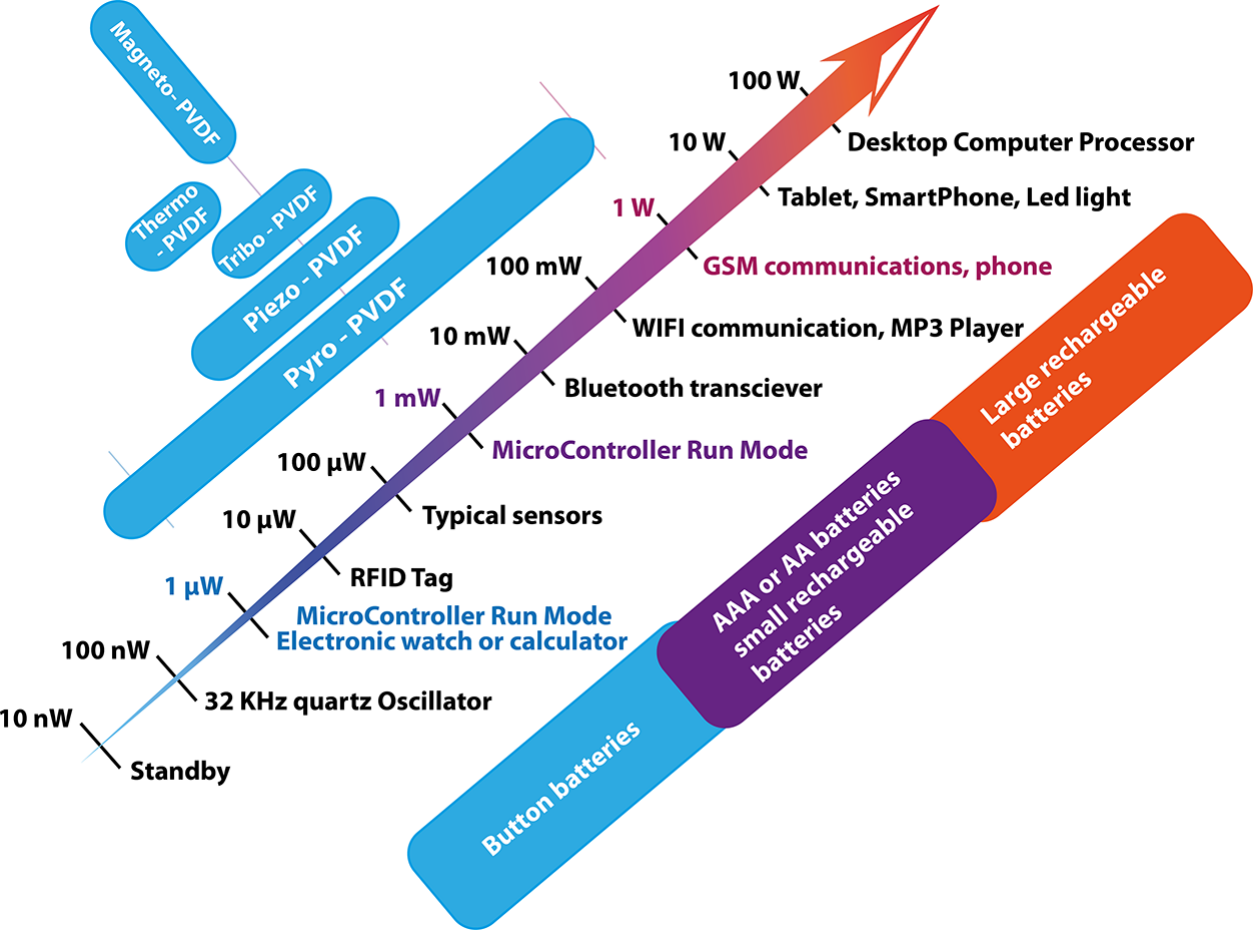
Energy generation systems and corresponding generated power and power consumption requirement for different devices. Image based on ref (395).

The fast development of CMOS technology and printing electronic circuits, coupled with the high-level integration capacity,^393,394^ is allowing reduction of the power consumption of the devices and the size of the systems, as shown in Figure 26.

Considering the emergence of hybrid solutions such as photonic and CMOS technology^396^ that allows high communication rates between subsystems, new highly efficient devices are being developed, with nA to μA consumptions (depending on microcontroller system execution state), faster and highly miniaturized.^396^

As represented in Figure 26, to respond to the energetic needs of IoT, it is necessary to incorporate energy generators capable of supplying up to some mW for systems operating in real-time, depending on the communication parameters.

On the other hand, systems in standby/sleep mode with wake up by low power timer, are increasingly optimized, allowing implementation of energy harvesting generators for lower power systems, including energy storage, sensors, and/or communication of information.

All of these combined developments have led to a reduction in the energy requirements of sensing, microactuation, and communication devices, and these correspond to basic solutions for the implementation of IoT systems in the areas of medicine, defense, interactivity, aeronautics, smart industry, and smart agriculture.

**Figure 27:**
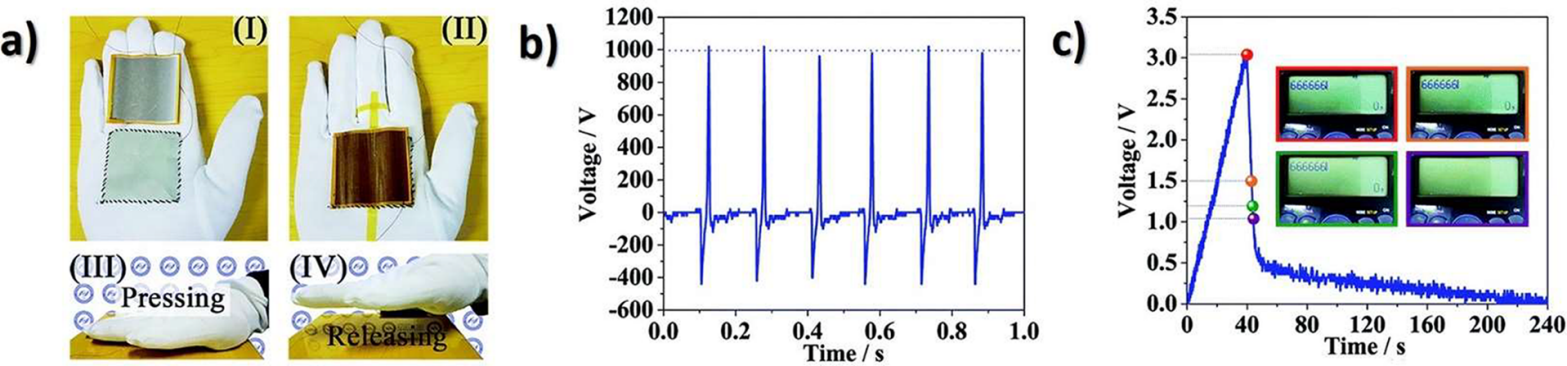
Demonstration of the heart-like micronanofiber (HMN-TENG) to harvest various biomechanical energies from the human body. (a) Photographs of (I) sewing of the HMN-TENG on a piece of cloth of a cotton glove, (II) fabrication of the HMN-TENG on the top surface, (III) pressing state, and (IV) releasing state during hand patting at a frequency of 6 Hz. (b) The output voltage and (c) charge and discharge curves of the HMN-TENG detected under the conditions described in (a). Reproduced with permission from ref (341). Copyright 2019 Royal Society of Chemistry.

Currently, it is possible to find functional examples of application prototypes of complete energy harvesting systems. In Zhang et al.,^341^ a system is presented that allows feeding a monochrome LCD solely with the movement of the palm or foot, based on the output power of 14.8 W·m^–2^, as shown in Figure 27.

The application optimized the generated voltage and current, in order of 1 kV and 150 μA (Figure 27b,c), presenting excellent harvesting performance when coupled to the load circuit.

In the biomedical area, there are also some examples for the application of these energy harvesting solutions, in particular for implantable medical devices.

Despite these examples of application, the power supplies of these devices remain a major challenge for the scientific community and the industry itself, making numerous applications impracticable, considering the minimum voltage value required for the rectification circuit (typically superior to 0.5 V) and the high minimum power for charging/storage circuit (typically >3 μW) as observed in available commercial applications, considering the low currents produced.

A device without batteries, which draws energy directly from its surroundings, is a promising way to provide continuous and inexhaustible power. The power source may be solar radiation, thermal gradient, and mechanical motion. Much research focuses on mechanical movement, especially vibration, as vibration is widely available in objects and the environment.

Over the past decade, there has been an exponential growth of triboelectric NGs and piezoelectric NGs architectures. Still, one of the biggest challenges for the true technological revolution in the area of self-power sensors and self-power devices is the energy storage circuit and its ability to acquire power from the generator/transductor. Currently, six main circuit topologies are the focus of investigation, as shown in Table 11.

**Table 11:**
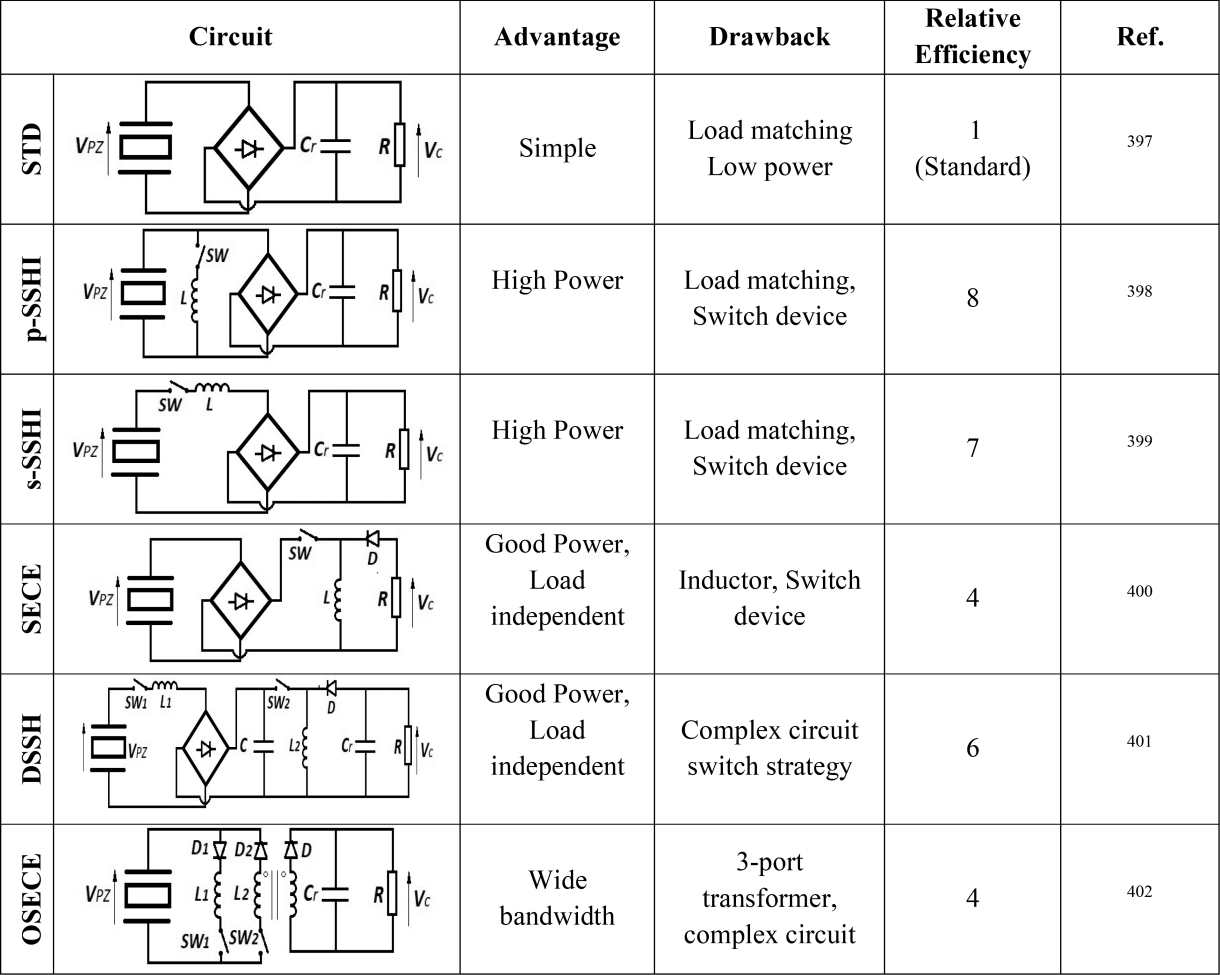
Working Principle of the More Representative Energy Harvesting Circuits^397−402^^402^^a^

a STD, standard technique DC mode; p-SSHI, parallel synchronized switch harvesting on inductor; s-SSHI, parallel synchronized switch harvesting on inductor; SECE, synchronous electric charge extraction; DSSH, double synchronized switch harvesting; and OSECE, optimized synchronous electric charge extraction.

However, it is important to note that despite the good results already achieved in laboratory conditions; when these systems are subjected to a real application environment, the generated voltages and currents are reduced, which makes coupling of the electronic harvesting circuit very difficult. Given the minimum operating conditions of the electronic components, this is a major challenge in the widespread implementation of this technology.

In conclusion, triboelectric NGs and piezoelectric NGs can be used as standalone and portable power sources for low power electronic devices such as sensors, low power, communication, and micro- or nanoactuation systems, with particular focus on implantable sensors and actuators, accuracy agriculture, self-predictive monitoring in industrial, medical, and automotive/aeronautic maintenance systems. Pyro-, thermo-, and magnetoelectric systems must be continuously developed, as the power generation is still insufficient for these types of devices. On the other hand, given the multifunctional characteristics of the materials used, such as the ones of PVDF, they can have a dual function and can be also used as motion, acceleration, or voltage sensors, among others. The increase in output power and the technological advancement of electronics will improve the applicability of these solutions, allowing meeting of the great challenges of the IoT and industry 4.0 era in a near future.

#### 3.3.2 Energy Storage Systems

Energy production and storage are particularly relevant topics in recent years, mainly due to the increasing energy demand from population, new lifestyles with increased mobility, and the need of an energy transition toward cleaner energy production. Once clean energy production is mainly based on intermittent energy resources, it is essential to associate energy storage systems capable of storing the generated energy to be used whenever required.^403^ According to this, energy storage systems, such as Li-ion batteries, the best and most used electrochemical energy storage system nowadays, electrochemical capacitors (supercapacitors), and electrostatic capacitors represent a research, development, and application priority. The major storage capability difference between batteries and capacitors is the higher energy storage capacity per unit weight from the battery systems and higher power capacity from capacitors. Also, battery systems are more applied to long-time operation, whereas capacitors are more suitable to provide high power in a short-time period. The increased society mobility, the ubiquitous use of portable electronic devices and, more recently, the implementation on electric vehicles, exponentially increase the demand of these systems.^404^

**Figure 28:**
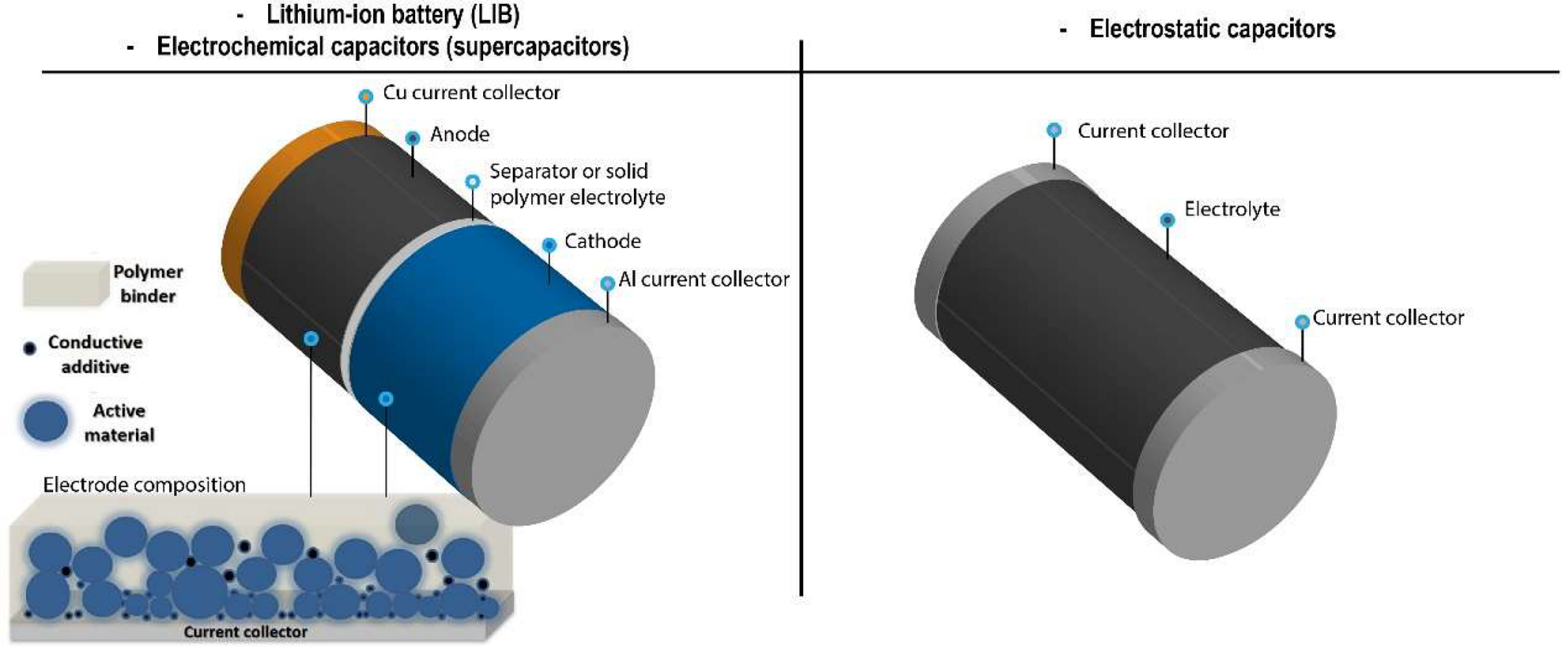
Schematic representation of different energy storage systems and its main components: Li-ion batteries, electrochemical capacitors, and electrostatic capacitors.

Figure 28 shows a schematic representation of the main energy storage systems components for Li-ion batteries, electrochemical capacitors, and electrostatic capacitors. PVDF based materials are widely used at these systems in different components, including cathode, anode, separator/SPEs for Li-ion batteries and electrochemical capacitors, and in the capacity layer for electrostatic capacitors.

Typically, in the Li-ion batteries and electrochemical capacitors, electrodes are composed by a polymer binder, a conductive additive, and an active material, which differs depending on whether the electrode is the anode or the cathode (Figure 28). PVDF is the most used polymer binder that holds together the active material and the conductive additive of the electrode, improves the mechanical stability/flexibility of the electrode, and ensure the good cohesion between particles and current collector.^405^ The main characteristics of PVDF as a polymer binder are the easy processability, high adhesion to the current collector, high voltage stability window at 5 V, degradation temperature above 400 °C, excellent mechanical properties, and good compatibility with the most used electrolyte solution, among others.^406^ Despite being used in a small percentage (0–15 wt %) with respect to the electrode overall composition, the polymer binder represents a relevant contribution in the battery performance, stability and electrochemical behavior.^407^

The separator/electrolyte structure can be categorized mainly in liquid and solid electrolytes (polymeric, inorganic, and composite). In conventional batteries the separator is typically a porous membrane soaked with electrolyte solution (liquid electrolyte) or a SPEs in solid-state batteries, typically based on embedded fillers dispersed in the polymer matrix in the latter case. It should be an electronic insulator and ionic conductor, with the main function promoting a medium for ions transfer, determining the cell kinetics between the electrodes in the charging, and discharging mechanisms.^408^

In addition, PVDF and copolymers are also used for battery separator/electrolyte applications due to their processability by a wide range of techniques from solvent casting to electrospinning, allowing tailoring of porosity/pore size and degree of crystallinity, by the dimensional stability with temperature variation, excellent mechanical properties, and high voltage stability.

In this context, the main advances in PVDF and copolymers as polymer binders for electrodes and polymer for separator/SPEs in Li-ion batteries/electrochemical capacitors applications and a capacitive layer in electrostatic capacitors are described in the following.

##### 3.3.2.1 Electrodes

As mentioned before, PVDF is widely used as polymer binder in electrodes composite for battery and capacitor applications due to its properties of binding the active and conductive materials, essential for the electrical, mechanical and thermal stability of the systems as well as for the ionic conduction process in the electrodes.^409^

Electrode components composition affects battery performance in distinct aspects. Different active material influences the battery capacity due to its theoretical discharge/charge capacity. Also, different ratios of active material, carbon black as conductive material, and PVDF binder on the cathode electrode has been evaluated, showing high performance when obtained with 90% active material and a carbon black/PVDF binder ratio of 0.8.^410^ In fact, it has been shown that the rate capability of the electrode is improved for a PVDF/conductive material ratio of 5:4.^411^

PVDF have also been studied in electrochemical capacitors as polymer binder, and the performance of PVDF has been compared with other polymers as PTFE and Nafion for active carbon active material electrodes. Higher specific capacitance value (160.6 F·g^–1^) was obtained for PVDF polymer binder with only 5 wt % compared to the PTFE and Nafion that require 10 wt % in the electrode slurry, to obtain similar response (156.6 and 131.3 F·g^–1^, respectively). Despite the 100% rate capability at 20 mV·s^–1^ from PTFE, PVDF retain a capacitance for about 79.7% after 2000 cycles with only 5 wt % of material.^412^

An important electrode property evaluated during its manufacturing is the rheological behavior of the electrode slurry. In this property, PVDF content have a great influence, where rheological measurements demonstrate that increasing its amount mainly increases matrix viscosity in the suspension without affecting the microstructure formed by active and conductive materials particles.^413^ Tuning this property opens the possibility to apply the electrode slurry in different manufacturing and additive manufacturing processes as doctor blade, screen-printing, DIW, and others, improving the battery quality and performance.^414^

Furthermore, the mechanical failure in the electrodes is dependent on the polymer binder where PVDF polymer plays an essential role in successfully addressing this issue, based on its excellent particle/binder^415^ and binder/current collector^416^ interfaces. Further, PVDF is essential for controlling the solid electrolyte interface (SEI) thickness.^417^

The role of PVDF binder in Si/graphite composite anodes for Li-ion batteries has been analyzed, showing that PVDF binder decomposes to form lithium fluoride (LiF) on the electrode surface during cycling, without affecting battery performance due to the good chemical interaction with both graphite and silicon.^418^

**Figure 29:**
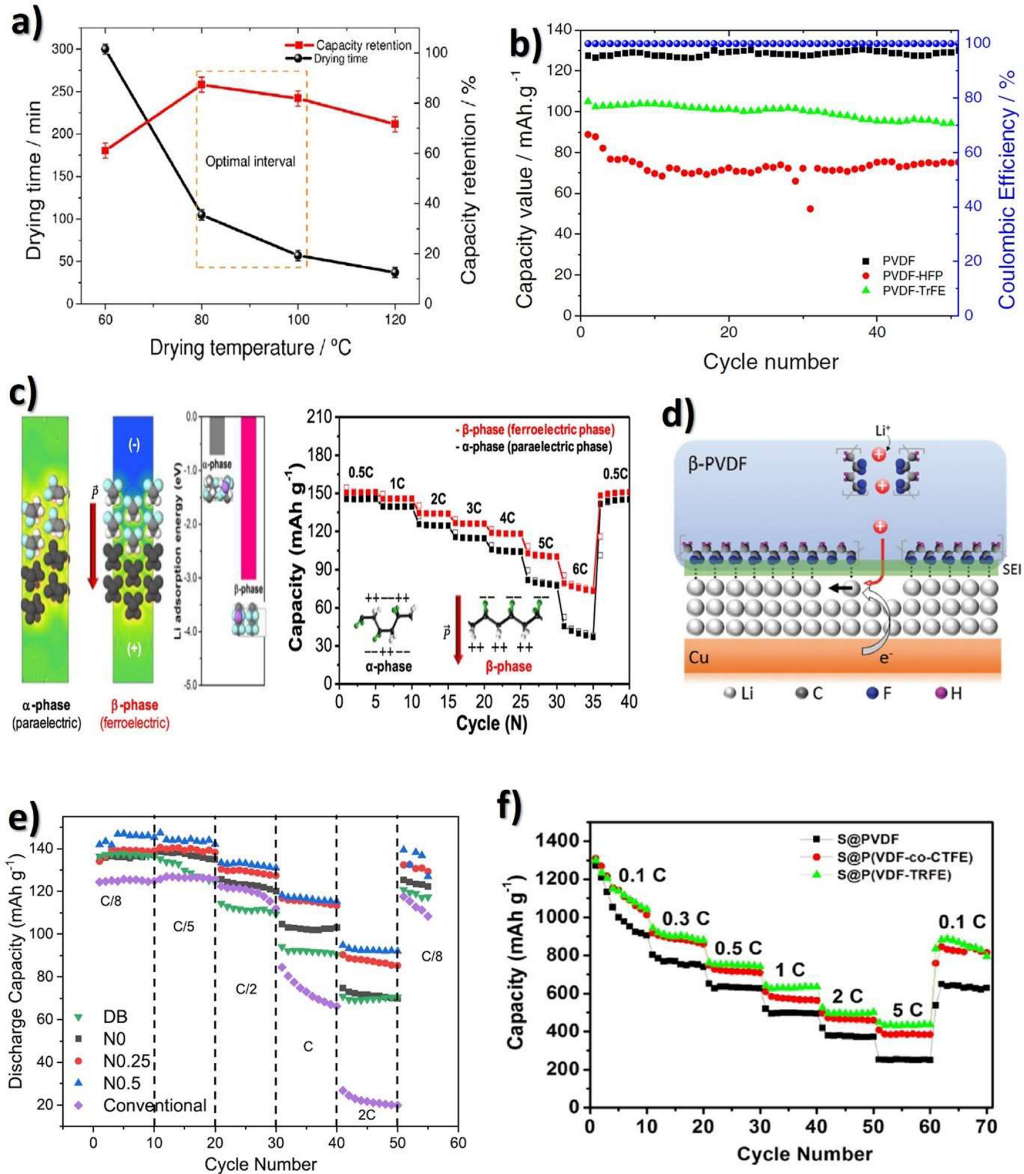
(a) Drying time and capacity retention as a function of the cathode drying temperature. Reproduced with permission from ref (419). Copyright 2016 Wiley-VCH. (b) Cycling performance and Coulombic efficiency at different C-rate of cathode films with different polymer binders. Reproduced with permission from ref (421). Copyright 2016 Elsevier. (c) Adsorption energy of Li-ions on F-terminated α- and β-PVDF surfaces and rate performance with PVDF binders in these two phases. Reproduced with permission from ref (434). Copyright 2017 Elsevier. (d) Schematic representation of the effect of the β-phase of PVDF in the diffusion pathways for Li-ions. Reproduced with permission from ref (435). Copyright 2018 Wiley-VCH. (e) Rate capability of printed cathode samples with poly(VDF-*ter*-TrFE-*ter*-CFE) binder. Reproduced with permission from ref (99). Copyright 2021 American Chemical Society. (f) Rate capability of sulfur S@PVDF, S@Poly(VDF-*co*-CTFE), and S@Poly(VDF-*co*-TrFE) electrodes. Reproduced with permission from ref (440). Copyright 2016 Elsevier.

Another important parameter that influences the electrode properties is the specific solvent used and the solvent evaporation rate to dissolve the PVDF binder.^419,420^ The optimal solvent evaporation temperature has been determined between 80 and 100 °C, as this process strongly influences the polymer binder distribution and the polar phase content of the polymer (Figure 29a).^419^ Also, for electrochemical capacitors, electrodes with oxygen-functionalized few-layer Gr as active material, PVDF as polymer binder and NMP as solvent, the solvent evaporation temperature that promotes the higher maximum specific capacitance (318 F·g^–1^, at 0.5 A·g^–1^) was at 170 °C when compared with 100 and 190 °C.^420^

Different fluoropolymer binders have been evaluated for Li-ion batteries, as shown in Figure 29b, mainly based on the different polarity of the materials. It was demonstrated that the polarity of the fluoropolymers, determined by the chain structure, number of fluorine atoms, and molecular weight, significantly affects cathode performance.^421^

Combining excellent battery performance and environmental concerns, a new aqueous PVDF latex binder was produced, demonstrating good cycling stability.^422^ Approaches including the combination of different polymer binders and addition of particles to improve electrode quality and performance have also been studied.^409^

To improve the PVDF binder performance, PVDF blends based on polyethylene-*block*-poly(ethylene glycol) (PE-PEG) copolymer and poly(propylene carbonates) (PPCs) were developed in order to improve structural uniformity, the addition of the second polymer reduces PVDF crystallinity and improves distribution of the conductive fillers, leading to better delivered capacity at different C-rates of the electrodes compared to neat PVDF binder at temperatures below 60 °C.^423^

Further, PVDF blends with styrene butadiene rubber (SBR) has been developed to improve the mechanical stability of Li metal.^424^ PVDF-grafted-BaTiO_3_ nanocomposites^425^ and PVDF blends with PEO^426^ and terpene resin (TX)^427^ were also applied as a polymer binder in Li-ion batteries to improve the fillers dispersion, adhesion to the current collector, and, consequently, overall battery performance.

PVDF polymer binder has been aslo implemented to improve active material’s electrochemical capacitors performance in MnO_2_,^428,429^ NiS,^430^ and MXene^431^ based electrodes.

Another issue that can be addressed through proper selection of the polymer binder is the heat generation during battery operation. Thus, thermally sensitive binders (TSB) based on PVDF and poly(VDF-*co*-HFP) have been developed, allowing reduction of the peak temperature associated with the internal short circuit without affecting battery performance.^432^ Furthermore, the nail penetration can be reduced by 20% to 40% by using the aforementioned binders, attributed to the softening of TSB at ∼80 °C.^433^

Considering its active role as a binder, the effect of the different crystalline phases of PVDF for high-rate Li-ions diffusion was determined, observing that the highly polar β-phase facilitates the diffusion of Li-ions and maintains the concentrations of Li-ions at the surface of the active electrode, resulting in much-improved capacity with lower cell resistance compared to α-phase PVDF as binder, as represented in Figure 29c.^434^ Further, the effect of β-PVDF as a promising artificial solid-electrolyte interphase coating on Cu and Li metal anodes was evaluated. Figure 29d shows a schematic representation of this effect, that leads to dendrite-free Li deposition/stripping and improved cycling performance.^435^ In the same way, PVDF polymer binder also has an active influence on the ionic transport pathways after percolation threshold, where the excess of binder in the electrode formulation increases the pathways and create an ion-blocking effect.^436^

With respect to printed Li-ion batteries, the polymer binder has an important role on electrode slurry due to its influence on rheological behavior allowing to tune this property to a desired printing technique. A new cathode ink was developed for screen-printing with suitable rheological and electrochemical properties.^437^ Taking into account the environmental issues for this battery, the conventionally used solvent for PVDF, NMP, was replaced by the “green solvent” DMPU, allowing the production of more environmentally friendly batteries.^69^

Furthermore, the substitution of NMP by DMF has been shown to benefit printed batteries at an industrial level by reducing cell manufacturing energy consumption by ∼30%.^438^

Considering the toxicity and cost from NMP solvent, efforts are being devoted to substitute it by DMSO solvent in Li-ion battery and electrochemical capacitors applications, once the latter is safer and eco-friendlier, although an additional washing step with ethanol is necessary.^439^

Poly(VDF-*ter*-TrFE-*ter*-CFE) *ter*-polymer has been also used as a polymer binder for cathode printed electrodes by DIW, showing suitable rate battery performance, as shown in Figure 29e.^99^ In fact, different PVDF copolymers have been also evaluated for Li-sulfur battery development (Figure 29f),^440^ poly(VDF-*co*-TrFE) showing improved performance with respect to PVDF and poly(VDF-*co*-CTFE) due to the strong chemical interaction with polysulfides based on its high molecular polarity.^440^

The influence of PVDF binders on the porosity of composite electrodes for lithium-sulfur (Li-S) batteries was also addressed, the highest battery performance being observed for poly(VDF-*co*-HFP).^441^ In addition to Li-S batteries, PVDF has been used as cathode binder in Li-O_2_ batteries.^442^

Taking into account the recycling process for end-of-life batteries, recycling of the PVDF binder in batteries has been addressed, determining that LiOH·H_2_O reacts with this polymer binder, avoiding Li removal and the doping of the cathode material, mitigating the effects of its disposal.^443^ Also, the peel-off efficiency to delaminate cathode active materials has been evaluated, showing 98.5% of efficiency for lithium acetate–lithium nitrate (LiOAc-LiNO_3_) eutectic system (molar ratio of 3:2). Compared with single salts systems, this system demonstrates reduced energy consumption. LiNO_3_ also facilitates the PVDF decomposition, demonstrating that the proposed process is an environmentally friendly alternative when compared to direct calcination for cathode active material recycling.^444^

##### 3.3.2.2 Separator Membranes

**Figure 30:**
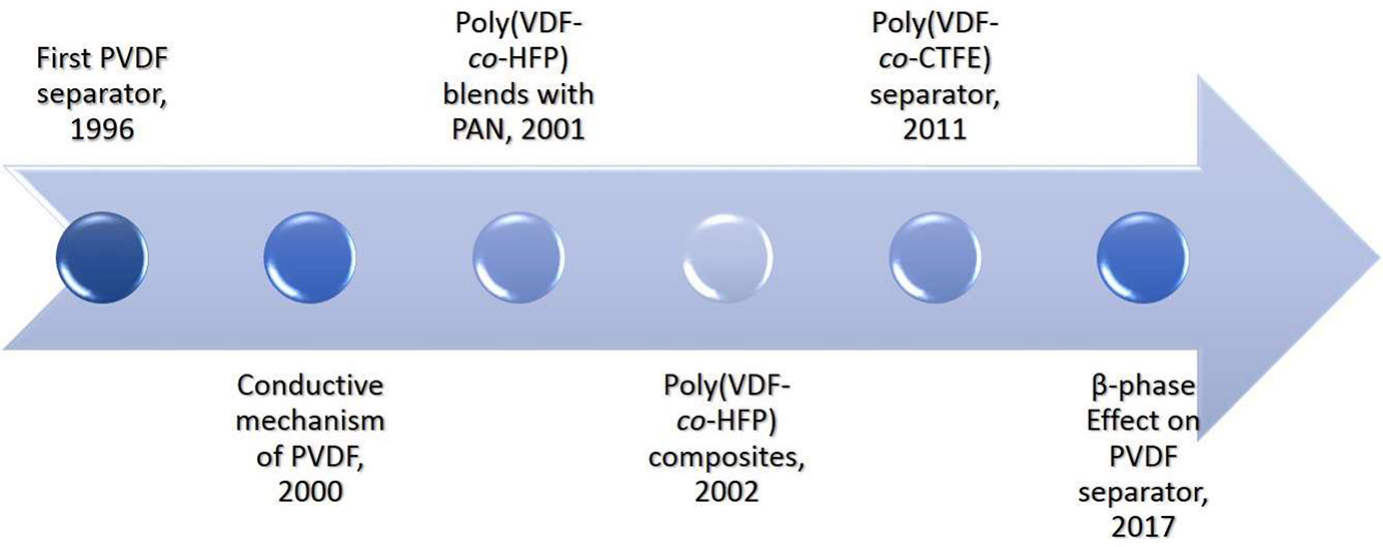
Main milestones regarding the development of PVDF-based separators.

PVDF-based separator membranes have been extensively studied, and Figure 30 shows some relevant milestones in this field over the years.

The first milestone in PVDF based separators was achieved by Tarascon et al. by developing the first battery with poly(VDF-*co*-HFP) as a separator membrane.^445^ Regarding PVDF, it was only in 1999 that a separator was produced by phase inversion, where the porous membrane was filled and swollen by the electrolyte solution, showing high ionic conductivity and good thermal stability.^446^

As a consequence of this result, the conduction mechanism of PVDF as separators was addressed, showing that the swollen gel dominates the ionic conduction process due to the interaction between the polymer and the electrolyte solution, which improves the mobility and content of the carrier.^447^

In the following years, the first poly(VDF-*co*-HFP) blends^448^ and poly(VDF-*co*-HFP) composites^449^ were applied in separator membranes, as shown in Figure 30.

Considering these scientific results, several blends and polymer composites have been developed, aiming to improve the thermal and mechanical properties, as well as the ionic conductivity of the separators.^86^

The first poly(VDF-*co*-CTFE) and poly(VDF-*co*-TrFE) separators were developed in 2011^450^ and 2012^451^ and applied in batteries of LiFePO_4_ and Sn–C half-cells,^452^ respectively.

**Figure 31:**
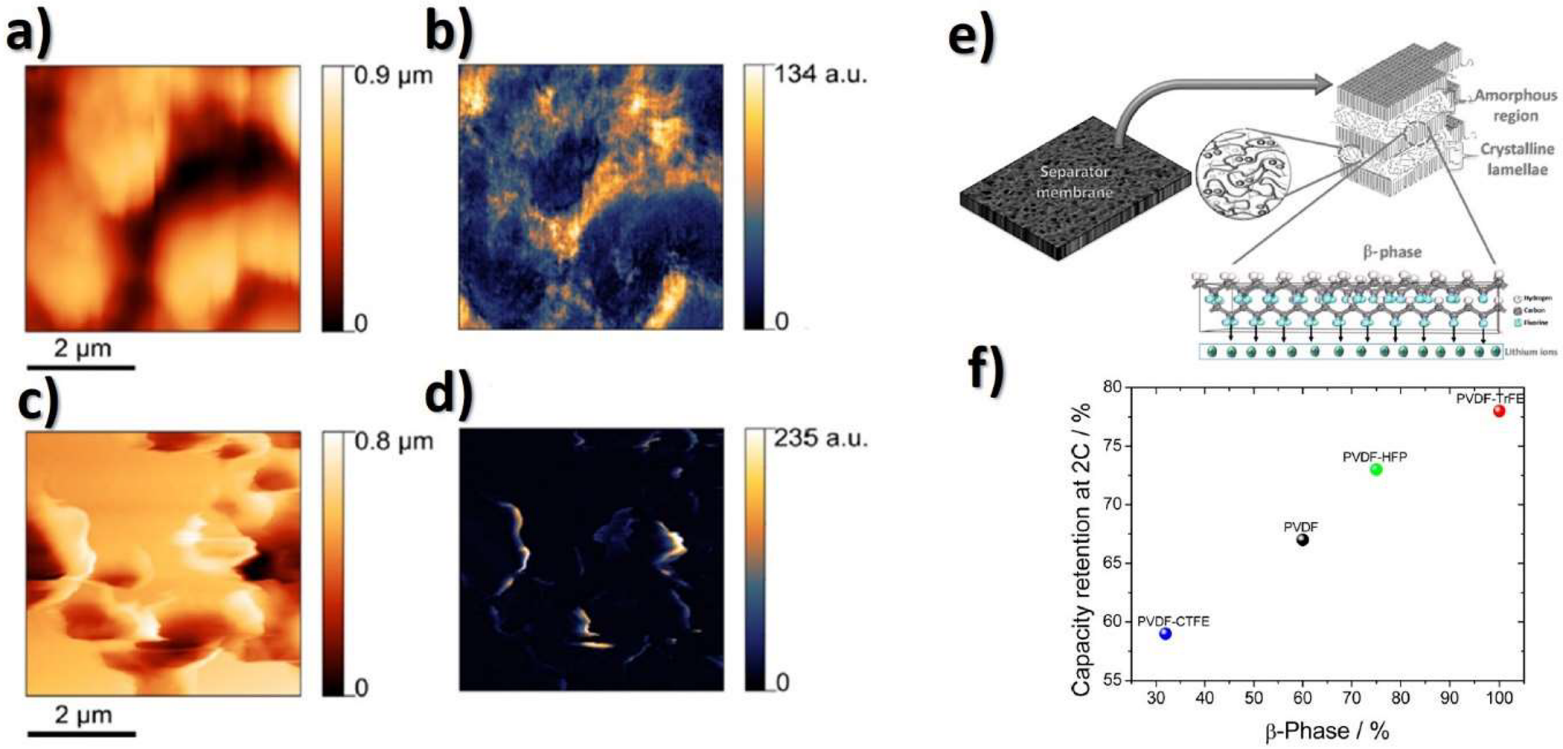
(a–c) Topography and (b–d) corresponding ESM magnitude of the poly(VDF-*co*-CTFE) and PVDF membranes, respectively. Reproduced with permission from ref (453). Copyright 2016 American Chemical Society. (e) Schematic representation of the interaction between Li-ions and the fluorine atoms of the β-phase of PVDF. (f) Capacity retention as a function of β-phase content for the different membranes. Reproduced with permission from ref (454). Copyright 2017 American Chemical Society.

Nanoscale Li diffusion and transport have been studied by electrochemical strain microscopy (ESM) in PVDF membranes, as shown in Figure 31a–d. The high strain observed was explained by the electroosmotic flow in the porous PVDF, which depends on the separator parameters: porosity, pore size, and electrolyte affinity.^453^

The effect of PVDF crystalline phase in battery separator performance was studied in 2017, demonstrating the high interaction between Li-ions and the polar β-phase (Figure 31e), the capacity retention increasing with increasing β-phase, resulting in faster migration of Li-ions within the membrane (Figure 31f).^454^

The intensive work and scientific advances on PVDF-based separators has been summarized in different reviews.^48,86,87^

Nowadays, PVDF and its copolymers are being implemented in all separator types, including microporous membranes, electrospun membranes, composites, polymer blends, and membranes with surface modification.^408^ Further, they are being implemented as separators for electrochemical capacitors, mainly due to PVDF strong C-F electron-withdrawing characteristics and high dielectric constant.^455^

Table 12 shows the most relevant works published on PVDF separators in the last years.

**Table 12:** Most Relevant Recent Works on Battery and Electrochemical Capacitor Separators Based on PVDF and Its Copolymers Sorted by Membrane Preparation Technique

membrane composition	preparation technique and procedure	electrolyte uptake/porosity (%)	ionic conductivity (mS·cm^–1^)	main goal/results	electrochemical systems	ref
poly(VDF-*co*-HFP) + Al_2_O_3_/ TiO_2_	coating	—/—	—	increased thermal stability	Li-ion	(471)
poly(VDF-*co*-HFP)	tape-casting	150/45	—	excellent thermal and mechanical properties	Li-ion	(473)
poly(VDF-*co*-TrFE)	template patterning	150–325%/—	0.8–1.6	increased discharge capacity	Li-ion	(468)
PVDF	melt blowing, electrospinning, and shear spinning	235–910/—	0.29–6.91	higher discharge capacity and rate capability	Li-ion	(457)
PEO/LIGC/Poly(VDF-*co*-TrFE)	electrospinning + coating	444/86	∼7	improved the mechanical strength and wettability	Li-ion	(469)
PVDF + GO	electrospinning	—/—	—	lower degree of crystallinity	Li-ion	(474)
poly(VDF-*co*-HFP) + Li_1.5_Al_0.5_Ge_1.5_(PO_4_)_3_	electrospinning	215/70	3.18	lower interfacial resistance change and a higher rate capability	Li-ion	(475)
PVDF + ZIF-8	electrospinning	1000/95	—	inhibit the growth of dendrites	Li-S	(476)
PVDF	electrospinning	200 ± 2/ 86.83 ± 2	—	superior electrochemical stability up to a voltage of about 2.5 V	electrochemical capacitor	(460)
PVDF/ montmorillonite	electrospinning	428/ 88.1	2.330	higher specific discharge capacitance and good compatibility with electrode materials	electrochemical capacitor	(462)
PVDF/TiO_2_	electrospinning	316/ 89	2.370	10 wt% PVdF/TiO_2_ membrane contributed to maximum ionic conductivity of and minimum crystallinity	electrochemical capacitor	(461)
PVDF + PVP + Al_0.1_Zr_0.9_O_1.95_	solvent casting	384/57	3–39	higher electrochemical stability window	Li-ion	(477)
PVDF + 2D NHNs	solvent casting	327.6/50–60	1.5	good cycling performance and rate capability	Li-ion	(464)
poly(VDF-*co*-HFP)	solvent casting	229/—	—	improves the mechanical and electrochemical performance	Li-ion	(466)
poly(VDF-*co*-HFP)/MXenes	solvent casting	—/—	—	improves Operation safety	Li-ion	(478)
poly(VDF-*co*-HFP) + SiO_2_	solvent casting	∼250/—	1.34 - 2	excellent thermal stability and dendrite suppression capability	Li-ion	(479)
poly(VDF-*co*-HFP) + Si_3_N_4_	solvent casting	∼250/75.2	0.884	improved electrochemical stability	Li-ion	(480)
poly(VDF-*co*-HFP) + ZIF-8/ MXene	solvent casting	—/—	4.4	higher Li ion transportation and enhanced tensile strength.	Li-ion	(481)
poly(VDF-*co*-HFP)/PMMA/CMC	solvent casting	—/—	∼4.4	improved migration rate of Li-ions	Li-ion	(482)
PVDF + ionic liquid	solvent casting	—/—	4.1	improved deposition/dissolution of the Li anode	Li-O_2_	(464)
poly(VDF-*co*-HFP) + different MOFs	solvent casting with thermal-induced phase separation (TIPS)	350–550/65–77	2.2–3.9	improved battery performance	Li-ion	(59)
PVDF	solvent casting + nonsolvent induced phase separation	162 – 304/54–65	1.2	good electrochemical stability and cycling performance	Li-ion	(456)
PVDF/PMIA	solvent casting + nonsolvent induced phase separation	—/—	0.75	improved wettability and higher thermal stability	Li-ion	(463)
PVDF/ TEABF_4_ electrolyte	solvent casting + table salt incorporated	—/—	—	direct conversion and storage of mechanical energy to electrical energy in an integrated device	electrochemical capacitor	(483)

With respect to microporous membranes, PVDF membranes have been prepared by the NIPS method in which the NMP/acetone mixture was used as solvent and ethanol/deionized water as nonsolvent. It was verified that the use of ethanol as a nonsolvent results in more uniform membranes with higher porosity, all produced membranes showing good electrochemical stability and cycling.^456^

PVDF membranes with different pore sizes and pore size distributions have been developed by melt blowing, electrospinning, and shear spinning. It was verified that shear spinning is a suitable emerging method to manufacture nanofibrous materials with morphology control from fibrous-like to nanosheet-like membranes, allowing tailoring of electrochemical cell response.^457^

Considering that electrospinning is one of the most used methods for obtaining porous membranes, electrospun PVDF membranes have been produced by combining electrospinning and TIPS techniques through the DMF/acetone solvent system in order to further tune membrane microstructure. The highest discharge capacity value being obtained for PVDF membranes prepared with the DMF/acetone (4:6) solvent system, leading to a highly interconnected porous morphology.^458^

Electrospun PVDF membranes have been optimized by using different polymer concentration (18 wt %, 21 wt %, 24 wt %, and 27 wt %) in solution with DMA, the PVDF solution concentrations of 24 wt % showing improved electrochemical properties.^459^ Moreover, PVDF fiber mats prepared with a polymer concentration of 20 wt % are demonstrated to be more suitable for supercapacitor applications.^460^ It has been also demonstrated that the addition of TiO_2_ particles increases the stability of the supercapacitors from 2.5 to 2.99 V.^461^ The addition of montmorillonite to poly(VDF-*co*-HFP) fibers (400 nm) leads to higher thermal stability and mechanical strength. Further, 5 wt % higher porosity (88.1%) than the prostine fiber mats and an ionic conductivity of 2.330 × 10^–3^ S·cm^–1^ allows obtaining of a specific capacitance of the Li-ion capacitor of 149.2 F·g^–1^ at a current density of 1 A·g^–1^ and a retention of 90% of the initial capacitance after 2000 cycles.^462^

To increase thermal stability and electrolyte wettability, ethyl cyanoacrylate (ECA) has been incorporated into a PVDF membranes, as ECA shows strong interaction with the organic solvents in the electrolyte solution, leading to superior capability for electrolyte uptake.^463^

For PVDF, composites have been produced with 2D nickel hydroxide nanosheets (NHNs), the composite showing high β-phase content, which benefits the ionic conductivity and electrochemical battery performance.^464^ Also, PVDF composites with vermiculite (V) and laponite nanoparticles have been developed, leading to lower interfacial impedance and excellent Coulombic efficiency.^465^

Another strategy in this separator type is the fabrication of multilayers with high porosity and lamellar structure, obtained by a combination of evaporation-induced phase separation and selective solvent etching methods that benefit the mechanical and electrochemical properties.^466^

To develop devices based on environmentally friendlier processes, such as additive manufacturing techniques, PVDF, and poly(VDF-*co*-HFP) membranes, have been prepared by DIW varying solvent evaporation temperature and fill density percentage. The highest ionic conductivity value of 3.8 mS·cm^–1^ was obtained for poly(VDF-*co*-HFP) membrane prepared with a fill density of 100.^467^

**Figure 32:**
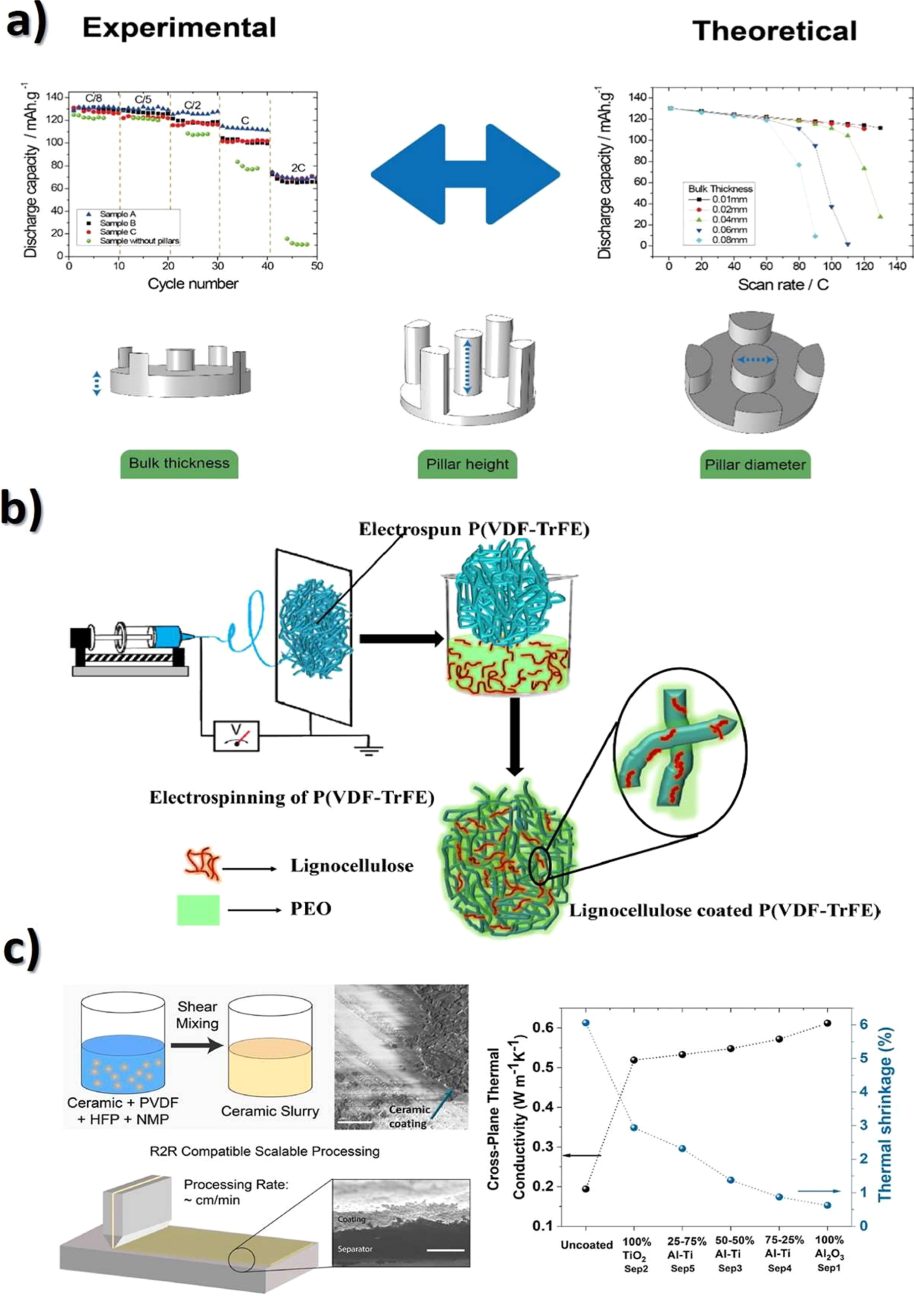
(a) Patterned separator membranes with pillar surface microstructures: experimental and theoretical work. Reproduced with permission from ref (468). Copyright 2021 Elsevier. (b) Schematic representation of PEO/ lignocellulose coated poly(VDF-*co*-TrFE) membranes. Reproduced with permission from ref (469). Copyright 2022 Elsevier. (c) Schematic representation of poly(VDF-*co*-HFP) coatings with thermal shrinkage results. Reproduced with permission from ref (471). Copyright 2020 Elsevier.

Due to its low degree of crystallinity, poly(VDF-*co*-TrFE) is ideal for separator membranes. For this polymer, membranes with surface pillar microstructures have been developed for battery separator applications, varying pillar diameter, height, and bulk thickness. Through the combination of experimental and theoretical work (Figure 32a), it has been shown that the parameter that most influences battery performance is the bulk thickness.^468^

In addition, to improve sustainability and wettability, electrospun poly(VDF-*co*-TrFE) membrane with PEO/lignocellulose coating has been produced, as presented in Figure 32b. This membrane exhibits superior properties when compared to commercial Celgard separators.^469^ A coating of polyvinyl butyral (PVB) with SiO_2_ nanoparticles has been also applied to the surface of PVDF separators. This separator structure leading to improved cycling performance and higher discharge capacity rate capability when compared to the neat PVDF separator.^470^

In order to improve the thermal stability and electrolyte affinity of commercial separators, the coating of poly(VDF-*co*-HFP) with ceramic particles (Al_2_O_3_ and TiO_2_) was carried out, as shown in Figure 32c, the coating improving the thermal stability and leading to batteries with excellent capacity retention.^471^ Further, the wettability of commercial separators can be improved by electrospinning or drop-casting of PVDF polymer solutions containing GO.^472^

With respect to composite membranes, metal–organic frameworks (MOFs) are being explored as fillers. ZIF-8@MXene has been found to provide ion transport channels to improve Li^+^ transport.^481^ Different MOFs (MOF-808, MIL-125 and UiO-66-NH_2_) have been used to improve battery performance by reducing the resistivity of the batteries, the best performance being achieved for UiO-66-NH_2_ due to the low half-cell resistivity value.^59^

In order to suppress Li dendrites and fire retardance, a novel separator based on poly(VDF-*co*-HFP) with MXenes was developed, leading to a homogeneous growth rate of Li dendrites and thus forming a smoother SEI layer during cyclings.^478^ In addition, composites based on poly(VDF-*co*-HFP)/Li_1.5_Al_0.5_Ge_1.5_(PO_4_)_3_^475^ and silicon nitride (Si_3_N_4_) whiskers^480^ have been produced to improve battery cycling performance.

For Li-S batteries, electrospun membranes based on MOFs@PVDF have been developed to facilitate uniform Li deposition and reduce the generation of Li dendrites.^476^

Further, new blends based on PVDF with poly(*m*-phenylene isophthalamide) (PMIA),^463^ PU,^484^ and polyacrylonitrile/vermiculite nanosheets (PAN/VNs)^485^ have been fabricated to improve wettability and thermal stability. Similarly, blends based on poly(VDF-*co*-HFP) with PMMA and carboxymethyl cellulose (CMC) allow improvement of thermal stability and electrochemical stability.^482^ A different concept of a self-charging electrochemical capacitors with porous PVDF and ionic-liquid electrolyte has been reported.^483^ In this study, the separator is placed between two Gr electrodes and its performance as capacitor and energy harvesting evaluated. It is shown that 4–11 V were generated with compressive forces between 5 and 20 N, respectively, and a specific capacitance of 28.46 F·g^–1^ was obtained for the device. The results provide new insights toward new generation all-in-one energy conversion/storage devices.

##### 3.3.2.3 Solid Polymer Electrolytes

Considering that the electrolyte solution used in the separator membrane is typically toxic and easily leaked, its elimination is important and essential for the next generation of safer and environmental friendlier solid-state batteries, where SPEs play an essential role.^486^ The SPEs is basically composed of one or two polymer matrices with one or more fillers and must be characterized by improved mechanical and thermal stability and large ionic conductivity.^478^

The polymer matrices in SPEs are responsible for the thermal and mechanical stability, while providing electronic insulation. In the case of fillers, they are responsible for the ionic conductivity value and Li transference number.^487^

In the case of PVDF for SPEs applications, there are different combinations, such as a polymer with one filler,^488^ a polymer with two fillers,^489^ and composites with more than three components.^490^

The fillers used in SPEs development for improving ionic conductivity are basically based on different Li salts and ILs. Table 13 shows the recent advances in PVDF-based SPEs

**Table 13:** Recent Advances in the PVDF-Based SPEs.

polymer matrix	fillers	preparation technique	ionic conductivity (mS·cm^–1^)	Li^+^ transfer number	electrochemical capacity/ capacitance (mAh·g^–1^/F·g^–1^)	main goal/ achievement	ref
PVDF	LLZTO + MA	solvent casting	1.15	0.596	170.5 at 0.2C	excellent long-term cycling stability	(494)
PVDF	Li_0.33_La_0.56_TiO_3_ (LLTO)	solvent casting	1.7	—	120 at 0.5C	suppress the Li dendrite growth	(509)
PVDF	LiTFSI	electrospinning	0.60	0.58	113 at 1.0C	outstanding rate capability and charge–discharge cycling behavior	(495)
PVDF	LiTFSI and ZIF-90	solvent casting	0.62	0.48	118 at 1.0C	high specific discharge capacity	(510)
PVDF	LiBOB	vacuum oven	0.061	—	267 at 1 A·g^–1^	excellent fexibility with almost zero performance degradation after 10 000 bending cycles	(497)
PVDF and PEC	Li_6.28_La_3_Zr_2_Al_0.24_O_12_ (Al-LLZO) and LiTFSI	solvent casting	0.391	0.78	162.59 at 0.1C	high capacity retention (92%) after 100 cycles	(511)
PVDF and PEO	LiTFSI and Gd-doped CeO_2_	electrospinning	0.23	0.64	119.4 at 1C	inhibit the growth of Li dendrites	(512)
PVDF and PEO	LATP	solvent casting	0.44	0.68	163.3 at 0.1C	wide electrochemical stability windows	(481)
PVDF and PEO	LiClO_4_ and ZnO	solvent casting	0.31 at 60 °C	0.768 at 60 °C	99 at 1C (60 °C)	excellent cycle stability (more than 1000 h)	(513)
PVDF and PEO	LiTFSI and Li_7_La_3_Zr_2_O_12_ (LLZO)	solvent casting and freeze-drying	4.22 × 10^–3^		109.7 at 1C (50 °C)	suppress Li dendritic growth and good interfacial contact	(514)
PVDF	PVA/H3PO_4_	solvent casting + coating			263 at 20 mV.s^–1^	good electrochemical performance	(515)
PVDF	BaTiO3	solvent casting + coating			312 at 20 mV.s^–1^	good electrochemical performance	(515)
poly(VDF-*co*-HFP)	LATP with graphitic-C3N4	solvent casting	2.55 × 10^–2^	0.65		good compatibility and electrochemical stability with Li metal.	(516)
poly(VDF-*co*-HFP)	LATP, LiTFSI with CeO_2_	solvent casting	1.66	0.35	166.6 at 0.1C	suppression of Li dendrites	(517)
poly(VDF-*co*-HFP)	LiTFSI	solvent casting	1.40 × 10^–2^	0.18	135 at 0.1C	improved interfacial contact between the electrodes and electrolyte	(518)
poly(VDF-*co*-HFP)	LLTO and LiTFSI	electrospinning	0.38	0.42	70 at 2.0C	improved rate capability and cycling stability	(519)
poly(VDF-*co*-HFP)	LiTFSI and LiNO_3_	solvent casting	0.566			dense Li deposits without dendrites	(520)
poly(VDF-*co*-HFP)	LiTFSI and porous carbon	solvent casting	0.56	0.26 at 60 °C	143.7 at 0.15C	excellent cycling stability	(507)
poly(VDF-*co*-HFP)	Li_7_La_3_Zr_2_O_12_ (LLZO) + LiODFB	solvent casting	0.165		135 at 0.5C	reduces polymer crystallinity and provides a large phase interface	(510)
poly(VDF-*co*-HFP)	Li_6.4_La_3_Zr_1.4_Ta_0.6_O_12_ (LLZTO) and LiTFSI	solvent casting	0.32	0.66	150 at 0.1C	good electrochemical performance	(521)
poly(VDF-*co*-HFP)	N-LLZTO	solvent casting	0.17	0.57	120 at 0.5C	suppress the Li dendrite and serves more than 2000 h	(522)
poly(VDF-*co*-HFP)	[Bmim][SCN] and clinoptilolite zeolite	solvent casting	0.19		160.3	excellent battery performance at room temperature	(523)
poly(VDF-*co*-HFP) and PEG	Li_6.4_La_3_Zr_1.4_Ta_0.6_O_12_ (LLZTO)	solvent casting	0.85	0.59	88 at 5C	suppress the Li dendrite growth	(524)
poly(VDF-*co*-HFP) and PEO	LiTFSI	electrospinning	2.57 at 80 °C	0.2–0.4 at 7–90 °C	160 at 0.1C	improves the cycle stability from short-circuiting at 144 h	(503)
poly(VDF-*co*-HFP) and PEO	LiTFSI and AgNWs@SiO_2_	electrospinning	0.04 at 50 °C	0.29	92.7 at 2C (90 °C)	high mechanical strength and wide electrochemical stability window	(506)
poly(VDF-*co*-HFP) and PEO	LLZTO	hot press	0.62 at 80 °C		161 at 0.2C	good electrochemical stability	(525)
poly(VDF-*co*-HFP) and PEO	Li_6.7_La_3_Zr_1.7_Ta_0.3_O_12_ (LLZTO) and LiTFSI	solvent casting	0.1	0.52 at 60 °C	133.4 at 0.5C	excellent rate performance	(521)
poly(VDF-*ter*-TrFE-*ter*-CTFE)	LiTFSI	solvent casting	0.31	0.33	106.7 at 0.3C	stable cycling performance at 25 °C	(526)

Li salts increase the ionic conductivity of the SPE, as a result of increasing the number and mobility of free charges in the system.^491^ Concerning ILs, there are two contributions: the increase in ionic conductivity through the introduction of specific anions and cations and also the reduction of crystalline domains, which promotes the conduction process in the amorphous component of the polymer matrix.^140^

For redox flow batteries, a new SPE composite membrane based on PVDF and Li_1.4_Al_0.4_Ge_0.2_Ti_1.4_(PO_4_)_3_ (LAGTP) has been investigated with a high ionic conductivity value of 0.27 mS·cm^–1^ and a Coulombic efficiency of over 97%.^492^ In this context, Li_1.3_Al_0.3_Ti_1.7_(PO_4_)_3_ (LATP) and LAGTP have also been used as fillers based on their electrochemical stability window.^493^

**Figure 33:**
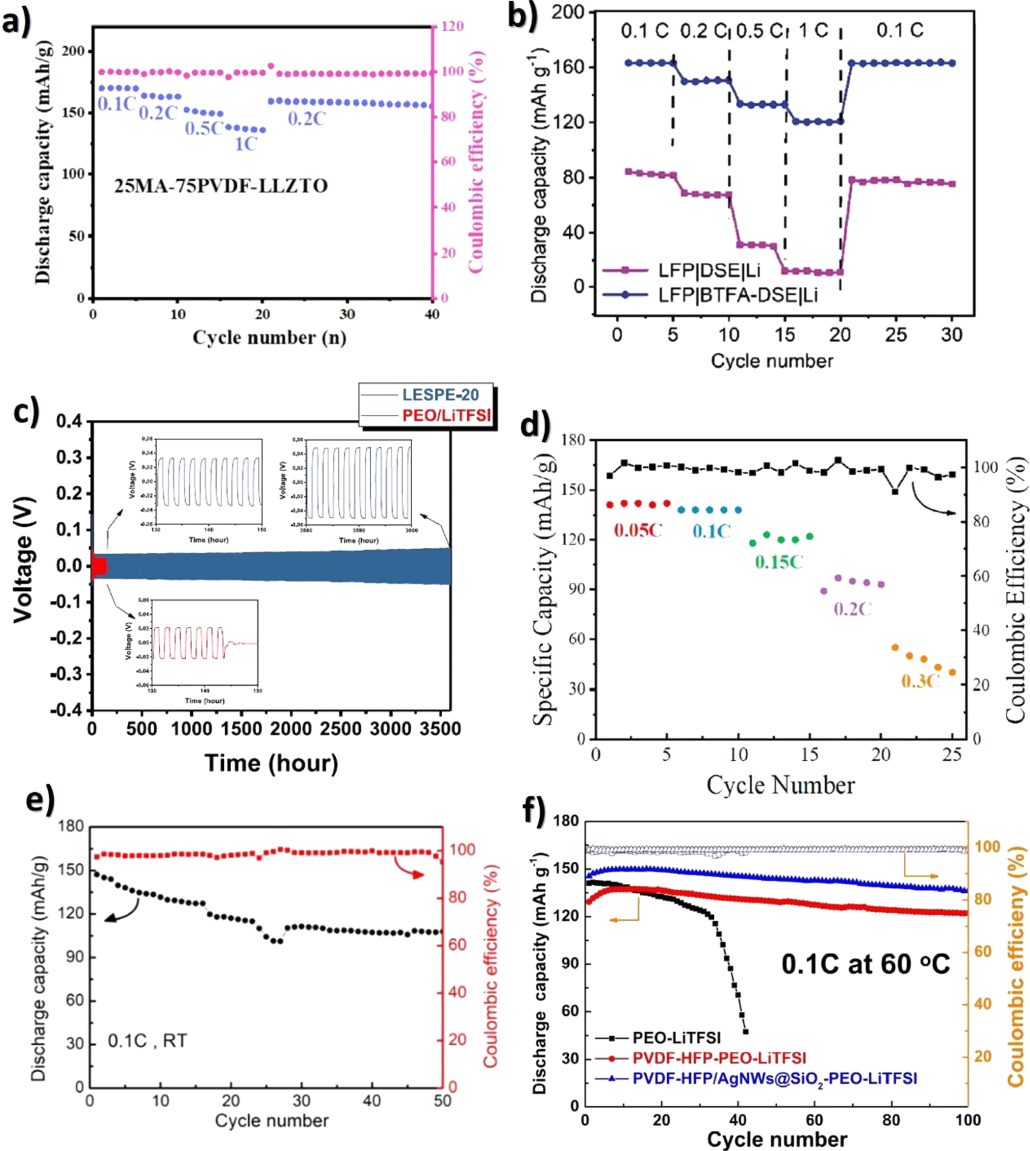
(a) Rate capability of 25MA-75PVDF-LLZTO. Reproduced with permission from ref (494). Copyright 2022 Elsevier. (b) Rate performances of LFP|DSE|Li and LFP|BTFA-DSE|Li cells. Reproduced with permission from ref (500). Copyright 2022 Wiley-VCH. (c) Lithium plating/stripping test for symmetric Li cells at 80 °C. Reproduced with permission from ref (503). Copyright 2021 American Chemical Society. (d) Discharge capacities and Coulombic efficiency of the SPE at 60 °C. Reproduced with permission from ref (504). Copyright 2021 Elsevier. (e) Cycling performance of SPE based on poly(VDF-*co*-HFP) at 0.1C in 50 cycles. Reproduced with permission from ref (505). Copyright 2021 Elsevier. (f) Cycle performance of SPEs based on poly(VDF-*co*-HFP)/AgNWs@SiO2-PEO/LiTFSI at 0.1 C at 60 °C. Reproduced with permission from ref (506). Copyright 2021 Wiley-VCH.

For Li-ion batteries, a composite SPE based on maleic acid (MA) with PVDF-LLZTO(Li_6.75_La_3_Zr_1.75_Ta_0.25_O_12_) has been produced (Figure 33a). This SPE shows a specific discharge capacity of 170.5 mAh·g^–1^ at 0.2 C and a high rate capability up to 1.0 C with 138 mAh·g^–1^.^494^ A SPE based on PVDF demonstrated that the crystalline phase affects battery performance, and the more suitable PVDF phase is the polar β-phase.^495^ Recently, a SPE based on PVDF with lithium perchlorate (LiClO_4_) has been developed and deposited in a polypropylene (PP) separator for mechanical reinforcement with a high ionic conductivity value of 0.15 mS·cm^–1^.^496^

Considering electrochemical capacitor applications, poly(VDF-co-HFP)/LiBOB SPE have been developed as flexible solvent-free Li-ion symmetric supercapacitors. In this study,^497^ BOB^–^ acts as a solid plasticizer and increases the ionic conductivity to 6.1 × 10^–5^ S·cm^–1^ (25 °C) and 5.7 × 10^–4^ S·cm^–1^ (80 °C) due to the enhancement of ion transport in te polymer solid electrolyte. The prepared material also was used as electrode layer to host GO/CNTs. An electrochemical capacitor with a specific capacitance of 267 F·g^–1^ at 1 A·g^–1^ was obtained for this solvent-free fexible supercapacitors. In order to improve the performance of this SPE, the same research group developed an on-chip solution with poly(VDF-*co*-HFP)/LiBOB/TiO_2_ SPE with *in situ* fabricated 3D polysilicon/nickel nanoforest electrodes, leading to a device capacitance of 4.32 F·cm^–3^ and high volumetric power density of 4.15 W·cm^–3^ at 1.25 mA·cm^–2^. The *in situ* monolithically integrated silicon/nickel nanoforests provided a new route to develop 3D electrode material with microscale patterns, avoiding *ex situ* active material transfer processes that normally require large interspace and electrode width (>50 μm).^498^

Another strategy to improve battery performance for SPEs based on PVDF is the synthesis with perfluoro-2-methyl-3-oxa-5-sulfonimido[-3-oxa-5-sulfonyl fluoride] vinyl ether (VEPFSIS) initiated by potassium persulfate, leading to ionic conductivity values higher than 10^–4^ S·cm^–1^ at 30 °C.^499^

An SPE composite with PEO, PVDF, lithium aluminum titanium phosphate (LATP), and bistrifluoroacetamide (BTFA) as plasticizer has been produced with excellent cycling and rate performance, as shown in Figure 33b.^500^ Another SPE based on LB, lithium bis(trifluoromethanesulfonyl)imide (LiTFSI), PMMA, and PVDF has been produced with continuous Li^+^ conduction pathways through LATP structure.^501^ Furthermore, a SPE based on PVP and polyetherimide (PEI) formed close stacks, with Janus PVP dispersed in the PVDF matrix. Together with LiTFSI salt, the developed SPE shows an ionic conductivity of 5.1 × 10^–4^ S·cm^–1^ and a high specific capacity of 122.1 mAh·g^–1^ discharge at the 100th cycle.^502^

A new approach for SPEs development involved the implementation of a PEO/LiTFSI electrolyte matrix laminated on both sides by electrospun poly(VDF-*co*-HFP) membranes, the main achievement being the absence of short-circuiting up to 3600 h as shown in Figure 33c.^503^ Another SPE with the same components but also with *N*-methyl-*N*-propylpyrrolidinium bis(trifluoromethanesulfonyl) imide (PYR_13_TFSI)^507^ and MWCNT-COOH^508^ showed excellent stability against the Li anode and significantly increased Li^+^ migration ability, respectively.

Also for poly(VDF-*co*-HFP), a SPE with Li salts and porous carbon has been produced with a high ionic conductivity of 0.56 mS·cm^–1^ at room temperature. The battery performance is shown in Figure 33d.^504^

Polymer/garnet solid oxide composite electrolytes based on Li_6.4_La_3_Zr_1.4_Ta_0.6_O_12_ (LLZTO) and LiTFSI has been produced to improve contact with the electrodes and therefore leading to improved electrochemical performance, as shown in Figure 33e.^505^

A electrospun composite polymer membrane has been also manufactured based on poly(VDF-*co*-HFP) with silica-coated Ag nanowires (AgNWs@SiO_2_) and PEO/LiTFSI. This SPE features high mechanical strength and wide electrochemical stability window, the battery performance being shown in Figure 33f.^506^

To increase battery performance at room temperature, a three-component SPE based on poly(VDF-*co*-HFP), the ionic liquid 1-butyl-3-methylimidazolium thiocyanate ([BMIM][SCN]), and clinoptilolite zeolite (CPT) has been produced, the SPE showing a battery performance of 160.3 mAh·g^–1^ at a C/15-rate, with a capacity retention of 76% after 50 cycles.^523^

Another strategy that has been applied for SPE development is *in situ* polymerization to reduce the interface resistance on the electrode surface of a porous polymer film (poly(VDF-*co*-HFP)/PVDF) by curable monomers, including PEGDA/PETMP/TFEMA.^507^ Further, in order to improve the compatibility between electrode and SPE, a hybrid SPE based on Al-doped LLZO and poly(VDF-*co*-HFP) has been obtained with an ionic conductivity of 0.4 mS·cm^–1^.^527^

Considering the high dielectric constant of poly(VDF-*ter-*TrFE-*ter*-CTFE) that promotes a stronger solvation capacity for Li-ions, a SPE has been developed with LiTFSI, showing a stable cycling performance at 25 °C.^526^

Another route involving the use of gel polymer electrolytes (GPEs) in electrochemical capacitors has been studied due to the quasi-solid-state polymer electrolyte characteristics. This system combines the best characteristics of solid (cohesive properties) and liquid (diffusive properties) electrolytes by promoting the amorphous polymer content and decreasing its glass transition temperature, increasing the ion mobility. The combination of PVDF with PVA/H_3_PO_4_ and BaTiO_3_ gel electrolytes for electrochemical paper based capacitors show that, compared with PVDF gel electrolytes, the prepared samples improve the specific capacitance from 176 F·g^–1^ to 263 F·g^–1^ and 312 F·g^–1^, respectively.^515^

In summary, PVDF and its copolymers are the most widely used polymers in the field of energy storage for the different battery components, due to the simple processing, high thermal and chemical stability, and excellent swelling properties in contact with different electrolyte solutions, among others. The focus of PVDF research in the area of energy storage is mainly to tailor overall properties to increase the electrochemical properties of the different battery components and the full battery. Further, green chemistry synthesis and recycling strategies of the polymer must be strongly improved to allow more sustainable energy storage systems.

##### 3.3.2.4 Electrostatic Capacitors

Regarding electrostatic capacitors, PVDF offers exciting possibilities for improving the performance of dielectric capacitors. Thus, PVDF-based films, nanocomposites, and blends have been evaluated to enhance the energy storage capacity, breakdown strength, and dielectric constant of capacitors.^528^ To increase the dielectric constant, parameters such as type of filler, compatibilization with the polymeric matrix, and filler content play an important role.^529^ In these composites, different carbonaceous materials,^530^ MXene,^531^ ceramic oxides,^532^ AgNbO_3_ (ANO),^533^ and other particles have been used as fillers to improve the dielectric constant and decrease the dielectric loss.^528^

For example, composites of plate-like (Ba_0.6_Sr_0.4_)TiO_3_ (P-BST) particles (particle size of 11.47 μm) with PVDF were prepared by tape casting, leading to a high dielectric constant of 62.^534^ Also, membranes based on PVDF with surface-modified graphene (SMG) were developed by electrospinning technique where the inclusion of 16 wt % of SMG leads to an increase of the dielectric constant to 84 at 1000 Hz, which is 10 times higher than for PVDF without filler.^535^

In order to develop a multifunctional composite in which the fillers can be aligned during the manufacturing process by an external magnetic field, nickel nanowires (Ni NWs) were introduced in a poly(VDF-*co*-HFP) matrix, the highest dielectric constant being 41 at 1 kHz for the composite with 1.3 vol % of Ni NWs.^536^ In addition, composites of poly(VDF-*ter*-TrFE-*ter*-CFE) with MXene were produced to improve the dielectric constant up to 10^5^ near the percolation limit of 15.3 wt % MXene content.^537^

By tailoring the molecular structure and incorporating suitable fillers or additives, PVDF-based dielectric materials can exhibit enhanced electrical properties. Future advancements may involve the development of PVDF-based capacitors with higher energy density, improved reliability, and increased operating temperatures.^538^

### 3.4 Environmental Monitoring and Remediation

Water contamination is a global problem that has received increased attention from the scientific community in recent decades. Anthropogenic pollution is the main cause of water contamination. The increasing lifestyle quality also enhanced the use of chemicals such as pharmaceuticals, personal care products, pesticides, and endocrine disruptors.^539^ The fate of all these compounds is invariably the effluents regardless if they have been previously treated or not. Generally, these chemicals are known as emergent pollutants and can be found in very low concentrations (ng·L^–1^ to mg·L^–1^) in contaminated and post-treated water.^540^ These pollutants are chemically stable and resistant to classical physical, chemical, and biological water treatments. Even at low concentrations, these pollutants threaten the aquatic environment though toxic and bioaccumulation processes of many hazardous compounds in aquatic organisms.^539,541^ Such context urges new approaches to tackle this problem. Among them, photocatalysis,^542^ adsorption,^543^ and membrane technologies^544^ have become attractive and are simple and efficient treatments against many water pollutants.

These techniques need advanced materials to be successfully applied, and fluorinated polymers such as PVDF, and its copolymers (e.g., poly(VDF-*co*-TrFE) and poly(VDF-*co*-HFP)) possess interesting physicochemical properties that allow several applications, including water remediation. Furthermore, such polymers can be easily processed into thin films, membranes, coatings, and fibers, broadening the range of possible applications. Additionally, these polymers show excellent chemical, mechanical, thermal, and UV radiation resilience, which is related to the polymer structure’s stable C–F bonds.^11,47,545^ The possibility of controlling the porosity and pore size is also critical for these applications.^546,547^ Similarly, these polymers withstand a large variety of fillers (e.g., nano/microparticles, zeolites, MOFs) that endow the membranes with photocatalytic and adsorptive properties, significantly enhancing their efficiency against specific contaminants or acquiring new properties.

Table 14 lists some works based on PVDF-based materials for water remediation applications, mainly focusing on photocatalysis and adsorption processes that are endowed by adding fillers with specific affinity for target contaminants.

**Table 14:** Relevant Works That Use PVDF-Based Materials to Develop Water Remediation Materials, With the Indication of Relevant Aspects Such As Composite Preparation Technique, Filler, Treated Contaminant, Type of Radiation, Contact Time, And Removal Efficiency

polymer matrix	fillers	preparation technique	contaminant	radiation	irradiation time	degradation efficiency	ref
Photocatalysis							
PVDF	Ni-ZnO	solvent casting	antifouling/MB	UVA	6 h	50% (105 min)	(548)
PVDF	MIL-125 (Ti)	wet phase inversion	antibacterial/Self-cleaning/ RhB	Xe lamp			(549)

PVDF	BiVO_4_-GO	ultrasonication	MB, RhB, Salfranin	Xe lamp	3 h	65% (MB)	(550)
						60% (RhB)	
						83% (Salfranin)	

PVDF/PVP	P25-TiO_2_	electrospinning	BPA and EE_2_	UVA	4 h (BPA)	>96%	(551)
					1.5 h (EE_2_)		

PVDF	ZnO and TiO_2_	CVD	diclofenac	UV	6 h	100%	(552)
PVDF	TiO_2_@MoSe	hydrothermal	RhB and LVX	white LED	2 h	100% (RhB) and 27% (LVX)	(553)
PVDF/PMMA	TiO_2_	VIPS and NIPS	MB	UV	3 h	99%	(554)
poly(VDF-*co*-HFP)	CuO_2_	ES	MB	tungsten bulb	3.2 h	76%	(555)

poly(VDF-*co*-HFP)	Ag-TiO_2_	TIPS /electrospinning	antifouling/NOR	UV(385 nm)	90 min (UV)	64% (UV)	(556)
				Xe lamp	300 min (Xe)	81% (Xe)	

polymer matrix	fillers	preparation technique	contaminant	contact time	removal	ref
Adsorption						
PVDF	La(OH)_3_	NIPS	phosphate	1.5 h	90%	(557)

poly(VDF-*co*-HFP)	MIL-68(Al)	TIPS	*p*-nitrophenol (PNP) MB	12 h	94% (PNP)	(558)
					96.3% (MB)	

Pani/PVDF-HFP		electrospinning	Cr (VI)	24 h	95% (1 cycle) and 71% (5 cycle)	(559)
PAA/PVDF	ZIF-8	phase inversion	Ni(II)	72 h	99% (qe = 219 mg·g^–1^)	(560)
PVDF/PAN	MOF808	ES	Hg and Pb		qe_Hg_= 213 and qe_Pb_= 86 mg·g^–1^	(561)
poly(VDF-*co*-HFP)	Fe_3_O_4_	TIPS	As(III) and As(V)	6 h	qe_As(III)_= 93 and qe_As(V)_= 138 mg·g^–1^	(562)
poly(VDF-*co*-HFP)	Y_2_(CO_3_)_3_	TIPS	As(V)	2 h	90%	(563)
PVDF	CNT-H-AC	NIPS/filtration	phenol, bisphenol A, nitrophenol, and sulfamerazine	2 h	60%, 70%, 40%, and 30%	(564)
PVDF-PEDOT		electrospinning	MO	25 h	qe_MO_ = 144	(565)
PVDF	HNTs–PSBMA and PDA	coating	Cu^2+^ and Cr^6+^		qe_Cu_^2+^ = 66.5 and qe_Cr_^6+^ = 76.1 mg^–1^	(566)
PVDF	BNN	NIPS	MB		qe_MB_ = 143 mg·g^–1^	(567)
PVDF	MIL-125	NIPS	RhB	7 h	∼90%	(568)
PVDF	SnO_2_	NIPS	Pb^2+^, Cu^2+^, Zn^2+^, Cd^2+^, and Ni^2+^		94%, 93%, 82%, 71%, and 64%	(569)
poly(VDF-*co*-HFP)	UiO-66-NH2	TIPS	Cr(VI)	24 h	Qe_C_ = 59.9 mg·g^–1^	(570)

Another possible application for these materials is for environmental sensing to monitor low concentrations of organic and inorganic contaminants in different water matrices. For instance, ZnO–TiO_2_ nanofibers have been produced by electrospinning and hydrothermal methods and deposited over a PVDF-formed nanofiber membrane in order to detect copper and lead ions.^571^ Poly(4-vinylpyridine) (P4VP) functionalized PVDF membranes have been used to detect mercury in water solution, detecting concentrations of 1 mg·L^–1^ in 2 h and l ng·L^–1^ after 24 h of adsorption. The authors also envisage this platform for detecting other heavy metals.^572^ In the framework of plasmonic sensors, a carbon nanotube/gold nanoparticle (CNT/AuNP) nanocomposite placed into a commercial PVDF has been used as a sensor.^573^ The sensor allowed to detect several molecular contaminants (e.g., melamine, paraquat) in concentrations ranging from 1 nM to 1 μM in aqueous samples, demonstrating its excellent field-testing and applicability characteristics for environmental monitoring. Despite the exciting news in environmental monitoring using PVDF and copolymers, the following subchapters will focus on environmental remediation based on photocatalysis and adsorption.

#### 3.4.1 Photocatalysis

As previously mentioned, photocatalysis is one of the emerging techniques suitable for addressing some relevant environmental problems. However, to apply photocatalytic processes in a polymeric substrate, it must also present a high chemical stability to resist UV radiation and oxidative species produced by the photocatalytic nanoparticles (e.g., TiO_2_, ZnO, and WO_3_). In this context, PVDF and its copolymers have the necessary processability and chemical stability to produce, use, and reuse photocatalytic nanocomposite membranes.

Reports on photocatalytic^544,574,575^ and adsorptive^576,577^ membranes for water treatment have strongly increased. Anran Zhou and co-workers have produced a PVDF-PVP-TiO_2_-dopamine (DA) (PPTD) functionalized ultrafiltration membrane. Including TiO_2_, PVP, and DA into the PVDF structure improved hydrophilicity, pore size, and porosity, favoring water flux through the polymeric matrix. Removal efficiencies of approximately 91% of sulfadiazine (SD) after 120 min under UV irradiation on the PPTD membrane are presented, corresponding to a significant increase of ∼20% compared with SD removal without UV irradiation.^578^

**Figure 34:**
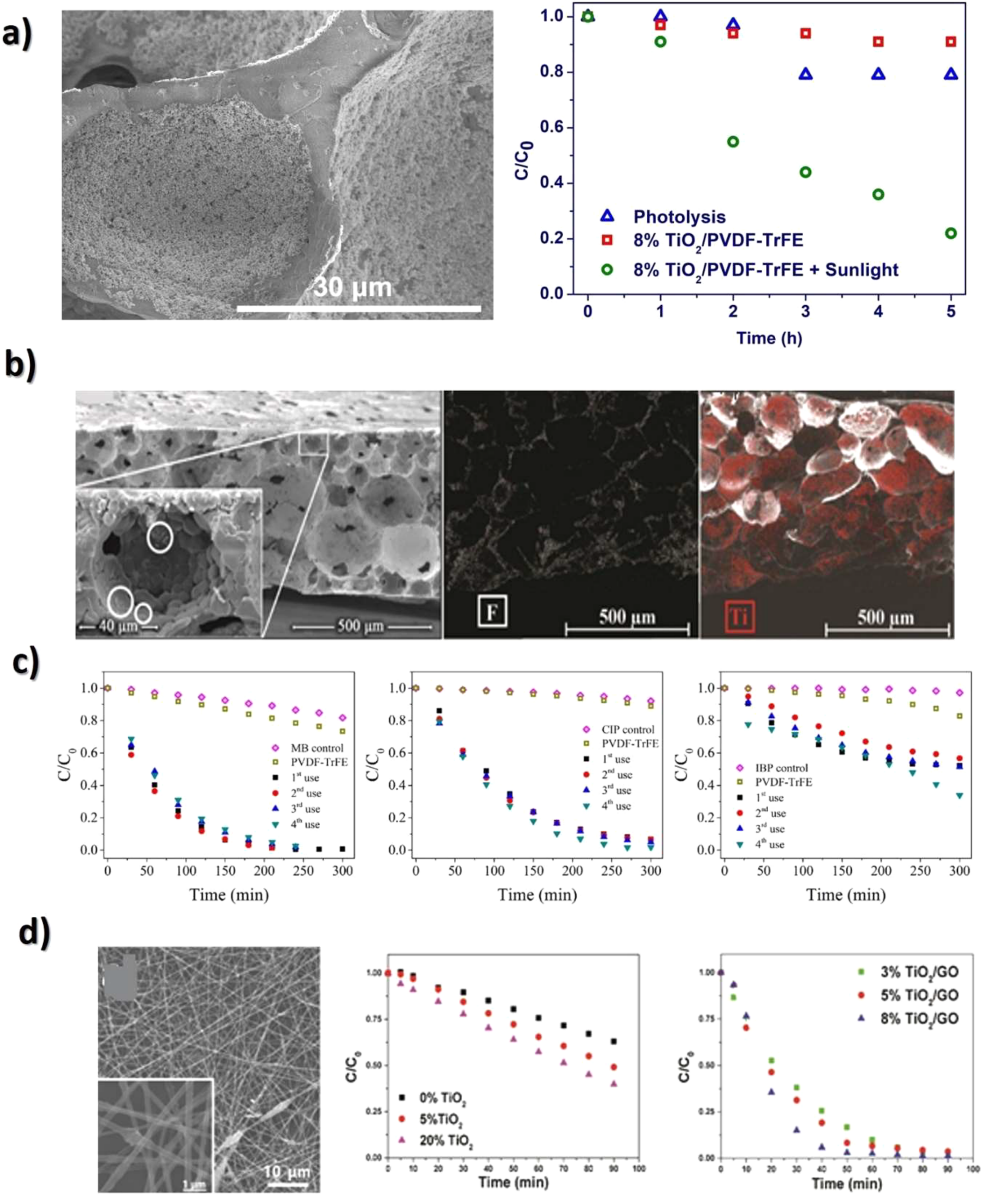
(a) Scanning electron microscopy (SEM) images of 8 wt % TiO_2_/poly(VDF-co-TrFE) membranes: cross-section and photocatalytic degradation of tartrazine (10 mg l^–1^) with the 8 wt % TiO_2_/poly(VDF-co-TrFE) nanocomposite, over 5 h of sunlight irradiation. Controls: irradiation of tartrazine solution without the nanocomposite (photolysis); the nanocomposite in tartrazine solution with no irradiation (adsorption). Reproduced with permission from ref (579). Copyright 2018 Elsevier. (b) Cross-section SEM images of 8 wt % TiO_2_/poly(VDF-co-TrFE) membranes with an inset showing a detail of an interconnected pore, with white circles for TiO_2_ nanoparticles and aggregates and EDX mapping image of the presence and distribution of F (fluorine) and Ti (red) in the poly(VDF-co-TrFE) matrix. Reproduced with permission from ref (580). Copyright 2019 Multidisciplinary Digital Publishing Institute. (c) Decreasing the content of methylene blue (MB), ciprofloxacin (CIP), and ibuprofenduring four cycles of photocatalytic treatment by 8% TiO_2_/poly(VDF-co-TrFE) sample under UV irradiation. Reproduced with permission from ref (581). Copyright 2019 Multidisciplinary Digital Publishing Institute. (d) SEM micrographs of poly(VDF-*co*-TrFE)/TiO_2_/GO electrospun membranes with 20% of GO/TiO_2_ with the inset corresponding to a higher magnification of the sample; photocatalytic degradation of MB under visible radiation for poly(VDF-co-TrFE) fiber membranes prepared with pure TiO_2_ and membranes prepared with TiO_2_/GO nanocomposite. Reproduced with permission from ref (582). Copyright 2016 Springer Nature.

Further, TiO_2_ nanoparticles have been immobilized into a poly(VDF-*co*-TrFE) matrix toward atrazine degradation in a solar photoreactor. An 8 wt % TiO_2_/poly(VDF-*co*-TrFE) nanocomposite was developed by solvent casting, obtaining a highly porous structure of ∼75% with interconnected pores (Figure 34a).

The obtained photocatalytic activity indicates a photoreactor degradation of ∼78% of tartrazine after 5 h of solar irradiation (Figure 34a). The nanocomposites’ reusability proved to be effective with just 10% efficiency loss after three uses, showing that the nanoparticles are efficiently attached to the porous structure, allowing remarkable efficiency and reusability.^579^ In a similar approach,^580^ the same nanocomposite was used to remediate oily wastewater. After 7 h of sunlight exposure, colorless oily wastewater was obtained, the photocatalytic results confirming the applicability for actual remediation strategies.

The SEM-EDX mapping images (Figure 34b) allow understanding that TiO_2_ nanoparticles are attached to the polymeric matrix and retained in the porous structure. Such information explains the remarkable reusability efficiency of these membranes, as most of the particles are kept inside the porous structure after use.

The TiO_2_/poly(VDF-*co*-TrFE) membranes were also tested against pharmaceuticals such as an antibiotic (CIP) and an anti-inflammatory (ibuprofen, IBP) besides MB).^581^ The materials were also reused four times, Figure 34c.

It was shown that the membrane is more efficient against MB and CIP, with 99 and 93% photocatalytic degradation, respectively. IBP reached 60% degradation, indicating the higher resilience of this pollutant. The membranes also show a remarkable reusability, as no substantial photocatalytic efficiency changes are identified after four uses, suggesting a suitable attachment of nanoparticles to the polymer substrate.

Poly(VDF-*co*-TrFE) membranes loaded with several amounts of TiO_2_ and TiO_2_/ GO (0, 3, 5, 8, and 20 wt %) (Figure 34d) have been produced by electrospinning, and the photocatalytic efficiency was assessed against MB, both under UV and visible radiation.^582^ The UV photocatalytic efficiencies are similar for the pristine TiO_2_ and the TiO_2_/GO nanocomposite (∼93% of MB degradation after 110 min). The results were different under visible radiation, with the 8% TiO_2_/GO nanocomposite completely degrading MB in 90 min (Figure 34d), compared to a 63% degradation for the sample containing the highest concentration of pristine TiO_2_ (20 wt %). The high surface area and porosity of the electrospun membranes and GO’s interesting electrical and structural properties allowed a notable performance.^582^

**Figure 35:**
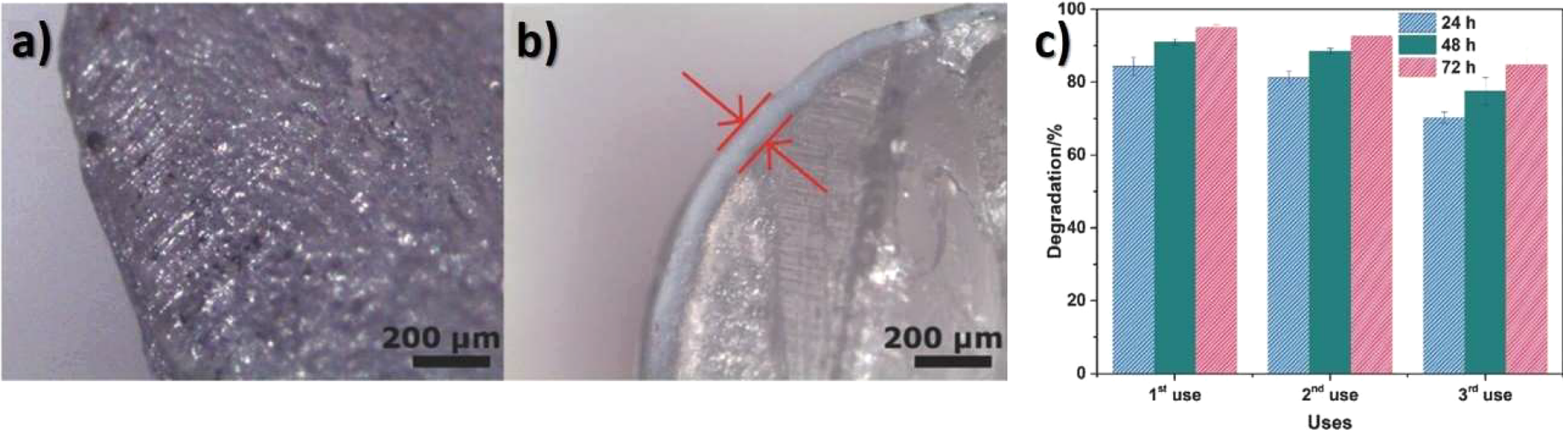
(a) Microscope images (amplification of 50×) of a commercial PMMA optical fiber; (b) coated with 50% w/w TiO_2_/PVDF by one dip and (c) Photocatalytic degradation versus the number of uses, of 5 mg·L^–1^ of CIP for 72 h under artificial sunlight using the 50 w/w % TiO_2_/PVDF-coated polymeric optical fibers. Reproduced with permission from ref (583). Copyright 2018 Wiley-VCH.

The easy processability of PVDF and its copolymers allowed production of a photocatalytic coating based on 50 wt % TiO_2_/PVDF over PMMA optical fibers, using the dip-coating technique,^583^ being possible to produce coatings with thicknesses ranging from 66 to 887 μm (Figure 35).

The mechanical stability of the PVDF coating was confirmed with a tape test. The sample with better mechanical properties achieved a CIP (5 mg.L^–1^) degradation of ∼95% under visible radiation (72 h). After three consecutive uses, the efficiency loss was approximately 11%, confirming the immobilization of TiO_2_ nanoparticles into the PVDF porous matrix. In this work, the optical fibers promote radiation transport and serve as a substrate to immobilize the TiO_2_ nanoparticles. The potential of this coating over PMMA optical fibers allows photocatalytic processes in aquatic environments where the sunlight does not penetrate (e.g., turbid, deep, or underground water).

Besides photocatalytic applications, other multifunctional materials based on PVDF and copolymers are being developed. Salazar et al. produced avant-garde materials with high multifunctionality and efficiency.^556^ Membranes were produced with different morphologies and different Ag/TiO_2_ nanoparticles loadings to evaluate norfloxacin degradation and antimicrobial activity. The obtained results indicate that increasing loadings of Ag/TiO_2_ nanoparticles increase the photocatalytic activity in both morphologies and under UV and visible radiation. The 10% Ag-TiO_2_/poly(VDF-*co*-HFP) solvent casting membrane degraded approximately 81% of norfloxacin in the solution and presented a reduced efficiency loss after three consecutive uses. Moreover, both morphologies induced 1.3 bacterial log reduction in *Escherichia coli*, indicating its antimicrobial and multifunctional nature.

A PVDF-based material with immobilized TiO_2_ and Ag nanoparticles has been applied as capacitive sensor with photocatalytic self-cleaning properties.^584^ A nonporous thin film was produced that assures good electric properties envisaging capacitive sensing applications. Still, it limits the photocatalytic self-cleaning effect exclusively to the thin film surface, where the MB solution is in contact with the TiO_2_ nanoparticles under UV irradiation.

MOFs are emergent materials also abundantly used in photocatalytic water treatments due to their highly porous structure and the possibility to functionalize them according to applications.^570^ A MIL-53/(Fe)/PVDF nanocomposite membrane was produced through a phase inversion method to assess tetracycline removal in a photocatalytic membrane reactor under UV radiation.^585^ The introduction of MIL-53(Fe) into the PVDF matrix improved surface morphology and increased membrane hydrophilicity (higher permeability), promoting the degradation of 93% of tetracycline in the water matrix.

#### 3.4.2 Adsorption

Similarly to photocatalytic processes, fluorinated polymers have also been employed in other water remediation processes, such as adsorption. In this case, the mechanical properties, porosity, and chemical stability of PVDF and its copolymers are particularly relevant. Because, in these cases, the used water flows are generally high, and unlike photocatalysis, the concentrations of fillers added to the membranes are typically higher.

**Figure 36:**
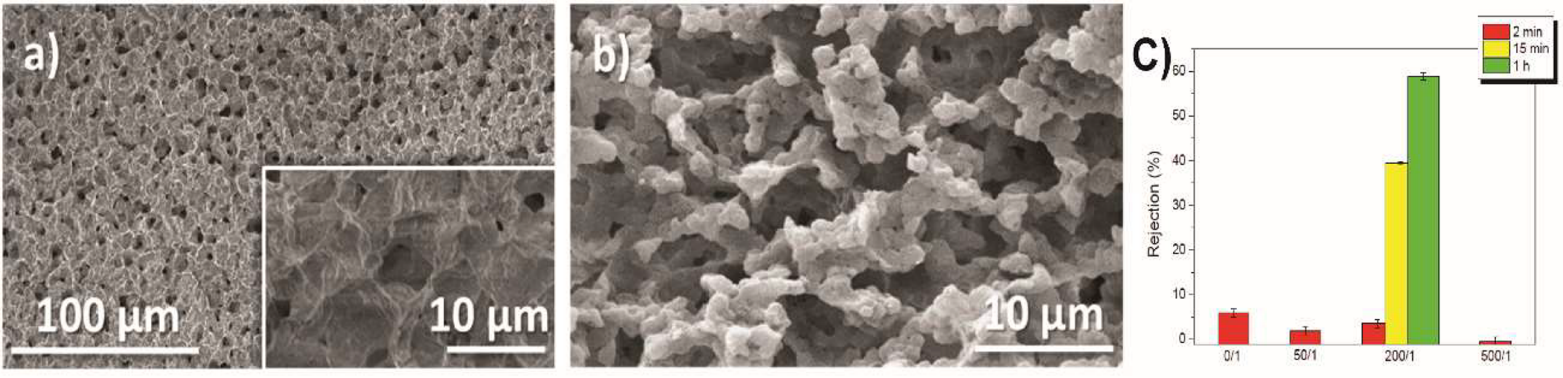
(a) Cross-section SEM micrographs of poly(VDF-*co*-HFP)/bayerite composite membranes with different nanofiller content 0/1, (b) 50/1, and (c) As rejection of the poly(VDF-*co*-HFP)/bayerite membranes. Reproduced with permission from ref (60). Copyright 2016 Elsevier.

In this scope, poly(VDF-*co*-HFP)/bayerite membranes have been prepared for arsenic (As) removal from water.^60^ These membranes possess interconnected porous structures with a degree of porosity from 65 to 75% (Figure 36a,b), a compressive strength higher than 100 kPa, and water flux between 65 and 215 Lh^–1^·m^2^.

The As adsorption assays reveal that using a bayrite/As ratio of 200/1 there was a rejection of ∼60% (40% of As removal) of As species present in the solution after 1 h of operation (Figure 36c). A novel microporous structure based on Fe_3_O_4_/poly(VDF-*co*-HFP) and Y_2_(CO_3_)_3_/poly(VDF-*co*-HFP) composites has been produced to remove As(V) and As(III) species from contaminated waters.^562^ The results indicate adsorption capacities around 93 mg·g^–1^ for As(III) and 137 mg·g^–1^ for As(V) removal employing the Fe_3_O_4_/poly(VDF-*co*-HFP) membrane. Additionally, this work contemplated reusability tests that resulted in an efficiency loss of 13% of the Fe_3_O_4_/poly(VDF-*co*-HFP) nanocomposite membrane and increased efficiency by around 6% with the yttrium carbonate Y_2_(CO_3_)_3_/poly(VDF-*co*-HFP) membrane, which confirms its stability after five uses.

**Figure 37:**
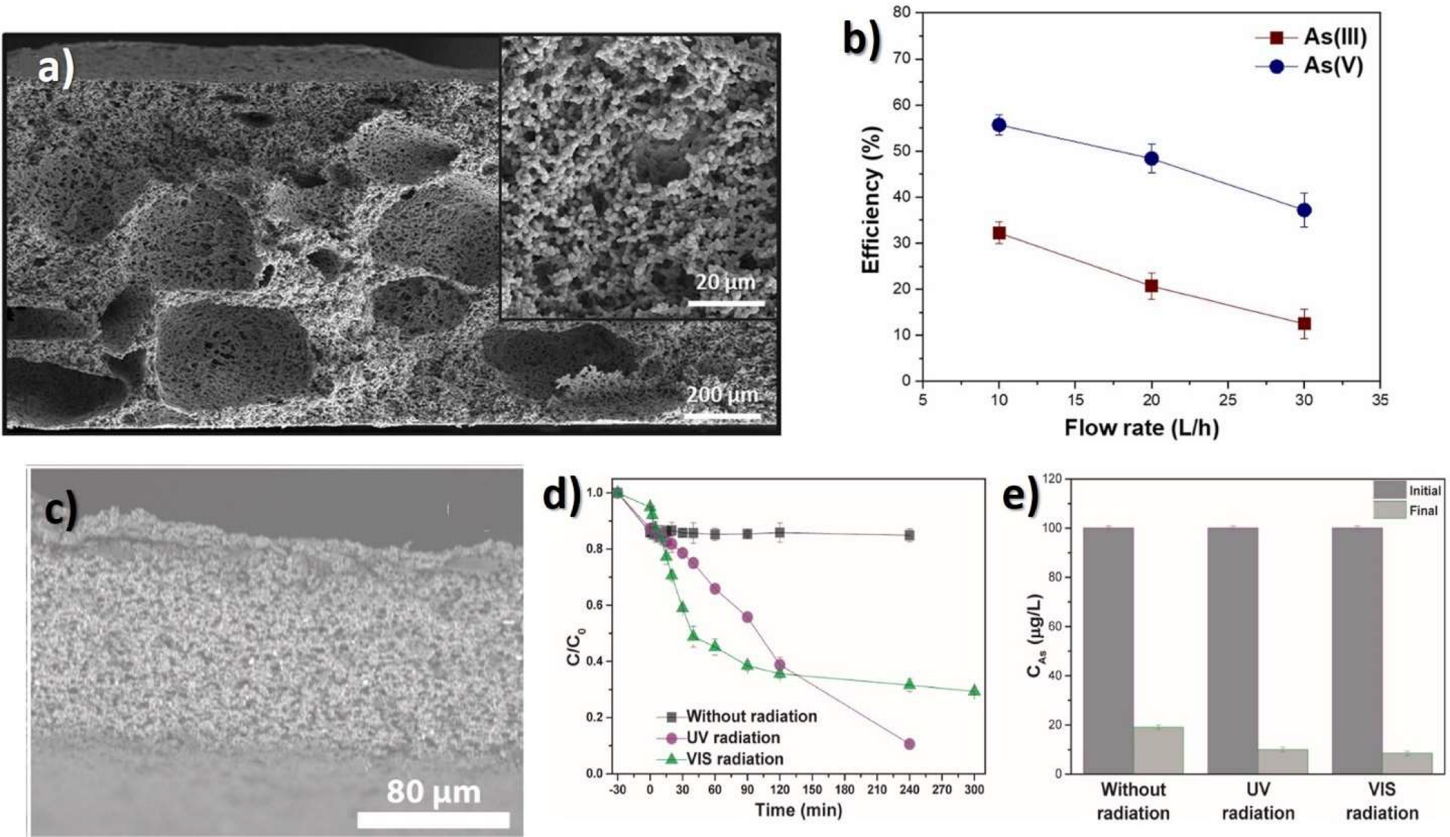
(a) SEM image of poly(VDF-*co*-HFP) filter membranes; (b) dependence of flow rate on adsorption efficiency ([As]_i_ = 5 mg·L^–1^; contact time: 24 h; pH = 7). Reproduced with permission from ref (586). Copyright 2022 Elsevier. (c) SEM image of the 10% Au/TiO_2_/Y_2_(CO_3_)_3_/poly(VDF-*co*-HFP) membrane used in removal of (d) NOR by photocatalytic degradation and e) As(V) adsorption in a simultaneous multifunctional assay ([NOR] = 5 mg·L^–1^; [As] = 100 μg·L^–1^; pH = 7; time: 240 min of UV and 300 min of VIS). Reproduced with permission from ref (563). Copyright 2022 Elsevier.

Still, in the adsorption treatment, Salazar et al. manufactured a poly(VDF-*co*-HFP) membrane containing yttrium carbonate (Y_2_(CO_3_)_3_) and Fe_3_O_4_ to be used in a filtration reactor to remove As species from different water matrixes.^586^ This membrane is particularly interesting because it combines two processes to produce porous structures: salt leaching and TIPS. Such a highly porous structure (Figure 37a) allowed employment of this material in a filtration reactor that requires high porosity to allow water permeability. The prepared filters achieved maximum adsorption capacities of 101.9 and 212.8 mg·g^–1^ for As(III) and As(V), respectively. Additionally, the process was scaled up to a reactor with treated effluent, allowing removal of As species from effluents in the presence of interfering contaminants with efficiencies of 21.9 and 51.8% for As(III) and As(V), respectively (flow rate 20 L·h^–1^, pH = 7, 24 h of contact) (Figure 37b).

Adsorptive membranes can be multifunctional, exhibiting more than one functional application. In this regard, a poly(VDF-*co*-HFP) membrane has been developed with sorbent and photocatalytic properties. Two different active materials were added, Au/TiO_2_ and Y_2_(CO_3_)_3_ nanoparticles (Figure 37c), to degrade norfloxacin and adsorb As, respectively. In the multifunctional assay, the hybrid membrane with 10 wt % of Au/TiO_2_ and 10 wt % of Y_2_(CO_3_)_3_ degraded ∼61% under UV radiation. Regarding the same assay under VIS radiation, 70% of the antibiotic was eliminated (Figure 37d). Regarding adsorption on the multifunctional assay, 81%, 90%, and 93% of As removal efficiencies were obtained after 240 min without radiation, under UV radiation, and VIS radiation, respectively (Figure 37e). The results highlight the relevance of the poly(VDF-*co*-HFP) processability, allowing the incorporation of two different active materials and its robust porous structure.

**Figure 38:**
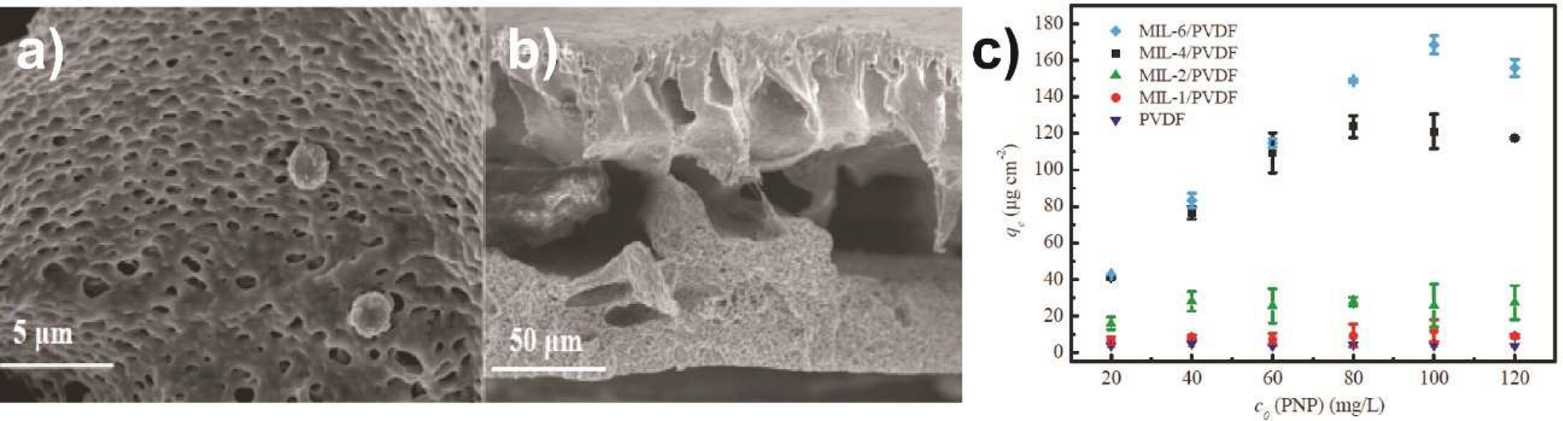
SEM images of MIL-1/PVDF (a,b); adsorption isotherms for p-nitrophenol (PNP) (c) on MIL-68(Al)/PVDF hybrid membranes with different contents of MIL-68(Al), which are 1 wt %, 2 wt %, 4 wt %, and 6 wt %, respectively. Reproduced with permission from ref (558). Copyright 2019 Elsevier.

In a similar context, a novel type of MOF/polymer matrix, MIL-68(Al)/PVDF (Figure 38a,b), was produced through solvent casting to test its adsorptive properties.^558^

The adsorptive properties of these new membranes were tested against *p*-nitrophenol (PNP), and all MIL-68(Al)/PVDF samples showed higher adsorption compared to the pristine PVDF membrane. The maximum adsorption capacity was 183.49 μg·cm^–2^_,_ almost 49.9 higher than pristine PVDF (Figure 38c).

The robustness of PVDF and copolymer membranes allows for reusability, which is very important to make the water purification processes less costly and more sustainable. The work developed by Queirós et al. focused on chromium adsorption (Cr(VI)) and the reusability of the produced membranes.^570^ The work employed Al(OH)_3_ and two metal–organic frameworks, namely the MIL-88-B(Fe) and UiO-66-NH2 Cr(VI) sorbents immobilized into a poly(VDF-*co*-HFP) matrix to remove Cr(VI) from water and real treated wastewater. It was shown that the membranes with Al(OH)_3_, MIL-88-B(Fe), and UiO-66-NH_2_ could retain 12, 62, and 97% of Cr in solution (5 mg.L^–1^), respectively, after 24 h of contact. Reusability studies were performed after composite membrane reactivation (stirring for 4 h with a 1 M HCl solution and washing with ultrapure water for 2 h). After three cycles, an efficiency loss of approximately 10% was observed, confirming the particle’s efficient attachment to the polymeric matrix and its suitability to reuse.

Similarly, a multifunctional membrane has been produced through TIPS, loaded with MIL-53 (Fe) for adsorption and catalytic oxidation.^587^ MB was used as a contaminant model to assess the produced PVDF membranes’ catalytic oxidation and adsorptive performance. Moreover, the microfiltration properties of the membrane were assessed using bovine serum albumin (BSA). The multifunctional membrane could remove over 75% of MB and retain 82–86% of BSA. Tests were also performed in natural wastewater, confirming the ability of this membrane to remove contaminants in a complex water matrix.

It has been shown in this section that water contamination is an increasingly worrying and complex problem to solve by humankind. One of the significant problems in this framework are the emergent contaminants (e.g., pharmaceuticals, pesticides, heavy metals), which can persist in water even after undergoing classical physicochemical or biological treatments and can be toxic even at low concentrations (ng to μg). In this context, novel materials and devices are necessary to address this massive variety of persistent contaminants. Water remediation techniques such as photocatalysis and adsorption have been widely applied due to their high efficiency and practicability. To employ these techniques, polymers such as PVDF and copolymers possess the perfect properties once it allows a large variety of processing conditions and morphology and it is mechanical, thermal and UV resistant. As stated in detail above, the production of polymeric membranes loaded with active materials of different types (e.g., TiO_2_ nanoparticles and MOFs) and controlling the porous microstructure and surface properties is essential to obtain an efficient removal of the contaminant. In the scope of photocatalysis, it is noted that TiO_2_ still is the most used photocatalyst despite new active materials’ arrival, whereas MOFs are emergent materials that present high flexibility in terms of design, allowing them to address complex water contamination problems in a customizable way.

Additionally, multifunctional materials are a noticeable trend, which is necessary as real contaminated water possesses a myriad of different contaminants to be addressed in different ways. In short, PVDF and copolymers reveal an outstanding processability that allows producing all types of structures in combination with a large variety of active materials and physicochemical functionalization. The polymeric matrix partly endows the presented materials’ efficiency, robustness, and reusability.

### 3.5 Microfluidics and Portable Analytical Devices

Microfluidics is the science of controlling and manipulating tiny amount of fluids, usually in the range of pico- to microliters, in networks of channels with sizes from ten to hundreds of micrometers.^588^ This discipline is perceived as a powerful tool for life science research and biotechnology by taking advantage of its small size, low volume sample consumption, user-friendly design, fast analysis, as well as the potential to carry out several analyses in parallel.^589,590^ These properties open the possibility of performing *in situ* and real-time measurements but depend on the design and integration of specific tools in order to allow multiple operational steps, including sampling, mixing, separation, isolation, detection, and analysis, among others.^591,592^ These tools must be simple, cost-effective, compact, easy to control, and simple to manufacture and assemble in the microfluidic platform.^593^ As previously described, PVDF-based polymers feature the highest piezoelectric coefficients among polymers, which together with its tunable structure, easy processing, low density, flexibility, and biocompatibility have been increasingly integrated or coupled to microfluidic platforms as piezoelectric sensors and actuators for diverse applications. In addition, PVDF-based materials have been used as passive membranes in microfluidic platforms, as described in the following.

#### 3.5.1 Poly(vinylidene fluoride)-Based Sensors

The development and integration of sensors in microfluidic systems play a key role to obtain low-cost, innovative, and portable platforms with a wide range of functionalities required for advanced applications.^594,595^ In this sense, piezoelectric materials that respond to both electrical and/or mechanical stimuli are suitable candidates for sensor applications. Within the class of piezoelectric materials, polymers such as PVDF and its copolymers represent an excellent alternative to common rigid piezoceramics due to their flexibility, versatility, facile processing, easy integration, and low cost. Moreover, their lower density usually results in a better sensitivity than quartz or PZT. In the following, relevant studies on designing and manufacturing PVDF-based sensors and their integration, testing, and characterization on microfluidic platforms are presented.

**Figure 39:**
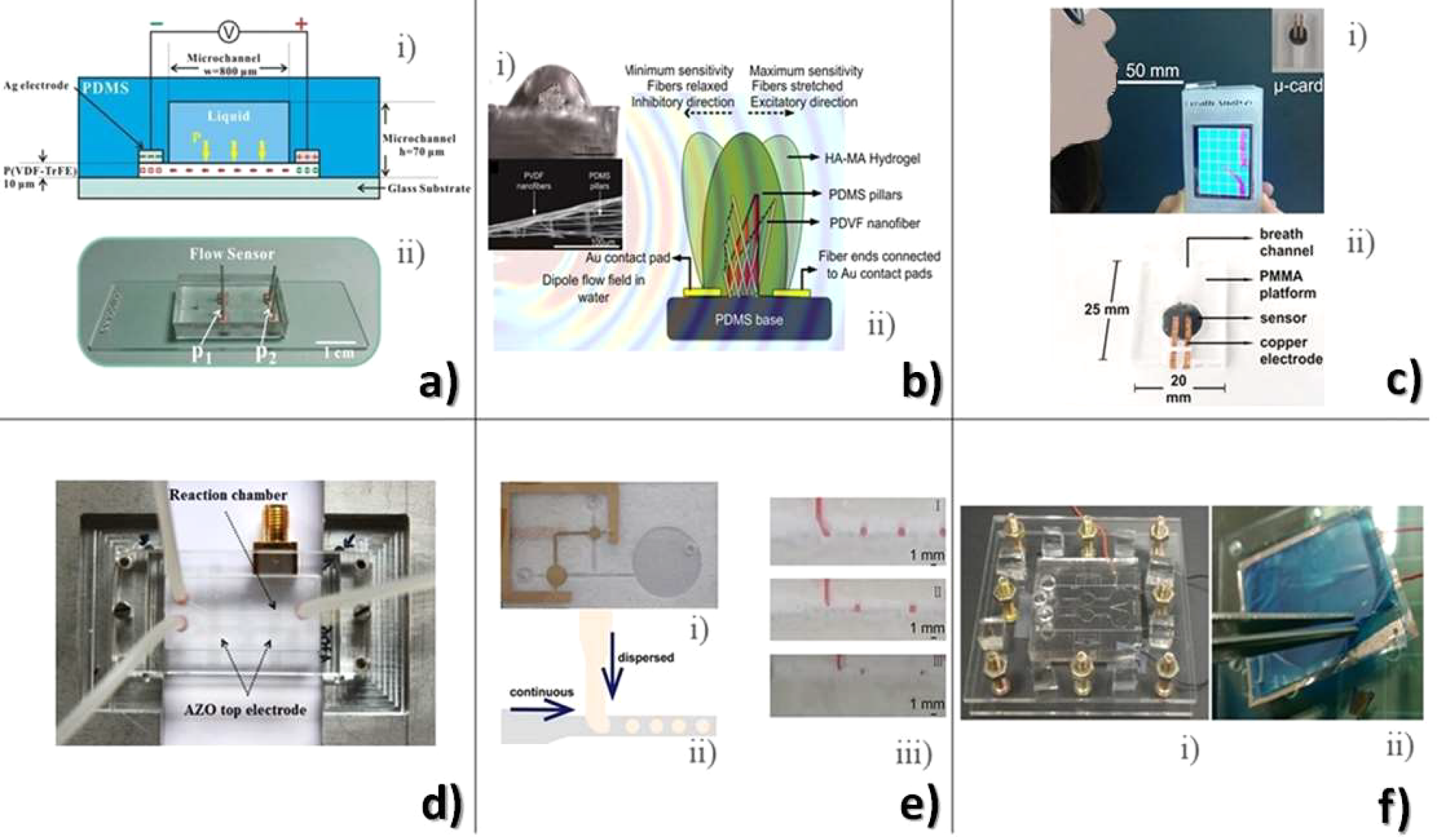
(a) (i) Schematic and (ii) photograph of the microfluidic flow sensor based on aligned piezoelectric poly(VDF-*co*-TrFE) nanofibers (p_1_ and p_2_: sensing parts). Reproduced with permission from ref (599). Copyright 2015 American Institute of Physics Publishing. (b) (i) Microscopic side-view of the artificial flow sensor showing the hydrogel cupula and the PDMS pillars, (ii) schematic showing the flow sensing mechanism in the presence of an oscillating dipole. Reproduced with permission from ref (600). Copyright 2016 Springer Nature. (c) Representation of a volatilome analyzer, (ii) photograph of the integrated μ-card. Reproduced with permission from ref (601). Copyright 2018 American Chemical Society. (d) Photograph of a microfluidic system with the patterned piezoelectric poly(VDF-*co*-TrFE) actuator placed underneath. Reproduced with permission from ref (57). Copyright 2014 Royal Society of Chemistry. (e) (i) Photograph of a droplet generating microfluidic system, (ii) schematic of the T-junction, (iii) photographs of droplet generator at (I) 5 Hz, 1.2 kV, (II) 5 Hz, 1.5 kV, and (III) 5 Hz, 2.0 kV. Reproduced with permission from ref (602). Copyright 2017 Springer Nature. (f) Photographs of (i) the dual light-activated optopiezoelectric PVDF pumps, (ii) dip-coated TiOPc/PVB photoconductive layer. Reproduced with permission from ref (603). Copyright 2017 Institute of Physics Publishing.

Microdiaphragm arrays based on piezoelectric poly(VDF-*co*-TrFE) copolymer with integrated microfluidic chip were designed and implemented for high-performance protein immunosensors. The diaphragm was fabricated using mold-transfer and hot-embossing techniques, allowing high throughput and reproducibility.^596^ The intrinsic hydrophobic surface of poly(VDF-*co*-TrFE) acts as a natural bioreceptor to bind proteins of interest, which greatly simplifies the sensor preparation by eliminating the self-assembled monolayers (SAM) formation and pretreatment steps. The poly(VDF-*co*-TrFE) diaphragm with 32 μm thickness and 1 mm of diameter (fundamental resonant frequency at 39.68 MHz) demonstrated good linearity for BSA concentrations from 1 to 1000 μg·mL^–1^. A sensorized microfluidic end-effector system has been also designed, calibrated, and implemented by means of a PTFE microtube for handling and deposition of nano- and microentities.^597^ The system integrates a highly sensitive PVDF beam sensing buffer between a DC microdiaphragm pump and a micropipette to provide controlled micro force and flow rate for precise microfluidic handling, droplet control, and manipulation. The PVDF sensing buffer consists of a double-end fixed PVDF sensing beam strip with one PVDF layer and one polyester layer. The microfluidic system showed a deposition success rate of CNTs close to 80%, demonstrating its potential for the manufacture of CNT-based sensors and detectors and ultimately for the manufacture of nano- and microsensors and devices. A capacitive-type microfluidic flow sensor based on piezoelectric PVDF was fabricated using microelectromechanical systems (MEMS) technology to detect flow rates and impulse pressure signals.^598^ The flow rate is monitored by the frequency amplitude response that differs with flow rate variations as the frequency signal is coupled to the resistance and capacitance connections of the PVDF equivalent circuit. Impulse pressure is measured using a charge amplifier. This piezoelectric microfluidic platform takes advantage of the flexibility of PVDF, which is difficult to obtain with piezoceramics, and polydimethylsiloxane (PDMS) to fabricate soft microfluidic sensors, proving to be effective in detecting flow rate at high pressure. A highly sensitive PDMS microfluidic flow sensor was also fabricated by integrating a 10 μm thick electrospun aligned piezoelectric poly(VDF-*co*-TrFE) nanofiber film (Figure 39a).^599^

The flow sensor can linearly measure low flow rates ranging from 13 to 301 μL·h^–1^ with a sensitivity of 0.36 mV per 1 μL·h^–1^. Additionally, the system could measure in dynamic flow the viscosity of ethylene glycol aqueous solution ranging from 1 to 16.1 mPa·s at 25 °C. Another electrospun aligned poly(VDF-*co*-TrFE) fiber sensor was interfaced with a flexible plastic to create a NG for energy harvesting devices and self-powered mechanical systems with potential application in robotic and microfluidic platforms.^604^ The NG demonstrated ability to produce average voltage peak signals of ± 0.4 V when deformed by 8 mN of cantilever pressure at both 2 and 3 Hz. The piezoelectric response did not change after platform sterilization but dampened when placed in cell culture media. A similar self-powered microfluidic sensor that can simultaneously harvest the mechanical energy of fluids and monitor their properties was designed and fabricated.^605^ The NG is composed of flexible electrospun piezoelectric PVDF nanofibers integrated into a PDMS microfluidic system. The platform generates open-circuit high output voltage up to 1.8 V when a droplet of water flow past the suspended PVDF nanofibers. Moreover, the platform features self-powered sensing behavior with a decreased voltage amplitude with increasing input pressure and liquid viscosity, which demonstrates its potential as a self-powered microfluidic sensor for *in situ* monitoring of viscosity and pressure. A miniaturized and biocompatible flow sensor that closely mimics the structural architecture of the hair cell bundles in fish was fabricated (Figure 39b).^600^ It is constituted by arrays of PDMS micropillars with graded heights connected to electrospun piezoelectric PVDF nanofibers that act as biological tip links and elicit electric charges proportional to the stress induced in the fibers. A hydrogel was used to encapsulated the sensor so that the resulting cupula bends in response to an external flow and causes, therefore, the sensor to bend as well. Water flow sensing assays performed using a dipole stimulus showed sensitivity and threshold detection limit of 300 mV·(m·s^–1^)^−1^ and of 8 μm·s^–1^, respectively. Biomedical and microfluidic applications can take advantage of this self-powered, sensitive, and flexible sensor. A sensing microfluidic platform based on electrospun PVDF, PS, and PMMA nanofibers incorporated with MWCNTs was fabricated for the detection of acetone and toluene, which are target volatilomes associated with diabetes and lung cancer.^601^ This hand-held volatilome analyzer (Figure 39c) takes advantage of the varying solubility of these three polymer nanofibers in contact with acetone and toluene. This noninvasive platform test responds selectively to acetone in a concentration range between 35 ppb and 3 ppm and toluene between 1 ppb and 10 ppm in exhaled breath. Another interesting study combined a gecko-inspired nanotentacle skin integrated with a microfluidic system and a PVDF-based piezoelectric NG for multisensing.^606^ The system was successfully tested for the simultaneous detection of three sweat indicators, namely pH, lactic acid and urea, and also wrist pulse, demonstrating its potential for various applications, such as personal care, human–machine interaction, and artificial intelligence. PVDF Al-coated microcantilever beams, considered as a mechanical transducer, were developed to detect the biomechanical interaction of the adenosine triphosphate (ATP) molecules and the heat shock protein 90 (HSP90).^607^ The proposed technique demonstrates higher sensitivity and shorter response time (5 times lower) than similar methods such as piezoresistive methods used for studying biomolecular interaction of antibodies and antigen. Thus, this biosensor system has the potential for the development of advanced microfluidic biosensors.

In addition to the applicability of the piezoelectricity of PVDF-based polymers, the pyroelectric property has also been used for temperature variation monitoring in microfluidic systems.^608^ The microfluidic platform consists on a micromilled 1.5 mm thick PMMA bulk sealed with a 24 μm thick metalized and poled PVDF sheet. The development of the polymer pyroelectric sensor represents an effective solution in terms of ease of integration, sensitivity, and speed when prompt and accurate temperature monitoring is required. The pyroelectric response of the platform demonstrated to be suitable for most biological applications, with a better responsivity between 3.2 and 200 ms, even with a large temperature gradient. A modified setup uses a 28 μm thick piezoelectric PVDF film integrated into a PMMA microfluidic system to fabricate a high-performance and cost-effective temperature sensor that allows rapid monitoring of the localized temperature of biological fluids.^168^ In this approach, an infrared laser is combined with the pyroelectric PVDF film, which stimulates an active element located on the top of the microfluidic channel and allows measuring of the absolute temperature without the use of an adjunctive reference temperature sensor. In another study, a preamplifier developed in a 180 nm CMOS process converts the charge generated by a pyroelectric 20 μm thick PVDF transducer into a voltage signal, proving a measurement of the temperature variation in biological fluids.^609^ The proposed concept can provide a conversion gain of 0.8 mV·pC^–1^ while consuming just 2.1 μW of power. With a total area of 0.038 mm^2^, the developed charge-sensitive preamplifier is suitable for implementation in a lab-on-a-chip system.

#### 3.5.2 Poly(vinylidene fluoride)-Based Actuators

Controlled fluid transport and rapid analysis results are essential to improve the efficiency of microfluidic platforms and their applicability.^610^ Thus, the automatic and controllable transport and manipulation of fluids and their mixture in the shortest possible time are key aspects that must be considered when developing microfluidic platforms.^611^ Microfluidic technology is associated with resistance to flow at a micrometer scale and difficulties in the laminar flow regime.^612^ To overcome these limitations, large efforts are being made to design and fabricate mixing and pumping systems of suitable size for integration in microfluidic devices. Some solutions described in the literature use MEMS, such as microvalves and micropumps, to define fluid transport.^613^ Although efficient, they can be challenging to fabricate and integrate into a miniaturized chip and may cause damage when sensitive fluids are used, such as those containing cells.^612,614^ Other methods rely on long and complex channel geometries to favor passive mixing, which is often associated with long transit and mixing time, and they depend on the diffusion coefficients of the fluids involved.^615,616^ A suitable approach comes from piezoelectric polymer actuators that can be easily integrated into microfluidic systems and promote the manipulation of entities and fluids by converting electrical into mechanical energy. Although piezoelectric PVDF-based polymers feature a lower electromechanical coupling coefficient that piezoceramics such as PZT, their lower acoustic impedance ensure a low reflection coefficient between the piezoelectric material and the propagation medium. Moreover, while ceramics break easily and feature hard and dense structures, PVDF-based polymers are flexible, with low density, and easily produced into thin films, facilitating their integration.^617^ Relevant examples of microfluidic platforms that integrate piezoelectric PVDF-based actuators are presented in the following.

A fully integrated SU-8 disposable microfluidic platform that integrates a piezoelectric PVDF polymer film was developed for clinical diagnosis.^618^ The platform takes advantage of the acoustic waves generated by a 110 μm piezoelectric PVDF film located underneath the reaction chambers to improve the mixing and reaction time of fluids, a phenomenon called acoustic streaming. The results demonstrated that applying an electrical signal at the resonance frequency to the electrodes of the piezoelectric film showed better results in terms of mixing time. In addition, the heating generated by the piezoelectric film also contributes to the reduction of the reaction time when endothermic reactions are involved. An optimized system was developed for the same purpose.^57^ In this case, the piezoelectric transducer is based on a 25 μm thick poly(VDF-*co*-TrFE) film with aluminum doped zinc oxide (AZO) electrodes fabricated by a layer-by-layer deposition approach so that the entire system is transparent in the visible light spectrum^71,619^ and allows optical detection (Figure 39d). The piezoelectric transducer, featuring a piezoelectric |*d*_33_| coefficient of 34 pC·N^–1^, was integrated underneath a PDMS microfluidic system and actuated with a peak-to-peak voltage amplitude of 10 V and a frequency of 48 MHz. In both cases, the reaction time for quantifying two clinically relevant analytes, uric acid and nitrite in urine, was reduced by 23% and 32%, respectively, compared with the reaction time achieved only by diffusion. In other studies, all inkjet-printed piezoelectric polymer actuators based on poly(VDF-*co*-TrFE) and Ag electrodes were fabricated on a PET substrate.^620^ The actuators feature piezoelectric *d*_31_ coefficients in the range of 7–10 pm.V^–1^, allowing the generation of significant actuator deflections and pump rates up to 130 μL·min^–1^ in microfluidic applications.^621^ A micropump based on a 28 μm thick commercial piezoelectric PVDF film was integrated into a PMMA microfluidic system and allowed for precise control of water flow rate in the range of 0–300 μL.min^–1^ by tuning the applied electrical signal voltage and frequency.^602^ The system was tested for the generation of droplets by integrating two piezoelectric PVDF pumps into one T-junction microfluidic system (Figure 39e). Controlled droplet size was achieved by tuning the applied electrical voltage. At low voltage, a nongassing miniature electroosmotic pump was also developed by assembling poly(2-ethyl aniline) (EPANI)–Prussian blue nanocomposite electrode and commercially available hydrophilic PVDF membranes (area of 0.28 cm^2^).^622^ Although linear with the applied voltage, the flow rate also depends on the electrode composition. At 5 V, flow rates were increased from 187.41 to 95.47 μL·min^–1^·cm^–2^ as the weight fraction of 2-ethyl aniline was increased.

The maximum stall pressure at zero flow for the best-developed pump was 1.2 KPa at 2 V. These characteristics make them promising for various microfluidic applications. A wearable, nozzle-diffuser microfluidic pump was designed, fabricated, and tested.^623^ The system is based on integrating core–shell structured Al_2_O_3_@CNTs nanofillers in poly(VDF-*co*-TrFE) to increase the induced strain. High controllability of the fluidic process is obtained with flow rates ranging from 13 to 135 μL·min^–1^. Dual light-activated optopiezoelectic microfluidic pumps based on a 9 μm thick commercial piezoelectric PVDF polymer film coated with a layer of titanyl phthalocyanine (TiOPc) photoconductive coating and ITO transparent electrode were fabricated and tested (Figure 39f).^603,624^ They feature the advantage of being selectively activated and controlled, both spatially and temporally, by a single masked light source and voltage source, allowing operation of various micropumps independently at the same time. This approach allows to reduce the complexity and size of the driving element of a microfluidic system. Volume flow rates of 28.89 μm·s^–1^ were reached, optimizing the synchronization of the operating frequency of the light source and the driving voltage. Other optopiezoelectric system was fabricated and optimized to work as optical control valve array for digital microfluidic applications.^625^ The system is based on a piezoelectric poly(VDF-*co*-TrFE) film deposited by spin-coating and coated with a thin TiOPc electrode layer.

#### 3.5.3 Poly(vinylidene fluoride)-Based Membranes

In addition to its use as active sensors and actuators in microfluidic platforms, PVDF-based materials have also been applied as passive membranes in microfluidic systems for diverse applications, taking advantage of their easy processing, hydrophobicity, and mechanical resistance.

PVDF is a material commonly employed for the adsorption of proteins in Western blot analysis that follows protein acrylamine gel electrophoresis. Thus, the development of nanofibrous PVDF membranes by electrospinning has the ability to improve protein adsorption in such assays because of the increase of its specific surface area. This approach has been used in a PDMS microfluidic platform for cross-array immunoassays that can simultaneously detect protein–protein interactions.^626^ The processed electrospun PVDF membranes feature eight times more capacity for adsorbing proteins than conventional track-etched polycarbonate (TEPC). The performance of PVDF substrates (Durapore PVDF, 5 μm pore size) in electrokinetic microfluidic-based analytical devices was also studied by developing a laminate glass-PDMS-PVDF-PDMS-glass laminate structure. For that, 1 mm thick borosilicate glass microscope slides, 500–700 μm thick PDMS sheets, and PVDF microchannels with lengths ranging from 1–4 cm cut using a direct laser writing instrument were used.^627^ PVDF demonstrates the ability for electrophoretic separations of three amino acids mixture. It was also reported the functionalization of commercial PVDF membranes and their integration in a microfluidic platform for the capture and purification of porcine carboxylesterase and porcine LDH.^628^ PVDF membranes were also coated with carbon nanodots for the development of a POC photoluminescence membrane strip for the quantification of DA.^629^ A portable POC immunosensing platform was also developed for sensitive detection of prostate-specific antigen (PSA) in biological fluids by coupling a digital multimeter readout with a flexible photosensitive pressure sensor made of photoactive methylammonium lead iodide (CH_3_NH_3_PbI_3_) and PVDF.^630^ Under optimum conditions, the digital multimeter immunoassay featured good analytical properties toward PSA within the dynamic linear range of 0.02–50 ng·mL^–1^ at a detection limit of 12.6 pg·mL^–1^. A POC electrochemical immunosensor that employed PVDF microparticles coated with streptavidin and AuNPs was developed for the highly sensitive detection of human thyroglobulin.^631^ Linear response from 2.0 to 10.0 ng·mL^–1^ with *R*^2^ of 0.985 was obtained with detection limits of 0.015 ng·mL^–1^. Porous hydrophobic flat and microstructured PVDF membranes fabricated by immersion precipitation and phase separation micromolding techniques, respectively, and integrated in a glass microfluidic platform to allow an efficient supply of gases into liquids or degassing of fluids within confined microchannels.^632^ O_2_ transport simulations and experiments were performed and shown that microstructured PVDF membranes enhance mass transport rates and exceed the performance of flat PVDF membranes. Another study reported a new type of wound dressing based on hybrid microfibers of konjac glucomannan and PVDF, having hydrophilic and hydrophobic segments, developed via microfluidic spinning.^633^ While PVDF is hydrophobic and allows good tensile strength, easy processing, and drug release properties, konjac glucomannan is a natural polysaccharide featuring hydrophilic properties, high drug-load efficiencies, good biocompatibility, and good biodegradability. Drug release tests were performed by loading the microfibers with enrofloxacin. The results show sustained drug release performance for 13 days, excellent heat resistance, antibacterial activity against *E. coli* and *Staphylococcus aureus*, and promotion of wound healing. An array of four PVDF membranes impregnated with cationic poly(3-alkoxy-4-methylthiophene) (PMNT) as an optical indicator was also developed and evaluated for the colorimetric detection of lung cancer biomarker microRNA (mir21) and hepatitis B virus DNA biomarker (HBV-DNA).^634^ Linear response for mir21 and HBV-DNA from 1 nM to 10 μM was obtained with a limit of detection of 0.6 nM and 2 nM in distilled water and plasma, respectively. Moreover, a logic gate system was proposed for discrimination of mir21 and HBV-DNA using the colorimetric assay response as inputs. This result offers a promising approach for colorimetric profiling of nucleic acids and thus POC diagnosis. PVDF membranes were also assembled with cationic poly[*N*,*N*,*N*-triethyl-3-((4-methylthiophen-3-yl)oxy)propan-1-aminium bromide] to form a conjugated polyelectrolytes that along with the use of a smartphone allows the precise quantification of nucleic acid assays concentrations in a range down to 1 mM.^635^ The obtained system could be used for POC colorimetric nucleic acids assays in complex matrices without the need of expensive and sophisticated software and instrumentation. In another study, PVDF membranes were used to increase the colorimetric signal (color intensity) and thus enhance the performance of a stopped 3,3′,5,5′-tetramethylbenzidine (TMB) colorimetric signal in paper-based biosensors.^636^ In fact, stopping the reaction of a colorimetric assay if often used in microfluidic systems to amplify and stabilize the colorimetric signal for detection, turning this approach extremely useful for this kind of application. Substrate-free electrospun PVDF and PVDF/zeolite A membranes were also produced using an optimized circle electrode collector with higher productivity and uniformity than traditional electrospinning process using flat collector.^637^ These membranes were successfully assembled and tested for humidity blocking, having also potential to be used and integrated into organ-on-a-chip, biochemical sensors, and microfluidic analytical platforms.

**Figure 40:**
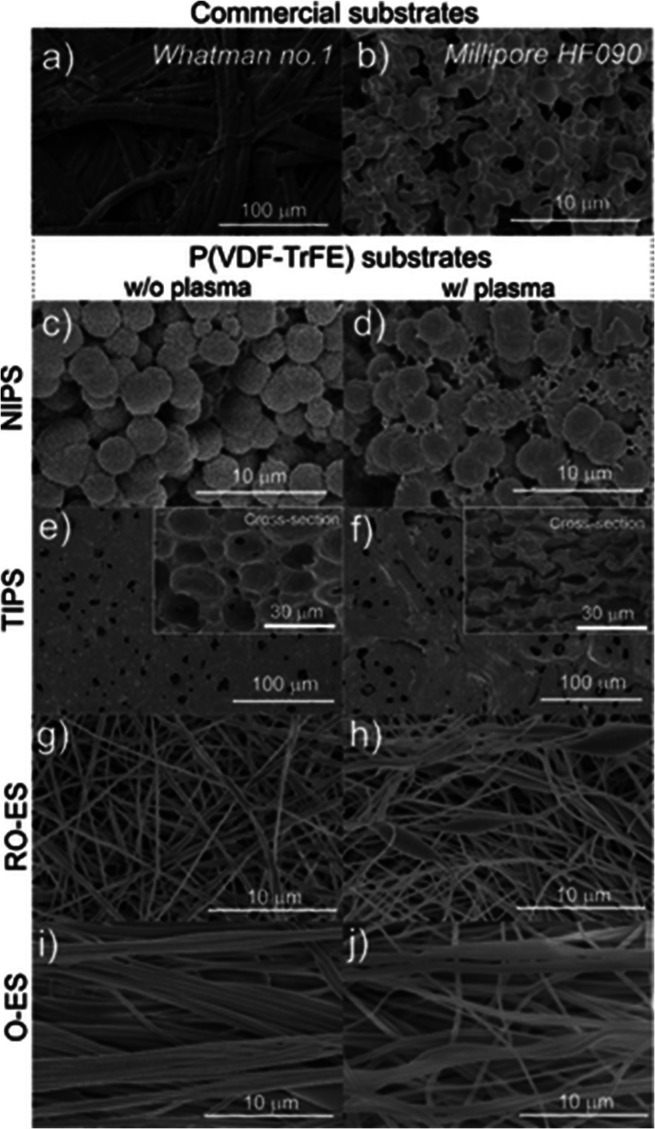
(a) Representative SEM images of the processed poly(VDF-*co*-TrFE) membranes before and after O_2_ plasma treatment (to tailor the hydrophobicity) together with commercial Whatman no. 1 and Millipore HF090 substrates, for comparison. Reproduced with permission from ref (61). Copyright 2021 American Chemical Society.

Last but not least, novel materials have been developed and tested as microfluidic substrates for the manufacture of portable analytical systems (or POC) as a potential pathway to complement the limited range of commercially available microfluidic substrates used for the manufacture of paper-based analytical devices (μPADs) mainly based on cellulose.^638^ The suitability, advantages, and application possibilities of microfluidic substrates based on poly(VDF-*co*-TrFE) membranes with tailored morphology, including spherulitic, porous, and randomly oriented and oriented fibers, were evaluated and compared with commercial paper substrates (Figure 40).^61^

**Figure 41:**
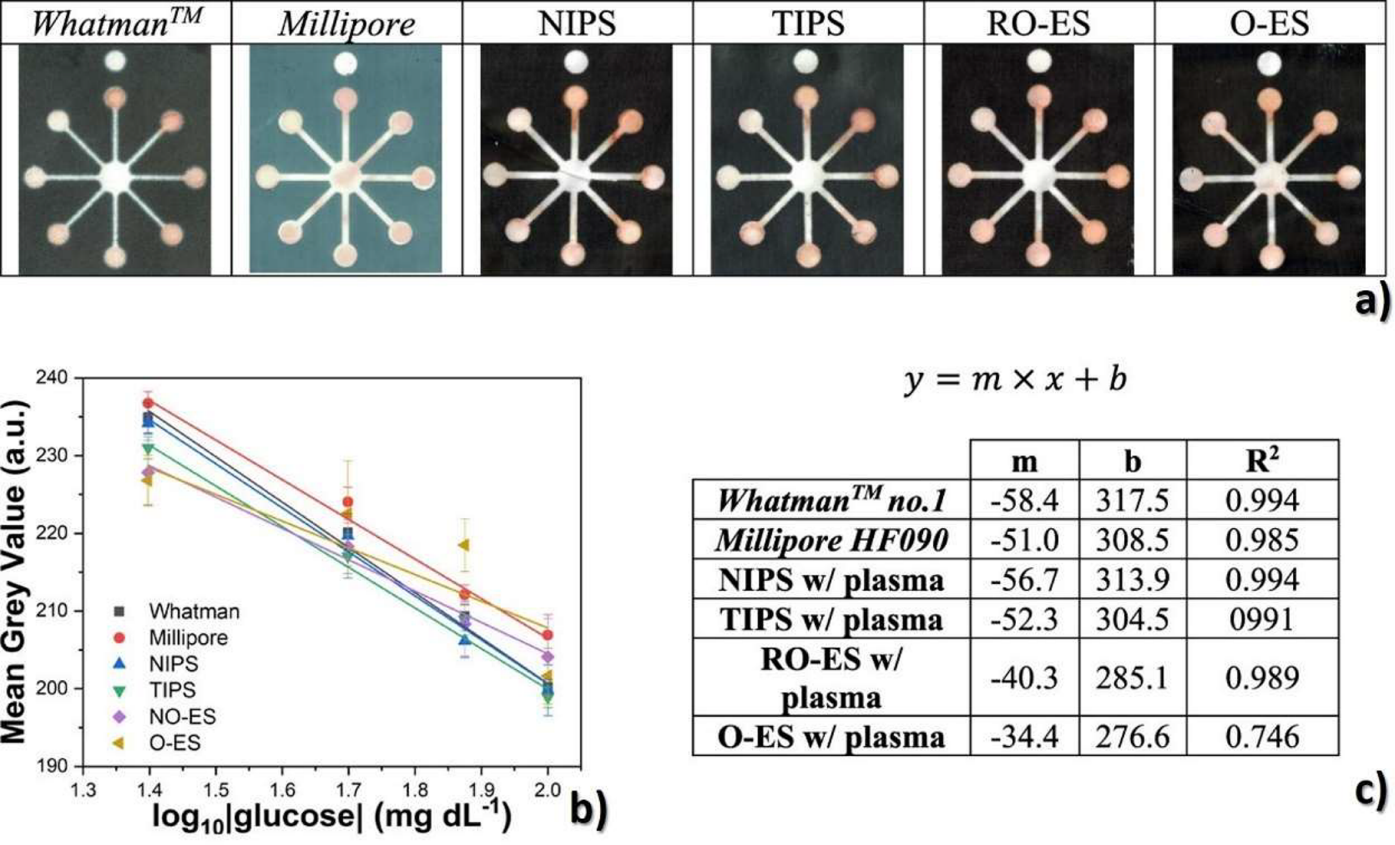
(a) Representative scanned images of commercial substrates and hydrophilic poly(VDF-*co*-TrFE) membranes after glucose assays. For identification of the glucose concentration, see Figure in ref (61). (b) Calibration curves of glucose for commercial substrates and hydrophilic poly(VDF-*co*-TrFE) membranes. The results are presented as mean gray values, and the corresponding standard deviations were measured on the reaction chambers of the microfluidic substrates using ImageJ software. (c) Corresponding linear fitting. Reproduced with permission from ref (61). Copyright 2021 American Chemical Society.

The poly(VDF-*co*-TrFE) membranes feature high wax printing quality, excellent mechanical properties (Young’s modulus from 71.4 ± 2.9 to 163.4 ± 5.1 MPa in the wet state, respectively), and tailorable capillary flow rate (from 35.7 ± 2.5 mm·min^–1^ to 88.3 mm·min^–1^), allowing matching process requirements for specific (bio)technological applications (such as collection, separation, preconcentration, mixing, among others). Moreover, wax-printed microfluidic platforms were designed, printed, and successfully tested for the colorimetric quantifications of glucose in the range of 25–100 mg·dL^–1^. Each microfluidic system includes eight reaction chambers, with each glucose concentration being measured in two reaction chambers separately and at the same time (Figure 41).

The results demonstrate the suitability of the developed poly(VDF-*co*-TrFE) as microfluidic substrates based on their tailorable morphology, improved capillary flow rate, wax print quality, homogeneous generation of colorimetric reaction, and excellent mechanical properties. Moreover, they can be reused after a simple cleaning process, while their electroactive properties make them suitable for the development of a new generation of eco-friendly and smart microfluidic substrates.

Despite the aforementioned advantages and advances in the use of electroactive PVDF-based materials in various aspects of the microfluidic technology, i.e., sensors, actuators, or even as functional substrates, which demonstrate the strong potential of this class of smart polymers, there is still a road ahead of intense and dynamic research to obtain specific tailored properties that will ultimately allow addressing of some of the most challenging (bio)technological applications in the near future.

### 3.6 Biomedical Applications: Tissue Engineering and Antimicrobial Surfaces

Electrical signals, including electromechanical ones, are present in a significat number of biological tissues within the human body. By applying electrical stimuli, important tissue regeneration may be achieved. More recently, the effect of this type of physical stimuli on prokaryotic cells (i.e., bacteria) has also been studied for antimicrobial purposes. Thus, electroactive materials and, in particular, piezoelectric ones, have been widely researched as a source of electrical and mechanoelectrical stimuli to a wide range of eukariotic and prokaryotic cells for advanced biomedical applications. The mechanism behind these phenomenon on both cells is based on the possibility of these material to create electroactive microenvironments (EAMs), upon mechanical stimulation, that act by itself as a tissue regenerator and/or antimicrobial agent. In the case of antimicrobial applications, the role of assisting and boosting the antimicrobial properties of other antimicrobial agents has also been researched.

The natural piezoelectric properties found in specific biological tissues such as bone, nerves, tendons, and skin make it evident that utilizing materials with similar characteristics could bring significant benefits to advanced tissue regeneration strategies, especially when adopting a biomimetic approach.^56,63^ The knowledge of the piezoelectric response of biological tissues may thus be important for highlighting their potential in this regard.^63^ Among the different piezoelectric polymers, PVDF presents the highest piezoelectric coefficient, is biocompatible and chemically stable, all important features in materials development for the biomedical field. For this reason, PVDF has been increasingly used for biomedical applications as it can be used as a sensor,^251,639−641^ actuator,^642−644^ health monitoring,^645−647^ and antimicrobial purposes.^648−650^

#### 3.6.1 Tissue Engineering

**Figure 42:**
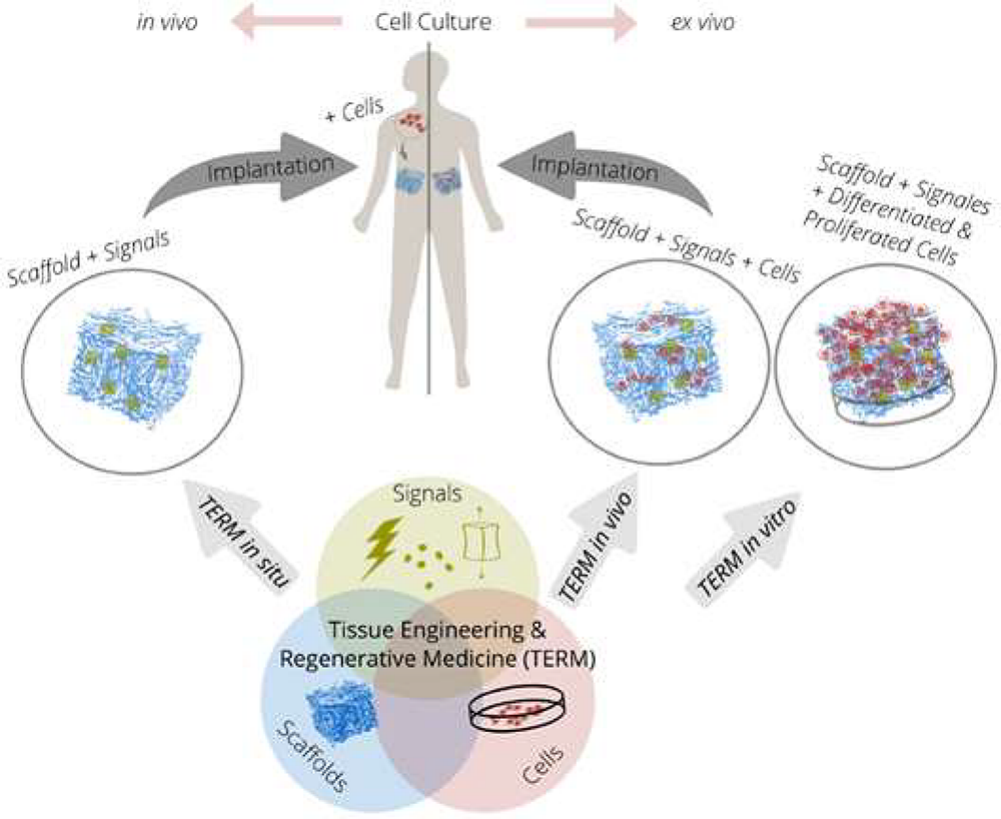
Triad of tissue engineering and regenerative medicine in the context of the *in situ*, *in vivo*, and *in vitro* strategies with *in vivo* and *ex vivo* cell culture. Reproduced with permission from ref (654). Copyright 2021 Multidisciplinary Digital Publishing Institute.

Tissue engineering is a multidisciplinary scientific approach that combines different areas, in particular: medicine, molecular biology, chemical engineering, bioengineering, physiology, developmental biology, nanotechnology, and material science, with the aim to develop materials and cells based strategies to substitute tissues and/or to promote tissue repair/regeneration impaired by disease and/or trauma.^651,652^ This strategy emerged as an alternative to conventional methods and to overcome the gap between the growing list of patients waiting for organ transplantation and the limited number of donated organs available for such procedures.^606,653^ For that, the triad of tissue engineering is based on the complement of three components: cells, scaffolds, and signals (Figure 42).^654^

One of the first paradigms of tissue engineering was the use of supportive materials/matrices capable of providing an appropriate environment for the different tissue grafts and organs of human origin for clinical therapeutics adhesion, growth, and differentiation toward the desired tissue, including fat, fascia, bone, skin, cornea, kidney, liver, heart, and dentin matrix, among others.^655^

Tissue engineering promotes the design and production of scaffolds with mechanical, chemical, and physical properties similar to the 3D biological systems. To positively mimic biological tissues, an optimized scaffold should favor cell penetration, growth, and integration into the host system, and during healing or after healing should, preferably, ensure the degradation into nontoxic byproducts.^656^

One primary requirement for a biomaterial is biocompatibility, and during the last 20 years, various biomaterials ranging from metals to ceramics and polymers have been proposed.^656,657^

Different materials from synthetic or natural origin and different morphologies have been evaluated in order to determine the most prone to replace the cell environment.^658^ Natural materials are attractive for biomedical and tissue engineering applications as they exhibit similar properties to the tissue to be replaced and can be obtained from natural sources.^659,660^ Nevertheless, natural polymers can be difficult to process and usually present poor mechanical and electrical properties.^661^

Polymers are among the most applied materials for tissue engineering due to their chemical and mechanical tunability.^662^ Other parameters such as macro- and microarchitecture, biodegradability, and the physical stability of polymers allow addressing the complex functionalities possessed by each tissue type.^656^ Therefore, a wide range of synthetic polymers have been used to construct different materials/matrices for tissue engineering,^663^ mostly in a passive way, i.e., just working as support for the cells/tissues.^664^ However, many of the key functions in cells and organs of the human body are controlled by dynamical stimuli such as electrical signals.^665^ For example, electrical fields influence the metabolism and growth at different stages and can guide the migration and movement of different cell types such as epidermal, epithelial, and corneal cells,^666−669^ and can modulate the phenotypes of vascular endothelial cells, regenerate nerve fibers, and influence ligament healing.^670−672^ Also, among the different clues that determine tissue development, cells/organs repair and/or regeneration, together with cell behavior and function, electrical and electromechanical signals are essential for tissues such as bone, cartilage, skeletal and cardiac muscle, skin, and neural.^673−675^

In this way, physical signals are particularly relevant parameters to be considered for the development of active materials/scaffolds in order to mimic the body microenvironment, providing the appropriate stimuli for specific cell responses.^676^ Therefore, a new paradigm for tissue engineering emerged, based on the use of active/smart biomaterials with appropriate forms and geometries, aiming to properly regenerate specific tissues.^677^

Such approach allows the induction of these stimuli more naturally, taking advantage of the presence of electrical or mechanical signals of the body.^678^ Therefore, multifunctional biomaterials based on smart materials have been applied in several tissue engineering fields, including bone, cartilage, skeletal and cardiac muscle, and neural regeneration. Among the different smart materials, PVDF-based polymers have already shown strong potential for novel tissue engineering and for such reason will be carefully reviewed in this section.

##### 3.6.1.1 Poly(vinylidene fluoride)-Based Tissue Engineering

Knowing that the electromechanical stimulation can be effectively conducted with the use of PVDF, some studies demonstrated the biocompatibility of PVDF-based materials, reporting also its influence on the cellular activity.^679,680^ Because the bone is piezoelectric, the first studies regarding the use of PVDF as a biomaterial were performed to study the influence of the PVDF’s piezoelectricity on bone regeneration.^659,681,682^

Additionally, the influence of PVDF́s surface charge on the interaction/adsorption of fibronectin was also studied, detecting a higher adsorption on surface charged PVDF rather than on nonsurface-charged PVDF. Later, the influence of the same surface charge on MC3T3-E1 preosteoblasts behavior, cultivated under static and dynamic conditions,^682^ was evaluated, verifying that positive charged surfaces promote higher osteoblast adhesion/proliferation, being even higher under dynamic conditions, i.e., with the application of a mechano-electrical stimuli. A similar approach was used by using human adipose stem cells (hASCs), being verified that both mechanical and electrical stimulation significantly improved the osteogenic differentiation of hASCs.^659^ Such osteogenic differentiation of hASCs was also enhanced by the dynamic piezoelectric stimulation of negative surface charged β-PVDF.^659^ Those studies demonstrated that negative surface charged β-PVDF films can provide the required electromechanical stimuli for the differentiation of specific cells, allowing the design of suitable bone tissue engineering strategies. The influence of electrical stimulation on the proliferation, migration, and maturation of MC3T3-E1 preosteoblasts were also studied with PVDF/BT/MWCNT films.^683^ After 21 days of cell culture and stimulation, it was found that the alkaline phosphatase (ALP) activity, the intracellular Ca^2+^ concentration and also the calcium deposition and mineralization were enhanced. These PVDF/BT/MWCNT films were also implanted in a rat model,^684^ and no inflammatory response was observed. Moreover, there is supporting evidence indicating the occurrence of foreign body reactions accompanied by fibrous encapsulation, wherein the thickness of the encapsulating capsule gradually diminishes over time.

**Figure 43:**
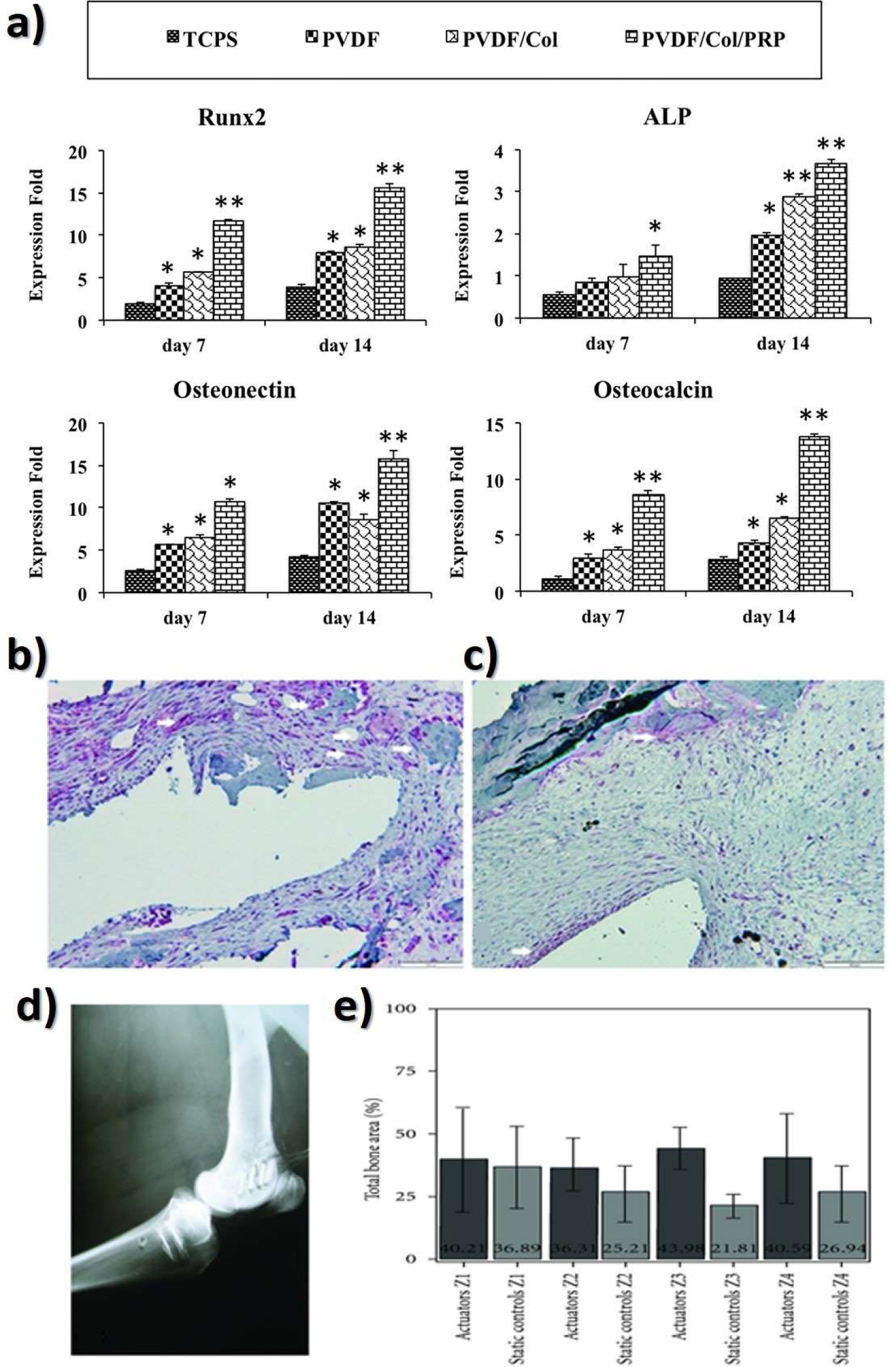
(a) Bone-related gene expression evaluation of human induced pluripotent stem cells while cultured on the PVDF, PVDF/collagen, and PVDF/collagen/PRP nanofibrous scaffolds and TCPS as a control one and two weeks after cell seeding. The significant differences (*p* < 0.05 and *p* < 0.01) between groups are indicated with one- and two-star signs, respectively. ALP; human induced pluripotent stem cells, induced pluripotent stem cell; PRP; PVDF; β-tricalcium phosphates (TCPs); tissue culture polystyrene (PS). Microphotograph of decalcified sections, osteopontin (white arrows), and proliferating cell nuclear antigen (PCNA) expression. Picture shows Z3 areas of femoral. Reproduced with permission from ref (685). Copyright 2020 Wiley-VCH. (b) Actuator and (c) static control, suggesting more extensive osteopontin labeling around actuator. Double Fast-Red and DAB immunohistochemistry staining for osteopontin and PCNA, respectively. Scale bar represents 100 μm. (d) Postoperative radiograph 30 days after implantation showing the six actuators in place (four in the femur, two in the tibia). There are neither signs of periostal or peri-implantar reaction nor signs of infection in neighboring soft tissues. (e) Total bone area measured around actuators and static controls, organized by areas Z1 to Z4. Bars represent means and error bars standard deviation. Reproduced with permission from ref (642). Copyright 2012 Hindawi.

Stem cells (human induced pluripotent) were also cultured on PVDF-based composite nanofibers.^685^ The developed PVDF/collagen/platelet-rich plasma (PRP) composite nanofibers exhibited good biocompatibility. Additionally, the results obtained through the ALP activity and also calcium content assays proved that PVDF-based nanofibers promote higher osteoinductivity. Such results make this composite a promising candidate for the treatment of bone lesions. According to bone-related gene expression evaluation results (Figure 43a), it was concluded that the developed PVDF/collagen/PRP-based biomaterial present higher osteoinductivity when compared to the PVDF/collagen composite, demonstrating a promising bone bioimplant. PVDF was also combined with polycaprolactone (PCL) to create fibrous scaffolds for bone regeneration,^686^ demonstrating promotion of human mesenchymal stem cells differentiation into osteoblasts.

*In vivo* studies were also carried out to evaluate the influence of the electroactive features of β-PVDF films on bone defect recovery, verifying that poled β-PVDF films leads to more defect closure and bone remodeling than nonpoled PVDF films and randomly oriented electrospun fiber mats.^687^ A piezoelectric actuator has been also set^642^ in steomy cuts in the femur and tibia of sheep in order to mechanically stimulate bone tissues (Figure 43b–e), reporting that, after one-month implantation, a significantly higher total and new bone area were observed in the regions close to the actuators. Furthermore, significantly higher bone deposition rate was also observed in the mechanically stimulated areas, together with increased osteopontin expression.

The introduction of magnetostrictive fillers into the PVDF’s matrix allows the development of magnetoelectric materials that have been also explored for bone tissue engineering. This approach can be useful in the case of the immobilization of the patient, where the natural mechanical stimulus is not fully ensured or even impossible,^688^ allowing the use of an external magnetic field to remotely stimulate tissue regeneration/differentiation. Keeping this in mind, Terfenol-D/poly(VDF-*co*-TrFe) magnetoelectric composites have been applied to study the proliferation of MC3T3-E1 preosteoblast cells, noticing that when the cells were cultured under mechanical and electrical stimulation or with the application of a magnetic field, the cell proliferation was enhanced.^689^ Thus, it was proven that the magnetoelectric materials can be a successful strategy for tissue engineering.

Following this route, magnetoelectric nanocomposite scaffold composed of PVDF/GO/CoFe_2_O_4_ were used for high yield differentiation of mesenchymal stem cells to neural-like cells.^690^ The neural-like cells tended to differentiate instead of proliferating when the magnetic field was applied to the cells, in same direction to the stimuli. The chemical differentiation factors, on the other hand, revealed just a lower cell differentiation compared to the cells incubated in the presence of a magnetic bioreactor. Beyond tissue engineering, the PVDF/GO/CoFe_2_O_4_ composites can also have potential applications as biosensors and bioactuators.

By placing materials on a secondary role, and geometry on a key role, a comparison of the potential of osteogenic differentiation of induced pluripotent stem cells on 2D and 3D PVDF-based scaffolds has been addressed.^691^ The 3D-PVDF nanofibrous scaffold revealed a better osteoinductive properties when compared with the 2D PVDF film counterpart. The osteogenic differentiation of human mesenchymal stem cells has been also studied on electrospun β-phase scaffolds and compared to tissue culture with PS.^692^ Human mesenchymal stem cells cultured on both types of the scaffolds were successfully attached as proven by a spread morphology. Additionally, cells on PVDF-based scaffolds were found to exhibit the greatest ALP activity and early mineralization by day 10 as compared to tissue culture PS. Such results can be explained by the material similarity of the scaffolds and the bone, concluding that the 3D structure could lead to better/more efficient bone differentiation.

PVDF-based biomaterials for muscular tissue engineering have shown quite promising results, in particular with respect to the effect of materials surface charge on the enhancement of myoblast cell proliferation^693^ and differentiation.^56^ Magnetoelectric CoFe_2_O_4_/poly(VDF-*co*-TrFE) films were also used to investigate the influence of mechano- and electrical stimuli on the differentiation of myoblast cells.^694^ The myoblast differentiation is enhanced with the application of the mechanical and/or electrical stimulation, with higher values of maturation index of the myotubes under the mechanoelectrical stimuli. This work demonstrates the potential of the use of magnetoelectric stimulation for skeletal muscle tissue engineering.

In the area of heart tissue regeneration, β-PVDF based electrospun nanofibers were introduced as a promising material for the development of cardiac patches.^695^

**Figure 44:**
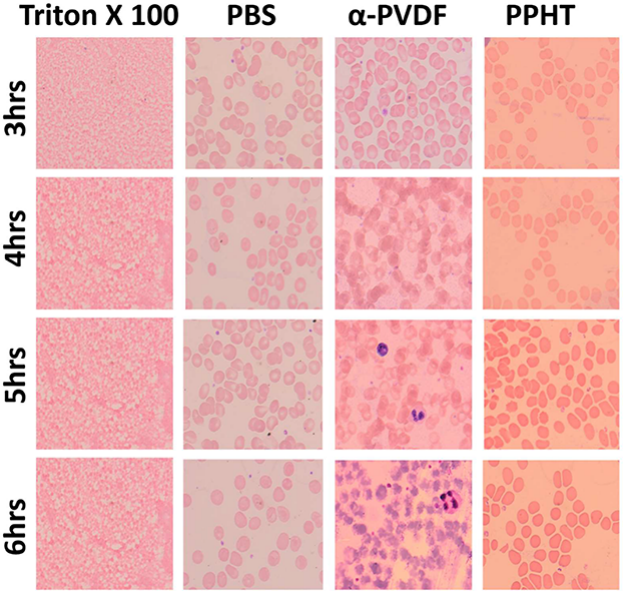
Stained blood smears of whole blood incubation with test materials (Triton X-100, phosphate buffer solution-PBS, α-PVDF, and β-PVDF-PMMA/HAp/TiO_2_ (PPHT)) at the end of 3, 4, 5, and 6 h time periods. Reproduced with permission from ref (696). Copyright 2018 Elsevier.

It was shown that the produced PVDF based biomaterial is mechanically stable, supporting the adhesion and differentiation of cardiomyocytes comparing to the standard nonpiezoelectric scaffolds used as control. These fibers were also coated with vitronectin-derived peptide-mussel adhesive protein fusion (VNm) and used to cultivation of human embryonic stem cells (hESCs).^697^ The results revealed that under cardiac differentiation conditions, more spontaneously beating colonies were generated, as well an upregulation of cardiac-related genes. Additionally, *in vitro* hemocompatibility studies (Figure 44) revealed that β-PVDF-PMMA/HAp/TiO_2_ (PPHT) nanofibers did not induced hemolysis to the red blood cells at the end of 6 h exposure time, where the PPHT scaffold can be interfaced with direct blood contact organs such as heart.^696^ This promising report conjugated with stem cell engineering strategies can be a disruptive tool for repairing damaged heart muscles. PVDF fibers have been also combined with gelatin and GO for cardiac tissue engineering^698^ in order to decrease the hydrophobic nature of the PVDF fibers. In this study, it was demonstrated that the introduction of GO into the PVDF matrix improves the piezoelectric properties of pristine PVDF. To study the potential of the produced PVDF/gelatin/GO fibers, embryonal cardiomyocyte cells (ECCs) were used, and the cell proliferation and gene expression (cardiac troponin, Connexin 43, and GATA-4 binding protein) was evaluated. It was verified that PVDF/gelatin/GO fibers present a higher gene expression and also induce a higher cell alignment when compared with the PVDF/gelatin and gelatin fibers. The results demonstrated the potential of the PVDF/gelatin/GO fibers to convert ECCs cell into heart muscle. Poly(VDF-*co*-TrFE) electrospun randomly oriented and aligned fibers were also produced to create myocardial implants.^699,700^ The differentiation of human-induced pluripotent stem cells (hiPSCs) into cardiomyocytes and their long-term culture/maturation with the poly(VDF-*co*-TrFE) fibers was demonstrated.^699^ Poly(VDF-*co*-TrFE) was also combined with ILs as novel and promising platform for cardiac tissue engineering.^701^

Drop-cast PVDF scaffolds have been also developed and treated by cold plasma for cardiac tissue engineering^702^*in vitro* and *in vivo* assays. In the *in vitro* assays, newborn rat primary cardiomyocytes were used and a higher cell adhesion, a well-organized sarcomeric structure and also higher gene expression related to adhesion and cardiac function were observed in the plasma-treated drop-cast PVDF scaffolds. After the promising results, an *in vivo* study was performed on healthy murine models in order to verify if the produced scaffolds when implanted on hearts did not increase inflammation. After 28 days of implantation, no toxic or immune responses were observed, demonstrating the potential of this kind of material to be used for cardiac tissue engineering.

**Figure 45:**
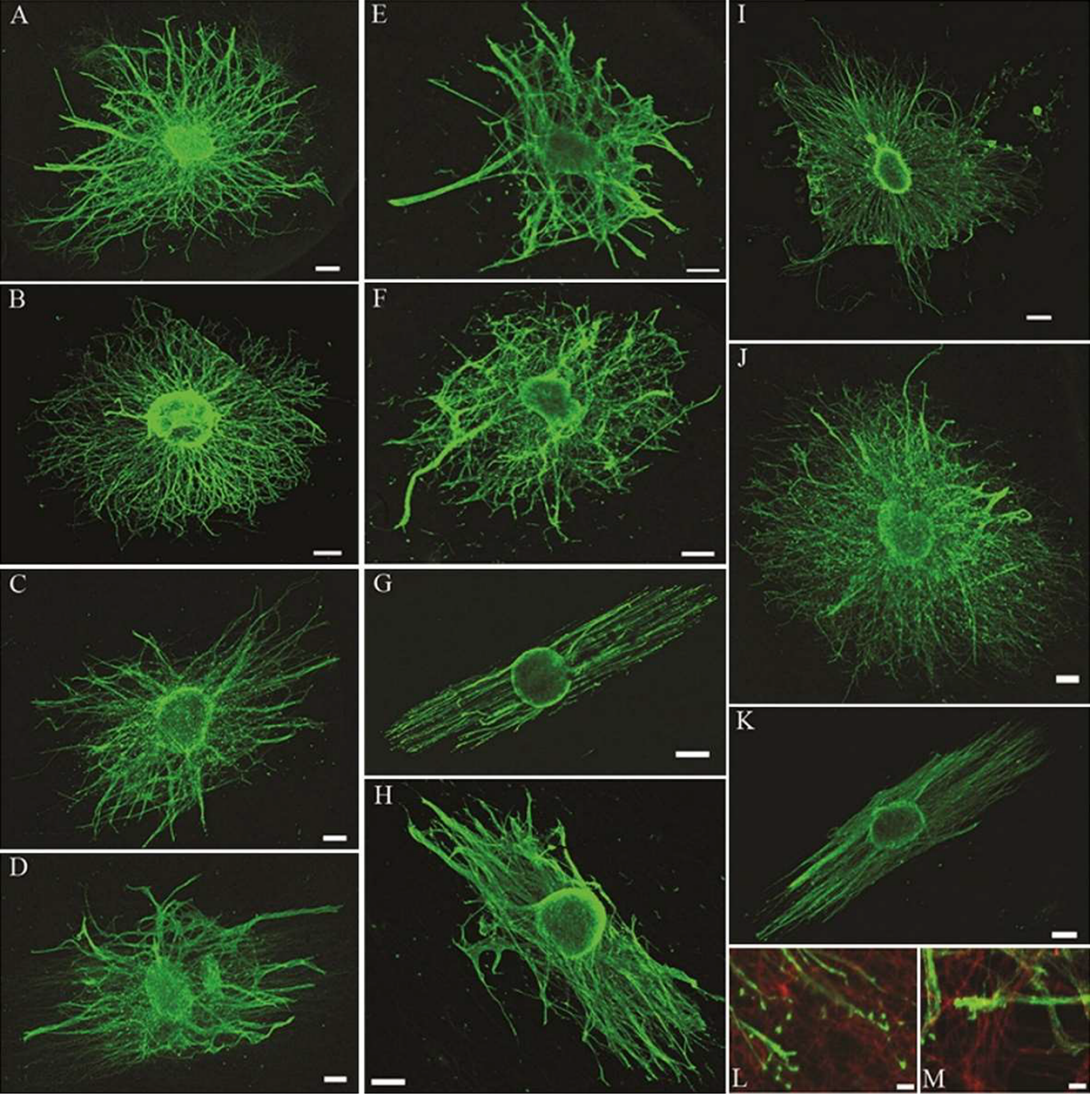
Confocal fluorescent images of root ganglion neurons stained with phalloidin (actin) on nanosized as-spun and annealed (a and b) random and (c and d) aligned poly(VDF-*co*-TrFE) and micrometer-sized as-spun and annealed (e and f) random and (g and h) aligned poly(VDF-*co*-TrFE) scaffolds, a collagen-coated surface (i), and nanosized (j) random and (k) aligned PVDF scaffolds (magnification 4×, scale bar 300 μm). Confocal fluorescent images of root ganglion neurons neurite tips stained with phalloidin (green) attached to poly(VDF-*co*-TrFE) micrometer-sized (l) annealed aligned and m) as-spun random fibrous scaffolds (red) (magnification 20×, scale bar 50 μm). Reproduced with permission from ref (703). Copyright 2011 Elsevier.

Concerning neural tissue engineering studies, PVDF and its copolymer poly(VDF-*co*-TrFE) have been evaluated under static and dynamic conditions, reporting that the number of differentiated neurons of mouse neuroblastoma cells increased with the interaction with poled (i.e., overall average surface charge) PVDF substrates.^671^ Electrospun piezoelectric scaffolds of poly(VDF-*co*-TrFE) were also found to increase the neurite extension of primary neurons,^703^ noting that dorsal root ganglion neurons were successfully attached to all fibrous scaffolds. The effect of the alignment state of the poly(VDF-*co*-TrFE) fiber scaffolds on neurite extension was monitored by confocal fluorescent images (Figure 45), and it was shown that neurite extension was improved on aligned and annealed (135 °C for 96 h and quenched with ice water) poly(VDF-*co*-TrFE) comparing with annealed as-spun random poly(VDF-*co*-TrFE) scaffolds.

Poly(VDF-*co*-TrFE) aligned fibers were also produced and used to support Schwann cells growth, neurite extension, and myelination,^704^ demonstrating great potential for spinal cord repair.

In a different scaffold morphology, PVDF membranes have been acoustically stimulated in order to study the effect of dynamically induced surface electrical charges on the behavior of neuritogenesis of PC12 cells.^705^ It was discovered that the calcium channels were successfully activated, generating neurites via a cyclic adenosine monophosphate (cAMP)-dependent pathway. PVDF films with different surface charge (none, positive, and negative) were also produced and submitted to dynamic mechanoelectrical stimuli in order to investigate their influence on neuron-like cells adhesion, proliferation, and differentiation.^706^ It was verified that piezoelectric dynamic stimulation can enhance the proliferation of SH-SY5Y cells and also improve neurite extension and differentiation.

Also produced through electrospinning, PVDF/Au nanoparticles composites were used for nerve tissue regeneration,^707^ the composites showing the ability to increase growth and adhesion of cells without any toxicity, exhibiting also a suitable proliferation after culturing for 24 h.

**Figure 46:**
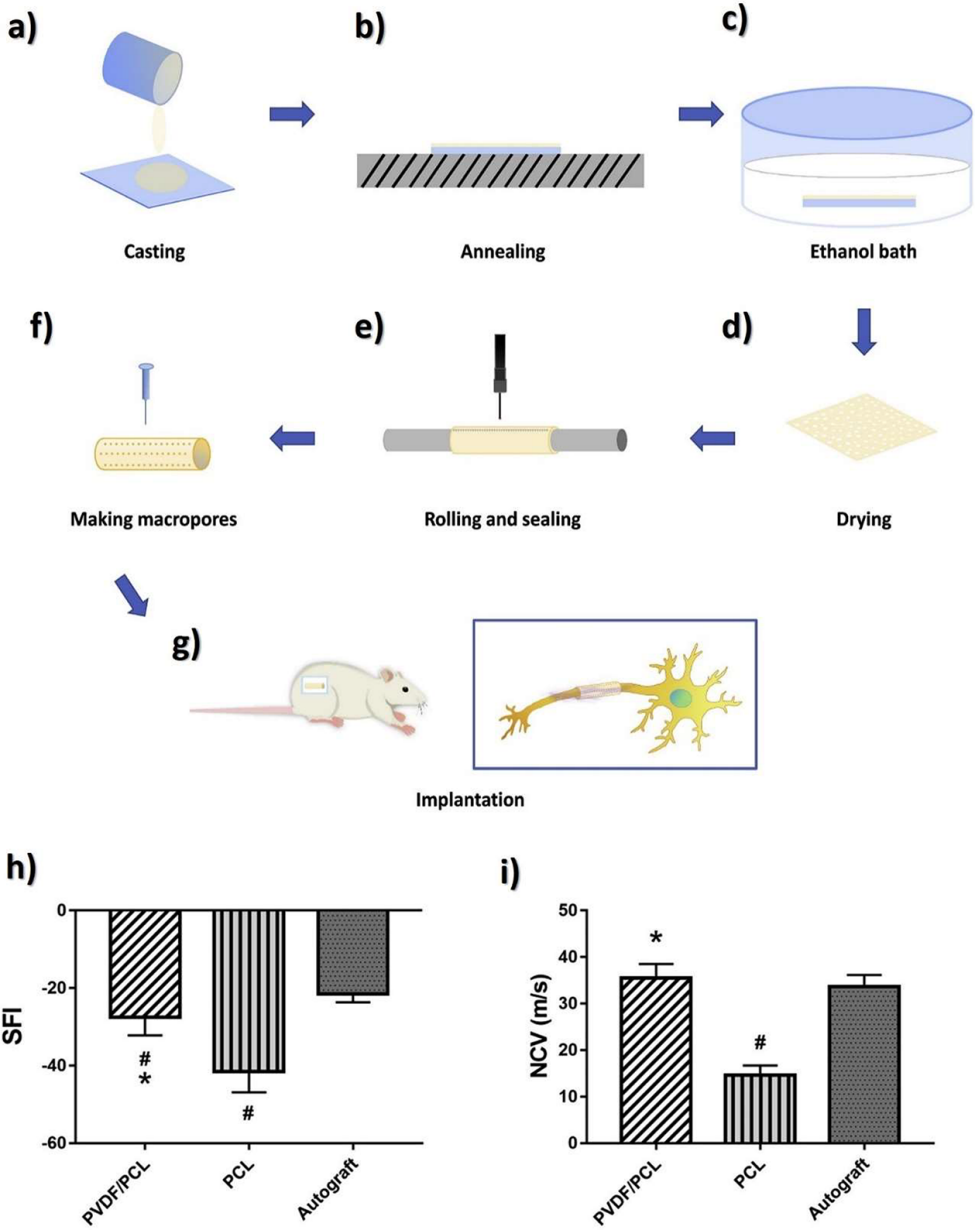
Fabrication of PVDF/PCL nerve guidance channels. (a) Casting on glass mold. (b) Annealing at 55 °C for 12 h. (c) Immersing in an ethanol bath. (d) Drying under vacuum at room temperature overnight. (e) Rolling around a cylindrical model with heat sealing process. (f) Microporous structures by needles. (g) Implantation of NGCs to connect 15 mm sciatic nerve defect in the SD rats. Functional and electrophysiological assay of the regenerated nerves. (h) Sciatic function index values of the sciatic nerves. (i) Nerve conduction velocity (NCV) of the sciatic nerves. Data are expressed as mean values ± SD (*n* = 5, *; *p* < 0.05 vs PCL, #; *p* < 0.05 vs autograft). Reproduced with permission from ref (708). Copyright 2019 Elsevier.

3D structured self-powered PVDF/ PCL scaffolds were optimized for peripheral nerve regeneration (Figure 46).^708^

It has been stated that 3D PVDF-based scaffolds promoted, *in vitro*, the adhesion and proliferation of the rat Schwann cells (RSCs), exhibiting substantial electrophysiological, morphological, and functional nerve restoration. Additionally, a significant electrophysiological recovery for the injured nerves was observed. The same materials (PVDF/PCL) were used to develop biodegradable piezoelectric nanotracts for long-gap peripheral nerve repair.^709^ The PVDF/PCL nanotracts were implanted in a 15 mm sciatic nerve defect of a rat model, and upon sono-electro-mechanical therapy, it was verified a higher renewal of complex motor functions, Schwann cell repopulation, axonal growth and maturity, and gastrocnemius muscle histology. The obtained results demonstrated that sono-electro-mechanical therapeutic system is a promising approach for clinical treatment of peripheral nerve injuries.

A novel strategy to induce neural differentiation based on piezotronics was also developed.^710^ For that, a layer of FeOOH nanorods was assembled on the surface of PVDF electrospun fibers. The hybrid nanofibrous membrane was stimulated under ultrasonic irradiation, inducing the neural differentiation of rat bone-marrow-derived mesenchymal stem cells (rBMSCs) cultured on the surface of these membranes. Furthermore, the differentiated cells generated fast peaking spontaneous [Ca^2+^], indicating the neural function of rBMSCs-derived neurons. In this way, this study provides a novel strategy for inducing neural differentiation without the need of neural inducing factors.

**Figure 47:**
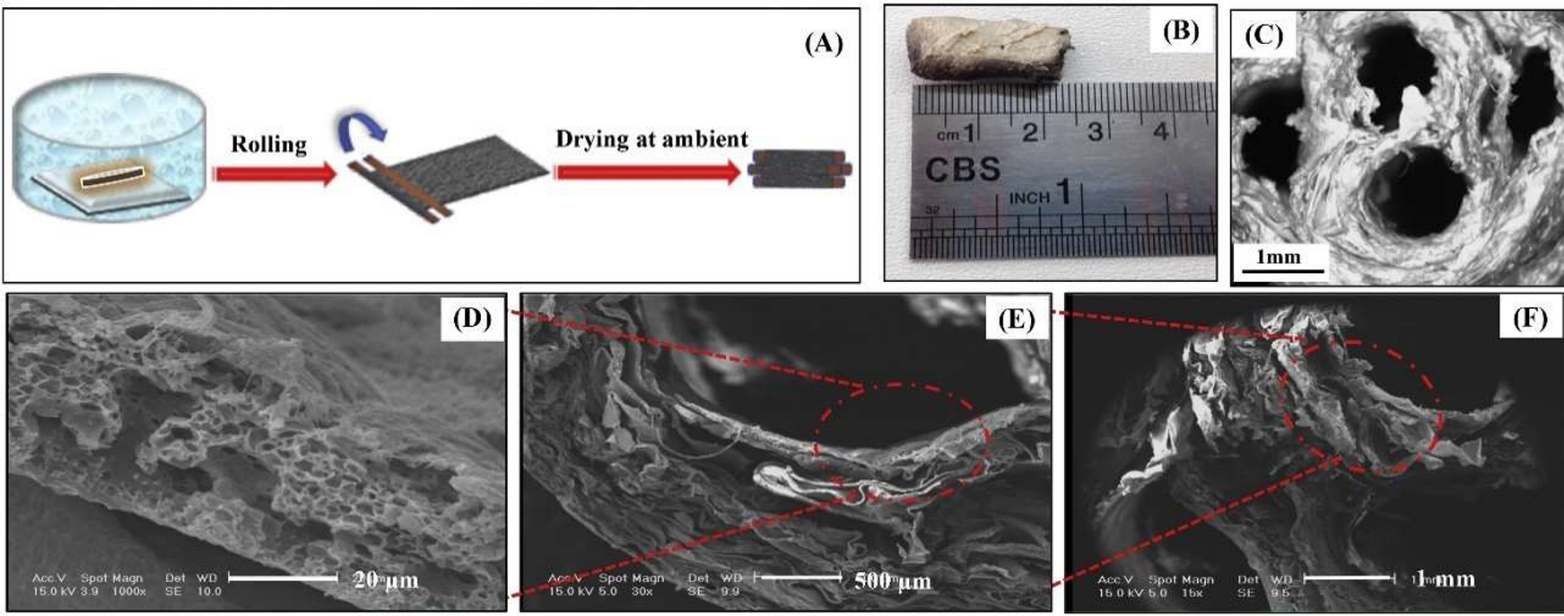
(a) Schematic of the nerve conduit fabrication. (b,c) Optical microscopy images of the nerve conduit. (d–f) SEM images of the nerve conduit at different magnifications (15×, 30×, and 1000×). Reproduced with permission from ref (711). Copyright 2019 Elsevier.

3D piezoelectric PVDF/GO scaffolds have been also produced in a porous morphology by NIPS method (Figure 47).^711^

PVDF/GO properties were modulated by the GO nanoparticles incorporation, finding that the addition of 0.5–1 wt % of GO promoted optimized mechanical (tensile modulus of ∼8.1 MPa) and electrical properties (impedance of ∼804 Ω at 10 Hz), that in turn increased PC12 cell proliferation. Such PVDF/GO scaffolds can be easily adapted to nerve guidance conduits with four internal longitudinally aligned channels.

PVDF has been also combined with other polymers such as PU in form of fibers for wound healing applications in order to improve the activity/functionality of cells. Such studies proved that when the electrospun composites were subjected to a mechanical deformation, the scaffolds enhanced the fibroblast activities (both *in vitro* and *in vivo*), proving their high potential for wound healing treatments.^712^

PVDF-based materials can also play pivotal role in patient’s bladder functional recovery as shown in PVDF nanofibrous scaffolds fabricated through electrospinning (with or without chitosan nanoparticles loading).^713^ 3-(4,5-Dimethylthiazol-2-yl)-2,5-(diphenyltetrazolium bromide) tetrazolium reduction (MTT), reverse transcription polymerase chain reaction (qRT-PCR), and immunocytochemistry results demonstrated that highest adipose tissue derived mesenchymal stem cells (AT-MSCs) proliferation rate and smooth muscle cell (SMC) differentiation potential were detected when cultured on the PVDF-transforming growth factor-β scaffold, enabling greater treatment possibilities in bladder tissue engineering applications.

Regarding esophagus tissue engineering, a PVDF mesh structure allows the local tissue regeneration of esophagus tissues. For that, semicircular esophageal defects of 0.5 × 1 cm^2^ were created 2 cm close to the cardia in 10 rabbits. These defects were filled with PVDF or polyglactin 910 and later covered by omental wrapping. The clinical results were evaluated by the clinical observation, X-ray contrast medium examinations, and regular esophagoscopies, the local tissue regeneration being verified by light microscopy and immunohistochemistry. The results revealed that after 3 months, no anastomotic structures were detected, reporting a complete mucosal regeneration, with negligible inflammation reaction and also an initial muscle layer regeneration in the group where PVDF was used. In the group treated with polyglactin 910, it was observed that the patch presents failures with consecutive anastomotic leakage occurrences.^714^

A electrospun poly(VDF-*co*-TrFE)/ZnO nanocomposite tissue engineering scaffold demonstrated to promote adhesion, migration, and proliferation of cells, as well as blood vessel formation (angiogenesis).^715^ Only minimal adverse effects have been detected of the poly(VDF-*co*-TrFE)/ZnO biomaterials with regard to *in vitro* blood compatibility, cytotoxicity, and biocompatibility, demonstrating that poly(VDF-*co*-TrFE)/ZnO nanocomposite scaffolds can be used for tissue engineering applications. Interestingly, human mesenchymal stem cells and human umbilical vein endothelial cells cultured on the nanocomposite scaffolds exhibited higher cell viability, adhesion, and proliferation when compared to cells cultured on tissue culture plates or neat poly(VDF-*co*-TrFE) scaffolds.

PVDF-based materials have been also used for the manipulation of differentiated Madin-Darby canine kidney (MDCK) cell sheets.^716^ The authors succeeded in harvesting confluent MDCK cell sheets and then transferring them intact to other culture plates using PVDF membranes that were hydrophilically modified as supporting materials. Immunocytochemistry tests in the transferred MDCK cells with anti-β-catenin antibody showed that the functional cell–cell junctions were well organized. The viability assay revealed that the transferred cells were not injured during the manipulation of the 2D cell sheet. Through transmission electron microscopy (TEM), it was observed that the harvested MDCK maintain the differentiated phenotypes, with a high number of microvilli and tight junctions at the apical and lateral plasma membranes, respectively. This technique of 2D cell-sheet manipulation^716^ opened other routes to the use of PVDF-biomaterials to be applied in epithelial cell sheets research.

The overall research scenario and the high number of developed materials/composites (Table 15) allows conclusion that the piezoelectric effect and PVDF, copolymers, and composites as active biomaterials have already successfully demonstrated its suitability for bone tissue engineering as well as the strong potential for other electrically active tissues, such as neural or muscular, that respond to electrical and mechano-electrical stimuli.

**Table 15:** PVDF Biomaterials, Stimuli Applied and Biorelated Features for Different Tissue Engineering Applications

material	stimuli type	biorelated features	ref
Bone Tissue			
PVDF film	mechano-electric	positive charged surfaces promote higher osteoblast adhesion and proliferation, being higher under dynamic stimulation	(682)
		both mechanical and electrical stimulation significantly improved the osteogenic differentiation of hASCs	(659)
	mechano-electric *in vivo*	the films lead to more defect closure and bone remodeling	(687)

PVDF/BT/MWCNT film	electrical	the electrical field stimulation lead to an enhanced osteogenic activity, with a significant increase of the ALP activity, intracellular Ca^2+^ concentration, and calcium deposition and mineralization	(683)
PVDF actuator	electrical	bone deposition rate was significantly higher in the mechanically stimulated areas	(642)
PVDF melt-spun fibers	topography/material	fibers processed with higher voltage present higher ALP activity and early mineralization	(692)
PVDF/collagen/platelet-rich plasma nanofibers	topography/material	the fibers present higher osteoinductivity compared with fibers without platelet-rich plasma	(685)
PVDF-Ba_0.9_Ca0_.1_TiO_3_/PVA core–shell fibrous membrane	topography/material	osteogenic differentiation of mesenchymal stem cells (MSCs), in the absence of osteogenic supplements was observed	(681)
terfenol-D/poly(VDF-*co*-TrFE) films	magneto-, mechano-electric	the proliferation of preosteoblast cells was enhanced under mechanical and electrical stimulation	(689)
Skeletal Muscle Tissue			
PVDF films and fibers	topography/material	PVDF with negatively charged surfaces improve cell adhesion and proliferation of C2C12 muscle cells. Also, aligned fibers promote the directional growth of the myoblast cells	(693)
		PVDF promotes myogenic differentiation of C2C12 cells	(47)

CoFe_2_O_4_/poly(VDF-*co*-TrFE) films	magneto-, mechano-electric	the magnetoelectric composites enhanced the proliferation and differentiation of the myoblast cells by the application of mechanical and/or electrical stimulation	(694)
Cardiac Muscle Tissue			
PVDF electrospun scaffolds	topography/material *in vivo*	the scaffolds support improved cell adhesion and differentiation when compared to standard non piezoelectric scaffolds	(695)
PVDF electrospun scaffolds coated with VNm	topography/material	under cardiac differentiation conditions, hESCs on the VNm–PVDF scaffold generated more spontaneously beating colonies and showed the upregulation of cardiac-related genes	(697)
Neural Tissue			
PVDF substrates	electric	the effect of pure piezoelectric stimulation on neurite generation in PC12 cells is comparable to the ones induced by neuronal growth factor (NGF). Also, the dynamic PVDF stimulation by ultrasonic waves activates the calcium channels, inducing the generation of neurites	(705)
		mouse neuroblastoma (Nb2a) cells grown on piezoelectric substrates exhibited significantly greater levels of process outgrowth and neurite lengths	(671)
		SH-SY5Y cell grown on PVDF films submitted to electrical stimulus improved neurite extension and differentiation	(706)

Poly(VDF-*co*-TrFE) electrospun aligned and random scaffolds	topography/material	dorsal root ganglion (DRG) neurons showed improved neurite extension in micron-sized aligned fiber scaffolds	(703)

PVDF/PCL scaffolds	topography/material *in vivo*	RSCs were cultured on top of the scaffolds, verifying that the electromechanical interactions stimulate the cell proliferation and differentiation	(708)
		*in vivo* assay, the group implanted with PVDF/PCL exhibits significant electrophysiological, morphological and functional nerve restoration	

Au NPs/PVDF electrospun scaffolds	topography/material	the composite nanofibers show the ability to encourage growth and adhesion of PC12 cells, showing normal proliferation besides elongated and spread-out morphology	(707)
FeOOH/PVDF nanofibrous hybrid membrane	electric	the hybrid scaffold enhanced the differentiation of rBMSCs into functional neurons	(710)
PVDF/GO3D scaffolds	topography/material	PC12 cells were cultured on the scaffolds and it was verified that the PVDF-GO scaffolds significantly promoted cell proliferation	(711)
Wound Healing Tissue			
PVDF/GO/CoFe_2_O_4_	magnetic- and electric	alignment of cells toward the same direction. Cells tend to differentiate and proliferate when a magnetic field is applied to the cells	(690)

#### 3.6.2 Antimicrobial Surfaces

A recent global study published by the journal *The Lancet* about antimicrobial resistance (AMR) reported that infections caused by antibiotic-resistant bacteria caused 1.27 million deaths in 2019, killing more people than HIV/AIDS (864 000 deaths) or malaria (643 000 deaths). The imminent risk of infections caused by these bacteria is one of the biggest threats to human health and considered by the World Health Organization (WHO) a priority health issue.^717^ Although new drugs are constantly being sought, the pace of development is slow compared with the evolution and spread of multidrug-resistant bacteria.^718^

**Figure 48:**
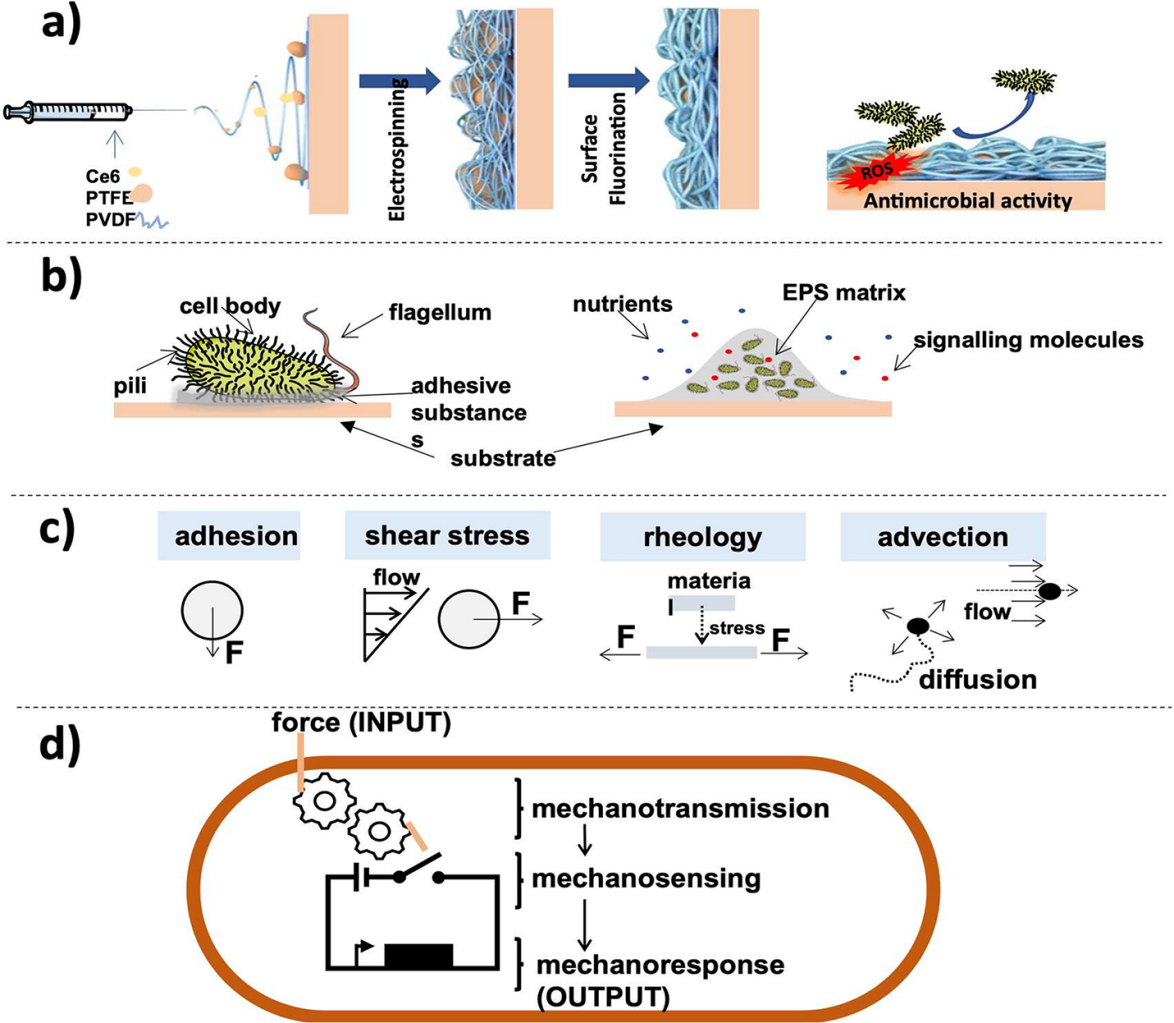
(a) Schematic illustration of the procedure for the preparation of composite coatings with both superhydrophobic and photodynamic antibacterial performances. (b) Representation of substances bacteria use for the attachment of individual bacterial cells and biofilm encased cells to surfaces. (c) Forces (*F*) that a cell feel when attaching to a surface or in a fluid-environment. (d) A switch-like model for bacterial mechanotransduction. Contact with a surface and fluid flow generates forces that are mechanically transmitted by force bearing structures (mechanotransmission). These are coupled with mechanosensors that modulate their biochemical activity upon force transmission which eventually yield a mechanoresponse such as transcriptional regulation.

That is why novel materials with antimicrobial properties and with capacity for inhibiting the adhesion of bacteria and inducing bactericidal effects have been highly researched. Even though PVDF and its copolymers are not intrinsically antimicrobial, they are often the polymers of choice to create such materials due to their low surface energy, chemical inertness, mechanical strength, and thermal stability, often related to the development of superhydrophobic membranes.^719^ Thus, these polymers are mainly used as a support for the incorporation of antimicrobial agents. As an example, PVDF-based superhydrophobic membranes have been developed^719^ where microsized PTFE particles and the photosensitizer Chlorin e6 were wrapped in nanosized PVDF fibers using electrospinning technique (Figure 48a). Upon light illumination, the membrane induced high antimicrobial properties, with extremely low bacterial survival rates (0% for *S. aureus* and ∼1% for *E. coli*). The bactericidal effect was photodynamically stimulated due to the formation of reactive oxidative species (ROS) upon Chlorin e6 irradiation. The same mechanism of action was used to obtain an antimicrobial PVDF-based material. The composite membrane composed of PVDF and zeolitic imidazolate framework-8 (ZIF8) induced strong bactericidal activity against *Staphylococcus aureus* and Gram-negative *E. coli* owed to the metal ions (Zn^2+^) and the generated ROS under photoexcitation.^720^

In these cases, PVDF is selected based on its physicochemical characteristics and reliance on environmental factora, acting solely as a polymeric matrix for bearing the compounds with antimicrobial properties, not taking part on the antimicrobial activity. Table 16 presents an overview on recent applications of PVDF materials as a carrier of antimicrobial compounds.

**Table 16:** Relevant PVDF-Based Materials for Antimicrobial Purposes

antimicrobial agent	functionalization	results	application	ref
nano CuAl_2_O_4_ spinel fabricated by coprecipitation that effectively stabilized copper with minimized Cu^2+^ leachability	PVDF membrane functionalized via both doping and coating methods	the *E. coli* attachment was reduced in 68% on the membrane coated with nano spinel, and the membrane is biocompatible	filtration membranes	(752)
Ag/Zn coatings and electrospun PVDF/PS nanofibers	cotton surface functionalize with Ag and Zn through magnetron sputtering layers with PVDF/PS nanofibers made by electrospinning, creating a bilayer structured composite filter	the composite medium can capture and sterilize the pathogenic contaminants in the air effectively, reducing inf 99.64% *E. coli* and 98.75% *S. aureus*	high-performance face mask media for public health protection	(753)
polyhexamethylene guanidine (PHMG) and tannic acid (TA)	PVDF micropore membrane modified with PHMG and further tagged with TA using a dip coating method	*S. aureus* and *E. coli* was inhibited in more than 90%	filtration membrane for wastewater treatment/ water purification	(754)
amino-modified GO, DA and 1,3-diaminoguanidine hydrochloride (DAGH)	PVDF membrane functionalized through surface grafting with oxidative deposition	the modified membrane reduced *E. coli* in 96%, while the raw membrane had no antimicrobial activity.	filtration membrane	(755)
tea polyphenol (TPs) and Ag	PVDF/TP and PVDF/TP/Ag composites produced by electrically assisted 3D printing method	TP nucleated PVDF β-phase and inhibited *E. coli* in 97.22%. With the presence of Ag, the antibacterial activity improved significantly reaching 99.58%	wound dressings for wound infection control	(756)
ZnO, ZnO/V, ZnO/V- CH and V_CH	PVDF nanofibers were prepared by electrospinning via one-step electrospinning process	variable antimicrobial results were obtained for *S. aureus* and *E. coli* being the best composite the PVDF_ZnO/V_CH	suitable for developing medical materials such as filters, medical textiles, mask or wound dressings	(110)
ammonium or quaternary pyridinium monomers	graft copolymers prepared by grafting the monomers to PVDF and synthesized by ATRP. Further films of these copolymers were obtained by solvent casting	the polymers could effectively kill *S. aureus*, *E. coli*, and the pathogenic yeast *C. albicans* (antimicrobial rates >99.99%) while the blends exhibited around 99% inhibition rates	general materials for biomedical applications	(757)
Ag nanoparticles	Ag NP-filled poly(VDF-*co*-HFP) fibers produced by electrospinning; further placing this fibers on an AAO porous template and heat them above the glass transition temperature led to the fabrication of hierarchical PVDF-HFP fibers with Ag nanoparticles	poly(VDF-*co*-HFP) fibers filled with Ag nanoparticles and the hierarchical poly(VDF-*co*-HFP) fibers filled with Ag nanoparticles exhibited inhibition against *methicillin-resistant Staphylococcus aureus* (MRSA), *Pseudomonas aeruginosa*, and *Candida albicans*	potential applications on air filtration, water treatment, protective clothing	(758)
Au/selenium (Se) nanoparticles	CA/ PVDF based nanofibrous with the nanoparticles were prepared by pulsed laser ablation in liquids	the presence of Au/Se nanoparticles induced antimicrobial activity against *Aspergillus niger* when irradiated with light due to the plasmonic effect of Au	filtration membranes	(759)
MOF-801 and Cu_2_O nanoparticles	PVDF nanofibers embedded with MOF-801 and Cu_2_O nanoparticles prepared using electrospinning	the membranes induce bactericidal activity on *E. coli*	filtration membranes	(760)
Gr	PVDF membranes loaded with Gr prepared through a phase inversion method	the membranes showed antifungal properties against *Curvularia sp*, owed to synergistic result of Gr toxicity and surface topography	seawater desalination membranes	(761)
Ag nanoparticles-GO hybrid nanosheet (AgNPs-GO)	the modification of the H-PVDF membrane was carried out through the polymerization of PVA and AgNPs-GO nanosheet using glutaraldehyde as the cross-linking agent	the membranes induced 100% inactivation of *Pseudomonas aeruginosa* in solution and 91% reduction in the membrane surface adhesion	filtration membranes	(524)
CdS/MIL-101 photocatalys and metal–organic frameworks (MOF)	CdS/MIL-101 modified PVDF membranes prepared by a phase inversion method via immersion precipitation	an inhibition rate of 92% for *Escherichia coli* and 95% for *Staphylococcus aureus* when irradiated by light	filtration membranes	(762)
functionalized nanodiamonds (NDs)	films comprising NDs processed by solvent casting	the films possessed antimicrobial and antifouling properties toward *E. coli*	general antimicrobial material	(763)
mesoporous graphitic carbon nitride (MCN) photocatalyst	the blending of MCN in PVDF was performed through immersion-precipitation phase transformation	MCN_80_-PVDF membrane achieved 3 log of *E. coli* inactivation under visible light irradiation for 4 h	filtration membrane for the treatment of real wastewater	(764)
TiO_2_ nanoparticles surface functionalized with Ag nanoparticles	poly(VDF-*co*-HFP) comprising the NPs were prepared using solvent casting or electrospinning	certain antimicrobial activity was attained against *E. coli* and *S. epidermidis*	multifunctional material for environmental remediation	(556)

Nonetheless, the possibility of piezoelectric polymers such as PVDF to take part on the antimicrobial process due to the possibility of these polymers to create EAMs on their surface when mechanically stimulated is a growing field of research. These are novel strategies that recently started to bloom and are interesting due to the possibility of piezoelectric materials to respond to mechanical cues present in our everyday life: touch, pressure, walk, run, and/or vibration. These technologies are even more appealing if we consider the fact that they avoid the emergence of resistant strains. The use of physical stimuli as an alternative to chemical compounds such as antibiotics mask the real “attacker” from recognition by the bacteria,^721^ which ultimately poses less evolutionary stress to bacteria and thus avoid AMR.

Understanding bacteria sensing mechanisms is essential to develop effective advanced approaches. Despite being an extremely simple organism, bacteria possess a remarkable capacity to develop resistance as a mechanism of survival and adapt to different environments, tolerating a big range of temperatures, pressures, and pHs. This is a result of their long evolutionary history, being exposed to vastly different physicochemical environments and being able to detect and respond to a wide range of signals such as chemical, thermal, mechanical, electrical, and magnetic fields. Nevertheless, this capacity for adaptation and sensing physical signals have been largely overlooked in bacterial cells.

On the other hand, it has been proven that mammalian cells feel the surrounding environment and respond to a range of physical signals. Therefore, a large amount of research has been performed on the piezoelectric effect present in polymers such as PVDF and its copolymers^673,689,693,722^ or even in natural piezoelectric polymers such as silk^723−725^ for triggering and enhancing cell responses such as adhesion, migration, differentiation, or proliferation. These materials allow the development of dynamic EAMs on their surface when mechanically stimulated and have been proven to enhance cell target functions, thus being successfully applied in regenerative medicine.^726−728^ When piezoelectric materials are used, besides the electrical cues, mechanical forces are also provided to the cells and tissues, which ultimately affect biological entities in a process called mechanotransduction.^729^ In this process, cells and tissues react to mechanical stimuli and translate them into biochemical and biological responses, as explained in the previous section.^668,730^ The potential of using these stimuli have thus been widely researched for tissue engineering applications but poorly investigated for antimicrobial strategies.

This is paradoxical if the natural environment of microorganisms is take into consideration, which is in constant contact with mechanical forces, generated by fluid flow, shear stress, or through the interaction with the surface of other cells or materials^731^ (Figure 48b,c). Predominantly, outside the oceans, bacteria live in community, attaching to surfaces through the secretion of adhesive structures such as pili and flagella, also growing in the form of a protective biofilm, encased in a structure composed of exopolysaccharides (Figure 48b). Such mode of growth is seen as a protective way to survive to harsh environment, imparting resistance to bacteria. Diffusible signaling molecules are also used by bacteria to communicate and interact in community in a processed called quorum sensing. Mechanisms able to disrupt these structures/mechanisms are ideal to obtain an efficient antimicrobial purpose and physically based approaches have been showing a clear potential. This is valid if we consider that bacteria are subjected to interface mechanics that include hydrodynamic and adhesive forces as well as the rheology of their surroundings.^731^ In fact, the mechanical environment that a bacterium feels when attached to a surface is completely different than when in planktonic state.

When attached in a dry environment, the bacteria experience a local force identified as adhesive force but when a liquid flow is present the viscosity of the fluid creates a shear force in the same direction of the flow. Bacteria also feel the rheological properties of their surrounding extracellular matrix, which flows and/or deforms upon application of forces. Additionally, the transport of soluble compounds secreted or released by bacteria within biofilm communities can be highly affected by the fluid flow (advection) and Brownian motion (diffusion) (Figure 48c).

In fact, one of the first reports on how bacteria are able to feel their external environment through physical cues has led to the conclusion that they act in a similar way to that of mammalian cells, being activated by mechanical forces in a mechanotransduction process.^732^ This topic is extremely interesting because bacteria possess vast sensing systems whose input signals remain unidentified, some of which could potentially participate in transducing forces into a developmental response.^733^

The bacterial mechanotransduction may be regarded as the succession of three elementary events: mechanotransmission, mechanosensing, and mechanoresponse^734^ (Figure 48d). Mechanotransmitting components are structures that bear and propagate the applied force. These are coupled with mechanosensors that modulate their biochemical activity in response to transmitted forces. Finally, mechanosensors induce a downstream mechanoresponse by modulating specific developmental programs.

Examples of mechanotransduction systems in bacteria may include the movement of cilia of certain microorganisms that transmit forces to membrane-associated mechanosensitive proteins. This result in mechanoresponses ranging from rapid influx of ions through membranes, transcriptional regulation, or morphogenesis.^734^

The motility capacity of bacteria, a flagellum-dependent form of movement observed in some bacterial species, is an example of bacterial mechanotransduction. For instance, bacteria causing gonorrhea, the *Neisseria gonorrheae*, possess retractable polymer type IV pili on their surface that exert forces at nN range on their surroundings, the same amplitude of forces that mammalian cells exert on their own microenvironments.^735^ These forces trigger events such as accumulation of actin and other proteins, which are critical for the colonization of the host.^736^ The ability of *Pseudomonas aeruginosa* to form biofilm is also activated by a mechanotransduction system, through a process that includes a surface-specific twitching motility machinery. Using this process, *P. aeruginosa* disarms predatory cells by injecting toxins upon contact with a host, activating specific transcriptional programs upon surface contact.^737^ More recently, it has been found that a mechanotransduction phenomenon also occurs in *E. coli*. The mechanism of action involves sensing the local mechanical environment through voltage-induced calcium flux, which causes the influx of calcium ions causing an electricity pulse through them.^738^

Currently, apart from mechanical stimuli, one of the most used physical stimuli for antimicrobial strategies is the application of electrical charges. It has been regarded as a mean for preventing device-related infections, which are caused by biofilm formation, or even to disinfect contaminated liquids.^739−743^ Nevertheless, these strategies require the direct application of a strong electrical fields which limits biomedical applicability.

The electrical-based killing mechanism of action involves cell membrane permeability increase, known as electropermeabilization. This occurs when the induced transmembrane voltage exceeds the threshold voltage (200–1000 mV), while the resting transmembrane potential ranges between −20 and −200 mV for most cells.^744^ For keeping the resting membrane potential nearly constant, the Na/K pump actively exudes three Na^+^ for every two K^+^ pumped into the cell. The perturbation of these ion concentrations can lead to hyperpolarization, wherein the membrane potential becomes more negative, or depolarization, wherein the membrane potential becomes less negative toward zero.^745^ The formation of ROS such as H_2_O_2_ and reactive nitrogen species (RNS) has also been indicated as a possible mechanism of action for the bactericidal effect of electric fields.^648,746^ These mechanisms however are not completely well-founded and may differ from Gram-positive and Gram-negative bacteria and thus should be investigated. Indeed, the knowledge of a drug’s mechanism of action enables better monitoring of its effects on the target pathway.

**Figure 49:**
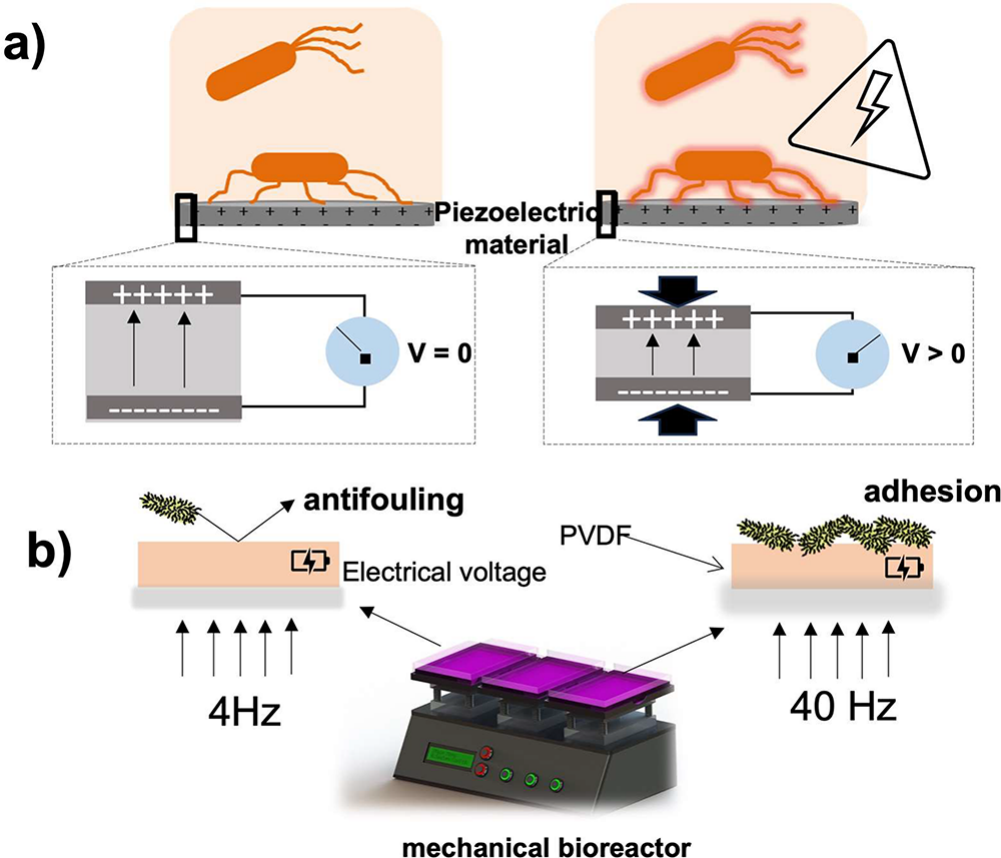
Schematic representation of the: (a) formation of electroactive microenvironments (EAMs) based on a piezoelectric material, depicting the impact of mechanical stimuli application on bacteria and (b) EAMs created by a mechanical bioreactor on a piezoelectric PVDF polarized scaffold, inducing different responses on bacterial cells including proliferation or growth inhibition/antifouling properties, depending on the frequency applied.

Recently, novel strategies based on the application of EAMs for antimicrobial strategies have been emerging. EAMs apply very low electrical voltage to the surrounding of cells, not posing a problem when biomedical applications are needed. EAMs may be induced on the bacterial cells using electroactive, piezoelectric, or magnetoelectric materials (Figure 49a).

Porous material may be important to increase the contact area between material and bacteria, providing better stimuli, while the nonporous material may assist on antifouling effect.^747^

One of the first reports emphasizing the effect of a piezoelectric material for killing bacteria was performed by Tan et al.^748^ Upon polarization of a piezoelectric ceramic, which results in a material with one side positively charged and the other one negatively charged, it has been found that the positively charged surface selectively killed bacteria through the formation of ROS owed to the microelectrolysis of water. The work suggested that the level of ROS necessary to kill bacteria was safe for normal mammalian cells.^748^

Recently, polarized PVDF was used for proving the concept of antimicrobial strategies under dynamic conditions, i.e., using a mechanical bioreactor, for the creation of EAMs on the surface of the material.^648,649,749^ It shown that bacterial cells behavior can be tailored depending on the surface charge of PVDF and on the application of EAMs, promoted by the stimulation of a PVDF poled film. The results showed a different behavior between Gram-positive and Gram-negative cells. The conditions had little effect on *E. coli*, but an antifouling effect was observed on *S. epidermidis* at static conditions. On the other hand, at dynamic conditions, i.e., in the presence of an electrical stimuli, the lower frequency promoted antifouling effect, while at higher frequencies bacteria adhesion is stimulated (Figure 49b). This was the first work proving the concept of bacteria susceptibility to physical stimuli. These strategies are important to further define suitable anti- and pro-microbial strategies intended for pathogenic and functional bacteria, respectively.

The multifunctional nature of bacteria can thus be explored to develop novel strategies to tailor the bacterial response by exposing them to EAMs. Bactericidal, antifouling, or sensitization effects on pathogenic bacteria may be sought with these new approaches, which exerts less evolutionary stress and thus avoid the occurrence of resistance mechanisms. It has been also shown that poly(VDF-*co*-TrFE) films composites sensitize the bacteria to low doses of green-synthesized Ag nanoparticles, previously dispersed in the polymer matrix. When stimulated at a mechanical frequency of 4 Hz more than 80% the *S. epidermidis* bacterial growth was reduced in planktonic and biofilm form, allowing to conclude that EAMs sensitize the bacteria for the action of a low dose of Ag nanoparticles (1.69% (wt %/wt %)), without compromising the viability of mammalian cells.^649^

The possibility for remote stimulation of a material though the EAMs and thus obtaining antimicrobial effects can also be achieved using magnetoelectric materials. This approach could also be valuable, for example, for the prevention of infection of orthopedic indwelling devices by external stimulation. Magnetostrictive nanostructures may be embedded in piezoelectric polymers to obtain magnetoelectric materials. When magnetically stimulated, the magnetostrictive component alters its size due to elastic deformation, which induces a mechanical stimulus on the piezoelectric polymer phase, further inducing an electrical polarization variation of the polymer. Magnetoelectric materials comprising magnetostrictive nanowires aligned within the polymer matrix may be used to obtain an anisotropic material and induce a strong EAMs. These materials allow to remotely stimulate tissues from outside of the human body.^750,751^

**Figure 50:**
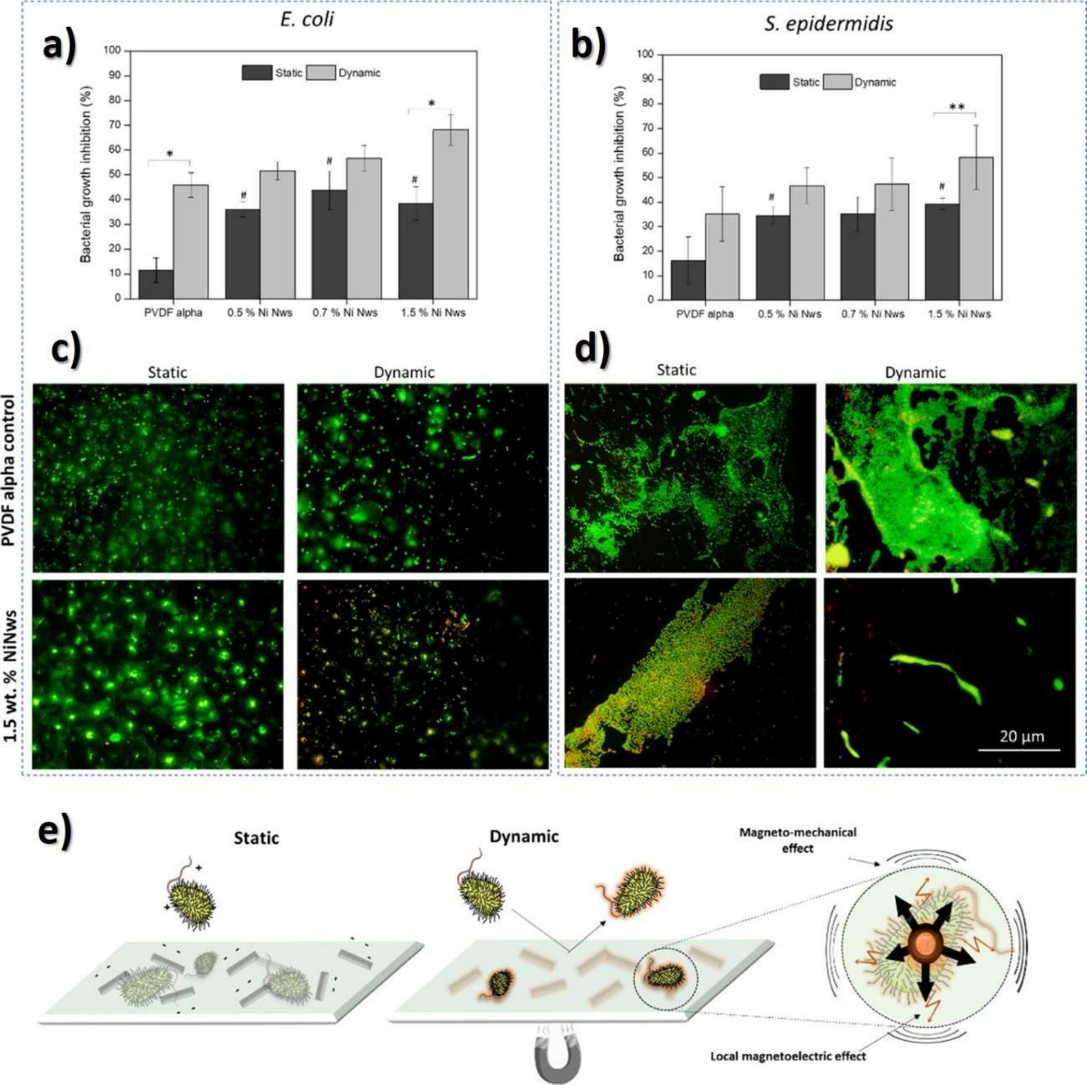
Bacterial growth inhibition of *E. coli* (a) and *Staphylococcus epidermidis* (b) in suspension in the presence of the control film (α-PVDF) and the NiNws loaded PVDF films with and without magnetic field application. *P < 0.01, **P < 0.1 when compared with each other, and #*P* < 0.01 when compared to the control sample α-PVDF at static conditions. Fluorescence microscopy live/dead images of *E. coli* (c) and *S. epidermidis* (d) incubated over the nanocomposites (PVDF with 1.5 wt % NiNws) and α-PVDF as a control for 16 h at static and magnetic bioreactor-assisted dynamic conditions. Live cells are represented in green and dead cells in red. Scale bars denote 20 μm for all images. (e) Schematic representation of magnetoelectric PVDF/NiNws material in contact with bacteria at static and dynamic conditions. Representation of magnetomechanical and local magnetoelectrical effects upon application of magnetic stimuli and its effect on bacterial cells. Reproduced with permission from ref (650). Copyright 2021 American Chemical Society.

This approach has been developed^650^ based on PVDF films filled with nickel nanowires (NiNws) in an attempt to control and enhance the antimicrobial activity of the materials via a magnetic stimulus. More than 55% of bacterial growth inhibition was achieved by controlled dynamic magnetic conditions for representative Gram-positive and Gram-negative bacteria, compared to only 25% inhibition obtained under static conditions, i.e., without magnetic stimuli application, with the antibiofilm activity clearly improved as well upon dynamic conditions (Figure 50).

The works herein described successfully demonstrated the proof-of-concept for materials able to boost on demand antimicrobial activity upon dynamic conditions (mechanically or magnetically stimulated) and paves the way for applications as coatings in high-touch and high-traffic surfaces to restrain bacterial adhesion and proliferation and thus provide infection control. Thus, it is expected that PVDF will play a significant role in the development of antimicrobial materials that can effectively respond to various stimuli encountered in our daily lives, such as touch, vibrations, walking, jumping, and more. Importantly, when PVDF is mechanically stimulated, it can overcome several limitations associated with currently used antimicrobial agents. These include: (i) Resistance development: Preliminary results indicate that electroactive microenvironments (EAMs) formed by PVDF do not induce resistance in bacteria. Instead, they act on highly evolved bacterial membranes by inducing depolarization, making it a promising approach to combat resistance development. (ii) Spectrum of activity: EAMs generated by PVDF do not discriminate between gram-positive and gram-negative bacteria. This broad-spectrum activity is advantageous as it can effectively target a wide range of bacterial species, enhancing its overall effectiveness. (iii) Toxicity concerns: The low levels of voltage induced by PVDF do not pose any cytotoxicity risks to mammalian cells. Additionally, PVDF itself has been extensively proven to be biocompatible, alleviating concerns about potential toxicity issues when used in biomedical applications; (iv) Material degradation: PVDF exhibits high stability and resilience, making it resistant to degradation, damage from cleaning procedures, and exposure to various environmental factors. No leachable components have. been observed, ensuring its long-term effectiveness and safety.

Thus, PVDF demonstrates great potential as an antimicrobial material, offering improved performance and compatibility with biological systems. Its unique properties make it a promising candidate for the development of advanced biomedical applications.

Table 16 summarizes relevant PVDF-based materials for antimicrobial purposes, indicating the antimicrobial agent, the functionalization, main results in terms of antibacterial activity, as well as the main focused application.

## 4 Conclusions and Future Perspectives

Poly(vinylidene fluoride), PVDF, is a polymer known for its excellent chemical, thermal, mechanical and radiation resistance, as well as for their outstanding electroactive properties among polymers, including high dielectric, piezoelectric, pyroelectric, and ferroelectric response, when the polymer crystallizes in specific phases.

PVDF crystallizes in five distinct polymorphs: α (TGTG′ (trans-gauche–trans-gauche)), β (plane zigzag all trans TTTT), δ (TGTG′), γ (T3GT3G′), and ε (T_3_GT_3_G′), α- and ε-PVDF being nonpolar, whereas β-, δ-, and γ-PVDF are polar crystalline phases.

PVDF and its copolymers, including poly(vinylidene fluoride-*co*-trifluoroethylene, poly(PVDF-*co*-TrFE), poly(vinylidene fluoride-*co*- hexafluoropropylene), poly(VDF-*co*-HFP), poly(vinylidene fluoride-*co*-chlorotrifluoroethylene), and poly(PVDF-*co*-CTFE), can crystallize in electroactive crystalline phases, and therefore are increasingly explored for a wide range of applications.

Poly(VDF-*co*-TrFE) copolymer is one of the most studied copolymers of PVDF as, when synthesized in specific copolymer contents, it always crystallizes in the crystallographic β phase, because the addition of the third fluorine atom in the TrFE monomer unit induces the ferroelectric β phase regardless of the processing method, either from the melt or from solution. In addition, poly(VDF-*co*-TrFE) shows high piezoelectric output, sensitivity, and wide frequency response with great potential for applications ranging from sensors/actuators to biomedical applications.

Poly(VDF-*co*-HFP) shows the highest piezoelectric constant *d*_31_ (21 pC·N^–1^) and a lower degree of crystallinity compared to PVDF, thus intensively used for energy storage applications, in particular for separator membranes and solid polymer electrolytes (SPE).

Finally, the properties of poly(VDF-*co*-CTFE) are dependent on the CTFE content, show a high electrostrictive strain response, and it is mostly used in energy storage applications.

PVDF and copolymers can be processed into a variety of morphologies, including dense thin films, porous membranes, fibers, microspheres, or specific patterns, among others. Further, the materials can be processed either from the melt or from solutions, by techniques including extrusion, doctor blade, electrospray, electrospinning, or additive manufacturing techniques, among others.

Based on those physicochemical and processing characteristics, PVDF and copolymers have been used for the development of a large variety of applications in areas including, sensors, actuators, energy harvesting, and storage systems, as well as membranes for environmental remediation and biomedical applications. For these applications, the control of morphology, polymer phase, degree of crystallinity and phsicochemistry electrical, mechanical, and electroactive properties is essential to obtain systems and devices with tailored functional performance.

This review presents an overview on PVDF and its copolymers as well as on their applications. The correlation between polymer chain conformation, processing, materials physicochemical characteristics and integrability is essential for achieving optimized performance within the different applications. For the main applications presented and discussed in this review, the challenges and future trends of PVDF-based materials are summarized in the following.

For sensors applications, PVDF-based materials mainly rely on the processing of the material in the electroactive β-phase and in optimizing the electroactive response, either piezo- or pyroelectric. Further, high dielectric constant and novel effects, such as magnetoelectricity, are obtained though the development of polymer composites, which are increasingly investigated. Challenges involve the fabrication of electroactive devices through additive manufacturing techniques that will allow low-cost, low-waste, high performance, and more sustainable systems with improved integration and free-form, allowing from micrometer-size to large area solutions. The main limitations of PVDF-based sensors are the still low electroactive response with respect to their ceramic counterparts; the introduction of specific fillers, including ceramics, mesoporous material, and ionic liquids (ILs) fillers allows addressing of this issue, but proper dispersion and stability over time must be achieved. Nevertheless, the flexibility, integrability and resilience of the materials make PVDF-based sensors an essential tool in the development of bionic robots, communication, and augmented reality gadgets, as well as for the implementation of smart environments, including smart cities and industry 4.0 solutions.

Regarding actuator applications, further developments are needed to improve actuator deformation in electronic actuators and actuator force in ionic ones, as well as to enhance stability over time in the latter. These improvements can rely on ternary composites with two different fillers, allowing improvement of functional response and stability over time simultaneously where interface engineering will be an important contribution.

Polymer composites are also essential to improve energy harvesting and SPE for energy storage systems. PVDF-based materials are increasingly used in energy harvesting and storage applications. With respect to energy harvesting systems, the focus should be on improving delivered power to integrate self-power sensors in various applications, including wearables and sensors and actuators in remote or difficult to access locations. In addition, it is essential to understand the interactions between fillers and polymer matrix for improving the device performance, stability over time, and to allow suitable processing by additive manufacturing techniques.

For energy storage systems, and as an overall challenge for PVDF-based materials, green chemistry synthesis and advanced recovering, recycling, and/or reuse strategies are necessary, considering that PVDF is the best material for applications in electrodes as a polymer binder. Furthermore, it is essential to study and understand the nanoscale interaction of the polymer with the electrolyte solution to reduce the amount of polymer binder and increase electrochemical performance. Concerning separator membranes and SPE, the improvement of the interaction with the electrolytes and fillers and the improvement of the ionic conductivity should still be addressed as well as to tune the interfaces with the electrodes through surface compatibilization.

In the area of environmental remediation, the excellent membrane forming characteristics, porosity, and pore size tunability, together with the chemical and radiation resistance, makes PVDF and copolymers one of the most suitable materials, the main challenge remaining the incorporation of specific fillers to match specific pollutants degradation and/or absorption requirements. Moreover, multifunctional materials such as adsorptive and photocatalytic or antimicrobial membranes will allow more robust membrane technologies to address a broader range of pollutants (organic, inorganic, and water disinfection). Stable composite membranes with different active layers will also allow avoiding or minimizing secondary pollution caused by the detachment and discharge of active materials like nanoparticles into natural water bodies. Improving the integration of the fillers into the polymer matrix and the regeneration of the membranes after use is essential to allow long-term reusability. In parallel, additive manufacturing will undoubtedly contribute to develop advanced multifunctional materials, mainly because of the possibility of producing tunable morphologies and functionalities with a layer-by-layer design.

Concerning microfluidics and portable analytical devices for biomedical, environmental, biodefense, and food monitoring applications, the main challenge is to implement sensors and actuators, among other operation systems, in order to allow the development of complete, autonomous, and more user-friendly platforms for specific applications. In this sense, electroactive polymer-based materials, such as PVDF and its copolymers, are suitable for microfluidic applications based on their biocompatibility, flexibility, low mechanical and acoustic impedance, and controlled optical transparency, critical parameters in many microfluidic applications. Moreover, they are easily integrated or can even be directly printed on the microfluidic platform to work not just as actuation or sensing systems but also as smart tailorable membranes to produce portable, cost-effective, and smart miniaturized platforms. Still, a road ahead of intense and dynamic research is required to obtain improved functionality and to address some of the most challenging microfluidic applications requirements, e.g., complex multistep analysis, with the ultimate goal of promoting their quicker standardization and further commercialization.

For biomedical applications, PVDF-based materials have proven their suitability for developing active electromechanical cell microenvironments for advanced tissue engineering strategies of various tissues, including bone, heart, skin, and neuronal, among others. Nevertheless, the proper combination of PVDF and copolymers with biochemical factors and the tailorability of the microstructure for specific 2D and 3D tissue microenvironments are a pivotal challenge to reach regeneration strategies translated to clinical use.

The possibility of using a versatile polymer such as PVDF for antimicrobial solutions, taking advantage of the physically and chemical stability of the polymer as well as the possibility of imparting stimuli (mechanically)-responsive properties to it, may change the paradigm of antimicrobial research. Smart antimicrobial materials that inactivate bacteria upon daily actions such as touch, vibration, or walking will undoubtedly pave the way for a new generation of materials to be applied in common high-traffic surfaces in clinical settings, schools, or domestic environments. Even though initial assessment indicates that the physical stimuli provided by piezoelectric materials avoid the emergence of resistant strains, a systematic study on their mechanism of action and resistance development is still needed to infer without any doubt that they avoid the undesired phenomenon of antimicrobial resistance.

Overall, PVDF, its copolymers, composites, and blends show a large variety of outstanding physicochemical characteristics, with a particular focus on the electroactive ones, that will certainly play an essential and increasing role in the ongoing energy transition, the digitalization of society and economy, and the new biomedical approaches. Precisely tailoring material characteristics and functional response, integration into devices and properly addressing the sustainability of materials and fabrication processes are critical challenges to further expand the limits of technologies and applications based on this extraordinary family of polymers.

## Abbreviations

3D: three-dimensional
AA: ascorbic acid
Ag: silver
Al: aluminum
ALP: alkaline phosphatase
AMR: antimicrobial resistance
As: arsenic
Au: gold
BaTiO_3_: barium titanate
BSA: bovine serum albumin
CA: cellulose acetate
CH: chlorhexidine
CIP: ciprofloxacin
CMC: carboxymethyl cellulose
CMOS: complementary metal-oxide semiconductor
CNTs: carbon nanotubes
CoFe_2_O_4_: cobalt ferrite
Cr: chromium
DA: dopamine
DE: dielectric elastomers
DIW: direct-ink-writing
DMA: *N*,*N*-dimethyl acetaminde
DMF: *N*,*N*-dimethylformamide
DMMP: dimethyl methyl phosphonate
DMPU: *N*,*N*′-dimethylpropyleneurea
DMSO: dimethyl sulfoxide
DXL: 1,3-dioxalane
EAC: electroactive ceramics
EAMs: electroactive microenvironments
EAP: electroactive polymers
EMI: electromagnetic interference
EPA: Environmental Protection Agency
EPANI: poly(2-ethyl aniline)
ESM: electrochemical strain microscopy
Fe_3_O_4_: Iron oxide
FE-PE: ferroelectric-paraelectric
FOM: figures of merit
FTIR: Fourier-transform infrared spectroscopy
GO: graphene oxide
GPS: global positioning system
Gr: graphene
ILs: ionic liquids
IoT: internet of things
IPMC: ionic polymer–metal composite
LATP: lithium aluminum titanium phosphate
LB: Langmuir–Blodgett
Li: lithium
LiClO_4_: lithium perchlorate
LiF: lithium fluoride
Li-S: lithium-sulfur
LiTFSI: lithium bis(trifluoromethanesulfonyl)imide
MB: methylene blue
MEMS: microelectromechanical systems
MOFs: metal–organic frameworks
MWCNTs: multiwalled carbon nanotubes
NG: nanogenerator
Ni: nickel
NIPS: nonsolvent-induced phase separation
NIR: near-infrared
NMP: *N*-methyl-2-pyrrolidone
PANI: poly(aniline)
PBS: phosphate-buffered saline
PC: poly(carbonate)
PCL: polycaprolactone
Pd: palladium
PDMS: polydimethylsiloxane
PEDOT:PSS: poly(3,4-ethylenedioxythiophene)-poly(styrenesulfonate)
PEO: poly(ethylene oxide)
PET: poly(ethylene terephthalate)
PHBV: poly(3-hydroxybutyrate-*co*-3-hydroxyvalerate)
PLGA: poly(lactic-*co*-glycolic acid)
PLLA: poly(l-lactic acid)
PMMA: poly(methyl methacrylate)
PNP: *p*-nitrophenol
POC: point-of-care
poly(VDF-*co*-CTFE): poly(vinylidene fluoride-*co*-chlorotrifluoroethylene)
poly(VDF-*co*-HFP): poly(vinylidene fluoride-*co*-hexafluoropropylene)
poly(VDF-*co*-TrFE): poly(vinylidene fluoride-*co*-trifluoroethylene)
poly(VDF-*ter*-TrFE-*ter*-CFE: poly(vinylidene fluoride-*ter*-trifluoroethylene-*ter*-chlorofluoroethylene
PPy: polypyrrole
PRP: platelet-rich plasma
PS: polystyrene
Pt: platinum
PTFE: poly(tetrafluoroethylene)
PU: poly(urethane)
PVA: poly(vinyl alcohol)
PVB: polyvinyl butyral
PVC: poly(vinyl chloride)
PVDF: poly(vinylidene fluoride)
PVP: poly(vinyl pyrrolidone)
PZT: lead zirconate titanate
rGO: reduced graphene oxide
ROS: reactive oxidative species
SEI: solid electrolyte interface
SEM: scanning electron microscopy
SiO_2_: silicon dioxide
SMA: shape memory alloys
SMG: surface-modified graphene
SO_2_: sulfur dioxide
SPE: solid polymer electrolyte
SWCNT: single-walled carbon nanotubes
TEM: transmission electron microscopy
Ti: titanium
TiO_2_: titanium dioxide
TIPS: temperature-induced phase separation
V: vermiculite
VDF: vinylidene fluoride
WHO: World Health Organization
ZnO: zinc oxide
